# Supplementary material for: Unlocking the Potential of Human-Induced Pluripotent Stem Cells: Cellular Responses and Secretome Profiles in Peptide Hydrogel 3D Culture
Source: Cells. 2024 Jan 12;13(2):143. doi: 10.3390/cells13020143 (PMC10814310; doi:10.3390/cells13020143)

Supplementary Figures

Unlocking the Potential of Human-Induced Pluripotent Stem Cells: Cellular Responses and Secretome Profiles in Peptide Hydrogel 3D Culture

Muyun Cui[1], Wei Wu[2,†] ,Quan Li[3,†], Guangyan Qi[1], Xuming Liu[4], Jianfa Bai[5], Mingshun Chen[4], Ping Li[2], Xiuzhi (Susan) Sun[1,3,*]

Author affinitions:

1 Department of Grain Science and Industry, Kansas State University, Manhattan, KS 66506, USA

2 Department of Chemistry, Kansas State University, Manhattan, KS 66506, USA

3 Carl and Melinda Helwig Department of Biological and Agricultural Engineering, Kansas State University, Manhattan, KS 66506, USA

4 Department of Entomology, Kansas State University, KS, 66506, USA

5 Department of Diagnostic Medicine/Pathobiology, College of Veterinary Medicine, Kansas State University, Manhattan, KS 66506, USA

† Authors with equal contributions as the second author.

***Corresponding authors: xss@ksu.edu**

Supplemental Figure S1. Different visualization method for Gene ontology (GO) analysis based on the mass spectrometry results of medium collected from hiPSC-P and hiPSC-F cultured in mTeSR1 based systems, showing the target gene categories and counts.

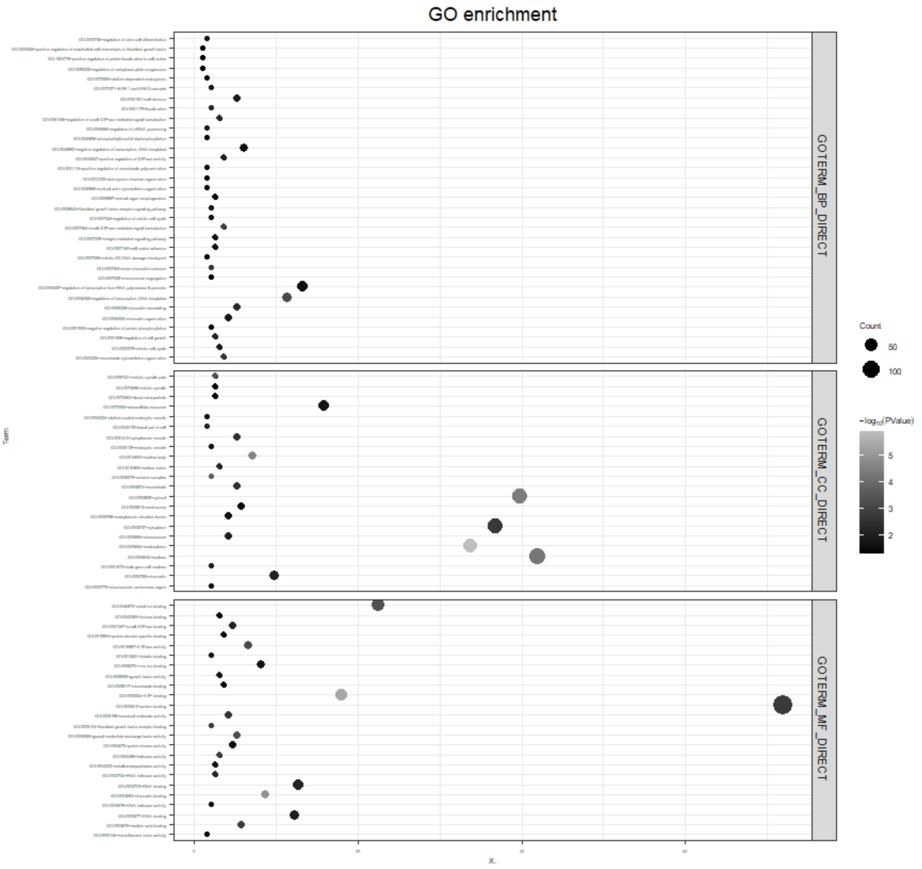

Supplemental Figure S2. Different visualization method for GO analysis based on the mass spectrometry results of medium collected from hiPSC-P and hiPSC-F cultured in E8 based system, showing the target gene categories and count.

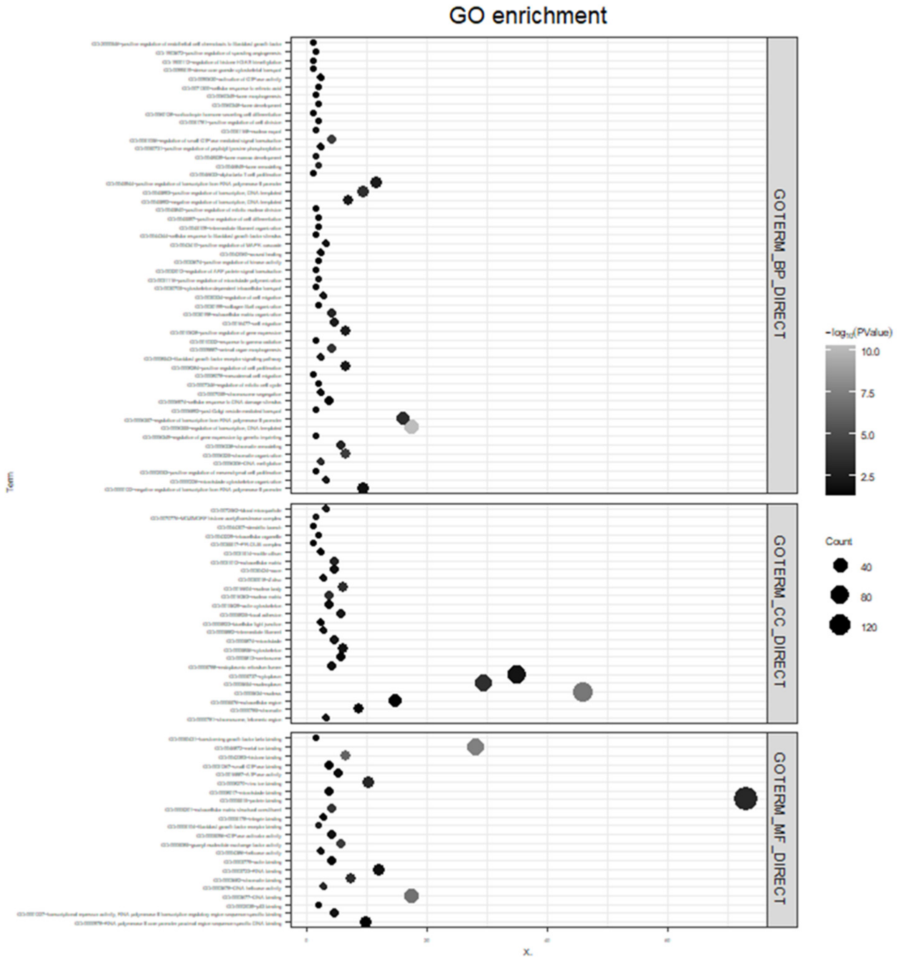

Supplement: Supplementary file 1 [file cells-13-00143-s001.zip › Supplementary Figures.pdf]
