# Supplementary material for: Unlocking the Potential of Human-Induced Pluripotent Stem Cells: Cellular Responses and Secretome Profiles in Peptide Hydrogel 3D Culture
Source: Cells. 2024 Jan 12;13(2):143. doi: 10.3390/cells13020143 (PMC10814310; doi:10.3390/cells13020143)
Supplement: Supplementary file 1 [file cells-13-00143-s001.zip › Supplementary Table S1. Proteins profile of 3D hiPSCs cultured in mTeSR1.pdf]

Supplementary Table S1. Proteins profile of 3D hiPSCs cultured in mTeSR medium

Accession	Peptide count	Unique peptides	Confidence score	Anova (p)	q Value	Max fold change	Power	Highest mean condition	Lowest mean condition	Mass	Description
Q99795	35	27	196.0983	1.608E-09	6.464E-11	1.591041	1	P-mT-Susp	mT blank	36259.29	Cell surface A33 antigen OS=Homo sapiens OX=9606 GN=GPA33 PE=1 SV=1
P02768;A0A0	35	24	279.9827	1.863E-07	4.053E-09	1.922179	1	P-mT-Susp	mT blank	71362.71	Albumin OS=Homo sapiens OX=9606 GN=ALB PE=1 SV=2
O75326	35	22	234.0038	5.814E-12	7.714E-13	2.235417	1	P-mT-Susp	P-0.5PG-mT	75907.39	Semaphorin-7A OS=Homo sapiens OX=9606 GN=SEMA7A PE=1 SV=1
P02787;C9JVG	22	19	247.1295	1.529E-09	6.243E-11	2.869405	1	P-mT-Susp	mT blank	79345.08	Serotransferrin OS=Homo sapiens OX=9606 GN=TF PE=1 SV=3
P18065	23	17	116.3566	5.701E-06	9.927E-08	1.409829	1	F-0.5PG-mT	F-mT-2D	35897.68	Insulin-like growth factor-binding protein 2 OS=Homo sapiens OX=9606 GN=IGFBP2 PE=1 SV=2
A0A0C4DFM7	18	17	106.7504	3.035E-10	1.611E-11	2.387918	1	P-mT-Susp	mT blank	188218.4	Terminal uridylyltransferase 4 OS=Homo sapiens OX=9606 GN=TUT4 PE=1 SV=1
A0A2U3TZM0	26	17	175.1846	5.807E-11	4.320E-12	3.232572	1	P-mT-Susp	mT blank	218420.6	DNA helicase OS=Homo sapiens OX=9606 GN=CHD4 PE=1 SV=1
P04264;A0A1	20	15	169.012	3.247E-04	4.386E-06	1.808992	0.99939	P-0.5PG-mT	F-0.5PG-mT	66209.9	Keratin_ type II cytoskeletal 1 OS=Homo sapiens OX=9606 GN=KRT1 PE=1 SV=6
Q86UP2;G3V4	19	14	115.0122	3.668E-10	1.814E-11	2.583584	1	P-mT-Susp	mT blank	156560.6	Kinectin OS=Homo sapiens OX=9606 GN=KTN1 PE=1 SV=1
Q8NDI1;B5M	16	14	86.038	2.033E-11	2.048E-12	3.315162	1	P-mT-Susp	mT blank	140644.8	EH domain-binding protein 1 OS=Homo sapiens OX=9606 GN=EHBP1 PE=1 SV=3
A0A669KB77;	19	11	148.2589	6.019E-02	6.637E-04	1.149354	0.61749	F-mT-2D	F-0.5PG-mT	217419.6	Microtubule-associated protein OS=Homo sapiens OX=9606 GN=MAP2 PE=1 SV=1
P38398;A0A2	15	11	98.9662	3.223E-09	1.182E-10	1.564146	1	P-0.5PG-mT	mT blank	210230.3	Breast cancer type 1 susceptibility protein OS=Homo sapiens OX=9606 GN=BRCA1 PE=1 SV=2
O76093	15	10	102.5651	2.599E-08	7.118E-10	2.090961	1	P-mT-Susp	mT blank	24330.89	Fibroblast growth factor 18 OS=Homo sapiens OX=9606 GN=FGF18 PE=1 SV=1
A0A087WY61	13	10	84.3613	1.601E-01	1.711E-03	1.633045	0.41944	mT blank	F-mT-2D	237531.4	Nuclear mitotic apparatus protein 1 OS=Homo sapiens OX=9606 GN=NUMA1 PE=1 SV=1
Q9H9B1;A0A1	13	10	79.8177	3.571E-10	1.777E-11	3.067795	1	P-mT-Susp	mT blank	144089.8	Histone-lysine N-methyltransferase EHMT1 OS=Homo sapiens OX=9606 GN=EHMT1 PE=1 SV=4
Q9H1A4	10	10	52.5067	2.308E-07	4.939E-09	1.766734	1	P-mT-Susp	mT blank	218667.5	Anaphase-promoting complex subunit 1 OS=Homo sapiens OX=9606 GN=ANAPC1 PE=1 SV=1
O43320	11	10	62.9362	3.148E-10	1.638E-11	11.05664	1	P-mT-Susp	P-0.5PG-mT	23872.9	Fibroblast growth factor 16 OS=Homo sapiens OX=9606 GN=FGF16 PE=1 SV=1
P31946	12	10	70.8095	4.037E-11	3.348E-12	2.73753	1	F-mT-2D	mT blank	28196.51	14-3-3 protein beta/alpha OS=Homo sapiens OX=9606 GN=YWHAB PE=1 SV=3
Q8TEU7;E9PC	12	9	81.3449	3.272E-03	3.941E-05	2.192957	0.96654	mT blank	P-mT-Susp	180962.9	Rap guanine nucleotide exchange factor 6 OS=Homo sapiens OX=9606 GN=RAPGEF6 PE=1 SV=2
Q9NZM3;A0A	10	9	51.0109	3.920E-09	1.369E-10	1.692759	1	P-mT-Susp	F-0.5PG-mT	194545.3	Intersectin-2 OS=Homo sapiens OX=9606 GN=ITSN2 PE=1 SV=3
Q9UIF8	10	9	46.0891	1.712E-04	2.387E-06	2.953753	0.99988	P-0.5PG-mT	P-mT-Susp	242227.4	Bromodomain adjacent to zinc finger domain protein 2B OS=Homo sapiens OX=9606 GN=BAZ2B PE=1
Q5TCS8;J3KP8	12	8	79.8553	1.791E-04	2.484E-06	1.640973	0.99986	P-mT-Susp	mT blank	222838.6	Adenylate kinase 9 OS=Homo sapiens OX=9606 GN=AK9 PE=1 SV=2
Q96N67	8	8	33.193	2.314E-03	2.825E-05	1.230843	0.97878	P-mT-Susp	P-0.5PG-mT	244443	Dedicator of cytokinesis protein 7 OS=Homo sapiens OX=9606 GN=DOCK7 PE=1 SV=4
Q12789;I3L1Z	9	8	54.2936	3.890E-07	8.023E-09	2.990492	1	P-0.5PG-mT	P-mT-Susp	241213.3	General transcription factor 3C polypeptide 1 OS=Homo sapiens OX=9606 GN=GTF3C1 PE=1 SV=4
Q5THK1;C9J9	16	8	105.0807	1.888E-03	2.323E-05	1.434947	0.98409	P-0.5PG-mT	F-0.5PG-mT	241291.9	Protein PRR14L OS=Homo sapiens OX=9606 GN=PRR14L PE=1 SV=1
A0A7I2V4I5;Q	12	8	78.1045	3.580E-08	9.282E-10	1.891177	1	P-mT-Susp	F-mT-2D	197391	Zinc finger CCCH domain-containing protein 13 OS=Homo sapiens OX=9606 GN=ZC3H13 PE=1 SV=1
Q8TBY8;F5GX	10	8	48.4183	1.161E-09	4.889E-11	1.740061	1	P-mT-Susp	P-0.5PG-mT	119033.8	Polyamine-modulated factor 1-binding protein 1 OS=Homo sapiens OX=9606 GN=PMFBP1 PE=2 SV=3
Q9GZV9	10	8	59.6855	2.763E-10	1.496E-11	2.053379	1	P-0.5PG-mT	P-mT-Susp	28353.09	Fibroblast growth factor 23 OS=Homo sapiens OX=9606 GN=FGF23 PE=1 SV=1
O14647;A0A1	8	8	51.2974	6.851E-12	8.390E-13	2.482952	1	P-mT-Susp	mT blank	212313.2	Chromodomain-helicase-DNA-binding protein 2 OS=Homo sapiens OX=9606 GN=CHD2 PE=1 SV=2
A0A6E1W127	11	8	67.0913	5.407E-14	3.913E-14	4.992645	1	P-mT-Susp	mT blank	222529.5	Myosin-7B OS=Homo sapiens OX=9606 GN=MYH7B PE=1 SV=1
Q9NWH9;H7B	10	8	53.984	1.945E-02	2.212E-04	1.474332	0.80926	mT blank	F-mT-2D	117433.7	SAFB-like transcription modulator OS=Homo sapiens OX=9606 GN=SLTM PE=1 SV=2
Q562F6	7	7	34.1557	1.366E-06	2.571E-08	1.720525	1	P-mT-Susp	F-0.5PG-mT	145879.9	Shugoshin 2 OS=Homo sapiens OX=9606 GN=SGO2 PE=1 SV=2
Q13136;E9PJZ	13	7	76.8224	4.470E-11	3.554E-12	3.021142	1	P-mT-Susp	mT blank	136349.3	Liprin-alpha-1 OS=Homo sapiens OX=9606 GN=PPFIA1 PE=1 SV=1
P78423	8	7	67.2726	2.934E-12	5.431E-13	5.384873	1	P-mT-Susp	P-0.5PG-mT	42658.95	Fractalkine OS=Homo sapiens OX=9606 GN=CX3CL1 PE=1 SV=1
Q86U86;E7EV	11	7	65.5957	4.768E-05	7.176E-07	2.656155	1	F-0.5PG-mT	P-mT-Susp	194202.8	Protein polybromo-1 OS=Homo sapiens OX=9606 GN=PBRM1 PE=1 SV=1
Q9BQE3	8	7	45.1049	7.726E-05	1.120E-06	1.247145	0.99999	P-0.5PG-mT	mT blank	50579.74	Tubulin alpha-1C chain OS=Homo sapiens OX=9606 GN=TUBA1C PE=1 SV=1
A0A2R8YF72;	8	7	57.0691	4.194E-03	5.006E-05	1.41699	0.95483	F-0.5PG-mT	F-mT-2D	137410	Myelin transcription factor 1-like protein OS=Homo sapiens OX=9606 GN=MYT1L PE=1 SV=1
P48723	8	7	56.5224	3.741E-11	3.168E-12	2.825657	1	P-mT-Susp	mT blank	51984.57	Heat shock 70 kDa protein 13 OS=Homo sapiens OX=9606 GN=HSPA13 PE=1 SV=1
P19174	7	6	32.9172	5.704E-07	1.141E-08	1.68233	1	P-mT-Susp	mT blank	149844	1-phosphatidylinositol 4_5-bisphosphate phosphodiesterase gamma-1 OS=Homo sapiens OX=9606 GN
Q8NHU2;A0A	7	6	39.0619	6.168E-06	1.066E-07	2.187184	1	P-0.5PG-mT	P-mT-Susp	143173.6	Cilia- and flagella-associated protein 61 OS=Homo sapiens OX=9606 GN=CFAP61 PE=2 SV=3
E7ESP2;J3KS3	6	6	37.4242	2.234E-02	2.526E-04	1.441847	0.78925	P-mT-Susp	F-mT-2D	50469.26	Cyclin-dependent kinase 11A OS=Homo sapiens OX=9606 GN=CDK11A PE=1 SV=1
J3QQJ5;Q9Y2L	7	6	61.006	1.519E-08	4.396E-10	2.569997	1	P-0.5PG-mT	F-0.5PG-mT	156467.2	Trafficking protein particle complex subunit 8 OS=Homo sapiens OX=9606 GN=TRAPPC8 PE=1 SV=1
Q14515	7	6	47.0333	4.245E-10	2.048E-11	2.757934	1	F-mT-2D	F-0.5PG-mT	76063.26	SPARC-like protein 1 OS=Homo sapiens OX=9606 GN=SPARCL1 PE=1 SV=2
O95248;H0Y5	8	6	54.3841	5.829E-09	1.925E-10	2.432403	1	P-mT-Susp	mT blank	210553.6	Myotubularin-related protein 5 OS=Homo sapiens OX=9606 GN=SBF1 PE=1 SV=4
Q07890;C9K0	9	6	56.2226	1.486E-09	6.096E-11	2.842994	1	P-0.5PG-mT	mT blank	154348.1	Son of sevenless homolog 2 OS=Homo sapiens OX=9606 GN=SOS2 PE=1 SV=2

O00160	10	6	62.3842	8.481E-06	1.418E-07	1.363425	1	P-0.5PG-mT	P-mT-Susp	125585.8	Unconventional myosin-If OS=Homo sapiens OX=9606 GN=MYO1F PE=1 SV=3
P49756;H0YE4	9	6	59.0699	1.237E-01	1.333E-03	1.117833	0.47249	mT blank	P-mT-Susp	100527.8	RNA-binding protein 25 OS=Homo sapiens OX=9606 GN=RBM25 PE=1 SV=3
Q9UQD0;A0A	6	5	32.5516	3.143E-11	2.858E-12	2.056325	1	P-0.5PG-mT	mT blank	227504.7	Sodium channel protein type 8 subunit alpha OS=Homo sapiens OX=9606 GN=SCN8A PE=1 SV=1
D6W5U7;Q9U	6	5	34.3658	2.516E-05	3.920E-07	1.33424	1	P-mT-Susp	mT blank	140432.5	Cohesin subunit SA-3 OS=Homo sapiens OX=9606 GN=STAG3 PE=1 SV=1
P10827;J3KTF	5	5	25.2558	3.145E-10	1.638E-11	2.970759	1	P-mT-Susp	mT blank	55899.15	Thyroid hormone receptor alpha OS=Homo sapiens OX=9606 GN=THRA PE=1 SV=1
Q9NSY1;H0Y9	5	5	27.7673	1.177E-10	7.439E-12	3.211304	1	F-mT-2D	F-0.5PG-mT	130027.1	BMP-2-inducible protein kinase OS=Homo sapiens OX=9606 GN=BMP2K PE=1 SV=2
A0A0D9SG04;	6	5	40.6413	2.217E-09	8.609E-11	2.0004	1	P-mT-Susp	mT blank	135659.9	Cordon-bleu protein-like 1 OS=Homo sapiens OX=9606 GN=COBLL1 PE=1 SV=2
Q86VW0;C9J4	6	5	28.843	1.371E-10	8.398E-12	2.39835	1	P-mT-Susp	mT blank	80089.78	SEC14 domain and spectrin repeat-containing protein 1 OS=Homo sapiens OX=9606 GN=SESTD1 PE=1
Q9H792;H0YN	5	5	39.1516	1.551E-13	6.498E-14	4.570707	1	P-mT-Susp	mT blank	195159.7	Inactive tyrosine-protein kinase PEAK1 OS=Homo sapiens OX=9606 GN=PEAK1 PE=1 SV=4
Q9HBG6;D6R	6	5	26.3454	1.304E-01	1.397E-03	1.090156	0.46168	F-0.5PG-mT	mT blank	143878.4	Intraflagellar transport protein 122 homolog OS=Homo sapiens OX=9606 GN=IFT122 PE=1 SV=2
Q9H9Y6	5	5	26.4362	4.999E-10	2.341E-11	2.445365	1	P-mT-Susp	mT blank	129883.6	DNA-directed RNA polymerase I subunit RPA2 OS=Homo sapiens OX=9606 GN=POLR1B PE=1 SV=2
Q5TB80	6	5	30.7505	4.986E-09	1.682E-10	2.21011	1	P-mT-Susp	mT blank	162456.5	Centrosomal protein of 162 kDa OS=Homo sapiens OX=9606 GN=CEP162 PE=1 SV=2
P31371	6	5	30.8225	9.408E-05	1.347E-06	2.312509	0.99998	mT blank	P-mT-Susp	23554.61	Fibroblast growth factor 9 OS=Homo sapiens OX=9606 GN=FGF9 PE=1 SV=3
O15067	5	5	33.8933	1.322E-03	1.654E-05	2.4264	0.99078	P-mT-Susp	mT blank	146388.4	Phosphoribosylformylglycinamidine synthase OS=Homo sapiens OX=9606 GN=PFAS PE=1 SV=4
O14795	5	5	28.4423	2.697E-11	2.562E-12	3.665899	1	P-mT-Susp	P-0.5PG-mT	182789	Protein unc-13 homolog B OS=Homo sapiens OX=9606 GN=UNC13B PE=1 SV=2
A0A494C0R8;	5	5	28.4962	9.916E-07	1.912E-08	1.927131	1	P-mT-Susp	mT blank	151892	Clustered mitochondria protein homolog OS=Homo sapiens OX=9606 GN=CLUH PE=1 SV=1
P55198;Q6P2	5	5	32.9597	2.854E-08	7.702E-10	1.988718	1	P-mT-Susp	mT blank	113530.8	Protein AF-17 OS=Homo sapiens OX=9606 GN=MLLT6 PE=1 SV=3
A0A0C4DH07;	6	5	33.2988	4.878E-09	1.652E-10	2.600665	1	P-mT-Susp	mT blank	178352.8	Latent-transforming growth factor beta-binding protein 4 OS=Homo sapiens OX=9606 GN=LTBP4 PE=1
Q92608;E5RFJ	7	5	43.0395	1.867E-04	2.580E-06	1.223702	0.99985	P-mT-Susp	mT blank	213260.4	Dedicator of cytokinesis protein 2 OS=Homo sapiens OX=9606 GN=DOCK2 PE=1 SV=2
P02647	10	5	62.9429	4.017E-08	1.028E-09	2.514365	1	P-mT-Susp	mT blank	30777.87	Apolipoprotein A-I OS=Homo sapiens OX=9606 GN=APOA1 PE=1 SV=1
Q13751	7	5	52.6095	1.167E-09	4.889E-11	2.212769	1	P-mT-Susp	mT blank	133450.6	Laminin subunit beta-3 OS=Homo sapiens OX=9606 GN=LAMB3 PE=1 SV=1
Q12873	7	5	32.4451	3.447E-04	4.612E-06	2.327643	0.9993	P-mT-Susp	F-mT-2D	228131.8	Chromodomain-helicase-DNA-binding protein 3 OS=Homo sapiens OX=9606 GN=CHD3 PE=1 SV=3
Q7Z6G8;H0YJ	8	5	52.4612	1.151E-10	7.329E-12	2.197737	1	F-mT-2D	mT blank	139321	Ankyrin repeat and sterile alpha motif domain-containing protein 1B OS=Homo sapiens OX=9606 GN=
F8WD26;J3KP	7	5	45.4662	2.001E-07	4.341E-09	2.370023	1	P-mT-Susp	F-0.5PG-mT	187376.4	LIM domain only protein 7 OS=Homo sapiens OX=9606 GN=LMO7 PE=1 SV=2
Q9P2R6;A0A5	8	5	50.4691	4.850E-05	7.285E-07	2.029078	1	P-mT-Susp	mT blank	173564.4	Arginine-glutamic acid dipeptide repeats protein OS=Homo sapiens OX=9606 GN=RERE PE=1 SV=2
Q9Y2P4;D6RA	7	5	52.5075	1.487E-08	4.319E-10	1.92185	1	P-mT-Susp	mT blank	70910.09	Long-chain fatty acid transport protein 6 OS=Homo sapiens OX=9606 GN=SLC27A6 PE=1 SV=1
G8JLD3;Q8IUD	4	4	27.2376	1.164E-05	1.911E-07	1.337228	1	F-0.5PG-mT	F-mT-2D	124991.5	ELKS/Rab6-interacting/CAST family member 1 OS=Homo sapiens OX=9606 GN=ERC1 PE=1 SV=1
Q8TE60	4	4	18.3873	2.520E-02	2.838E-04	1.258499	0.77088	P-0.5PG-mT	F-mT-2D	139558.4	A disintegrin and metalloproteinase with thrombospondin motifs 18 OS=Homo sapiens OX=9606 GN=
Q96MT7	5	4	26.1391	4.851E-08	1.211E-09	3.113624	1	P-0.5PG-mT	F-mT-2D	215120.3	Cilia- and flagella-associated protein 44 OS=Homo sapiens OX=9606 GN=CFAP44 PE=1 SV=2
Q49AJ0	5	4	26.0243	2.162E-04	2.978E-06	1.366471	0.99977	P-mT-Susp	P-0.5PG-mT	157138.6	Protein FAM135B OS=Homo sapiens OX=9606 GN=FAM135B PE=1 SV=2
G3V419;Q9UH	5	4	31.0409	4.214E-06	7.488E-08	2.17655	1	P-mT-Susp	F-0.5PG-mT	145826.8	DNA mismatch repair protein Mlh3 OS=Homo sapiens OX=9606 GN=MLH3 PE=1 SV=1
A2RUS2;E9PF3	5	4	20.7992	4.674E-07	9.515E-09	2.028531	1	P-0.5PG-mT	P-mT-Susp	137829.3	DENN domain-containing protein 3 OS=Homo sapiens OX=9606 GN=DENND3 PE=1 SV=2
O00308;H3BP	5	4	25.3138	1.733E-02	1.977E-04	1.287212	0.82503	P-mT-Susp	F-mT-2D	99482.48	NEDD4-like E3 ubiquitin-protein ligase WWP2 OS=Homo sapiens OX=9606 GN=WWP2 PE=1 SV=2
A0A0C4DFQ3;	5	4	23.3195	4.713E-08	1.187E-09	2.123548	1	P-0.5PG-mT	mT blank	71227.38	Zinc finger and SCAN domain-containing protein 2 OS=Homo sapiens OX=9606 GN=ZSCAN2 PE=1 SV=1
Q02410	4	4	30.018	5.495E-11	4.127E-12	8.270616	1	P-mT-Susp	P-0.5PG-mT	93321.34	Amyloid-beta A4 precursor protein-binding family A member 1 OS=Homo sapiens OX=9606 GN=APBA
G3V200;O753	4	4	23.1875	2.702E-08	7.366E-10	3.732787	1	P-mT-Susp	mT blank	142564.7	Liprin-alpha-2 OS=Homo sapiens OX=9606 GN=PPFIA2 PE=1 SV=2
A0A2R8Y855;	5	4	29.4	3.659E-11	3.132E-12	8.738727	1	P-mT-Susp	P-0.5PG-mT	41988.16	SWI/SNF-related matrix-associated actin-dependent regulator of chromatin subfamily E member 1 OS
Q58EX7;H3BM	5	4	36.597	1.737E-05	2.776E-07	1.574066	1	P-0.5PG-mT	P-mT-Susp	132513.6	Puratrophin-1 OS=Homo sapiens OX=9606 GN=PLEKHG4 PE=1 SV=1
Q92620	4	4	22.9054	2.111E-03	2.589E-05	3.757159	0.98133	mT blank	P-mT-Susp	141358.4	Pre-mRNA-splicing factor ATP-dependent RNA helicase PRP16 OS=Homo sapiens OX=9606 GN=DHX38
O15523;C9J8	4	4	20.729	5.410E-11	4.102E-12	4.377966	1	P-mT-Susp	P-0.5PG-mT	73609.96	ATP-dependent RNA helicase DDX3Y OS=Homo sapiens OX=9606 GN=DDX3Y PE=1 SV=2
B7ZAX5;Q014	4	4	26.0577	8.040E-06	1.356E-07	1.758448	1	P-mT-Susp	mT blank	48547.6	N-acetylgalactosamine kinase OS=Homo sapiens OX=9606 GN=GALK2 PE=1 SV=1
A0A0G2JPU2;	4	4	24.4319	1.641E-09	6.543E-11	2.119935	1	P-mT-Susp	P-0.5PG-mT	99612.2	Alpha-helical coiled-coil rod protein OS=Homo sapiens OX=9606 GN=CCHCR1 PE=1 SV=1
O60765	4	4	29.5068	2.329E-11	2.289E-12	2.541249	1	P-mT-Susp	mT blank	71061.74	Zinc finger protein 354A OS=Homo sapiens OX=9606 GN=ZNF354A PE=1 SV=2
C9J066;E9PN6	5	4	33.018	1.787E-07	3.929E-09	2.047777	1	P-mT-Susp	F-mT-2D	240189.4	Ninein OS=Homo sapiens OX=9606 GN=NIN PE=1 SV=1
Q92613	4	4	29.2702	2.746E-09	1.031E-10	2.160855	1	P-mT-Susp	mT blank	95462.1	Protein Jade-3 OS=Homo sapiens OX=9606 GN=JADE3 PE=1 SV=1
A8MQ14;A0A	4	4	35.0575	6.031E-05	8.973E-07	1.46618	1	F-0.5PG-mT	F-mT-2D	129651.2	Zinc finger protein 850 OS=Homo sapiens OX=9606 GN=ZNF850 PE=3 SV=2
A0A0A0MS30	4	4	23.2785	4.235E-11	3.448E-12	2.936724	1	P-mT-Susp	P-0.5PG-mT	34654.11	Protein PRRC2C OS=Homo sapiens OX=9606 GN=PRRC2C PE=1 SV=1
Q96KG7;A0A5	4	4	29.4899	2.153E-07	4.645E-09	1.650594	1	F-mT-2D	mT blank	130645.5	Multiple epidermal growth factor-like domains protein 10 OS=Homo sapiens OX=9606 GN=MEGF10 PE
A0A087WTJ9;	4	4	32.0976	8.895E-09	2.788E-10	1.815108	1	P-mT-Susp	F-mT-2D	125780.9	E3 ubiquitin-protein ligase OS=Homo sapiens OX=9606 GN=UBR1 PE=1 SV=1
Q9Y5Q9;A0A4	4	4	36.003	1.470E-06	2.753E-08	2.170982	1	P-mT-Susp	mT blank	102070.8	General transcription factor 3C polypeptide 3 OS=Homo sapiens OX=9606 GN=GTF3C3 PE=1 SV=1

A0A2R8YFV2;	4	4	27.3037	5.246E-03	6.224E-05	1.651034	0.94183	P-0.5PG-mT	mT blank	185512.9	Protein Wiz OS=Homo sapiens OX=9606 GN=WIZ PE=1 SV=1
B7WNT5;P364	4	4	33.126	5.778E-02	6.379E-04	2.103588	0.62539	P-0.5PG-mT	mT blank	52447.75	Transcription factor 7 OS=Homo sapiens OX=9606 GN=TCF7 PE=1 SV=1
F5H619;Q86X	4	4	18.9836	2.649E-04	3.598E-06	1.333549	0.99962	P-0.5PG-mT	F-mT-2D	226068.7	HEAT repeat-containing protein 5A OS=Homo sapiens OX=9606 GN=HEATR5A PE=1 SV=1
O75717;H3BS	4	4	26.6567	5.888E-11	4.340E-12	2.603615	1	P-mT-Susp	mT blank	127450.2	WD repeat and HMG-box DNA-binding protein 1 OS=Homo sapiens OX=9606 GN=WDHD1 PE=1 SV=1
Q14678;Q5W	5	4	23.9872	5.639E-08	1.373E-09	2.097207	1	P-0.5PG-mT	mT blank	149114.3	KN motif and ankyrin repeat domain-containing protein 1 OS=Homo sapiens OX=9606 GN=KANK1 PE=
F5H538;J3KN	5	4	34.2814	1.930E-12	4.151E-13	3.13144	1	P-mT-Susp	mT blank	182949.4	Mitogen-activated protein kinase kinase kinase 4 OS=Homo sapiens OX=9606 GN=MAP3K4 PE=1 SV=1
Q6UXG2;H0Y	6	4	37.7519	2.443E-12	4.862E-13	2.805056	1	P-mT-Susp	mT blank	114518.2	Endosome/lysosome-associated apoptosis and autophagy regulator 1 OS=Homo sapiens OX=9606 GN
Q9BY89	4	4	19.6354	2.507E-06	4.557E-08	1.82226	1	P-mT-Susp	F-mT-2D	197737.6	Uncharacterized protein KIAA1671 OS=Homo sapiens OX=9606 GN=KIAA1671 PE=1 SV=2
P06239	7	4	49.7097	2.487E-09	9.472E-11	3.000732	1	P-0.5PG-mT	F-mT-2D	58513.95	Tyrosine-protein kinase Lck OS=Homo sapiens OX=9606 GN=LCK PE=1 SV=6
E9PHY5;O434	7	4	35.2358	4.376E-08	1.106E-09	3.122011	1	P-0.5PG-mT	P-mT-Susp	104873.2	Band 4.1-like protein 2 OS=Homo sapiens OX=9606 GN=EPB41L2 PE=1 SV=1
P05787	5	4	48.3781	2.736E-05	4.221E-07	1.846893	1	F-0.5PG-mT	P-mT-Susp	53704.36	Keratin_ type II cytoskeletal 8 OS=Homo sapiens OX=9606 GN=KRT8 PE=1 SV=7
A0A3B3ISG5;	5	4	28.4836	1.038E-09	4.465E-11	3.229183	1	P-mT-Susp	mT blank	118741.2	Insulin-degrading enzyme OS=Homo sapiens OX=9606 GN=IDE PE=1 SV=1
Q6B0I6;K7ES2	5	4	22.7227	1.172E-08	3.533E-10	2.786705	1	P-mT-Susp	mT blank	59230.15	Lysine-specific demethylase 4D OS=Homo sapiens OX=9606 GN=KDM4D PE=1 SV=3
Q13009	5	4	27.3478	2.922E-08	7.857E-10	1.465494	1	P-mT-Susp	F-mT-2D	178820	Rho guanine nucleotide exchange factor TIAM1 OS=Homo sapiens OX=9606 GN=TIAM1 PE=1 SV=2
G3V0I5;P4982	5	4	35.2835	6.291E-12	7.858E-13	2.85053	1	P-mT-Susp	mT blank	50738.67	NADH dehydrogenase [ubiquinone] flavoprotein 1_ mitochondrial OS=Homo sapiens OX=9606 GN=ND
G3XAE9;Q9Y4	8	4	47.2194	3.057E-02	3.418E-04	1.347047	0.73993	P-0.5PG-mT	P-mT-Susp	196689.1	KIAA0423_ isoform CRA_a OS=Homo sapiens OX=9606 GN=TOGARAM1 PE=1 SV=1
A0A286YEW9;	6	4	41.093	2.286E-09	8.790E-11	2.297286	1	P-mT-Susp	F-mT-2D	63770.86	Mitogen-activated protein kinase OS=Homo sapiens OX=9606 GN=MAPK10 PE=1 SV=1
E2QRF0;Q70E	5	4	30.4862	6.821E-04	8.815E-06	1.48041	0.99711	P-0.5PG-mT	mT blank	87540.69	Ubiquitin carboxyl-terminal hydrolase OS=Homo sapiens OX=9606 GN=USP45 PE=1 SV=1
A6NNK5;Q128	5	4	31.1197	1.891E-12	4.151E-13	3.398461	1	P-mT-Susp	P-0.5PG-mT	211015.6	TP53-binding protein 1 OS=Homo sapiens OX=9606 GN=TP53BP1 PE=1 SV=2
A0A0U1RQK4	5	4	23.6334	6.477E-07	1.283E-08	2.077454	1	P-mT-Susp	mT blank	213471.2	[Protein ADP-ribosylarginine] hydrolase-like protein 1 OS=Homo sapiens OX=9606 GN=ADPRHL1 PE=1
H0YJB5;Q6GY	8	4	58.5444	3.041E-08	8.151E-10	2.065534	1	P-mT-Susp	F-0.5PG-mT	241791.7	Ral GTPase-activating protein subunit alpha-1 OS=Homo sapiens OX=9606 GN=RALGAPA1 PE=1 SV=2
H0Y6E7;P381	7	4	30.4336	8.980E-06	1.492E-07	1.348031	1	F-0.5PG-mT	F-mT-2D	31745.77	RNA-binding motif protein_ X chromosome (Fragment) OS=Homo sapiens OX=9606 GN=RBMX PE=1 S
P13497;B7ZK	3	3	16.6957	6.506E-05	9.645E-07	2.331128	0.99999	mT blank	F-mT-2D	113586.9	Bone morphogenetic protein 1 OS=Homo sapiens OX=9606 GN=BMP1 PE=1 SV=2
E9PKG2	4	3	19.272	1.268E-08	3.779E-10	3.623045	1	P-0.5PG-mT	P-mT-Susp	67707.93	Low-density lipoprotein receptor-related protein 8 OS=Homo sapiens OX=9606 GN=LRP8 PE=1 SV=2
A0A1W2PR94	3	3	16.9118	8.409E-02	9.157E-04	1.670498	0.55134	P-mT-Susp	F-0.5PG-mT	103457.7	Death domain-containing protein 1 OS=Homo sapiens OX=9606 GN=DTHD1 PE=1 SV=1
A0A1B0GW10	4	3	22.7012	9.635E-04	1.229E-05	2.142609	0.99457	P-mT-Susp	F-mT-2D	162898	Methyl-CpG-binding domain protein 5 OS=Homo sapiens OX=9606 GN=MBD5 PE=4 SV=2
Q9HC52;C9J6	4	3	25.2879	2.594E-05	4.025E-07	1.467485	1	F-mT-2D	mT blank	43509.83	Chromobox protein homolog 8 OS=Homo sapiens OX=9606 GN=CBX8 PE=1 SV=3
O14640;A0A0	3	3	24.7716	2.758E-11	2.583E-12	7.299549	1	F-0.5PG-mT	P-0.5PG-mT	75586.07	Segment polarity protein dishevelled homolog DVL-1 OS=Homo sapiens OX=9606 GN=DVL1 PE=1 SV=2
A0A7P0T8Y0;	3	3	6.967	8.983E-11	6.062E-12	6.492913	1	P-mT-Susp	mT blank	196337.4	Probable ATP-dependent RNA helicase DDX60 OS=Homo sapiens OX=9606 GN=DDX60 PE=4 SV=1
A0A494C1F2;	4	3	17.3408	2.053E-06	3.774E-08	2.367838	1	P-mT-Susp	mT blank	213226	Nuclear pore complex protein Nup214 OS=Homo sapiens OX=9606 GN=NUP214 PE=1 SV=1
Q9Y4F5;J3KQ	3	3	9.4007	6.560E-05	9.688E-07	1.680852	0.99999	P-mT-Susp	mT blank	172258.5	Centrosomal protein of 170 kDa protein B OS=Homo sapiens OX=9606 GN=CEP170B PE=1 SV=4
Q9BZF1;F8VQ	4	3	28.5559	2.647E-01	2.814E-03	1.652167	0.31749	mT blank	F-mT-2D	101823	Oxysterol-binding protein-related protein 8 OS=Homo sapiens OX=9606 GN=OSBPL8 PE=1 SV=3
P49765	4	3	27.7866	3.358E-09	1.221E-10	3.8403	1	P-0.5PG-mT	P-mT-Susp	22058.03	Vascular endothelial growth factor B OS=Homo sapiens OX=9606 GN=VEGFB PE=1 SV=2
Q7Z6I6;A0A0	4	3	20.9513	9.126E-08	2.162E-09	1.682656	1	F-mT-2D	mT blank	119722.8	Rho GTPase-activating protein 30 OS=Homo sapiens OX=9606 GN=ARHGAP30 PE=1 SV=3
Q8TDR0	4	3	19.9836	1.738E-06	3.225E-08	2.233626	1	P-mT-Susp	mT blank	78974.04	TRAF3-interacting protein 1 OS=Homo sapiens OX=9606 GN=TRAF3IP1 PE=1 SV=1
A0A3B3IRJ9;	3	3	15.8157	3.821E-10	1.877E-11	2.65718	1	F-0.5PG-mT	P-mT-Susp	122106.2	Phosphatidylinositide phosphatase SAC2 OS=Homo sapiens OX=9606 GN=INPP5F PE=1 SV=1
Q5JWF2;A0A0	3	3	16.1634	7.526E-02	8.218E-04	1.218922	0.57358	P-mT-Susp	F-0.5PG-mT	111766	Guanine nucleotide-binding protein G(s) subunit alpha isoforms XLas OS=Homo sapiens OX=9606 GN=
D6RJB7;Q8N7	3	3	16.1182	9.694E-09	2.991E-10	2.494468	1	P-mT-Susp	P-0.5PG-mT	219731.4	Ankyrin repeat domain-containing protein 31 OS=Homo sapiens OX=9606 GN=ANKRD31 PE=4 SV=2
Q6NUP7	3	3	14.9981	4.812E-01	5.080E-03	1.453069	0.20279	P-0.5PG-mT	F-mT-2D	100535.7	Serine/threonine-protein phosphatase 4 regulatory subunit 4 OS=Homo sapiens OX=9606 GN=PPP4R4
P56199	3	3	15.0191	1.231E-02	1.419E-04	1.966558	0.86718	P-mT-Susp	mT blank	132387.7	Integrin alpha-1 OS=Homo sapiens OX=9606 GN=ITGA1 PE=1 SV=2
A0A087X2D8;	4	3	32.607	2.993E-07	6.302E-09	2.131124	1	P-mT-Susp	F-mT-2D	145967.9	C-Jun-amino-terminal kinase-interacting protein 4 OS=Homo sapiens OX=9606 GN=SPAG9 PE=1 SV=1
Q8TAQ5;K7EL	3	3	13.9763	1.619E-03	2.007E-05	4.251231	0.98735	mT blank	P-mT-Susp	82756.16	Zinc finger protein 420 OS=Homo sapiens OX=9606 GN=ZNF420 PE=1 SV=1
Q8WXA9	3	3	14.3705	3.839E-08	9.890E-10	3.617452	1	P-mT-Susp	P-0.5PG-mT	59437.39	Splicing regulatory glutamine/lysine-rich protein 1 OS=Homo sapiens OX=9606 GN=SREK1 PE=1 SV=1
Q6SA08;H7C3	3	3	29.268	3.057E-09	1.127E-10	2.117769	1	P-mT-Susp	mT blank	37853.61	Testis-specific serine/threonine-protein kinase 4 OS=Homo sapiens OX=9606 GN=TSSK4 PE=1 SV=1
A8MQ02	3	3	16.4656	7.163E-06	1.216E-07	2.08845	1	F-0.5PG-mT	F-mT-2D	202832.5	Afadin OS=Homo sapiens OX=9606 GN=AFDN PE=1 SV=2
A0A3B3IRX3;	3	3	15.6789	5.286E-09	1.768E-10	12.63155	1	P-0.5PG-mT	P-mT-Susp	246927.1	Mediator of RNA polymerase II transcription subunit 13 OS=Homo sapiens OX=9606 GN=MED13L PE=
A0A286YFC2;	3	3	14.582	1.057E-01	1.143E-03	1.395836	0.5048	P-0.5PG-mT	F-0.5PG-mT	88036.76	Tubulin polyglutamylase TTLL13P OS=Homo sapiens OX=9606 GN=TTLL13P PE=4 SV=1
P45844;E9PG	3	3	21.346	2.247E-07	4.821E-09	2.408303	1	P-mT-Susp	mT blank	76561.7	ATP-binding cassette sub-family G member 1 OS=Homo sapiens OX=9606 GN=ABCG1 PE=1 SV=3
M0R0P8;M0R	3	3	21.7684	1.717E-07	3.786E-09	3.932092	1	P-0.5PG-mT	mT blank	244997	Unconventional myosin-IXb OS=Homo sapiens OX=9606 GN=MYO9B PE=1 SV=1
A0A087X0R2;	3	3	21.7779	2.315E-05	3.628E-07	1.389674	1	F-0.5PG-mT	mT blank	15532.1	Mediator of DNA damage checkpoint protein 1 OS=Homo sapiens OX=9606 GN=MDC1 PE=1 SV=1

Q9Y5I2;A0A5F	3	3	16.6045	5.516E-08	1.347E-09	1.850957	1	F-mT-2D	mT blank	103673.7	Protocadherin alpha-10 OS=Homo sapiens OX=9606 GN=PCDHA10 PE=2 SV=1
Q9HCE5;A0A0	3	3	20.4985	1.809E-05	2.880E-07	1.641066	1	P-mT-Susp	F-mT-2D	52720.79	N6-adenosine-methyltransferase non-catalytic subunit OS=Homo sapiens OX=9606 GN=METTL14 PE=1
F5GXT3;Q9NC	3	3	20.5649	1.607E-04	2.245E-06	2.743197	0.9999	P-mT-Susp	mT blank	114684.9	Anoctamin OS=Homo sapiens OX=9606 GN=ANO2 PE=1 SV=2
C9J4K5;C9JSP0	3	3	21.2306	9.051E-11	6.062E-12	5.813746	1	P-mT-Susp	mT blank	55210.98	Protein downstream neighbor of Son OS=Homo sapiens OX=9606 GN=DONSON PE=1 SV=1
F8WA39;Q136	3	3	17.8428	3.030E-05	4.639E-07	2.364059	1	mT blank	F-mT-2D	76459.46	Phosphatidylinositol-3_5-bisphosphate 3-phosphatase OS=Homo sapiens OX=9606 GN=MTMR1 PE=1 S
Q9Y239;G3XA	3	3	13.7579	8.870E-06	1.477E-07	4.670439	1	P-0.5PG-mT	P-mT-Susp	109288.1	Nucleotide-binding oligomerization domain-containing protein 1 OS=Homo sapiens OX=9606 GN=NOD
B1AM31;Q5T1	3	3	26.5784	1.124E-10	7.282E-12	2.710276	1	P-mT-Susp	mT blank	102348.9	Axonemal dynein light chain domain-containing protein 1 (Fragment) OS=Homo sapiens OX=9606 GN=
Q96R06	3	3	16.2308	5.127E-10	2.387E-11	3.209396	1	P-mT-Susp	mT blank	135790.7	Sperm-associated antigen 5 OS=Homo sapiens OX=9606 GN=SPAG5 PE=1 SV=2
Q8IX18;J3KTK	3	3	23.6222	1.456E-05	2.361E-07	1.857836	1	P-0.5PG-mT	F-mT-2D	89871.86	Probable ATP-dependent RNA helicase DHX40 OS=Homo sapiens OX=9606 GN=DHX40 PE=1 SV=2
E2QRD4;Q6ZR	3	3	21.2892	5.379E-09	1.791E-10	2.854248	1	P-mT-Susp	mT blank	139214.9	Methyl methanesulfonate-sensitivity protein 22-like OS=Homo sapiens OX=9606 GN=MMS22L PE=1 SV
Q6IV72	3	3	18.7251	7.609E-11	5.507E-12	2.068856	1	P-mT-Susp	mT blank	90686.35	Zinc finger protein 425 OS=Homo sapiens OX=9606 GN=ZNF425 PE=1 SV=1
Q53TQ3	3	3	27.4095	1.158E-06	2.205E-08	2.520024	1	F-mT-2D	P-mT-Susp	114399.8	INO80 complex subunit D OS=Homo sapiens OX=9606 GN=INO80D PE=1 SV=3
P49321	3	3	14.3682	5.513E-05	8.234E-07	2.445764	1	mT blank	P-mT-Susp	85522.96	Nuclear autoantigenic sperm protein OS=Homo sapiens OX=9606 GN=NASP PE=1 SV=2
Q96JB5;J3QQ	3	3	15.9285	1.168E-11	1.328E-12	2.715016	1	F-mT-2D	F-0.5PG-mT	57262.93	CDK5 regulatory subunit-associated protein 3 OS=Homo sapiens OX=9606 GN=CDK5RAP3 PE=1 SV=2
Q6ZMS4;A0A3	3	3	22.7062	2.465E-10	1.382E-11	7.846731	1	P-mT-Susp	P-0.5PG-mT	63680.76	Zinc finger protein 852 OS=Homo sapiens OX=9606 GN=ZNF852 PE=1 SV=4
G3V1R7;O149	4	3	28.809	1.883E-12	4.151E-13	17.18815	1	P-mT-Susp	mT blank	44343.85	Solute carrier family 27 (Fatty acid transporter)_ member 2_ isoform CRA_b OS=Homo sapiens OX=96
Q05516	3	3	10.951	8.745E-09	2.753E-10	2.11252	1	F-mT-2D	P-0.5PG-mT	75699.9	Zinc finger and BTB domain-containing protein 16 OS=Homo sapiens OX=9606 GN=ZBTB16 PE=1 SV=2
Q96RR1	3	3	18.7463	4.323E-04	5.726E-06	2.326532	0.99885	P-0.5PG-mT	F-mT-2D	77667.54	Twinkle protein_ mitochondrial OS=Homo sapiens OX=9606 GN=TWNK PE=1 SV=1
P21128	3	3	16.9917	1.078E-08	3.301E-10	2.044928	1	P-mT-Susp	mT blank	48126.94	Poly(U)-specific endoribonuclease OS=Homo sapiens OX=9606 GN=ENDOU PE=1 SV=2
H0Y3Q0;P291	3	3	19.678	3.346E-09	1.221E-10	3.096003	1	P-mT-Susp	P-0.5PG-mT	91635.2	Proprotein convertase subtilisin/kexin type 6 (Fragment) OS=Homo sapiens OX=9606 GN=PCSK6 PE=1
P11215	3	3	26.3586	3.080E-11	2.851E-12	2.459858	1	F-mT-2D	F-0.5PG-mT	128490.3	Integrin alpha-M OS=Homo sapiens OX=9606 GN=ITGAM PE=1 SV=2
A0A087X241;	3	3	20.2483	5.593E-03	6.626E-05	1.384823	0.93764	P-mT-Susp	F-mT-2D	123998.7	Small G protein-signaling modulator 1 OS=Homo sapiens OX=9606 GN=SGSM1 PE=1 SV=1
Q495T6	3	3	16.6049	2.793E-13	1.011E-13	11.45908	1	P-mT-Susp	P-0.5PG-mT	90051.34	Membrane metallo-endopeptidase-like 1 OS=Homo sapiens OX=9606 GN=MMEL1 PE=2 SV=2
Q86V71	4	3	21.1231	2.600E-04	3.538E-06	3.687063	0.99964	mT blank	P-mT-Susp	80547.58	Zinc finger protein 429 OS=Homo sapiens OX=9606 GN=ZNF429 PE=2 SV=2
G3V5P6;O954	3	3	17.2359	4.321E-09	1.476E-10	5.937691	1	P-mT-Susp	mT blank	103186.2	Papilin OS=Homo sapiens OX=9606 GN=PAPLN PE=1 SV=1
O15360;F5H8	4	3	26.8943	1.436E-02	1.647E-04	4.34875	0.84911	mT blank	P-mT-Susp	164942.5	Fanconi anemia group A protein OS=Homo sapiens OX=9606 GN=FANCA PE=1 SV=2
A0A494C1R4;	4	3	27.535	1.304E-08	3.874E-10	4.480283	1	P-0.5PG-mT	F-mT-2D	167972.3	Protein-tyrosine-phosphatase OS=Homo sapiens OX=9606 GN=PTPRZ1 PE=1 SV=1
Q8IW36;F6W2	5	3	19.7869	2.618E-07	5.542E-09	2.350717	1	P-mT-Susp	mT blank	62373.27	Zinc finger protein 695 OS=Homo sapiens OX=9606 GN=ZNF695 PE=1 SV=4
E7EVA0;P2781	5	3	31.9842	1.027E-01	1.114E-03	1.571443	0.51068	mT blank	P-mT-Susp	246699.4	Microtubule-associated protein OS=Homo sapiens OX=9606 GN=MAP4 PE=1 SV=1
Q7RTP6;J3QL	4	3	37.4912	2.350E-05	3.676E-07	1.648014	1	P-mT-Susp	F-mT-2D	225435.7	[F-actin]-monooxygenase MICAL3 OS=Homo sapiens OX=9606 GN=MICAL3 PE=1 SV=2
A0A0C4DG89;	4	3	20.6554	7.585E-05	1.106E-06	1.758266	0.99999	P-mT-Susp	P-0.5PG-mT	117974.7	RNA helicase OS=Homo sapiens OX=9606 GN=DDX46 PE=1 SV=1
Q6UB98;F5GY	5	3	27.8818	2.767E-04	3.752E-06	2.241379	0.99958	F-0.5PG-mT	F-mT-2D	237191.6	Ankyrin repeat domain-containing protein 12 OS=Homo sapiens OX=9606 GN=ANKRD12 PE=1 SV=3
C9JFF0;Q9ULI	4	3	26.6282	2.531E-12	4.913E-13	2.59438	1	F-mT-2D	mT blank	182318.3	Kinesin-like protein KIF26A OS=Homo sapiens OX=9606 GN=KIF26A PE=1 SV=1
Q9H3R0	4	3	29.8841	1.380E-01	1.476E-03	1.24134	0.45008	P-mT-Susp	F-mT-2D	122035	Lysine-specific demethylase 4C OS=Homo sapiens OX=9606 GN=KDM4C PE=1 SV=2
P16415;C9J2N	4	3	32.166	9.656E-10	4.178E-11	4.040692	1	P-mT-Susp	P-0.5PG-mT	72836.72	Zinc finger protein 823 OS=Homo sapiens OX=9606 GN=ZNF823 PE=2 SV=2
A0A0B4J1T8;	4	3	23.3963	6.996E-05	1.028E-06	3.41636	0.99999	F-mT-2D	F-0.5PG-mT	128329.7	Receptor protein-tyrosine kinase OS=Homo sapiens OX=9606 GN=EPHA6 PE=1 SV=1
Q8NDV7	6	3	33.8208	4.518E-02	5.016E-04	4.200845	0.67163	mT blank	F-mT-2D	211095.9	Trinucleotide repeat-containing gene 6A protein OS=Homo sapiens OX=9606 GN=TNRC6A PE=1 SV=2
Q9HCE3;K7EJ	4	3	27.4336	1.591E-05	2.553E-07	2.17503	1	P-0.5PG-mT	F-mT-2D	144091.4	Zinc finger protein 532 OS=Homo sapiens OX=9606 GN=ZNF532 PE=1 SV=2
O96028	5	3	25.6458	6.109E-12	7.858E-13	5.648246	1	P-mT-Susp	mT blank	156307.4	Histone-lysine N-methyltransferase NSD2 OS=Homo sapiens OX=9606 GN=NSD2 PE=1 SV=1
A0A0A0MRA8	6	3	31.3948	6.724E-10	3.041E-11	3.15808	1	P-mT-Susp	mT blank	102744.3	Band 4.1-like protein 3 OS=Homo sapiens OX=9606 GN=EPB41L3 PE=1 SV=1
O75362;A2A3	5	3	46.9965	2.895E-03	3.507E-05	1.805762	0.97138	F-0.5PG-mT	P-mT-Susp	117154.1	Zinc finger protein 217 OS=Homo sapiens OX=9606 GN=ZNF217 PE=1 SV=1
A0A0C4DG98;	4	3	15.2044	5.258E-10	2.433E-11	2.172756	1	P-mT-Susp	mT blank	171235.7	THO complex subunit 2 OS=Homo sapiens OX=9606 GN=THOC2 PE=1 SV=1
O95782;A0A0	4	3	29.4295	8.206E-10	3.650E-11	6.374033	1	mT blank	F-0.5PG-mT	108629.6	AP-2 complex subunit alpha-1 OS=Homo sapiens OX=9606 GN=AP2A1 PE=1 SV=3
A0A2R8Y425	13	3	81.6517	1.046E-03	1.324E-05	2.062402	0.99374	F-0.5PG-mT	P-mT-Susp	216922.8	DNA helicase (Fragment) OS=Homo sapiens OX=9606 GN=CHD4 PE=1 SV=1
Q9H4G0;A0A0	8	3	52.277	1.111E-01	1.200E-03	1.203818	0.49466	P-0.5PG-mT	F-mT-2D	99073.28	Band 4.1-like protein 1 OS=Homo sapiens OX=9606 GN=EPB41L1 PE=1 SV=2
Q10570	5	3	31.5577	1.046E-03	1.324E-05	1.699815	0.99374	P-0.5PG-mT	F-mT-2D	162138.7	Cleavage and polyadenylation specificity factor subunit 1 OS=Homo sapiens OX=9606 GN=CPSF1 PE=1
P23381;G3V2	3	3	16.967	6.745E-08	1.627E-09	2.349677	1	P-0.5PG-mT	F-0.5PG-mT	53507.68	Tryptophan--tRNA ligase_ cytoplasmic OS=Homo sapiens OX=9606 GN=WARS1 PE=1 SV=2
Q9UGU0	4	3	18.374	1.272E-10	7.909E-12	2.517189	1	P-mT-Susp	mT blank	213254.1	Transcription factor 20 OS=Homo sapiens OX=9606 GN=TCF20 PE=1 SV=3
H0YJG4	4	3	21.3014	2.804E-09	1.043E-10	2.308594	1	P-mT-Susp	mT blank	209392.2	Chromodomain-helicase-DNA-binding protein 8 (Fragment) OS=Homo sapiens OX=9606 GN=CHD8 PE=
A0A7I2YBM0;	6	3	38.3124	4.805E-06	8.407E-08	2.373995	1	P-0.5PG-mT	P-mT-Susp	208006.9	Endoribonuclease Dicer OS=Homo sapiens OX=9606 GN=DICER1 PE=1 SV=1

P18615;A0A0	6	3	49.5791	5.161E-01	5.441E-03	1.332398	0.18989	mT blank	P-mT-Susp	43353.91	Negative elongation factor E OS=Homo sapiens OX=9606 GN=NELFE PE=1 SV=3
P35354;Q6ZY	4	3	25.9846	3.268E-11	2.923E-12	9.972472	1	P-0.5PG-mT	F-0.5PG-mT	69737.6	Prostaglandin G/H synthase 2 OS=Homo sapiens OX=9606 GN=PTGS2 PE=1 SV=2
Q9BZ29;A0A0	4	3	22.8118	5.713E-13	1.819E-13	5.540048	1	P-mT-Susp	P-0.5PG-mT	238670.1	Dedicator of cytokinesis protein 9 OS=Homo sapiens OX=9606 GN=DOCK9 PE=1 SV=2
O15015;C9J3L	6	3	31.8276	1.879E-10	1.084E-11	2.448216	1	P-mT-Susp	mT blank	205444.5	Zinc finger protein 646 OS=Homo sapiens OX=9606 GN=ZNF646 PE=1 SV=2
J3KNI1;Q9H9E	5	3	25.0937	1.310E-06	2.477E-08	3.32029	1	P-0.5PG-mT	F-mT-2D	90470.22	Component of oligomeric Golgi complex 4 OS=Homo sapiens OX=9606 GN=COG4 PE=1 SV=1
J3QR65;Q9NY	2	2	16.0168	1.509E-07	3.374E-09	2.00115	1	mT blank	P-0.5PG-mT	129522.2	Protein-tyrosine-phosphatase OS=Homo sapiens OX=9606 GN=MTMR4 PE=1 SV=1
Q3SY46	2	2	20.8668	7.350E-14	4.501E-14	7.874845	1	P-mT-Susp	P-0.5PG-mT	20490.16	Keratin-associated protein 13-3 OS=Homo sapiens OX=9606 GN=KRTAP13-3 PE=1 SV=1
F8VZC3	2	2	10.9928	8.543E-03	1.003E-04	1.249747	0.90435	F-0.5PG-mT	mT blank	3761.25	Calcyphosin-2 (Fragment) OS=Homo sapiens OX=9606 GN=CAPS2 PE=1 SV=8
Q03181	2	2	11.5512	3.991E-12	6.484E-13	2.544149	1	P-mT-Susp	mT blank	50701.91	Peroxisome proliferator-activated receptor delta OS=Homo sapiens OX=9606 GN=PPARD PE=1 SV=1
H0Y650;Q86Y	2	2	13.4827	3.313E-03	3.983E-05	2.749793	0.96602	F-0.5PG-mT	F-mT-2D	73442.21	Dynein assembly factor 5_ axonemal (Fragment) OS=Homo sapiens OX=9606 GN=DNAAF5 PE=1 SV=1
A0A7I2V554;C	2	2	16.4961	1.528E-07	3.408E-09	2.992609	1	F-mT-2D	P-mT-Susp	94084.51	UV-stimulated scaffold protein A OS=Homo sapiens OX=9606 GN=UVSSA PE=1 SV=1
H0YL38;H0YL	2	2	16.1825	8.788E-03	1.030E-04	2.001887	0.90176	F-0.5PG-mT	P-mT-Susp	85753.8	Zinc finger protein 280D OS=Homo sapiens OX=9606 GN=ZNF280D PE=1 SV=1
M0QZ12;Q96	2	2	10.9372	9.006E-04	1.151E-05	1.956307	0.99518	mT blank	F-mT-2D	90419.63	Protein Aster-A OS=Homo sapiens OX=9606 GN=GRAMD1A PE=1 SV=1
A0A0A0MSI8;	2	2	15.698	1.393E-06	2.616E-08	2.02558	1	mT blank	P-mT-Susp	83145.8	Exocyst complex component 5 OS=Homo sapiens OX=9606 GN=EXOC5 PE=1 SV=1
P52736	2	2	9.5516	2.866E-01	3.042E-03	1.383824	0.30176	F-mT-2D	mT blank	75612.44	Zinc finger protein 133 OS=Homo sapiens OX=9606 GN=ZNF133 PE=1 SV=2
Q96P53;A0A0	2	2	17.2107	6.529E-05	9.661E-07	1.786584	0.99999	P-mT-Susp	P-0.5PG-mT	45952.8	WD repeat and FYVE domain-containing protein 2 OS=Homo sapiens OX=9606 GN=WDFY2 PE=1 SV=2
Q8WU20	2	2	10.8024	4.258E-06	7.532E-08	1.815706	1	F-mT-2D	mT blank	57428.33	Fibroblast growth factor receptor substrate 2 OS=Homo sapiens OX=9606 GN=FRS2 PE=1 SV=4
A0A669KAX4;	2	2	15.8092	7.109E-05	1.042E-06	3.87386	0.99999	mT blank	P-mT-Susp	103096.9	Cullin-4B OS=Homo sapiens OX=9606 GN=CUL4B PE=1 SV=1
A0A494BZY0;	2	2	16.7311	2.145E-08	6.014E-10	1.730701	1	P-mT-Susp	mT blank	63979.47	Caspase recruitment domain-containing protein 14 OS=Homo sapiens OX=9606 GN=CARD14 PE=1 SV=
Q14585;E9PL	2	2	15.9397	2.469E-09	9.451E-11	2.754257	1	P-mT-Susp	mT blank	57265.22	Zinc finger protein 345 OS=Homo sapiens OX=9606 GN=ZNF345 PE=1 SV=1
A0A087WZT0	2	2	22.005	2.600E-03	3.165E-05	2.142067	0.97514	P-mT-Susp	mT blank	72870.48	Protein Smaug homolog 2 OS=Homo sapiens OX=9606 GN=SAMD4B PE=1 SV=1
F6WQW2;P43	2	2	18.6023	3.390E-04	4.559E-06	2.990076	0.99933	mT blank	P-mT-Susp	32303.51	Ran-specific GTPase-activating protein OS=Homo sapiens OX=9606 GN=RANBP1 PE=1 SV=1
Q5R372	2	2	9.6234	1.549E-04	2.175E-06	2.624754	0.99991	mT blank	F-0.5PG-mT	93425.53	Rab GTPase-activating protein 1-like OS=Homo sapiens OX=9606 GN=RABGAP1L PE=1 SV=1
A0A2R8YG59;	2	2	10.0729	2.209E-05	3.483E-07	1.962513	1	P-mT-Susp	P-0.5PG-mT	42657.28	Oxysterol-binding protein OS=Homo sapiens OX=9606 GN=OSBPL2 PE=1 SV=1
P0C2Y1	2	2	18.1968	3.409E-10	1.731E-11	2.983148	1	P-mT-Susp	mT blank	48603.47	Putative neuroblastoma breakpoint family member 7 OS=Homo sapiens OX=9606 GN=NBPF7 PE=5 SV
Q9Y6D9	2	2	12.4643	7.459E-07	1.466E-08	3.819469	1	F-mT-2D	mT blank	83352.17	Mitotic spindle assembly checkpoint protein MAD1 OS=Homo sapiens OX=9606 GN=MAD1L1 PE=1 SV
P10072	2	2	9.6293	1.801E-05	2.873E-07	3.126691	1	mT blank	P-mT-Susp	77067.24	Zinc finger protein 875 OS=Homo sapiens OX=9606 GN=ZNF875 PE=2 SV=4
D6RCC7;Q9P2	2	2	14.9119	1.016E-05	1.677E-07	1.38149	1	F-0.5PG-mT	F-mT-2D	125104	Protein FAM135A OS=Homo sapiens OX=9606 GN=FAM135A PE=1 SV=1
Q9P2K8;H0YN	2	2	11.2153	8.272E-11	5.813E-12	3.54518	1	P-0.5PG-mT	F-0.5PG-mT	188393.5	eIF-2-alpha kinase GCN2 OS=Homo sapiens OX=9606 GN=EIF2AK4 PE=1 SV=3
A0A7P0T8I1	2	2	15.6744	2.217E-09	8.609E-11	3.245463	1	P-mT-Susp	mT blank	62832.52	Sodium-dependent neutral amino acid transporter B(0)AT2 OS=Homo sapiens OX=9606 GN=SLC6A15
H0YFS2	3	2	35.062	1.271E-11	1.405E-12	5.555911	1	P-mT-Susp	P-0.5PG-mT	26161.72	4F2 cell-surface antigen heavy chain (Fragment) OS=Homo sapiens OX=9606 GN=SLC3A2 PE=1 SV=1
H0YB24;Q8N1	2	2	8.6085	6.664E-02	7.297E-04	3.249324	0.59764	P-0.5PG-mT	mT blank	69382.98	Cell cycle and apoptosis regulator protein 2 (Fragment) OS=Homo sapiens OX=9606 GN=CCAR2 PE=1 S
Q9ULG6	3	2	14.721	4.005E-07	8.238E-09	4.374824	1	P-mT-Susp	P-0.5PG-mT	88081.83	Cell cycle progression protein 1 OS=Homo sapiens OX=9606 GN=CCPG1 PE=1 SV=3
O43306;B3KW	2	2	12.5894	1.278E-01	1.374E-03	3.314811	0.46588	P-mT-Susp	F-0.5PG-mT	132383.5	Adenylate cyclase type 6 OS=Homo sapiens OX=9606 GN=ADCY6 PE=1 SV=2
G8JLP4;Q9Y4	2	2	9.3983	1.333E-06	2.515E-08	46.53284	1	F-mT-2D	P-mT-Susp	177770.4	Limkain-b1 OS=Homo sapiens OX=9606 GN=MARF1 PE=1 SV=1
Q969K3;H7BY	2	2	16.6321	9.432E-12	1.104E-12	3.587789	1	P-mT-Susp	mT blank	42952.41	E3 ubiquitin-protein ligase RNF34 OS=Homo sapiens OX=9606 GN=RNF34 PE=1 SV=1
H0YLK7;H0YK	2	2	10.9123	9.633E-05	1.374E-06	1.564948	0.99998	P-mT-Susp	mT blank	66515.95	Spatacsin (Fragment) OS=Homo sapiens OX=9606 GN=SPG11 PE=1 SV=1
Q7Z5M8	2	2	23.7684	4.702E-06	8.245E-08	1.643184	1	P-mT-Susp	mT blank	41346.68	Protein ABHD12B OS=Homo sapiens OX=9606 GN=ABHD12B PE=1 SV=1
A0A0A0MT64	2	2	12.2124	1.018E-02	1.180E-04	2.091247	0.8875	mT blank	P-mT-Susp	58788.01	NADPH:adrenodoxin oxidoreductase_ mitochondrial OS=Homo sapiens OX=9606 GN=FDXR PE=1 SV=1
Q32P51	2	2	19.0408	1.398E-08	4.107E-10	2.582081	1	F-mT-2D	mT blank	34396.47	Heterogeneous nuclear ribonucleoprotein A1-like 2 OS=Homo sapiens OX=9606 GN=HNRNPA1L2 PE=2
H3BM67;H3B	2	2	18.4614	1.794E-08	5.099E-10	5.805873	1	P-0.5PG-mT	P-mT-Susp	15152.77	Nucleolar protein 3 (Fragment) OS=Homo sapiens OX=9606 GN=NOL3 PE=1 SV=8
E7EVK1	2	2	11.3332	1.802E-03	2.224E-05	42.04637	0.98514	mT blank	F-0.5PG-mT	116488.5	Nuclear receptor corepressor 1 (Fragment) OS=Homo sapiens OX=9606 GN=NCOR1 PE=1 SV=2
E5RFZ5;E5RI0	2	2	13.0526	1.068E-08	3.282E-10	2.893012	1	P-mT-Susp	P-0.5PG-mT	20173.66	Double-strand-break repair protein rad21 homolog OS=Homo sapiens OX=9606 GN=RAD21 PE=1 SV=1
Q96KR7;F6RP	2	2	14.8362	2.258E-06	4.141E-08	1.608271	1	P-0.5PG-mT	F-mT-2D	62723.35	Phosphatase and actin regulator 3 OS=Homo sapiens OX=9606 GN=PHACTR3 PE=1 SV=1
O00409	2	2	10.4737	7.041E-04	9.084E-06	2.001574	0.99693	mT blank	P-mT-Susp	54348.23	Forkhead box protein N3 OS=Homo sapiens OX=9606 GN=FOXN3 PE=1 SV=1
A6NDA9	2	2	10.5755	1.901E-05	3.014E-07	2.877558	1	F-mT-2D	mT blank	61365.67	Leucine-rich repeat_ immunoglobulin-like domain and transmembrane domain-containing protein 2 O
Q13177	2	2	16.3584	2.504E-10	1.394E-11	2.655563	1	P-mT-Susp	mT blank	58327.86	Serine/threonine-protein kinase PAK 2 OS=Homo sapiens OX=9606 GN=PAK2 PE=1 SV=3
A0A590UJN0;	2	2	16.1094	3.729E-09	1.325E-10	1.659466	1	P-0.5PG-mT	F-mT-2D	67810.38	Enhancer of polycomb homolog OS=Homo sapiens OX=9606 GN=EPC1 PE=1 SV=1
A0A7P0T7Z8;	2	2	18.5514	8.795E-03	1.030E-04	1.71233	0.90168	mT blank	F-mT-2D	136809.4	Jouberin OS=Homo sapiens OX=9606 GN=AHI1 PE=4 SV=1
O60309	2	2	13.2129	1.185E-05	1.941E-07	2.130101	1	F-0.5PG-mT	F-mT-2D	181761.7	Leucine-rich repeat-containing protein 37A3 OS=Homo sapiens OX=9606 GN=LRRC37A3 PE=2 SV=2

Q86WZ6	2	2	8.7501	8.879E-11	6.062E-12	3.787073	1	F-mT-2D	F-0.5PG-mT	94656.23	Zinc finger protein 227 OS=Homo sapiens OX=9606 GN=ZNF227 PE=1 SV=1
Q9HCD6	2	2	8.1576	3.158E-08	8.325E-10	41.99076	1	P-mT-Susp	mT blank	221132.7	Protein TANC2 OS=Homo sapiens OX=9606 GN=TANC2 PE=1 SV=3
Q05397	2	2	16.0238	7.582E-13	2.110E-13	6.082905	1	P-mT-Susp	P-0.5PG-mT	120031.6	Focal adhesion kinase 1 OS=Homo sapiens OX=9606 GN=PTK2 PE=1 SV=2
O75976;J3QQ	2	2	10.7389	7.170E-02	7.840E-04	1.322863	0.58321	P-mT-Susp	F-mT-2D	154014.8	Carboxypeptidase D OS=Homo sapiens OX=9606 GN=CPD PE=1 SV=2
A0A7P0T897;	2	2	10.1279	7.848E-06	1.329E-07	1.73651	1	F-mT-2D	P-0.5PG-mT	95069.38	Very low-density lipoprotein receptor OS=Homo sapiens OX=9606 GN=VLDLR PE=4 SV=1
F8VV64;Q63H	2	2	16.3525	2.371E-06	4.328E-08	7.205759	1	P-mT-Susp	P-0.5PG-mT	147745.2	Tensin-2 OS=Homo sapiens OX=9606 GN=TNS2 PE=1 SV=1
Q13574;E9PK	2	2	22.9742	3.906E-09	1.369E-10	1.780986	1	P-0.5PG-mT	F-0.5PG-mT	105463.8	Diacylglycerol kinase zeta OS=Homo sapiens OX=9606 GN=DGKZ PE=1 SV=4
Q96RE7;K7EL	2	2	12.3589	1.475E-10	8.897E-12	2.924349	1	P-mT-Susp	mT blank	57942.7	Nucleus accumbens-associated protein 1 OS=Homo sapiens OX=9606 GN=NACC1 PE=1 SV=1
G3V236;G3V3	2	2	10.7432	2.559E-07	5.447E-09	1.650644	1	P-mT-Susp	mT blank	108375.2	X-linked retinitis pigmentosa GTPase regulator-interacting protein 1 OS=Homo sapiens OX=9606 GN=R
Q14028	2	2	13.8427	2.703E-11	2.562E-12	2.956866	1	P-mT-Susp	mT blank	140590.4	Cyclic nucleotide-gated cation channel beta-1 OS=Homo sapiens OX=9606 GN=CNGB1 PE=1 SV=2
E5RIF2;Q9Y6D	2	2	11.4695	1.834E-11	1.920E-12	4.144889	1	P-mT-Susp	mT blank	147092.4	Brefeldin A-inhibited guanine nucleotide-exchange protein 1 (Fragment) OS=Homo sapiens OX=9606 G
A0A087X0M7	2	2	8.8952	2.285E-02	2.580E-04	1.72036	0.78586	P-mT-Susp	P-0.5PG-mT	68702.19	Protein FAM13C OS=Homo sapiens OX=9606 GN=FAM13C PE=1 SV=1
H3BPL3;A0A7	2	2	10.1524	1.156E-06	2.205E-08	1.717074	1	P-mT-Susp	F-mT-2D	19924.33	Probable E3 ubiquitin-protein ligase makorin-3 OS=Homo sapiens OX=9606 GN=MKRN3 PE=1 SV=1
O15031	2	2	12.7383	1.603E-04	2.242E-06	3.378223	0.9999	mT blank	P-mT-Susp	207864.8	Plexin-B2 OS=Homo sapiens OX=9606 GN=PLXNB2 PE=1 SV=3
F8VUX9;Q96K	2	2	16.1444	3.321E-08	8.668E-10	2.194011	1	P-mT-Susp	mT blank	191045.1	WD repeat-containing protein 90 OS=Homo sapiens OX=9606 GN=WDR90 PE=1 SV=1
Q96L14	2	2	14.7242	1.906E-04	2.629E-06	10.36119	0.99984	mT blank	P-mT-Susp	32648.48	Cep170-like protein OS=Homo sapiens OX=9606 GN=CEP170P1 PE=5 SV=2
H0YBY1;E7EV	2	2	10.6621	1.497E-05	2.418E-07	1.736995	1	P-mT-Susp	mT blank	137082.2	Thyroglobulin (Fragment) OS=Homo sapiens OX=9606 GN=TG PE=1 SV=1
O00267	2	2	10.7901	1.828E-02	2.082E-04	1.418641	0.81781	mT blank	F-mT-2D	121399	Transcription elongation factor SPT5 OS=Homo sapiens OX=9606 GN=SUPT5H PE=1 SV=1
C9IZJ4;Q8N58	2	2	10.1878	6.863E-09	2.239E-10	2.339443	1	P-mT-Susp	mT blank	33286.45	Zinc finger protein 561 (Fragment) OS=Homo sapiens OX=9606 GN=ZNF561 PE=1 SV=1
P50552	2	2	10.2018	8.687E-10	3.821E-11	2.729483	1	P-mT-Susp	mT blank	40000.87	Vasodilator-stimulated phosphoprotein OS=Homo sapiens OX=9606 GN=VASP PE=1 SV=3
Q2M218;A0A	2	2	10.5762	1.552E-09	6.273E-11	3.397638	1	F-0.5PG-mT	F-mT-2D	78204.06	Zinc finger protein 630 OS=Homo sapiens OX=9606 GN=ZNF630 PE=2 SV=1
O95125	2	2	11.0335	2.546E-10	1.408E-11	6.206093	1	P-mT-Susp	F-mT-2D	76089.06	Zinc finger protein 202 OS=Homo sapiens OX=9606 GN=ZNF202 PE=1 SV=4
Q8NB42	2	2	10.2361	5.172E-08	1.275E-09	6.215668	1	P-mT-Susp	P-0.5PG-mT	72670.04	Zinc finger protein 527 OS=Homo sapiens OX=9606 GN=ZNF527 PE=2 SV=2
H0Y3U1	2	2	11.3027	3.121E-08	8.309E-10	3.478073	1	F-0.5PG-mT	F-mT-2D	20659.38	Phosphatase and actin regulator (Fragment) OS=Homo sapiens OX=9606 GN=PHACTR1 PE=1 SV=9
Q719I0	2	2	10.1671	3.614E-09	1.296E-10	4.191572	1	F-mT-2D	F-0.5PG-mT	34034.18	Putative activator of 90 kDa heat shock protein ATPase homolog 2 OS=Homo sapiens OX=9606 GN=AH
A0A0G2JH68;	2	2	9.3344	3.026E-02	3.393E-04	1.587247	0.74159	F-0.5PG-mT	F-mT-2D	142043.1	Protein diaphanous homolog 1 OS=Homo sapiens OX=9606 GN=DIAPH1 PE=1 SV=1
A0A075B6P6;	2	2	11.7066	7.226E-09	2.338E-10	9.883194	1	P-mT-Susp	P-0.5PG-mT	30063.65	Potassium voltage-gated channel subfamily G member 1 (Fragment) OS=Homo sapiens OX=9606 GN=
Q58F21;A0A7	2	2	9.2987	2.511E-09	9.518E-11	3.596649	1	P-mT-Susp	mT blank	108582	Bromodomain testis-specific protein OS=Homo sapiens OX=9606 GN=BRDT PE=1 SV=4
P78524	2	2	9.4378	6.560E-09	2.149E-10	2.022682	1	P-mT-Susp	F-0.5PG-mT	127682.4	DENN domain-containing protein 2B OS=Homo sapiens OX=9606 GN=DENND2B PE=1 SV=3
Q9Y5B0;A0A0	2	2	9.5785	2.194E-02	2.488E-04	3.04928	0.79189	mT blank	P-mT-Susp	105368.8	RNA polymerase II subunit A C-terminal domain phosphatase OS=Homo sapiens OX=9606 GN=CTDP1
E7ES84	2	2	9.1292	1.354E-08	3.993E-10	6.52252	1	P-mT-Susp	mT blank	130643.1	Kinetochore-associated protein 1 OS=Homo sapiens OX=9606 GN=KNTC1 PE=1 SV=1
A0A087WY96	2	2	10.4773	3.773E-05	5.709E-07	2.590362	1	P-0.5PG-mT	P-mT-Susp	82593.84	Transporter OS=Homo sapiens OX=9606 GN=SLC6A6 PE=1 SV=1
C9JID3	2	2	9.8448	1.301E-06	2.466E-08	2.347235	1	P-0.5PG-mT	F-mT-2D	15336.04	Zinc finger protein 490 (Fragment) OS=Homo sapiens OX=9606 GN=ZNF490 PE=1 SV=1
P07550	2	2	17.0797	5.425E-07	1.088E-08	3.225889	1	F-mT-2D	mT blank	47200.11	Beta-2 adrenergic receptor OS=Homo sapiens OX=9606 GN=ADRB2 PE=1 SV=3
B9A061;F8VW	2	2	12.0092	1.660E-03	2.055E-05	2.266408	0.98685	mT blank	P-mT-Susp	54831.16	Calcyphosin-2 OS=Homo sapiens OX=9606 GN=CAPS2 PE=1 SV=1
H7BXR3	2	2	10.0404	1.170E-07	2.662E-09	3.954115	1	P-0.5PG-mT	F-mT-2D	70175.15	Sorbin and SH3 domain-containing protein 2 (Fragment) OS=Homo sapiens OX=9606 GN=SORBS2 PE=
A0A7I2SVS4;P	2	2	11.2454	2.211E-03	2.708E-05	1.74674	0.98008	P-mT-Susp	mT blank	120503	Histone deacetylase OS=Homo sapiens OX=9606 GN=HDAC4 PE=1 SV=1
A0A0U1RRL5;	2	2	10.9988	4.046E-09	1.402E-10	2.619654	1	F-mT-2D	mT blank	67280.69	ETS-related transcription factor Elf-1 (Fragment) OS=Homo sapiens OX=9606 GN=ELF1 PE=1 SV=1
A0A0A0MQW	2	2	10.6801	2.376E-08	6.590E-10	3.003117	1	P-mT-Susp	mT blank	45344.37	Serpin B13 OS=Homo sapiens OX=9606 GN=SERPINB13 PE=1 SV=1
Q9NZ94;E7EV	2	2	11.116	6.417E-03	7.579E-05	2.046881	0.92794	P-mT-Susp	mT blank	94522.75	Neuroligin-3 OS=Homo sapiens OX=9606 GN=NLGN3 PE=1 SV=2
H7C2X8;H7C0	2	2	10.5745	2.291E-06	4.193E-08	1.916076	1	P-0.5PG-mT	P-mT-Susp	28164.47	cAMP-regulated phosphoprotein 21 (Fragment) OS=Homo sapiens OX=9606 GN=ARPP21 PE=1 SV=1
Q9P0L1	2	2	9.7343	2.966E-03	3.588E-05	4.678258	0.97046	P-mT-Susp	F-mT-2D	86972.13	Zinc finger protein with KRAB and SCAN domains 7 OS=Homo sapiens OX=9606 GN=ZKSCAN7 PE=1 SV
P78362	2	2	10.0522	2.246E-07	4.821E-09	1.606724	1	P-mT-Susp	mT blank	78268.13	SRSF protein kinase 2 OS=Homo sapiens OX=9606 GN=SRPK2 PE=1 SV=3
M0R2P6;Q8TE	2	2	16.105	5.645E-05	8.415E-07	1.516601	1	P-mT-Susp	P-0.5PG-mT	74335.05	SH3KBP1 binding protein 1_ isoform CRA_c OS=Homo sapiens OX=9606 GN=SHKBP1 PE=1 SV=1
A0A2R8Y5S6;	2	2	16.13	4.419E-06	7.801E-08	2.572363	1	P-mT-Susp	mT blank	93434.19	Connector enhancer of kinase suppressor of ras 2 OS=Homo sapiens OX=9606 GN=CNKSR2 PE=1 SV=1
B4DNK4;H3BR	2	2	11.49	2.521E-05	3.920E-07	1.46563	1	P-0.5PG-mT	P-mT-Susp	50468.13	Pyruvate kinase OS=Homo sapiens OX=9606 GN=PKM PE=1 SV=1
B9EG95;O957	2	2	11.3104	4.853E-08	1.211E-09	2.175979	1	P-0.5PG-mT	F-mT-2D	61243.2	ZNF682 protein OS=Homo sapiens OX=9606 GN=ZNF682 PE=1 SV=1
O95153;J3KT	2	2	15.3088	3.754E-09	1.328E-10	3.454389	1	P-mT-Susp	mT blank	202389.3	Peripheral-type benzodiazepine receptor-associated protein 1 OS=Homo sapiens OX=9606 GN=TSPOA
Q92835	2	2	10.0672	1.010E-02	1.172E-04	4.015072	0.8883	F-mT-2D	F-0.5PG-mT	134205.1	Phosphatidylinositol 3_4_5-trisphosphate 5-phosphatase 1 OS=Homo sapiens OX=9606 GN=INPP5D P
Q7L2E3	2	2	9.713	3.512E-09	1.265E-10	2.369684	1	P-mT-Susp	mT blank	135021.9	ATP-dependent RNA helicase DHX30 OS=Homo sapiens OX=9606 GN=DHX30 PE=1 SV=1

Q9H2S9	2	2	9.2373	3.307E-06	5.957E-08	4.214425	1	P-0.5PG-mT	P-mT-Susp	65303.65	Zinc finger protein Eos OS=Homo sapiens OX=9606 GN=IKZF4 PE=1 SV=2
A0A087WU35	2	2	16.2414	4.271E-10	2.048E-11	4.438378	1	P-mT-Susp	mT blank	65993.49	Zinc finger protein 714 OS=Homo sapiens OX=9606 GN=ZNF714 PE=1 SV=1
O15438	2	2	15.8885	1.891E-09	7.454E-11	2.579457	1	P-0.5PG-mT	P-mT-Susp	170768.8	ATP-binding cassette sub-family C member 3 OS=Homo sapiens OX=9606 GN=ABCC3 PE=1 SV=3
A0A087WVP9	2	2	10.688	8.430E-02	9.168E-04	1.785558	0.55082	F-mT-2D	mT blank	68108.01	Zinc finger protein 461 OS=Homo sapiens OX=9606 GN=ZNF461 PE=1 SV=1
Q14686	2	2	8.5714	9.151E-07	1.777E-08	8.657734	1	F-0.5PG-mT	F-mT-2D	219488.2	Nuclear receptor coactivator 6 OS=Homo sapiens OX=9606 GN=NCOA6 PE=1 SV=3
Q7Z6E9	2	2	11.8206	1.058E-09	4.529E-11	2.241156	1	P-mT-Susp	mT blank	202477	E3 ubiquitin-protein ligase RBBP6 OS=Homo sapiens OX=9606 GN=RBBP6 PE=1 SV=1
H0Y340	2	2	17.0509	5.077E-12	7.348E-13	7.811291	1	F-0.5PG-mT	F-mT-2D	12853.25	E3 ubiquitin-protein ligase COP1 (Fragment) OS=Homo sapiens OX=9606 GN=COP1 PE=1 SV=1
A0A1B0GV02;	2	2	10.0204	3.726E-07	7.729E-09	1.960073	1	P-0.5PG-mT	P-mT-Susp	140620.5	Zinc finger E-box-binding homeobox 2 OS=Homo sapiens OX=9606 GN=ZEB2 PE=1 SV=1
O75145	2	2	12.9764	1.158E-07	2.642E-09	1.678334	1	P-mT-Susp	F-0.5PG-mT	134066.9	Liprin-alpha-3 OS=Homo sapiens OX=9606 GN=PPFIA3 PE=1 SV=3
Q8TF39;Q6P0	2	2	16.9011	3.174E-08	8.325E-10	2.198982	1	P-mT-Susp	mT blank	87037.1	Zinc finger protein 483 OS=Homo sapiens OX=9606 GN=ZNF483 PE=1 SV=3
Q9BWG4;U3K	2	2	11.2448	1.224E-02	1.412E-04	2.232278	0.86783	F-0.5PG-mT	F-mT-2D	39559.61	Single-stranded DNA-binding protein 4 OS=Homo sapiens OX=9606 GN=SSBP4 PE=1 SV=1
A0A0G2JRB3;/	2	2	10.1639	5.387E-08	1.323E-09	2.3157	1	P-mT-Susp	mT blank	181790.7	Leucine-rich repeat-containing protein 37A OS=Homo sapiens OX=9606 GN=LRRC37A PE=4 SV=1
Q8TAE7	2	2	12.0353	4.244E-11	3.448E-12	4.82283	1	F-0.5PG-mT	F-mT-2D	50391.09	Potassium voltage-gated channel subfamily G member 3 OS=Homo sapiens OX=9606 GN=KCNG3 PE=1
Q9H7Z3	2	2	16.337	4.102E-09	1.407E-10	2.896407	1	P-mT-Susp	P-0.5PG-mT	133927.7	Nuclear exosome regulator NRDE2 OS=Homo sapiens OX=9606 GN=NRDE2 PE=1 SV=3
Q96QB1	2	2	15.1713	1.065E-03	1.346E-05	1.561974	0.99354	P-0.5PG-mT	F-mT-2D	172302.5	Rho GTPase-activating protein 7 OS=Homo sapiens OX=9606 GN=DLC1 PE=1 SV=4
E9PFB9;J3KP9	2	2	9.6716	9.570E-05	1.368E-06	4.999131	0.99998	P-0.5PG-mT	F-mT-2D	152347.3	Coiled-coil domain-containing protein 18 OS=Homo sapiens OX=9606 GN=CCDC18 PE=1 SV=2
Q13275;C9IYS	2	2	9.6359	2.671E-03	3.247E-05	1.268246	0.97423	P-mT-Susp	P-0.5PG-mT	89579.08	Semaphorin-3F OS=Homo sapiens OX=9606 GN=SEMA3F PE=2 SV=2
Q9GZX5	2	2	9.3971	1.551E-09	6.273E-11	2.050785	1	P-mT-Susp	mT blank	61323.05	Zinc finger protein 350 OS=Homo sapiens OX=9606 GN=ZNF350 PE=1 SV=3
A0A075B6F9;I	2	2	14.5679	4.451E-10	2.121E-11	4.925482	1	F-mT-2D	mT blank	33944.48	Nitric oxide synthase-interacting protein OS=Homo sapiens OX=9606 GN=NOSIP PE=1 SV=1
Q9NUT2;C9JY	2	2	10.1126	3.152E-08	8.325E-10	2.357466	1	P-mT-Susp	mT blank	80844.4	Mitochondrial potassium channel ATP-binding subunit OS=Homo sapiens OX=9606 GN=ABCB8 PE=1 S\
I3L3C6;Q9UQI	2	2	18.0324	1.076E-09	4.579E-11	3.621867	1	P-mT-Susp	mT blank	23400.21	Brain-specific angiogenesis inhibitor 1-associated protein 2 (Fragment) OS=Homo sapiens OX=9606 GN
E7EWN3;H0Y3	2	2	9.4427	5.072E-12	7.348E-13	13.79218	1	P-mT-Susp	P-0.5PG-mT	161032.8	Histone-lysine N-methyltransferase SETD5 OS=Homo sapiens OX=9606 GN=SETD5 PE=1 SV=1
A0A6Q8PGJ2;	2	2	9.0743	1.093E-07	2.524E-09	2.504062	1	P-mT-Susp	mT blank	196607.9	Intraflagellar transport protein 172 homolog OS=Homo sapiens OX=9606 GN=IFT172 PE=1 SV=1
Q9NQ66	2	2	9.6187	3.406E-10	1.731E-11	2.944349	1	P-mT-Susp	mT blank	139422.5	1-phosphatidylinositol 4_5-bisphosphate phosphodiesterase beta-1 OS=Homo sapiens OX=9606 GN=P
Q6RI45	2	2	14.4405	3.897E-02	4.345E-04	1.589926	0.69828	P-mT-Susp	P-0.5PG-mT	205365.7	Bromodomain and WD repeat-containing protein 3 OS=Homo sapiens OX=9606 GN=BRWD3 PE=1 SV=
Q13200	2	2	11.8605	1.033E-05	1.698E-07	2.180885	1	P-mT-Susp	mT blank	100941.3	26S proteasome non-ATPase regulatory subunit 2 OS=Homo sapiens OX=9606 GN=PSMD2 PE=1 SV=3
O75936	2	2	9.7799	2.602E-08	7.118E-10	6.485569	1	P-mT-Susp	mT blank	45228.25	Gamma-butyrobetaine dioxygenase OS=Homo sapiens OX=9606 GN=BBOX1 PE=1 SV=1
P59923	2	2	11.1298	5.601E-12	7.714E-13	4.923808	1	P-mT-Susp	mT blank	121358.1	Zinc finger protein 445 OS=Homo sapiens OX=9606 GN=ZNF445 PE=1 SV=1
Q9UJW2;Q5T	3	2	17.2609	1.074E-04	1.527E-06	1.650964	0.99997	F-mT-2D	F-0.5PG-mT	55973.24	Tubulointerstitial nephritis antigen OS=Homo sapiens OX=9606 GN=TINAG PE=2 SV=3
Q8IWJ2	3	2	18.163	3.496E-06	6.268E-08	3.398644	1	P-mT-Susp	mT blank	196993.4	GRIP and coiled-coil domain-containing protein 2 OS=Homo sapiens OX=9606 GN=GCC2 PE=1 SV=4
H0Y2V6	5	2	26.8727	2.942E-14	2.342E-14	17.12667	1	P-mT-Susp	mT blank	172372.6	Centrosomal protein of 170 kDa (Fragment) OS=Homo sapiens OX=9606 GN=CEP170 PE=1 SV=1
A0A0U1RRB6;	3	2	21.3754	1.306E-07	2.945E-09	2.291971	1	P-mT-Susp	mT blank	95427.84	Exocyst complex component OS=Homo sapiens OX=9606 GN=EXOC6B PE=1 SV=1
Q7Z4S6;A0A1	3	2	12.3913	4.477E-09	1.523E-10	3.13047	1	mT blank	F-0.5PG-mT	188491	Kinesin-like protein KIF21A OS=Homo sapiens OX=9606 GN=KIF21A PE=1 SV=2
A0A6Q8PF81;	3	2	25.9885	9.789E-06	1.624E-07	2.473662	1	P-mT-Susp	F-0.5PG-mT	83283.32	Dymeclin OS=Homo sapiens OX=9606 GN=DYM PE=1 SV=1
J3KTM7;O145	3	2	16.3321	5.924E-07	1.182E-08	3.994872	1	F-mT-2D	mT blank	25225.29	Suppressor of cytokine-signaling 6 (Fragment) OS=Homo sapiens OX=9606 GN=SOCS6 PE=1 SV=1
A0A0C4DFX7;	3	2	23.3345	2.273E-05	3.575E-07	1.707086	1	P-mT-Susp	F-mT-2D	63295.48	WD repeat-containing protein 76 OS=Homo sapiens OX=9606 GN=WDR76 PE=1 SV=1
E9PGT3;Q154	3	2	21.2556	3.388E-04	4.559E-06	3.208634	0.99933	mT blank	P-mT-Susp	81870.85	Ribosomal protein S6 kinase OS=Homo sapiens OX=9606 GN=RPS6KA1 PE=1 SV=1
A0A0A0MS79	3	2	21.0152	9.315E-09	2.896E-10	2.409557	1	F-0.5PG-mT	P-mT-Susp	210359.2	Nck-associated protein 5 OS=Homo sapiens OX=9606 GN=NCKAP5 PE=1 SV=1
P51161	3	2	17.0721	9.117E-03	1.063E-04	1.981926	0.89833	mT blank	P-mT-Susp	14371.28	Gastrotropin OS=Homo sapiens OX=9606 GN=FABP6 PE=1 SV=2
Q5SW79	5	2	28.5655	2.849E-03	3.457E-05	3.130037	0.97197	P-0.5PG-mT	F-mT-2D	175692.4	Centrosomal protein of 170 kDa OS=Homo sapiens OX=9606 GN=CEP170 PE=1 SV=1
Q08462;H0YA	3	2	39.4208	2.779E-09	1.039E-10	2.310236	1	P-0.5PG-mT	P-mT-Susp	125257.4	Adenylate cyclase type 2 OS=Homo sapiens OX=9606 GN=ADCY2 PE=1 SV=5
A0A2R8Y6V7;	3	2	16.0522	1.589E-04	2.227E-06	1.341097	0.9999	F-0.5PG-mT	P-mT-Susp	127564.3	Solute carrier family 12 member 1 OS=Homo sapiens OX=9606 GN=SLC12A1 PE=1 SV=1
J3KPH8;Q8WU	3	2	25.6785	3.160E-11	2.858E-12	2.345174	1	P-mT-Susp	mT blank	109993.7	Histone deacetylase OS=Homo sapiens OX=9606 GN=HDAC7 PE=1 SV=1
J3QQM1;J3QS	3	2	21.6642	1.442E-05	2.342E-07	2.028791	1	P-mT-Susp	mT blank	29461.12	26S proteasome regulatory subunit 8 (Fragment) OS=Homo sapiens OX=9606 GN=PSMC5 PE=1 SV=1
P41229	4	2	25.8222	3.728E-07	7.729E-09	2.812523	1	P-mT-Susp	P-0.5PG-mT	178400.9	Lysine-specific demethylase 5C OS=Homo sapiens OX=9606 GN=KDM5C PE=1 SV=2
P32780;E9PM	4	2	36.0373	3.235E-07	6.741E-09	2.661271	1	P-mT-Susp	mT blank	62373.88	General transcription factor IIH subunit 1 OS=Homo sapiens OX=9606 GN=GTF2H1 PE=1 SV=1
H9KV53;Q5U5	4	2	26.7894	2.498E-03	3.045E-05	6.048969	0.97645	P-mT-Susp	F-mT-2D	105605	Cytosolic carboxypeptidase 2 OS=Homo sapiens OX=9606 GN=AGBL2 PE=1 SV=1
A0A087WW66	3	2	29.3953	9.863E-11	6.543E-12	2.631362	1	P-mT-Susp	mT blank	102523.9	26S proteasome non-ATPase regulatory subunit 1 OS=Homo sapiens OX=9606 GN=PSMD1 PE=1 SV=2
O00410;H0Y8	5	2	31.2083	1.991E-02	2.261E-04	2.102044	0.80591	P-mT-Susp	F-0.5PG-mT	125113	Importin-5 OS=Homo sapiens OX=9606 GN=IPO5 PE=1 SV=4
Q7Z406;M0Q	4	2	33.5937	4.607E-11	3.561E-12	2.297648	1	F-mT-2D	mT blank	228840.3	Myosin-14 OS=Homo sapiens OX=9606 GN=MYH14 PE=1 SV=2

A8K2U0;H0YG	3	2	13.3584	2.662E-07	5.621E-09	2.625301	1	F-mT-2D	F-0.5PG-mT	162532.5	Alpha-2-macroglobulin-like protein 1 OS=Homo sapiens OX=9606 GN=A2ML1 PE=1 SV=3
A0A087WZ84;	3	2	15.995	3.658E-12	6.196E-13	3.156643	1	P-mT-Susp	mT blank	76193.93	Zinc finger protein 568 OS=Homo sapiens OX=9606 GN=ZNF568 PE=1 SV=1
Q9P227	3	2	17.4584	5.370E-05	8.050E-07	1.836565	1	mT blank	F-mT-2D	163446.7	Rho GTPase-activating protein 23 OS=Homo sapiens OX=9606 GN=ARHGAP23 PE=1 SV=2
Q9UBW7;A0A	5	2	32.4189	1.037E-03	1.316E-05	1.472386	0.99384	mT blank	P-mT-Susp	158503.8	Zinc finger MYM-type protein 2 OS=Homo sapiens OX=9606 GN=ZMYM2 PE=1 SV=1
A0A384DVL6;	3	2	16.951	7.856E-09	2.522E-10	4.281279	1	P-mT-Susp	mT blank	223348.5	Nuclear receptor corepressor 2 OS=Homo sapiens OX=9606 GN=NCOR2 PE=1 SV=1
Q96E39	6	2	28.033	5.451E-08	1.335E-09	3.602489	1	F-mT-2D	mT blank	42198.66	RNA binding motif protein_ X-linked-like-1 OS=Homo sapiens OX=9606 GN=RBMXL1 PE=1 SV=1
Q9NQI0;D6RC	3	2	21.138	1.644E-09	6.543E-11	2.16717	1	P-mT-Susp	mT blank	80163.05	Probable ATP-dependent RNA helicase DDX4 OS=Homo sapiens OX=9606 GN=DDX4 PE=1 SV=2
Q9Y2J0;F8W1	5	2	38.0976	8.267E-06	1.388E-07	5.014024	1	mT blank	P-mT-Susp	77728.02	Rabphilin-3A OS=Homo sapiens OX=9606 GN=RPH3A PE=1 SV=1
H7C1F9;Q2PP	6	2	42.6889	1.852E-07	4.039E-09	3.358802	1	P-mT-Susp	mT blank	197269.8	Ral GTPase-activating protein subunit alpha-2 (Fragment) OS=Homo sapiens OX=9606 GN=RALGAPA2
E7ERS3;Q86V	3	2	21.18	4.657E-07	9.506E-09	4.407742	1	P-0.5PG-mT	P-mT-Susp	109167.7	Zinc finger CCCH domain-containing protein 18 OS=Homo sapiens OX=9606 GN=ZC3H18 PE=1 SV=1
P82970	5	2	24.3742	7.978E-06	1.348E-07	3.374702	1	P-0.5PG-mT	P-mT-Susp	31524.64	High mobility group nucleosome-binding domain-containing protein 5 OS=Homo sapiens OX=9606 GN
A0A0A0MQS9	3	2	13.2914	3.864E-10	1.887E-11	3.046207	1	P-mT-Susp	mT blank	205191.8	Laminin subunit alpha-4 OS=Homo sapiens OX=9606 GN=LAMA4 PE=1 SV=1
A0A075B6E9;	3	2	18.5856	8.843E-05	1.275E-06	3.091022	0.99998	mT blank	P-mT-Susp	169302.4	Leucine-rich repeat-containing protein 7 OS=Homo sapiens OX=9606 GN=LRRC7 PE=1 SV=1
C9JL08	3	2	17.5681	1.173E-01	1.266E-03	3.414996	0.48342	mT blank	F-0.5PG-mT	27977.9	1-phosphatidylinositol 3-phosphate 5-kinase (Fragment) OS=Homo sapiens OX=9606 GN=PIKFYVE PE=
A0A0G2JPG6;	3	2	26.1567	3.215E-12	5.816E-13	7.066527	1	P-mT-Susp	P-0.5PG-mT	46005.58	Killer cell immunoglobulin-like receptor 2DL4 OS=Homo sapiens OX=9606 GN=KIR2DL4 PE=1 SV=1
A0A7I2PHE7;	5	2	21.8802	2.439E-08	6.742E-10	3.843783	1	P-mT-Susp	mT blank	144938.3	Centrosome and spindle pole-associated protein 1 OS=Homo sapiens OX=9606 GN=CSPP1 PE=1 SV=1
K7ENM7;Q9U	3	2	20.5353	3.878E-09	1.366E-10	3.925647	1	P-mT-Susp	mT blank	70678.58	Uncharacterized protein OS=Homo sapiens OX=9606 PE=4 SV=1
A0A0C4DG86;	3	2	24.9062	5.112E-02	5.652E-04	1.216786	0.64868	F-0.5PG-mT	F-mT-2D	73795.45	Syntabulin OS=Homo sapiens OX=9606 GN=SYBU PE=1 SV=1
H0Y486;Q5TA	3	2	22.9725	1.335E-09	5.534E-11	7.672899	1	P-mT-Susp	P-0.5PG-mT	19601.58	Tetratricopeptide repeat protein 22 (Fragment) OS=Homo sapiens OX=9606 GN=TTC22 PE=1 SV=1
C9JFJ0;D6REE	3	2	16.5191	6.533E-04	8.470E-06	1.350161	0.99734	F-0.5PG-mT	F-mT-2D	23440.55	Rho-associated protein kinase 2 (Fragment) OS=Homo sapiens OX=9606 GN=ROCK2 PE=1 SV=1
Q9HCD5	3	2	28.8987	1.701E-07	3.771E-09	2.325792	1	P-mT-Susp	mT blank	65764.68	Nuclear receptor coactivator 5 OS=Homo sapiens OX=9606 GN=NCOA5 PE=1 SV=2
P23142;B1AH	3	2	15.885	8.413E-11	5.824E-12	7.690768	1	F-0.5PG-mT	F-mT-2D	81320.11	Fibulin-1 OS=Homo sapiens OX=9606 GN=FBLN1 PE=1 SV=4
E7EVQ6;Q145	3	2	20.8069	2.712E-10	1.479E-11	4.036669	1	P-mT-Susp	mT blank	53352.77	Squalene monooxygenase OS=Homo sapiens OX=9606 GN=SQLE PE=1 SV=1
A0A494C0S0;	3	2	16.2927	2.261E-08	6.314E-10	2.759442	1	P-mT-Susp	mT blank	115065.2	Sorting nexin-25 OS=Homo sapiens OX=9606 GN=SNX25 PE=1 SV=1
A0A7I2YQ90;	3	2	30.4424	8.871E-10	3.880E-11	2.150588	1	P-mT-Susp	mT blank	67318.15	Polyadenylate-binding protein 1 OS=Homo sapiens OX=9606 GN=PABPC1 PE=1 SV=1
Q6KB66	3	2	32.4368	8.418E-04	1.079E-05	2.335396	0.99573	P-0.5PG-mT	F-mT-2D	51038.63	Keratin_ type II cytoskeletal 80 OS=Homo sapiens OX=9606 GN=KRT80 PE=1 SV=2
A0A494C0Q6	4	2	30.5764	1.392E-13	6.498E-14	5.0582	1	P-mT-Susp	mT blank	67136.39	Poly(A)-specific ribonuclease PARN OS=Homo sapiens OX=9606 GN=PARN PE=1 SV=1
Q9NZQ3;C9JS	5	2	31.0377	3.053E-02	3.418E-04	1.353306	0.74014	F-0.5PG-mT	P-mT-Susp	79701.73	NCK-interacting protein with SH3 domain OS=Homo sapiens OX=9606 GN=NCKIPSD PE=1 SV=1
O75197	4	2	13.5387	4.055E-09	1.402E-10	2.60317	1	P-mT-Susp	mT blank	182395.4	Low-density lipoprotein receptor-related protein 5 OS=Homo sapiens OX=9606 GN=LRP5 PE=1 SV=2
A0A0U1RR27;	3	2	15.4897	8.579E-10	3.794E-11	2.120215	1	P-mT-Susp	mT blank	217446.6	C-myc promoter-binding protein OS=Homo sapiens OX=9606 GN=DENND4A PE=1 SV=2
Q8N823;A0A1	3	2	17.1704	1.582E-06	2.956E-08	1.608426	1	P-mT-Susp	F-0.5PG-mT	84186.86	Zinc finger protein 611 OS=Homo sapiens OX=9606 GN=ZNF611 PE=1 SV=2
A6NHJ4	3	2	17.1209	1.014E-05	1.677E-07	Infinity	1	P-0.5PG-mT	P-mT-Susp	75638.66	Zinc finger protein 860 OS=Homo sapiens OX=9606 GN=ZNF860 PE=1 SV=3
P54753	3	2	15.043	1.540E-02	1.764E-04	1.181895	0.84043	mT blank	F-0.5PG-mT	111926.8	Ephrin type-B receptor 3 OS=Homo sapiens OX=9606 GN=EPHB3 PE=1 SV=2
J3QT46;Q9Y4	3	2	17.7933	8.251E-06	1.388E-07	1.902652	1	P-mT-Susp	mT blank	68748.99	HBS1-like protein OS=Homo sapiens OX=9606 GN=HBS1L PE=1 SV=1
A0A3B3ITH6;	3	2	15.5567	6.241E-07	1.242E-08	12.50759	1	mT blank	P-mT-Susp	33811.82	Zinc finger imprinted 2 OS=Homo sapiens OX=9606 GN=ZIM2 PE=4 SV=1
A0A087WUR8	3	2	29.6491	4.436E-04	5.866E-06	2.183235	0.99879	F-0.5PG-mT	F-mT-2D	47345.47	Inactive serine protease PAMR1 OS=Homo sapiens OX=9606 GN=PAMR1 PE=1 SV=1
A0A590UJQ1;	4	2	27.8774	1.314E-03	1.648E-05	1.64458	0.99086	P-mT-Susp	mT blank	103749.5	E3 ubiquitin-protein ligase OS=Homo sapiens OX=9606 GN=ITCH PE=1 SV=1
A0A2R8YGD3;	8	2	44.3865	2.915E-10	1.568E-11	6.521009	1	P-0.5PG-mT	F-0.5PG-mT	189394.2	Cyclic nucleotide ras GEF OS=Homo sapiens OX=9606 GN=RAPGEF2 PE=1 SV=1
Q9UBB9	3	2	23.0383	5.712E-06	9.927E-08	1.944462	1	P-mT-Susp	mT blank	97219.18	Tuftelin-interacting protein 11 OS=Homo sapiens OX=9606 GN=TFIP11 PE=1 SV=1
P18433;Q5JW	3	2	21.4604	3.659E-05	5.548E-07	4.368115	1	P-0.5PG-mT	F-mT-2D	91631.64	Receptor-type tyrosine-protein phosphatase alpha OS=Homo sapiens OX=9606 GN=PTPRA PE=1 SV=3
Q8TDI0;K7EM	4	2	20.0255	2.879E-05	4.416E-07	1.783679	1	P-0.5PG-mT	mT blank	224646.7	Chromodomain-helicase-DNA-binding protein 5 OS=Homo sapiens OX=9606 GN=CHD5 PE=1 SV=1
A0A2R8Y555;	3	2	14.388	1.264E-08	3.779E-10	2.231796	1	P-mT-Susp	mT blank	65624.68	Calcium channel voltage-dependent subunit beta 2 (Fragment) OS=Homo sapiens OX=9606 GN=CACN
F8WFA6	3	2	16.0901	3.026E-04	4.097E-06	2.792986	0.99948	P-mT-Susp	F-mT-2D	14952.92	E3 ubiquitin-protein ligase RAD18 OS=Homo sapiens OX=9606 GN=RAD18 PE=1 SV=1
Q16825	4	2	18.1794	1.735E-10	1.016E-11	5.670719	1	P-mT-Susp	P-0.5PG-mT	134193.7	Tyrosine-protein phosphatase non-receptor type 21 OS=Homo sapiens OX=9606 GN=PTPN21 PE=1 SV
Q9Y263;E5RI	3	2	24.9959	6.294E-07	1.249E-08	3.100183	1	P-mT-Susp	F-0.5PG-mT	88696.94	Phospholipase A-2-activating protein OS=Homo sapiens OX=9606 GN=PLAA PE=1 SV=2
B1AKL4;Q9NR	4	2	29.0128	4.262E-10	2.048E-11	2.790955	1	P-mT-Susp	mT blank	105892.1	Eukaryotic translation initiation factor 4E transporter OS=Homo sapiens OX=9606 GN=EIF4ENIF1 PE=1
A0A384DVU0;	4	2	21.0952	1.834E-07	4.010E-09	3.925898	1	P-mT-Susp	mT blank	151406.6	Patatin-like phospholipase domain-containing protein 6 OS=Homo sapiens OX=9606 GN=PNPLA6 PE=1
F5GWN5;O00	3	2	20.281	1.124E-03	1.419E-05	2.624994	0.99293	P-0.5PG-mT	P-mT-Susp	183796.5	Phosphatidylinositol-4-phosphate 3-kinase OS=Homo sapiens OX=9606 GN=PIK3C2B PE=1 SV=1
Q01118	4	2	26.1316	3.917E-06	6.975E-08	9.619152	1	P-0.5PG-mT	P-mT-Susp	195775	Sodium channel protein type 7 subunit alpha OS=Homo sapiens OX=9606 GN=SCN7A PE=1 SV=2
Q13591;D6RA	4	2	33.3193	8.273E-11	5.813E-12	3.346832	1	P-0.5PG-mT	F-0.5PG-mT	124151.1	Semaphorin-5A OS=Homo sapiens OX=9606 GN=SEMA5A PE=1 SV=3

F5H658;Q145	3	2	15.6861	6.839E-06	1.168E-07	3.803921	1	P-mT-Susp	P-0.5PG-mT	135028.3	RNA helicase OS=Homo sapiens OX=9606 GN=DHX8 PE=1 SV=1
E7EU96;A0A2	4	2	22.4696	2.109E-12	4.419E-13	9.980005	1	P-mT-Susp	P-0.5PG-mT	45482.08	Casein kinase II subunit alpha OS=Homo sapiens OX=9606 GN=CSNK2A1 PE=1 SV=1
Q16821;C9JZE	3	2	21.0383	5.584E-04	7.303E-06	1.720395	0.99804	P-0.5PG-mT	P-mT-Susp	127078.5	Protein phosphatase 1 regulatory subunit 3A OS=Homo sapiens OX=9606 GN=PPP1R3A PE=1 SV=3
Q96LW9;C9JP	3	2	22.7231	8.180E-09	2.615E-10	3.336398	1	P-mT-Susp	P-0.5PG-mT	48262.58	Zinc finger and SCAN domain-containing protein 31 OS=Homo sapiens OX=9606 GN=ZSCAN31 PE=1 SV
J3KN75;Q0IIM	3	2	15.5388	1.347E-03	1.683E-05	1.388116	0.9905	F-0.5PG-mT	F-mT-2D	129591.2	TBC1 domain family member 8B OS=Homo sapiens OX=9606 GN=TBC1D8B PE=1 SV=1
Q9H3C7	3	2	29.6815	2.226E-02	2.521E-04	1.390308	0.78972	P-0.5PG-mT	F-mT-2D	81367.64	Gametogenetin-binding protein 2 OS=Homo sapiens OX=9606 GN=GGNBP2 PE=1 SV=1
P05165;A0A2	3	2	21.4325	3.184E-05	4.865E-07	1.775318	1	P-mT-Susp	mT blank	80686.4	Propionyl-CoA carboxylase alpha chain_ mitochondrial OS=Homo sapiens OX=9606 GN=PCCA PE=1 SV
A0A140TA76;	3	2	24.5081	2.280E-04	3.124E-06	3.17403	0.99974	mT blank	P-mT-Susp	38247.05	Protein LSM14 homolog A (Fragment) OS=Homo sapiens OX=9606 GN=LSM14A PE=1 SV=1
D6REX3;H7BX	3	2	19.7986	2.348E-04	3.211E-06	1.364007	0.99972	P-mT-Susp	P-0.5PG-mT	137196	Protein transport protein Sec31A OS=Homo sapiens OX=9606 GN=SEC31A PE=1 SV=1
Q96JM4	4	2	20.5344	1.664E-05	2.665E-07	2.138493	1	mT blank	P-mT-Susp	201410.6	Leucine-rich repeat and IQ domain-containing protein 1 OS=Homo sapiens OX=9606 GN=LRRIQ1 PE=2
Q5TYW1	3	2	14.9885	5.651E-10	2.600E-11	23.63215	1	P-0.5PG-mT	P-mT-Susp	125752.9	Zinc finger protein 658 OS=Homo sapiens OX=9606 GN=ZNF658 PE=1 SV=2
A0A087X0R0;	4	2	33.8565	3.103E-08	8.289E-10	3.952257	1	P-0.5PG-mT	mT blank	161727.1	Lysine-specific demethylase 6A OS=Homo sapiens OX=9606 GN=KDM6A PE=1 SV=2
Q96SB8;A0A0	3	2	23.9058	6.599E-13	1.946E-13	2.611938	1	P-mT-Susp	mT blank	127295.3	Structural maintenance of chromosomes protein 6 OS=Homo sapiens OX=9606 GN=SMC6 PE=1 SV=2
O15068	3	2	14.303	1.810E-06	3.351E-08	2.798997	1	P-mT-Susp	P-0.5PG-mT	129420.9	Guanine nucleotide exchange factor DBS OS=Homo sapiens OX=9606 GN=MCF2L PE=1 SV=2
X5D778	3	2	20.9132	2.006E-08	5.642E-10	3.923971	1	F-mT-2D	P-0.5PG-mT	47447.7	Ankyrin repeat domain 11 isoform A (Fragment) OS=Homo sapiens OX=9606 GN=ANKRD11 PE=1 SV=1
A0A2R8Y7T1;	4	2	22.6006	1.922E-06	3.550E-08	1.555376	1	P-mT-Susp	mT blank	17402.71	Casein kinase II subunit alpha OS=Homo sapiens OX=9606 GN=CSNK2A1 PE=4 SV=1
F5H7W8;Q96	3	2	23.5628	6.127E-04	7.969E-06	2.513253	0.99765	F-mT-2D	F-0.5PG-mT	31921.92	Protein CUSTOS OS=Homo sapiens OX=9606 GN=C12orf43 PE=1 SV=2
A0A087WVK0	3	2	24.9158	2.407E-06	4.385E-08	Infinity	1	mT blank	P-mT-Susp	54479.12	Dual-specificity protein kinase CLK1 OS=Homo sapiens OX=9606 GN=CLK1 PE=1 SV=1
Q5H9U9;D6R	3	2	15.3817	3.414E-10	1.731E-11	3.593144	1	P-mT-Susp	mT blank	199955	Probable ATP-dependent RNA helicase DDX60-like OS=Homo sapiens OX=9606 GN=DDX60L PE=2 SV=
A0A7P0T9G4;	3	2	23.2345	1.976E-14	1.748E-14	32.99023	1	P-mT-Susp	P-0.5PG-mT	163532	Chimeric ERCC6-PGBD3 protein OS=Homo sapiens OX=9606 GN=ERCC6 PE=4 SV=1
Q9H7N4	4	2	23.8258	4.131E-05	6.228E-07	1.515728	1	P-0.5PG-mT	P-mT-Susp	139897.3	Splicing factor_ arginine/serine-rich 19 OS=Homo sapiens OX=9606 GN=SCAF1 PE=1 SV=3
D6RAM3;D6R	1	1	4.9938	1.155E-07	2.642E-09	2.566336	1	P-mT-Susp	mT blank	48286.3	Docking protein 3 OS=Homo sapiens OX=9606 GN=DOK3 PE=1 SV=1
A0A087WWR	1	1	4.7133	2.871E-09	1.063E-10	7.4912	1	P-mT-Susp	mT blank	12978.98	Kidney mitochondrial carrier protein 1 (Fragment) OS=Homo sapiens OX=9606 GN=SLC25A30 PE=1 SV
D6R905;D6RA	1	1	5.4118	6.560E-02	7.193E-04	1.615141	0.60074	P-mT-Susp	mT blank	26228.23	Exosome complex component RRP45 (Fragment) OS=Homo sapiens OX=9606 GN=EXOSC9 PE=1 SV=1
Q14192	1	1	4.0838	3.251E-04	4.386E-06	3.304778	0.99939	mT blank	P-mT-Susp	34188.85	Four and a half LIM domains protein 2 OS=Homo sapiens OX=9606 GN=FHL2 PE=1 SV=3
F8VXK3;Q9NP	1	1	6.1115	3.374E-10	1.731E-11	5.504427	1	P-mT-Susp	mT blank	18754.53	Carbohydrate sulfotransferase (Fragment) OS=Homo sapiens OX=9606 GN=CHST11 PE=1 SV=1
K7EJE5	1	1	4.9145	4.537E-06	7.973E-08	2.32586	1	F-0.5PG-mT	F-mT-2D	13227.25	Zinc finger protein 233 (Fragment) OS=Homo sapiens OX=9606 GN=ZNF233 PE=4 SV=1
H3BR66;P082	1	1	4.2071	7.604E-05	1.107E-06	1.577548	0.99999	F-mT-2D	mT blank	23175.43	PEX (Fragment) OS=Homo sapiens OX=9606 GN=MMP2 PE=1 SV=1
Q5TA45;Q96H	1	1	5.4282	1.986E-08	5.606E-10	3.240922	1	P-mT-Susp	mT blank	68404.09	Integrator complex subunit 11 OS=Homo sapiens OX=9606 GN=INTS11 PE=1 SV=2
Q9Y6N8	1	1	4.4631	6.562E-07	1.296E-08	2.347881	1	P-0.5PG-mT	P-mT-Susp	88850.63	Cadherin-10 OS=Homo sapiens OX=9606 GN=CDH10 PE=1 SV=2
A0A087WTR9	1	1	5.251	1.153E-07	2.642E-09	4.165818	1	F-0.5PG-mT	F-mT-2D	30833.28	Protein Mdm4 OS=Homo sapiens OX=9606 GN=MDM4 PE=1 SV=1
E7EWD6	1	1	4.2484	1.576E-08	4.529E-10	5.308744	1	P-0.5PG-mT	P-mT-Susp	151777.8	Putative Polycomb group protein ASXL2 OS=Homo sapiens OX=9606 GN=ASXL2 PE=1 SV=1
H7C0W8	1	1	14.4966	7.028E-14	4.501E-14	15.60326	1	P-mT-Susp	P-0.5PG-mT	11955.23	Adipocyte enhancer-binding protein 1 (Fragment) OS=Homo sapiens OX=9606 GN=AEBP1 PE=1 SV=1
A0A0B4J2A4;	1	1	5.7775	7.778E-13	2.110E-13	6.146567	1	P-mT-Susp	mT blank	42058.06	3-ketoacyl-CoA thiolase_ mitochondrial OS=Homo sapiens OX=9606 GN=ACAA2 PE=1 SV=1
Q13099	1	1	4.4386	3.495E-10	1.761E-11	4.976847	1	P-0.5PG-mT	F-mT-2D	93761.95	Intraflagellar transport protein 88 homolog OS=Homo sapiens OX=9606 GN=IFT88 PE=1 SV=3
D6RB01;Q9Y6	1	1	5.2104	6.315E-03	7.470E-05	2.180956	0.92913	F-0.5PG-mT	P-mT-Susp	68340.1	Soluble lamin-associated protein of 75 kDa OS=Homo sapiens OX=9606 GN=FAM169A PE=1 SV=1
H0Y860;H7C5	1	1	5.0503	2.407E-12	4.862E-13	15.09355	1	P-mT-Susp	mT blank	85672.66	Stromal interaction molecule 2 OS=Homo sapiens OX=9606 GN=STIM2 PE=1 SV=2
A0A075B771;	1	1	5.6413	6.237E-12	7.858E-13	3.354335	1	P-mT-Susp	mT blank	21318.93	Zinc finger protein 582 OS=Homo sapiens OX=9606 GN=ZNF582 PE=4 SV=1
A0A7P0N7C4;	1	1	11.439	6.756E-04	8.745E-06	2.407202	0.99716	F-0.5PG-mT	P-mT-Susp	215996.8	Zinc finger protein 142 OS=Homo sapiens OX=9606 GN=ZNF142 PE=4 SV=1
Q15424	1	1	4.0321	1.011E-03	1.286E-05	2.498548	0.99409	mT blank	P-mT-Susp	103098	Scaffold attachment factor B1 OS=Homo sapiens OX=9606 GN=SAFB PE=1 SV=4
A0A590UJ62;	1	1	5.5654	3.873E-08	9.945E-10	3.054946	1	F-0.5PG-mT	F-mT-2D	82509.89	Potassium voltage-gated channel subfamily C member 3 OS=Homo sapiens OX=9606 GN=KCNC3 PE=1
O94888	1	1	5.8316	3.856E-05	5.824E-07	1.540558	1	F-0.5PG-mT	F-mT-2D	55261.61	UBX domain-containing protein 7 OS=Homo sapiens OX=9606 GN=UBXN7 PE=1 SV=2
K7EJC2;Q9P1	1	1	5.0763	9.118E-09	2.846E-10	2.864647	1	F-mT-2D	P-mT-Susp	33709.49	GEM-interacting protein (Fragment) OS=Homo sapiens OX=9606 GN=GMIP PE=1 SV=1
O95377	1	1	5.0207	1.347E-10	8.311E-12	3.144201	1	P-mT-Susp	mT blank	31829.7	Gap junction beta-5 protein OS=Homo sapiens OX=9606 GN=GJB5 PE=1 SV=2
K7ERI5	1	1	5.4254	2.572E-09	9.705E-11	5.303257	1	P-mT-Susp	mT blank	18494.12	KRAB domain-containing protein (Fragment) OS=Homo sapiens OX=9606 PE=4 SV=1
Q9Y3Q7	1	1	5.1575	3.406E-04	4.564E-06	2.627728	0.99932	mT blank	P-mT-Susp	85365.8	Disintegrin and metalloproteinase domain-containing protein 18 OS=Homo sapiens OX=9606 GN=ADA
Q9H7X2	1	1	14.3584	8.462E-12	1.005E-12	3.215895	1	P-0.5PG-mT	P-mT-Susp	15573.66	Uncharacterized protein C1orf115 OS=Homo sapiens OX=9606 GN=C1orf115 PE=2 SV=1
A0A087WZG5	1	1	13.1678	7.672E-07	1.504E-08	2.258335	1	P-mT-Susp	F-0.5PG-mT	17368.96	WW domain-containing oxidoreductase OS=Homo sapiens OX=9606 GN=WWOX PE=1 SV=1
Q6NY19	1	1	4.9551	6.899E-06	1.176E-07	1.706809	1	F-0.5PG-mT	P-mT-Susp	89052.75	KN motif and ankyrin repeat domain-containing protein 3 OS=Homo sapiens OX=9606 GN=KANK3 PE=
A0A0A0MRF9	1	1	4.2517	2.410E-01	2.565E-03	1.198975	0.33624	F-0.5PG-mT	F-mT-2D	147257.6	1-phosphatidylinositol 4_5-bisphosphate phosphodiesterase gamma OS=Homo sapiens OX=9606 GN=

Q12965	1	1	5.1929	1.007E-07	2.344E-09	1.793572	1	P-mT-Susp	mT blank	127632.4	Unconventional myosin-Ie OS=Homo sapiens OX=9606 GN=MYO1E PE=1 SV=2
Q86WV1	1	1	5.0152	3.164E-08	8.325E-10	2.100201	1	F-mT-2D	mT blank	41717.65	Src kinase-associated phosphoprotein 1 OS=Homo sapiens OX=9606 GN=SKAP1 PE=1 SV=3
A0A0J9YWN0	1	1	9.1398	6.681E-03	7.879E-05	1.680622	0.9249	F-mT-2D	F-0.5PG-mT	21260.26	Band 4.1-like protein 3 (Fragment) OS=Homo sapiens OX=9606 GN=EPB41L3 PE=1 SV=3
I3L3E0	1	1	6.522	3.465E-12	5.997E-13	9.294036	1	P-mT-Susp	P-0.5PG-mT	18077.55	Uromodulin (Fragment) OS=Homo sapiens OX=9606 GN=UMOD PE=1 SV=1
H0Y7V2	1	1	12.9978	2.586E-10	1.420E-11	2.696003	1	P-mT-Susp	mT blank	11742.43	Small G protein-signaling modulator 3 (Fragment) OS=Homo sapiens OX=9606 GN=SGSM3 PE=1 SV=1
J3KR97;Q9BTV	1	1	9.9668	2.405E-11	2.335E-12	2.926923	1	P-mT-Susp	mT blank	138433.6	Tubulin-specific chaperone D OS=Homo sapiens OX=9606 GN=TBCD PE=1 SV=1
A0A0U1RQQ9	1	1	4.1252	1.493E-13	6.498E-14	6.850248	1	P-mT-Susp	mT blank	104796.7	SCY1-like protein 2 OS=Homo sapiens OX=9606 GN=SCYL2 PE=1 SV=1
Q8NGT0	1	1	12.1578	8.766E-06	1.463E-07	9.303802	1	P-0.5PG-mT	F-mT-2D	36310.2	Olfactory receptor 13C9 OS=Homo sapiens OX=9606 GN=OR13C9 PE=3 SV=1
P61978;Q5T6V	1	1	6.265	6.898E-03	8.123E-05	1.924946	0.92242	mT blank	P-mT-Susp	51261.48	Heterogeneous nuclear ribonucleoprotein K OS=Homo sapiens OX=9606 GN=HNRNPK PE=1 SV=1
F5H5K1;J3QL1	1	1	13.2334	1.303E-04	1.840E-06	2.793216	0.99994	P-mT-Susp	mT blank	97345.63	Leucine-rich repeat-containing protein 37B OS=Homo sapiens OX=9606 GN=LRRC37B PE=1 SV=1
Q6UWB1	1	1	5.0668	3.179E-08	8.325E-10	3.96098	1	P-mT-Susp	P-0.5PG-mT	70386.31	Interleukin-27 receptor subunit alpha OS=Homo sapiens OX=9606 GN=IL27RA PE=1 SV=2
H0YBC9;Q141	1	1	4.4962	9.660E-08	2.275E-09	1.859671	1	P-0.5PG-mT	F-mT-2D	54994.97	DNA repair-scaffolding protein (Fragment) OS=Homo sapiens OX=9606 GN=SPIDR PE=1 SV=1
Q5VZT6;Q8IYX	1	1	5.4742	1.208E-04	1.708E-06	2.811815	0.99995	F-0.5PG-mT	P-mT-Susp	8942.111	Stabilizer of axonemal microtubules 1 OS=Homo sapiens OX=9606 GN=SAXO1 PE=1 SV=2
P17036;Q86U	1	1	8.724	2.113E-13	8.409E-14	12.06773	1	F-mT-2D	mT blank	51885.42	Zinc finger protein 3 OS=Homo sapiens OX=9606 GN=ZNF3 PE=1 SV=3
E9PJF3;E9PP5	1	1	12.9408	1.962E-09	7.692E-11	3.601636	1	P-mT-Susp	mT blank	32134.69	Flavin-containing monooxygenase OS=Homo sapiens OX=9606 GN=FMO5 PE=1 SV=1
Q8IVP9	1	1	10.1512	2.535E-06	4.597E-08	2.578831	1	P-mT-Susp	mT blank	47496.06	Zinc finger protein 547 OS=Homo sapiens OX=9606 GN=ZNF547 PE=1 SV=2
P26374	1	1	12.213	1.571E-08	4.529E-10	2.462015	1	P-mT-Susp	F-0.5PG-mT	75326.13	Rab proteins geranylgeranyltransferase component A 2 OS=Homo sapiens OX=9606 GN=CHML PE=1 S
A0A1W2PR36	1	1	6.6248	9.927E-04	1.264E-05	2.491162	0.99428	P-mT-Susp	mT blank	24032.41	Guanidinoacetate N-methyltransferase OS=Homo sapiens OX=9606 GN=GAMT PE=1 SV=1
P22460	1	1	5.6422	6.613E-08	1.600E-09	2.503939	1	P-mT-Susp	mT blank	67798.17	Potassium voltage-gated channel subfamily A member 5 OS=Homo sapiens OX=9606 GN=KCNA5 PE=1
A8MQD4	1	1	6.479	5.004E-11	3.830E-12	9.900773	1	P-mT-Susp	P-0.5PG-mT	16091.07	Mesoderm induction early response protein 3 (Fragment) OS=Homo sapiens OX=9606 GN=MIER3 PE=
P23229	1	1	5.606	2.363E-02	2.665E-04	1.393212	0.78078	F-0.5PG-mT	F-mT-2D	127803.2	Integrin alpha-6 OS=Homo sapiens OX=9606 GN=ITGA6 PE=1 SV=5
A0A7P0T8R2	1	1	12.7432	7.166E-08	1.718E-09	5.055366	1	P-0.5PG-mT	P-mT-Susp	5380.803	Excitatory amino acid transporter 1 (Fragment) OS=Homo sapiens OX=9606 GN=SLC1A3 PE=4 SV=1
A6NJL1;K7EJD	1	1	4.8159	3.763E-06	6.716E-08	63.79619	1	P-0.5PG-mT	P-mT-Susp	56681.41	Zinc finger and SCAN domain-containing protein 5B OS=Homo sapiens OX=9606 GN=ZSCAN5B PE=1 SV
J3QQY7	1	1	5.4616	1.386E-03	1.729E-05	1.483176	0.99005	F-0.5PG-mT	mT blank	11941.44	Protein TANC2 (Fragment) OS=Homo sapiens OX=9606 GN=TANC2 PE=1 SV=1
A0A0A0MSM0	1	1	4.5632	3.533E-10	1.769E-11	3.578696	1	P-0.5PG-mT	P-mT-Susp	89526.08	Heat shock protein 105 kDa OS=Homo sapiens OX=9606 GN=HSPH1 PE=1 SV=1
Q05707	1	1	4.475	1.643E-10	9.836E-12	4.328996	1	P-mT-Susp	mT blank	194599.3	Collagen alpha-1(XIV) chain OS=Homo sapiens OX=9606 GN=COL14A1 PE=1 SV=3
Q9BXU3	1	1	4.8741	2.271E-08	6.322E-10	2.079766	1	P-mT-Susp	P-0.5PG-mT	45924.91	Testis-expressed protein 13A OS=Homo sapiens OX=9606 GN=TEX13A PE=1 SV=1
I3L311	1	1	6.2879	4.610E-12	7.058E-13	6.576169	1	mT blank	F-mT-2D	7429.853	Myb-binding protein 1A (Fragment) OS=Homo sapiens OX=9606 GN=MYBBP1A PE=1 SV=1
A0A590UJA7;	1	1	5.6395	8.614E-09	2.732E-10	2.527523	1	P-0.5PG-mT	mT blank	19218.1	Speckle-type POZ protein OS=Homo sapiens OX=9606 GN=SPOP PE=1 SV=1
A0A2R8Y6Y9	1	1	5.5381	1.162E-09	4.889E-11	10.74946	1	P-0.5PG-mT	F-mT-2D	9424.005	Breast cancer type 1 susceptibility protein OS=Homo sapiens OX=9606 GN=BRCA1 PE=1 SV=1
H3BN49;H3BS	1	1	11.2444	3.374E-12	5.969E-13	3.379093	1	P-mT-Susp	mT blank	12167.69	Transforming growth factor beta-1-induced transcript 1 protein OS=Homo sapiens OX=9606 GN=TGFB
H0YIV4;P5520	1	1	5.2364	1.631E-11	1.755E-12	10.18271	1	P-mT-Susp	mT blank	44999.63	Nucleosome assembly protein 1-like 1 (Fragment) OS=Homo sapiens OX=9606 GN=NAP1L1 PE=1 SV=1
P30304	1	1	4.9314	9.338E-08	2.206E-09	2.255186	1	P-mT-Susp	mT blank	59771.23	M-phase inducer phosphatase 1 OS=Homo sapiens OX=9606 GN=CDC25A PE=1 SV=2
A0A286YF22;	1	1	6.08	4.319E-07	8.839E-09	2.15696	1	P-mT-Susp	mT blank	56680.14	D-3-phosphoglycerate dehydrogenase OS=Homo sapiens OX=9606 GN=PHGDH PE=1 SV=1
A0A3B3ITD8;	1	1	5.0188	4.182E-08	1.060E-09	2.917687	1	P-mT-Susp	mT blank	144018	Nuclear pore complex protein Nup98-Nup96 OS=Homo sapiens OX=9606 GN=NUP98 PE=1 SV=1
P08172	1	1	11.6442	1.720E-10	1.014E-11	9.419725	1	P-mT-Susp	P-0.5PG-mT	52456.51	Muscarinic acetylcholine receptor M2 OS=Homo sapiens OX=9606 GN=CHRM2 PE=1 SV=1
A0A087WZX6	1	1	5.1636	9.774E-08	2.282E-09	9.265631	1	P-0.5PG-mT	P-mT-Susp	30318.19	Phospholipid-transporting ATPase ABCA7 (Fragment) OS=Homo sapiens OX=9606 GN=ABCA7 PE=1 SV
Q9BRS2	1	1	5.4939	2.112E-07	4.570E-09	6.000947	1	P-0.5PG-mT	mT blank	65925.29	Serine/threonine-protein kinase RIO1 OS=Homo sapiens OX=9606 GN=RIOK1 PE=1 SV=2
D6R9P5;D6RC	1	1	5.6437	1.865E-14	1.748E-14	24.78028	1	P-mT-Susp	P-0.5PG-mT	13917.67	Prolactin receptor (Fragment) OS=Homo sapiens OX=9606 GN=PRLR PE=1 SV=1
A0A0R4J2G7;	1	1	4.4432	3.606E-11	3.120E-12	9.86696	1	P-mT-Susp	mT blank	166535.9	Neurexin-1-beta OS=Homo sapiens OX=9606 GN=NRXN1 PE=1 SV=1
Q9UNQ0	1	1	4.4893	1.321E-12	3.186E-13	14.39779	1	F-mT-2D	mT blank	72998.66	Broad substrate specificity ATP-binding cassette transporter ABCG2 OS=Homo sapiens OX=9606 GN=A
C9JH98	1	1	5.7143	1.814E-11	1.920E-12	3.31885	1	P-mT-Susp	mT blank	11893.21	Neuropilin-2 (Fragment) OS=Homo sapiens OX=9606 GN=NRP2 PE=1 SV=8
Q6Y2X3	1	1	6.1317	5.894E-04	7.692E-06	1.722191	0.99782	P-0.5PG-mT	F-mT-2D	79481.31	DnaJ homolog subfamily C member 14 OS=Homo sapiens OX=9606 GN=DNAJC14 PE=2 SV=2
A0A087X0B8;	1	1	11.8644	2.554E-15	6.776E-15	10.51402	1	P-mT-Susp	F-0.5PG-mT	8056.146	Acyl-CoA-binding domain-containing protein 5 OS=Homo sapiens OX=9606 GN=ACBD5 PE=1 SV=1
M0QZ20;M0R	1	1	5.2986	3.463E-06	6.224E-08	3.89312	1	P-0.5PG-mT	P-mT-Susp	20406.18	Calpain-12 (Fragment) OS=Homo sapiens OX=9606 GN=CAPN12 PE=3 SV=1
M0QZS6	1	1	6.8642	2.550E-06	4.613E-08	4.912598	1	mT blank	P-mT-Susp	29650.71	SUMO-activating enzyme subunit 1 OS=Homo sapiens OX=9606 GN=SAE1 PE=1 SV=1
P09067	1	1	4.1672	9.244E-02	1.004E-03	2.95316	0.53216	F-0.5PG-mT	F-mT-2D	29605.6	Homeobox protein Hox-B5 OS=Homo sapiens OX=9606 GN=HOXB5 PE=1 SV=3
A0A087WUL9	1	1	5.54	7.255E-05	1.062E-06	1.685733	0.99999	P-mT-Susp	F-0.5PG-mT	43024.55	26S proteasome non-ATPase regulatory subunit 13 OS=Homo sapiens OX=9606 GN=PSMD13 PE=1 SV
D6RA24;Q9NZ	1	1	6.0311	2.235E-09	8.637E-11	6.762911	1	mT blank	P-mT-Susp	7170.202	Placenta-specific 8_ isoform CRA_b OS=Homo sapiens OX=9606 GN=PLAC8 PE=1 SV=1
P45983	1	1	5.2959	1.032E-02	1.194E-04	2.375598	0.88613	mT blank	P-mT-Susp	48865.95	Mitogen-activated protein kinase 8 OS=Homo sapiens OX=9606 GN=MAPK8 PE=1 SV=2

E9PKT7;Q68D	1	1	11.7516	1.221E-10	7.653E-12	4.822232	1	P-mT-Susp	P-0.5PG-mT	15097.24	Schlafen family member 13 (Fragment) OS=Homo sapiens OX=9606 GN=SLFN13 PE=1 SV=8
B4DY26;P368	1	1	5.3696	4.487E-10	2.126E-11	3.992761	1	P-mT-Susp	mT blank	49673.9	Receptor protein serine/threonine kinase OS=Homo sapiens OX=9606 GN=TGFBR1 PE=1 SV=1
E7ERK2;Q025	1	1	4.9587	1.698E-10	1.009E-11	11.69054	1	P-mT-Susp	P-0.5PG-mT	30377.61	Paired box protein Pax-5 OS=Homo sapiens OX=9606 GN=PAX5 PE=1 SV=1
Q8TAQ9	1	1	4.5385	4.536E-03	5.406E-05	2.323333	0.95055	mT blank	P-mT-Susp	40788.52	SUN domain-containing protein 3 OS=Homo sapiens OX=9606 GN=SUN3 PE=1 SV=4
Q9Y4D1;G3V2	3	1	16.2252	5.806E-12	7.714E-13	3.523516	1	P-mT-Susp	F-0.5PG-mT	124044	Disheveled-associated activator of morphogenesis 1 OS=Homo sapiens OX=9606 GN=DAAM1 PE=1 SV
J3QRC2	1	1	12.0124	3.147E-07	6.593E-09	2.99123	1	P-mT-Susp	mT blank	8204.119	Rho GTPase-activating protein 28 (Fragment) OS=Homo sapiens OX=9606 GN=ARHGAP28 PE=1 SV=1
A0A087WTZ7	1	1	5.8624	2.480E-04	3.381E-06	8.147671	0.99968	P-0.5PG-mT	P-mT-Susp	22363.36	Pleckstrin homology-like domain family B member 1 (Fragment) OS=Homo sapiens OX=9606 GN=PHLD
O00590;V9GY	1	1	6.0434	9.919E-07	1.912E-08	41.65356	1	P-mT-Susp	P-0.5PG-mT	43956.18	Atypical chemokine receptor 2 OS=Homo sapiens OX=9606 GN=ACKR2 PE=1 SV=2
P51801	1	1	5.7969	1.136E-06	2.174E-08	7.591785	1	P-mT-Susp	F-0.5PG-mT	76073.45	Chloride channel protein ClC-Kb OS=Homo sapiens OX=9606 GN=CLCNKB PE=1 SV=3
M0R0A3;Q8IV	1	1	5.2264	3.061E-10	1.614E-11	4.104789	1	P-mT-Susp	mT blank	44323.5	Zinc finger protein 584 OS=Homo sapiens OX=9606 GN=ZNF584 PE=1 SV=1
A8MSM7	1	1	5.1444	5.001E-03	5.951E-05	1.693581	0.94482	F-mT-2D	mT blank	21934.74	Microtubule-associated protein 9 OS=Homo sapiens OX=9606 GN=MAP9 PE=1 SV=1
Q7Z6W7	1	1	6.0625	7.922E-04	1.017E-05	57.19401	0.99617	P-0.5PG-mT	P-mT-Susp	35604.79	DnaJ homolog subfamily B member 7 OS=Homo sapiens OX=9606 GN=DNAJB7 PE=2 SV=2
K7EJ03;Q9279	1	1	11.8894	8.808E-03	1.030E-04	1.902175	0.90155	P-mT-Susp	mT blank	9655.899	Endoplasmic reticulum protein SC65 (Fragment) OS=Homo sapiens OX=9606 GN=P3H4 PE=1 SV=1
H7C2V2;Q9P2	1	1	4.5668	4.760E-07	9.665E-09	3.587159	1	mT blank	P-mT-Susp	18452.65	Ankyrin repeat and IBR domain-containing protein 1 (Fragment) OS=Homo sapiens OX=9606 GN=ANKI
F8VQT2;F8VY	1	1	5.3232	9.012E-07	1.754E-08	11.08172	1	P-mT-Susp	mT blank	8319.967	Zinc finger CCCH domain-containing protein 10 (Fragment) OS=Homo sapiens OX=9606 GN=ZC3H10 PE
A6XGL2;P013	1	1	6.3372	8.261E-07	1.612E-08	1.927379	1	P-0.5PG-mT	P-mT-Susp	11300.96	Insulin OS=Homo sapiens OX=9606 GN=INS PE=1 SV=1
Q8NC26	1	1	5.9984	8.353E-05	1.207E-06	7.878572	0.99999	F-0.5PG-mT	F-mT-2D	48830.2	Zinc finger protein 114 OS=Homo sapiens OX=9606 GN=ZNF114 PE=1 SV=1
Q6ZU80	1	1	4.2416	6.354E-09	2.090E-10	15.60631	1	P-mT-Susp	mT blank	128642.6	Centrosomal protein of 128 kDa OS=Homo sapiens OX=9606 GN=CEP128 PE=1 SV=2
A0A7P0T847;	1	1	5.7909	1.180E-08	3.546E-10	Infinity	1	mT blank	P-mT-Susp	43353.94	A-kinase anchor protein 8-like OS=Homo sapiens OX=9606 GN=AKAP8L PE=4 SV=1
O14986	1	1	6.1693	4.476E-06	7.882E-08	1.973203	1	P-0.5PG-mT	mT blank	61264.55	Phosphatidylinositol 4-phosphate 5-kinase type-1 beta OS=Homo sapiens OX=9606 GN=PIP5K1B PE=1
K7EL32	1	1	7.7996	3.009E-07	6.320E-09	4346.232	1	P-mT-Susp	P-0.5PG-mT	24423.42	Thimet oligopeptidase (Fragment) OS=Homo sapiens OX=9606 GN=THOP1 PE=1 SV=1
A0A024R3M2	1	1	4.2758	9.891E-03	1.151E-04	1.918764	0.89042	P-mT-Susp	mT blank	81199.1	HCG39893_ isoform CRA_a OS=Homo sapiens OX=9606 GN=GRAMD1B PE=1 SV=1
E9PJF0;E9PQV	1	1	4.6204	5.857E-10	2.680E-11	7.85744	1	P-mT-Susp	mT blank	36193.53	Mitogen-activated protein kinase OS=Homo sapiens OX=9606 GN=MAPK3 PE=1 SV=1
Q7Z3V5	1	1	5.1902	3.956E-04	5.257E-06	5.611998	0.99905	P-0.5PG-mT	mT blank	73244.74	Zinc finger protein 571 OS=Homo sapiens OX=9606 GN=ZNF571 PE=2 SV=3
A0A0D9SGJ8;	1	1	5.3532	4.024E-07	8.256E-09	251.6246	1	P-mT-Susp	P-0.5PG-mT	82199.72	PWWP domain-containing DNA repair factor 3A OS=Homo sapiens OX=9606 GN=PWWP3A PE=1 SV=1
A0A2R8Y516;	1	1	5.0832	1.556E-05	2.507E-07	11.92576	1	mT blank	P-0.5PG-mT	17401.57	Autism susceptibility gene 2 protein (Fragment) OS=Homo sapiens OX=9606 GN=AUTS2 PE=1 SV=1
A6NK53	1	1	4.8557	3.536E-06	6.325E-08	3.628457	1	F-mT-2D	F-0.5PG-mT	78857.08	Zinc finger protein 233 OS=Homo sapiens OX=9606 GN=ZNF233 PE=2 SV=3
A0A140T947;	1	1	4.6637	2.425E-07	5.176E-09	1.797335	1	F-0.5PG-mT	P-mT-Susp	77275.19	Pre-mRNA-splicing factor ATP-dependent RNA helicase DHX16 (Fragment) OS=Homo sapiens OX=9606
E9PM19;P295	1	1	5.1583	2.895E-12	5.431E-13	4.278654	1	P-mT-Susp	mT blank	114617.3	Tyrosine-protein kinase OS=Homo sapiens OX=9606 GN=TYK2 PE=1 SV=1
A0A6Q8PF27;	1	1	6.8024	3.753E-08	9.701E-10	13.06336	1	P-mT-Susp	F-0.5PG-mT	91342.17	Polyphosphoinositide phosphatase OS=Homo sapiens OX=9606 GN=FIG4 PE=1 SV=1
O95238	1	1	5.9387	1.327E-07	2.984E-09	13.07398	1	P-mT-Susp	P-0.5PG-mT	37802.8	SAM pointed domain-containing Ets transcription factor OS=Homo sapiens OX=9606 GN=SPDEF PE=1
Q8N4M1	1	1	5.6884	7.719E-03	9.076E-05	2.876312	0.91325	P-mT-Susp	F-mT-2D	75152.19	Choline transporter-like protein 3 OS=Homo sapiens OX=9606 GN=SLC44A3 PE=1 SV=4
Q96MT3	1	1	5.0543	7.035E-09	2.286E-10	1001.509	1	P-0.5PG-mT	P-mT-Susp	96239.34	Prickle-like protein 1 OS=Homo sapiens OX=9606 GN=PRICKLE1 PE=1 SV=2
Q8IUC2	1	1	5.972	1.811E-04	2.507E-06	176.6085	0.99986	F-0.5PG-mT	P-0.5PG-mT	7053.731	Keratin-associated protein 8-1 OS=Homo sapiens OX=9606 GN=KRTAP8-1 PE=1 SV=1
E5RHS3;O602	1	1	4.7308	8.325E-11	5.813E-12	Infinity	1	F-0.5PG-mT	P-mT-Susp	69714.06	Suppression of tumorigenicity 18 protein (Fragment) OS=Homo sapiens OX=9606 GN=ST18 PE=1 SV=1
P46937	1	1	5.0444	2.229E-04	3.064E-06	Infinity	0.99976	P-mT-Susp	F-0.5PG-mT	54518.7	Transcriptional coactivator YAP1 OS=Homo sapiens OX=9606 GN=YAP1 PE=1 SV=2
A0A0A0MTT2	1	1	5.0197	4.889E-06	8.536E-08	27.23466	1	P-mT-Susp	mT blank	71794.24	Zinc finger protein 559 OS=Homo sapiens OX=9606 GN=ZNF559 PE=1 SV=1
P16070	1	1	5.3733	4.472E-11	3.554E-12	Infinity	1	F-mT-2D	mT blank	82050.98	CD44 antigen OS=Homo sapiens OX=9606 GN=CD44 PE=1 SV=3
M0R2Q7	1	1	6.9073	3.216E-01	3.404E-03	4.510895	0.27924	P-mT-Susp	mT blank	20549.11	Signal-induced proliferation-associated 1-like protein 3 (Fragment) OS=Homo sapiens OX=9606 GN=SI
V9GYG1	1	1	5.7496	5.757E-08	1.397E-09	Infinity	1	mT blank	P-mT-Susp	10391.07	Histone-lysine N-methyltransferase SETD7 (Fragment) OS=Homo sapiens OX=9606 GN=SETD7 PE=1 SV
A0A0J9YX90	1	1	4.9599	2.572E-02	2.891E-04	Infinity	0.76776	F-0.5PG-mT	P-mT-Susp	31662.21	Glucose-6-phosphate isomerase (Fragment) OS=Homo sapiens OX=9606 GN=GPI PE=1 SV=1
O60825	1	1	6.4902	1.951E-01	2.081E-03	3.212476	0.37902	mT blank	P-0.5PG-mT	58990.14	6-phosphofructo-2-kinase/fructose-2_6-bisphosphatase 2 OS=Homo sapiens OX=9606 GN=PFKFB2 PE
A0A1B0GUB2;	1	1	4.8492	1.052E-02	1.215E-04	8.493974	0.8842	F-mT-2D	P-mT-Susp	51568.39	Parafibromin OS=Homo sapiens OX=9606 GN=CDC73 PE=1 SV=1
H7C1J8	1	1	5.6945	2.409E-01	2.565E-03	7.088612	0.33629	mT blank	P-mT-Susp	11354.88	Heterogeneous nuclear ribonucleoprotein A3 (Fragment) OS=Homo sapiens OX=9606 GN=HNRNPA3 P
A0A0B4J260	1	1	5.5237	4.128E-02	4.596E-04	3.385085	0.68801	P-mT-Susp	P-0.5PG-mT	6198.911	PRKC apoptosis WT1 regulator protein (Fragment) OS=Homo sapiens OX=9606 GN=PAWR PE=1 SV=5
F2Z3N3;Q68B	1	1	5.1246	1.022E-07	2.372E-09	Infinity	1	mT blank	P-mT-Susp	84811.48	Olfactomedin-like protein 2B OS=Homo sapiens OX=9606 GN=OLFML2B PE=1 SV=1
A0A0G2JK11;	1	1	5.2003	7.604E-01	8.007E-03	Infinity	0.11923	F-0.5PG-mT	P-mT-Susp	33222.47	MHC class I polypeptide-related sequence A OS=Homo sapiens OX=9606 GN=MICA PE=1 SV=1
B1ANH2	1	1	5.5113	3.041E-03	3.668E-05	3.772485	0.9695	P-mT-Susp	F-mT-2D	27806.16	Guanylate kinase (Fragment) OS=Homo sapiens OX=9606 GN=GUK1 PE=1 SV=1
A0A7I2V3U5;	1	1	6.0073	1.134E-10	7.282E-12	Infinity	1	F-mT-2D	F-0.5PG-mT	44993.05	Glycylpeptide N-tetradecanoyltransferase OS=Homo sapiens OX=9606 GN=NMT1 PE=1 SV=1
P07686;Q5UR	1	1	4.8553	1.018E-05	1.678E-07	154.0049	1	P-0.5PG-mT	P-mT-Susp	63567.64	Beta-hexosaminidase subunit beta OS=Homo sapiens OX=9606 GN=HEXB PE=1 SV=3

A0A0A0MT39	1	1	4.8916	1.535E-04	2.159E-06	2.109344	0.99991	F-0.5PG-mT	F-mT-2D	223639.6	Sodium channel protein OS=Homo sapiens OX=9606 GN=SCN5A PE=1 SV=1
C9J3R7;C9J48	1	1	5.342	1.830E-05	2.907E-07	12.01888	1	F-mT-2D	F-0.5PG-mT	6888.897	Zinc finger protein 789 OS=Homo sapiens OX=9606 GN=ZNF789 PE=4 SV=1
A0A669KB17;	1	1	5.3211	7.837E-04	1.008E-05	187.996	0.99625	F-0.5PG-mT	P-mT-Susp	32521.54	Signal transducer and activator of transcription 1-alpha/beta OS=Homo sapiens OX=9606 GN=STAT1 P
J3KTM9;Q149	1	1	4.849	2.976E-03	3.595E-05	4.152831	0.97033	F-0.5PG-mT	P-mT-Susp	77648.34	Importin subunit beta-1 (Fragment) OS=Homo sapiens OX=9606 GN=KPNB1 PE=1 SV=1
E9PIZ2	1	1	4.6039	6.430E-04	8.350E-06	7.346681	0.99742	P-mT-Susp	F-0.5PG-mT	79270.86	LARGE xylosyl- and glucuronyltransferase 2 OS=Homo sapiens OX=9606 GN=LARGE2 PE=1 SV=1
A0A087WVE9	1	1	4.7969	8.750E-09	2.753E-10	Infinity	1	F-0.5PG-mT	F-mT-2D	79638.2	Aryl hydrocarbon receptor nuclear translocator 2 OS=Homo sapiens OX=9606 GN=ARNT2 PE=1 SV=1
E7END6	1	1	4.169	1.271E-02	1.463E-04	6.078999	0.86353	P-mT-Susp	F-0.5PG-mT	56815.78	Vitamin K-dependent protein C OS=Homo sapiens OX=9606 GN=PROC PE=1 SV=1
H7BYF0	1	1	5.5256	2.646E-05	4.091E-07	Infinity	1	P-0.5PG-mT	mT blank	11352.37	Leucine carboxyl methyltransferase 1 OS=Homo sapiens OX=9606 GN=LCMT1 PE=1 SV=1
A0A7I2V5M7;	1	1	5.2044	5.030E-09	1.689E-10	24.49225	1	P-mT-Susp	mT blank	53386.78	Ras GTPase-activating protein-binding protein 1 OS=Homo sapiens OX=9606 GN=G3BP1 PE=1 SV=1
C9JYY6;Q9282	1	1	4.3584	3.854E-07	7.969E-09	9.356371	1	F-mT-2D	P-mT-Susp	134669.2	Neuronal cell adhesion molecule OS=Homo sapiens OX=9606 GN=NRCAM PE=1 SV=3
I3L1N0;Q9BU	1	1	5.7771	1.716E-06	3.192E-08	17.53895	1	P-mT-Susp	P-0.5PG-mT	30073.67	PHD finger protein 23 OS=Homo sapiens OX=9606 GN=PHF23 PE=1 SV=1
Q12872	1	1	4.8031	1.754E-14	1.748E-14	18.03621	1	P-mT-Susp	P-0.5PG-mT	105220.8	Splicing factor_ suppressor of white-apricot homolog OS=Homo sapiens OX=9606 GN=SFSWAP PE=1 S
Q9H0E9	1	1	4.0712	1.239E-06	2.354E-08	27.36017	1	mT blank	F-mT-2D	136134.5	Bromodomain-containing protein 8 OS=Homo sapiens OX=9606 GN=BRD8 PE=1 SV=2
C9J6P7;E9PH7	1	1	5.0711	3.421E-05	5.198E-07	3.342576	1	P-mT-Susp	F-mT-2D	20145.92	Nuclear valosin-containing protein-like (Fragment) OS=Homo sapiens OX=9606 GN=NVL PE=1 SV=1
Q9GZU1	1	1	5.4959	2.943E-10	1.573E-11	7.942213	1	P-0.5PG-mT	F-0.5PG-mT	66162.92	Mucolipin-1 OS=Homo sapiens OX=9606 GN=MCOLN1 PE=1 SV=1
P55287	1	1	4.6821	8.182E-08	1.956E-09	10.50172	1	F-mT-2D	P-mT-Susp	88421.65	Cadherin-11 OS=Homo sapiens OX=9606 GN=CDH11 PE=2 SV=2
K7EPR9	1	1	5.6138	2.443E-04	3.336E-06	2.353428	0.99969	P-mT-Susp	mT blank	3342.103	Apolipoprotein C-I OS=Homo sapiens OX=9606 GN=APOC1 PE=4 SV=1
Q9NRR3	1	1	5.5562	1.651E-02	1.886E-04	1.777633	0.83145	P-mT-Susp	F-0.5PG-mT	9394.614	CDC42 small effector protein 2 OS=Homo sapiens OX=9606 GN=CDC42SE2 PE=1 SV=1
Q6ZT52	1	1	5.3073	1.256E-05	2.049E-07	4.392747	1	P-mT-Susp	mT blank	37289.43	Protein FAM43B OS=Homo sapiens OX=9606 GN=FAM43B PE=2 SV=1
H3BRB6;Q8N	1	1	4.4114	5.035E-07	1.017E-08	39.9505	1	mT blank	P-mT-Susp	22163.67	E3 ubiquitin-protein ligase ZNRF1 OS=Homo sapiens OX=9606 GN=ZNRF1 PE=1 SV=1
O75052	1	1	9.3634	8.112E-05	1.174E-06	2.292252	0.99999	F-0.5PG-mT	P-mT-Susp	56492.02	Carboxyl-terminal PDZ ligand of neuronal nitric oxide synthase protein OS=Homo sapiens OX=9606 GN
H0YMW5	1	1	5.0103	9.397E-09	2.911E-10	2.970569	1	P-mT-Susp	mT blank	14224.74	Proprotein convertase subtilisin/kexin type 6 (Fragment) OS=Homo sapiens OX=9606 GN=PCSK6 PE=1
A0A0B4J210;	1	1	5.3247	1.768E-10	1.027E-11	8.789312	1	F-mT-2D	F-0.5PG-mT	90993.69	La-related protein 1 (Fragment) OS=Homo sapiens OX=9606 GN=LARP1 PE=1 SV=5
A0A494BZX2	1	1	5.8595	1.028E-10	6.763E-12	7.392984	1	P-mT-Susp	P-0.5PG-mT	12922.67	Protein kinase C delta type (Fragment) OS=Homo sapiens OX=9606 GN=PRKCD PE=1 SV=1
A0A7I2V3F8	1	1	5.0365	2.778E-08	7.523E-10	3.320443	1	P-mT-Susp	mT blank	21614.15	DNA (cytosine-5)-methyltransferase 1 OS=Homo sapiens OX=9606 GN=DNMT1 PE=1 SV=1
A0A494C0P1;	1	1	4.9	1.550E-13	6.498E-14	22.75082	1	P-mT-Susp	P-0.5PG-mT	83498.1	Glucocorticoid receptor OS=Homo sapiens OX=9606 GN=NR3C1 PE=1 SV=1
A0A087X043;	1	1	7.352	1.144E-07	2.633E-09	3.832467	1	F-mT-2D	mT blank	105007.5	Glutamate receptor OS=Homo sapiens OX=9606 GN=GRID2 PE=1 SV=1
Q9NQC3	1	1	4.7267	8.338E-13	2.141E-13	15.9863	1	P-mT-Susp	mT blank	130330.8	Reticulon-4 OS=Homo sapiens OX=9606 GN=RTN4 PE=1 SV=2
A0A0R4J2E4;	1	1	12.2646	5.808E-06	1.007E-07	2.749013	1	P-mT-Susp	mT blank	121276.3	Fas-binding factor 1 (Fragment) OS=Homo sapiens OX=9606 GN=FBF1 PE=1 SV=2
Q9H6J7	1	1	4.7792	1.113E-04	1.579E-06	3.791319	0.99996	F-0.5PG-mT	P-mT-Susp	37923.79	UPF0705 protein C11orf49 OS=Homo sapiens OX=9606 GN=C11orf49 PE=1 SV=2
K7EIJ2;K7EIU8	1	1	5.5751	9.036E-05	1.301E-06	1.33521	0.99998	P-0.5PG-mT	F-mT-2D	16244.47	Mothers against decapentaplegic homolog 4 (Fragment) OS=Homo sapiens OX=9606 GN=SMAD4 PE=1
Q99581	1	1	6.2017	1.720E-03	2.126E-05	44.79794	0.98614	F-0.5PG-mT	P-mT-Susp	25144.59	Protein FEV OS=Homo sapiens OX=9606 GN=FEV PE=1 SV=1
P51617	1	1	9.1562	4.978E-08	1.238E-09	3.922923	1	P-mT-Susp	F-mT-2D	77506.24	Interleukin-1 receptor-associated kinase 1 OS=Homo sapiens OX=9606 GN=IRAK1 PE=1 SV=2
Q4KMG0	1	1	5.009	9.940E-03	1.155E-04	1.888854	0.88992	P-mT-Susp	mT blank	140743.9	Cell adhesion molecule-related/down-regulated by oncogenes OS=Homo sapiens OX=9606 GN=CDON
P04746;P0DT	1	1	11.3818	4.516E-04	5.962E-06	3.887322	0.99874	F-mT-2D	P-mT-Susp	58391.27	Pancreatic alpha-amylase OS=Homo sapiens OX=9606 GN=AMY2A PE=1 SV=2
A0A087X254;	1	1	5.3551	3.730E-02	4.164E-04	1.884948	0.70601	F-0.5PG-mT	mT blank	87082.99	Zinc finger protein 615 OS=Homo sapiens OX=9606 GN=ZNF615 PE=1 SV=1
A0A0D9SFB9;	1	1	11.1942	8.883E-03	1.037E-04	4.502833	0.90076	P-mT-Susp	F-0.5PG-mT	19227.7	Maternal embryonic leucine zipper kinase OS=Homo sapiens OX=9606 GN=MELK PE=1 SV=1
E9PK59;E9PP	1	1	5.0778	1.461E-10	8.879E-12	6.015307	1	P-mT-Susp	mT blank	86810.79	N-terminal kinase-like protein OS=Homo sapiens OX=9606 GN=SCYL1 PE=1 SV=1
A0A3B3IS70	1	1	4.991	1.282E-01	1.375E-03	1.865502	0.46517	mT blank	F-mT-2D	30195.66	Inositol 1_4_5-trisphosphate receptor type 1 (Fragment) OS=Homo sapiens OX=9606 GN=ITPR1 PE=1
H0YGI8	1	1	6.1293	3.433E-08	8.931E-10	2.817621	1	F-mT-2D	mT blank	16298.53	Stress-induced-phosphoprotein 1 (Fragment) OS=Homo sapiens OX=9606 GN=STIP1 PE=1 SV=1
F8VY42	1	1	5.5632	9.310E-05	1.335E-06	10.70552	0.99998	P-0.5PG-mT	F-mT-2D	10575.14	Ceramide synthase 5 OS=Homo sapiens OX=9606 GN=CERS5 PE=1 SV=1
A2RRD8;M0Q	1	1	5.4436	9.771E-08	2.282E-09	2.136564	1	P-mT-Susp	mT blank	61151.35	Zinc finger protein 320 OS=Homo sapiens OX=9606 GN=ZNF320 PE=1 SV=1
A0A087WWN	1	1	5.9575	2.764E-06	4.989E-08	3.451603	1	P-mT-Susp	F-0.5PG-mT	20030.9	Cullin-4A OS=Homo sapiens OX=9606 GN=CUL4A PE=1 SV=1
F5GYG7	1	1	5.0389	9.718E-08	2.282E-09	9.879343	1	F-mT-2D	mT blank	9160.554	Alpha-2-macroglobulin-like protein 1 (Fragment) OS=Homo sapiens OX=9606 GN=A2ML1 PE=1 SV=1
P41227	1	1	5.0411	3.417E-05	5.198E-07	399.4092	1	P-0.5PG-mT	P-mT-Susp	26629.65	N-alpha-acetyltransferase 10 OS=Homo sapiens OX=9606 GN=NAA10 PE=1 SV=1
A0A087X0R9;	1	1	5.171	4.068E-09	1.402E-10	3.508063	1	mT blank	P-mT-Susp	63799.74	Neutral and basic amino acid transport protein rBAT OS=Homo sapiens OX=9606 GN=SLC3A1 PE=1 SV=
K7ERR8;Q96N	1	1	4.8191	2.632E-05	4.076E-07	12.60216	1	F-0.5PG-mT	P-mT-Susp	15383.11	Zinc finger protein 396 (Fragment) OS=Homo sapiens OX=9606 GN=ZNF396 PE=1 SV=1
H3BND4;Q6P	1	1	4.385	1.909E-05	3.021E-07	91.84344	1	mT blank	P-mT-Susp	89788.88	Pyridoxal-dependent decarboxylase domain-containing protein 1 OS=Homo sapiens OX=9606 GN=PDX
Q5JNZ3	1	1	4.4383	1.094E-07	2.524E-09	3.306238	1	P-mT-Susp	mT blank	78545.69	Zinc finger protein 311 OS=Homo sapiens OX=9606 GN=ZNF311 PE=2 SV=2
C9JUF0;E7EM	1	1	5.7603	5.494E-05	8.221E-07	110.3776	1	F-mT-2D	P-mT-Susp	6325.851	Eukaryotic initiation factor 4A-II (Fragment) OS=Homo sapiens OX=9606 GN=EIF4A2 PE=1 SV=1

P51795	1	1	4.4128	1.214E-05	1.984E-07	2.26491	1	P-mT-Susp	mT blank	91754.85	H(+)/Cl(-) exchange transporter 5 OS=Homo sapiens OX=9606 GN=CLCN5 PE=1 SV=2
Q9UHJ3	1	1	4.42	2.280E-10	1.287E-11	8.086814	1	F-mT-2D	mT blank	99738.36	Scm-like with four MBT domains protein 1 OS=Homo sapiens OX=9606 GN=SFMBT1 PE=1 SV=2
O60500	1	1	4.8467	3.618E-04	4.832E-06	7.537257	0.99922	P-mT-Susp	P-0.5PG-mT	135882.9	Nephrin OS=Homo sapiens OX=9606 GN=NPHS1 PE=1 SV=1
Q5STZ8;Q8NE	1	1	4.1743	6.806E-07	1.341E-08	3.9259	1	mT blank	P-mT-Susp	96510.23	ATP-binding cassette sub-family F member 1 OS=Homo sapiens OX=9606 GN=ABCF1 PE=1 SV=10
A0A087X1U1;	1	1	5.4795	1.217E-03	1.528E-05	113.5274	0.99193	mT blank	P-mT-Susp	83658.63	Arf-GAP with GTPase_ ANK repeat and PH domain-containing protein 1 OS=Homo sapiens OX=9606 G
E9PJU8	1	1	5.7379	4.928E-04	6.462E-06	2.423007	0.99849	P-mT-Susp	mT blank	21458.31	Ester hydrolase C11orf54 (Fragment) OS=Homo sapiens OX=9606 GN=C11orf54 PE=1 SV=1
B0QYA5;B0QY	1	1	5.2355	7.617E-05	1.107E-06	8.946289	0.99999	mT blank	P-mT-Susp	31957.2	Eukaryotic translation initiation factor 3 subunit D (Fragment) OS=Homo sapiens OX=9606 GN=EIF3D
Q969H0	1	1	4.4902	4.917E-02	5.451E-04	1.525291	0.65598	P-mT-Susp	P-0.5PG-mT	80632.55	F-box/WD repeat-containing protein 7 OS=Homo sapiens OX=9606 GN=FBXW7 PE=1 SV=1
Q6IMN6	1	1	4.2138	1.046E-04	1.490E-06	128.9892	0.99997	P-0.5PG-mT	P-mT-Susp	126552.3	Caprin-2 OS=Homo sapiens OX=9606 GN=CAPRIN2 PE=1 SV=1
H7C4F3	1	1	5.312	1.445E-08	4.229E-10	7.061301	1	P-0.5PG-mT	P-mT-Susp	13565.28	Inactive histone-lysine N-methyltransferase 2E (Fragment) OS=Homo sapiens OX=9606 GN=KMT2E PE
K7EL58;Q86Y	1	1	5.2069	1.709E-07	3.780E-09	2.830988	1	F-0.5PG-mT	mT blank	14639.29	Rab11 family-interacting protein 4 (Fragment) OS=Homo sapiens OX=9606 GN=RAB11FIP4 PE=1 SV=1
A6NHC0	1	1	5.3288	6.684E-06	1.144E-07	400.4926	1	mT blank	P-mT-Susp	80171.06	Calpain-8 OS=Homo sapiens OX=9606 GN=CAPN8 PE=1 SV=3
E9PJC9	1	1	5.6137	6.329E-02	6.950E-04	1.960117	0.60773	P-0.5PG-mT	F-mT-2D	18389.86	Inactive pancreatic lipase-related protein 1 (Fragment) OS=Homo sapiens OX=9606 GN=PNLIPRP1 PE=
Q86YJ7	1	1	5.4052	2.313E-05	3.628E-07	2.784847	1	P-mT-Susp	mT blank	70661.7	Ankyrin repeat domain-containing protein 13B OS=Homo sapiens OX=9606 GN=ANKRD13B PE=1 SV=4
C9JP21;Q0182	1	1	4.6198	1.482E-03	1.841E-05	4.210702	0.98895	mT blank	P-mT-Susp	25004.55	DNA-binding protein SATB1 (Fragment) OS=Homo sapiens OX=9606 GN=SATB1 PE=1 SV=1
Q8IZ26	1	1	4.7052	1.390E-02	1.597E-04	1.659444	0.85304	P-0.5PG-mT	F-mT-2D	65578.24	Zinc finger protein 34 OS=Homo sapiens OX=9606 GN=ZNF34 PE=1 SV=3
O94875	1	1	5.0226	1.341E-08	3.970E-10	3.291275	1	F-mT-2D	F-0.5PG-mT	125248.2	Sorbin and SH3 domain-containing protein 2 OS=Homo sapiens OX=9606 GN=SORBS2 PE=1 SV=3
G3V4I0;O7535	1	1	4.6538	2.761E-08	7.501E-10	3.233444	1	P-0.5PG-mT	mT blank	45735.04	Ectonucleoside triphosphate diphosphohydrolase 5 OS=Homo sapiens OX=9606 GN=ENTPD5 PE=1 SV=
A0A7P0T8A6;	1	1	4.9772	1.498E-12	3.507E-13	17.95118	1	P-mT-Susp	mT blank	80491.59	MICAL-like protein 1 OS=Homo sapiens OX=9606 GN=MICALL1 PE=4 SV=1
U3KPZ6	1	1	19.5955	2.024E-11	2.048E-12	8.103277	1	P-mT-Susp	P-0.5PG-mT	4993.244	E3 ubiquitin-protein ligase RNF220 OS=Homo sapiens OX=9606 GN=RNF220 PE=4 SV=1
B4DJM9	1	1	9.4436	7.631E-09	2.459E-10	1.940873	1	P-mT-Susp	mT blank	26712.95	Serine--tRNA ligase_ mitochondrial OS=Homo sapiens OX=9606 GN=SARS2 PE=1 SV=1
P16284;A0A0	3	1	31.9964	3.954E-03	4.726E-05	2.124457	0.95786	P-0.5PG-mT	F-mT-2D	83434.77	Platelet endothelial cell adhesion molecule OS=Homo sapiens OX=9606 GN=PECAM1 PE=1 SV=2
A0A2R8Y549;	3	1	26.4168	5.360E-07	1.078E-08	2.765399	1	P-mT-Susp	mT blank	205496.3	InaD-like protein OS=Homo sapiens OX=9606 GN=PATJ PE=1 SV=1
A0A1X7SBR3;	3	1	31.6652	3.508E-03	4.206E-05	3.897773	0.96352	P-0.5PG-mT	P-mT-Susp	54301.36	Glial fibrillary acidic protein OS=Homo sapiens OX=9606 GN=GFAP PE=1 SV=1
A0A087X0K9;	2	1	9.9879	3.422E-03	4.109E-05	12.17321	0.96462	P-0.5PG-mT	F-mT-2D	197801.5	Tight junction protein 1 (Zona occludens 1)_ isoform CRA_a OS=Homo sapiens OX=9606 GN=TJP1 PE=
Q02156;C9JR2	2	1	17.9672	2.188E-05	3.456E-07	4.745537	1	P-0.5PG-mT	P-mT-Susp	85042.6	Protein kinase C epsilon type OS=Homo sapiens OX=9606 GN=PRKCE PE=1 SV=1
Q12901;K7EL	2	1	22.4444	1.422E-09	5.867E-11	4.01127	1	P-mT-Susp	mT blank	63963.27	Zinc finger protein 155 OS=Homo sapiens OX=9606 GN=ZNF155 PE=1 SV=4
Q9P2R3;I3L1Z	2	1	10.9497	2.254E-03	2.756E-05	2.528927	0.97954	P-mT-Susp	mT blank	129996.2	Rabankyrin-5 OS=Homo sapiens OX=9606 GN=ANKFY1 PE=1 SV=2
Q8NEV1	3	1	17.4754	1.007E-06	1.937E-08	1.489815	1	P-0.5PG-mT	mT blank	45333.86	Casein kinase II subunit alpha 3 OS=Homo sapiens OX=9606 GN=CSNK2A3 PE=1 SV=2
A0A7I2V360;A	1	1	5.6641	1.474E-08	4.299E-10	Infinity	1	P-0.5PG-mT	P-mT-Susp	11701.83	Heterogeneous nuclear ribonucleoprotein A1 (Fragment) OS=Homo sapiens OX=9606 GN=HNRNPA1 P
Q6YHU6	3	1	25.5509	4.831E-12	7.257E-13	5.686083	1	F-mT-2D	mT blank	223029.3	Thyroid adenoma-associated protein OS=Homo sapiens OX=9606 GN=THADA PE=1 SV=1
A0A6I8PRE0;A	3	1	15.3452	4.712E-04	6.200E-06	Infinity	0.99862	P-0.5PG-mT	mT blank	56724.66	NEDD4-binding protein 2-like 2 OS=Homo sapiens OX=9606 GN=N4BP2L2 PE=1 SV=1
Q96PV7;H0Y9	2	1	17.4776	3.995E-11	3.348E-12	6.509887	1	P-mT-Susp	P-0.5PG-mT	98025.4	Protein FAM193B OS=Homo sapiens OX=9606 GN=FAM193B PE=1 SV=3
F2Z2W7;Q8IZ	2	1	13.7169	7.627E-02	8.317E-04	2.628204	0.57091	F-mT-2D	F-0.5PG-mT	71861.58	tRNA (uracil-5-)-methyltransferase homolog A OS=Homo sapiens OX=9606 GN=TRMT2A PE=1 SV=1
A0A2R8YDQ9;	2	1	12.9349	4.246E-02	4.721E-04	8.574967	0.68294	F-mT-2D	P-mT-Susp	44184.19	Succinate--CoA ligase [ADP-forming] subunit beta_ mitochondrial OS=Homo sapiens OX=9606 GN=SU
Q8N2N9;A0A	3	1	19.578	5.994E-10	2.726E-11	2.075467	1	P-mT-Susp	F-mT-2D	155122.4	Ankyrin repeat domain-containing protein 36B OS=Homo sapiens OX=9606 GN=ANKRD36B PE=1 SV=4
Q96JN2;C9JE1	2	1	10.8329	9.586E-12	1.106E-12	79.4382	1	F-mT-2D	P-mT-Susp	135699.3	Coiled-coil domain-containing protein 136 OS=Homo sapiens OX=9606 GN=CCDC136 PE=1 SV=3
A0A5F9ZH29;	3	1	17.4981	6.318E-12	7.858E-13	10.00795	1	P-mT-Susp	mT blank	44133.64	Cell division control protein 45 homolog OS=Homo sapiens OX=9606 GN=CDC45 PE=1 SV=1
O75821	2	1	9.2684	9.318E-10	4.053E-11	5.983869	1	P-mT-Susp	mT blank	35896.2	Eukaryotic translation initiation factor 3 subunit G OS=Homo sapiens OX=9606 GN=EIF3G PE=1 SV=2
Q8NDW4;A0A	3	1	20.9428	6.504E-06	1.118E-07	3.325623	1	P-mT-Susp	mT blank	68911.55	Zinc finger protein 248 OS=Homo sapiens OX=9606 GN=ZNF248 PE=1 SV=1
A0A1B0GTW1	2	1	9.2464	1.577E-05	2.536E-07	4.583066	1	P-mT-Susp	F-0.5PG-mT	141126.2	Tight junction protein ZO-2 OS=Homo sapiens OX=9606 GN=TJP2 PE=1 SV=1
B4DQX9	2	1	17.4303	6.806E-05	1.003E-06	4.810075	0.99999	P-mT-Susp	mT blank	14067.7	Microtubule-actin cross-linking factor 1_ isoforms 1/2/3/5 OS=Homo sapiens OX=9606 GN=MACF1 PE
Q9UBC5;C9JU	2	1	15.5331	3.750E-04	5.001E-06	1.511058	0.99916	mT blank	F-mT-2D	119313.2	Unconventional myosin-Ia OS=Homo sapiens OX=9606 GN=MYO1A PE=1 SV=1
Q58FF8	2	1	15.6829	8.056E-07	1.576E-08	170.8564	1	P-0.5PG-mT	P-mT-Susp	44520.09	Putative heat shock protein HSP 90-beta 2 OS=Homo sapiens OX=9606 GN=HSP90AB2P PE=1 SV=2
G3V5X9;Q9UN	3	1	20.7485	1.395E-03	1.737E-05	56.25748	0.98996	mT blank	P-mT-Susp	25925.21	Sorting nexin-6 (Fragment) OS=Homo sapiens OX=9606 GN=SNX6 PE=1 SV=2
O43861	2	1	8.0108	6.787E-08	1.632E-09	2.970434	1	P-0.5PG-mT	F-mT-2D	130843.5	Probable phospholipid-transporting ATPase IIB OS=Homo sapiens OX=9606 GN=ATP9B PE=2 SV=4
Q05513;E9PB	2	1	11.3187	4.984E-02	5.518E-04	2.258671	0.65345	P-0.5PG-mT	mT blank	68572.52	Protein kinase C zeta type OS=Homo sapiens OX=9606 GN=PRKCZ PE=1 SV=4
E7EU81	2	1	10.6295	1.322E-09	5.511E-11	2.647957	1	P-mT-Susp	mT blank	188742	Golgin subfamily B member 1 (Fragment) OS=Homo sapiens OX=9606 GN=GOLGB1 PE=1 SV=1
H7C4Q8	2	1	10.8048	1.053E-01	1.141E-03	5.064054	0.50554	P-0.5PG-mT	F-mT-2D	30937.4	General transcription factor II-I repeat domain-containing protein 1 (Fragment) OS=Homo sapiens OX=
K7EPJ0	2	1	9.5377	7.620E-04	9.815E-06	75.56867	0.99644	F-0.5PG-mT	P-mT-Susp	26087.48	Cysteine protease OS=Homo sapiens OX=9606 GN=ATG4D PE=1 SV=1

A0A7I2V2J0;A	2	1	9.249	3.614E-03	4.326E-05	50.07389	0.96218	F-0.5PG-mT	P-mT-Susp	131723	Laminin subunit beta-1 OS=Homo sapiens OX=9606 GN=LAMB1 PE=1 SV=1
A0A7P0TA23;	2	1	10.3214	7.252E-10	3.243E-11	2.618531	1	P-mT-Susp	mT blank	65720.33	O-phosphoseryl-tRNA(Sec) selenium transferase OS=Homo sapiens OX=9606 GN=SEPSECS PE=4 SV=1
F6SRV7;F8WB	2	1	9.347	5.311E-07	1.070E-08	2.050926	1	P-mT-Susp	F-mT-2D	81941.93	[Histone H3]-dimethyl-L-lysine(36) demethylase OS=Homo sapiens OX=9606 GN=KDM2B PE=1 SV=1
F5GZ28;P1885	1	1	11.5942	2.069E-10	1.185E-11	3.580665	1	P-mT-Susp	mT blank	94575.02	DNA ligase OS=Homo sapiens OX=9606 GN=LIG1 PE=1 SV=1
F8VRS8;F8W1	2	1	10.4722	4.558E-11	3.557E-12	4.656235	1	P-mT-Susp	mT blank	29982.02	CCR4-NOT transcription complex subunit 2 (Fragment) OS=Homo sapiens OX=9606 GN=CNOT2 PE=1 S
C9JDA4;Q9NR	2	1	14.3319	1.496E-07	3.355E-09	4.912565	1	mT blank	P-mT-Susp	31684.73	Serine/threonine-protein kinase 36 (Fragment) OS=Homo sapiens OX=9606 GN=STK36 PE=1 SV=8
H0YMW2	2	1	11.3168	8.490E-08	2.017E-09	2.588069	1	P-mT-Susp	mT blank	188360.7	A-kinase anchor protein 13 (Fragment) OS=Homo sapiens OX=9606 GN=AKAP13 PE=1 SV=1
J3KMZ8;Q927	2	1	16.0061	4.509E-11	3.554E-12	Infinity	1	F-mT-2D	P-mT-Susp	46920.91	Zinc finger protein ubi-d4 OS=Homo sapiens OX=9606 GN=DPF2 PE=1 SV=1
A0A087WT21	2	1	8.4183	1.936E-11	2.002E-12	9.898209	1	P-mT-Susp	F-0.5PG-mT	160879.8	Cat eye syndrome critical region protein 2 OS=Homo sapiens OX=9606 GN=CECR2 PE=1 SV=1
Q15811;F8W7	2	1	11.9168	3.401E-04	4.564E-06	4.394476	0.99932	F-0.5PG-mT	F-mT-2D	196277.4	Intersectin-1 OS=Homo sapiens OX=9606 GN=ITSN1 PE=1 SV=3
A0A3F2YNW7	2	1	9.7001	3.940E-04	5.245E-06	92.75368	0.99906	P-mT-Susp	mT blank	246565	AT-rich interactive domain-containing protein 1B OS=Homo sapiens OX=9606 GN=ARID1B PE=1 SV=1
A0A1B0GVR6;	2	1	15.1228	3.955E-12	6.484E-13	Infinity	1	mT blank	P-mT-Susp	73135.3	Transcription factor 4 OS=Homo sapiens OX=9606 GN=TCF4 PE=1 SV=2
H3BPJ7;A0A0	2	1	16.0252	3.643E-09	1.300E-10	340.1143	1	P-mT-Susp	P-0.5PG-mT	68711.24	Transcription factor 4 OS=Homo sapiens OX=9606 GN=TCF4 PE=1 SV=1
D6RIY9	2	1	16.1091	1.385E-11	1.510E-12	5.562319	1	P-mT-Susp	mT blank	12096.31	Ankyrin-2 (Fragment) OS=Homo sapiens OX=9606 GN=ANK2 PE=1 SV=1
H7C1N2;J3KR	3	1	15.6993	2.826E-05	4.343E-07	1.698622	1	P-mT-Susp	mT blank	119893.1	Protein cordon-bleu (Fragment) OS=Homo sapiens OX=9606 GN=COBL PE=1 SV=1
Q13464	2	1	9.2657	1.272E-05	2.070E-07	1.749927	1	P-0.5PG-mT	mT blank	159201.5	Rho-associated protein kinase 1 OS=Homo sapiens OX=9606 GN=ROCK1 PE=1 SV=1
F8VNV8;P542	2	1	11.5551	3.684E-13	1.222E-13	4.559459	1	P-0.5PG-mT	mT blank	51841.69	Calcium channel voltage-dependent subunit beta 3 OS=Homo sapiens OX=9606 GN=CACNB3 PE=1 SV=
E7ENC7	2	1	15.6302	6.299E-06	1.085E-07	2.048496	1	P-mT-Susp	P-0.5PG-mT	4127.974	Negative elongation factor E OS=Homo sapiens OX=9606 GN=NELFE PE=1 SV=1
H3BPW9;Q96	3	1	19.5547	9.949E-13	2.475E-13	2.320839	1	P-0.5PG-mT	P-mT-Susp	28269.09	Enhancer of mRNA-decapping protein 3 (Fragment) OS=Homo sapiens OX=9606 GN=EDC3 PE=1 SV=1
A0A087X0M8	2	1	8.7879	4.892E-04	6.426E-06	2.762564	0.99851	F-0.5PG-mT	mT blank	131878.3	Neural cell adhesion molecule L1-like protein OS=Homo sapiens OX=9606 GN=CHL1 PE=1 SV=1
O75808;A0A1	2	1	10.8168	4.516E-01	4.774E-03	Infinity	0.21458	mT blank	F-0.5PG-mT	119937.5	Calpain-15 OS=Homo sapiens OX=9606 GN=CAPN15 PE=1 SV=1
H3BSE5	2	1	23.306	1.829E-09	7.242E-11	4.113566	1	P-mT-Susp	mT blank	18811.23	Alpha-mannosidase 2C1 (Fragment) OS=Homo sapiens OX=9606 GN=MAN2C1 PE=1 SV=2
E9PL66	2	1	18.0717	8.255E-12	9.956E-13	2.831263	1	P-mT-Susp	mT blank	17637.26	Syntabulin (Fragment) OS=Homo sapiens OX=9606 GN=SYBU PE=1 SV=1
H0YJN0;Q9UI	2	1	14.2458	5.060E-08	1.253E-09	12.93177	1	F-0.5PG-mT	F-mT-2D	22056.61	Ena/VASP-like protein (Fragment) OS=Homo sapiens OX=9606 GN=EVL PE=1 SV=1
A0A1B0GVI3;	3	1	33.3316	3.577E-13	1.222E-13	5.231595	1	F-mT-2D	mT blank	63574.34	Keratin_ type I cytoskeletal 10 OS=Homo sapiens OX=9606 GN=KRT10 PE=1 SV=2
A0A0C4DFL8;	2	1	9.2777	4.909E-07	9.944E-09	3.587621	1	P-mT-Susp	mT blank	123779.6	[Histone H3]-trimethyl-L-lysine(9) demethylase OS=Homo sapiens OX=9606 GN=KDM4B PE=1 SV=1
Q9UDV7	2	1	12.3478	7.772E-16	3.093E-15	Infinity	1	P-0.5PG-mT	F-mT-2D	75379.08	Zinc finger protein 282 OS=Homo sapiens OX=9606 GN=ZNF282 PE=1 SV=3
Q8TC05;F5H5	2	1	15.1583	2.823E-05	4.343E-07	22.53922	1	mT blank	P-mT-Susp	81020.43	Nuclear protein MDM1 OS=Homo sapiens OX=9606 GN=MDM1 PE=1 SV=2
A0A1W2PNT1	2	1	11.7037	1.000E+00	1.052E-02	1	1	---	---	26603.05	Nck-associated protein 5 (Fragment) OS=Homo sapiens OX=9606 GN=NCKAP5 PE=1 SV=1
Q01085;E7ETJ	2	1	19.8252	5.995E-15	9.545E-15	10.46226	1	P-0.5PG-mT	P-mT-Susp	41932.97	Nucleolysin TIAR OS=Homo sapiens OX=9606 GN=TIAL1 PE=1 SV=1
P03951;H0Y5	2	1	16.457	8.326E-06	1.395E-07	4.769669	1	F-mT-2D	F-0.5PG-mT	72162.18	Coagulation factor XI OS=Homo sapiens OX=9606 GN=F11 PE=1 SV=1
P17023	2	1	17.8801	4.651E-04	6.130E-06	2.421397	0.99866	F-0.5PG-mT	F-mT-2D	53703.71	Zinc finger protein 19 OS=Homo sapiens OX=9606 GN=ZNF19 PE=1 SV=4
E9PNL0;E9PP4	2	1	17.4831	5.302E-12	7.537E-13	2.391472	1	P-0.5PG-mT	mT blank	17921.72	Leucine-rich repeat-containing protein 14 (Fragment) OS=Homo sapiens OX=9606 GN=LRRC14 PE=1 S
Q6NSJ2;M0R0	2	1	21.6274	4.129E-08	1.053E-09	1.779751	1	F-mT-2D	mT blank	72653.22	Pleckstrin homology-like domain family B member 3 OS=Homo sapiens OX=9606 GN=PHLDB3 PE=1 SV
Q9NS15	3	1	8.0888	2.022E-06	3.725E-08	25.24493	1	mT blank	P-mT-Susp	146545	Latent-transforming growth factor beta-binding protein 3 OS=Homo sapiens OX=9606 GN=LTBP3 PE=1
C9JIS1;C9JXA5	2	1	28.3208	9.830E-07	1.904E-08	9.136178	1	P-0.5PG-mT	P-mT-Susp	26051.15	Guanine nucleotide-binding protein G(I)/G(S)/G(T) subunit beta-2 (Fragment) OS=Homo sapiens OX=9
Q9H0W8	2	1	9.2941	1.709E-08	4.876E-10	2.625202	1	F-mT-2D	F-0.5PG-mT	57878.93	Protein SMG9 OS=Homo sapiens OX=9606 GN=SMG9 PE=1 SV=1
C9J9M8;Q86Y	3	1	14.9929	5.983E-04	7.795E-06	2.960459	0.99776	mT blank	P-mT-Susp	76407.61	RING-type E3 ubiquitin transferase (Fragment) OS=Homo sapiens OX=9606 GN=DZIP3 PE=1 SV=1
Q9UHB7	2	1	14.9334	5.067E-08	1.253E-09	2.962196	1	P-0.5PG-mT	mT blank	127858.5	AF4/FMR2 family member 4 OS=Homo sapiens OX=9606 GN=AFF4 PE=1 SV=1
E9PG71;P547	2	1	11.8544	9.061E-11	6.062E-12	5.900621	1	P-mT-Susp	mT blank	107370	Receptor protein-tyrosine kinase OS=Homo sapiens OX=9606 GN=EPHA4 PE=1 SV=1
P98187	3	1	14.5491	4.811E-08	1.208E-09	3.139956	1	P-mT-Susp	F-0.5PG-mT	60679.06	Cytochrome P450 4F8 OS=Homo sapiens OX=9606 GN=CYP4F8 PE=1 SV=1
Q13123;A0A0	3	1	19.8225	4.232E-06	7.504E-08	4.546982	1	P-mT-Susp	mT blank	65716.43	Protein Red OS=Homo sapiens OX=9606 GN=IK PE=1 SV=3
Q9Y473	2	1	9.5618	1.206E-07	2.734E-09	2.66565	1	mT blank	P-mT-Susp	84061.75	Zinc finger protein 175 OS=Homo sapiens OX=9606 GN=ZNF175 PE=1 SV=1
Q9P2P5;A0A2	3	1	14.6319	1.719E-04	2.392E-06	7.115813	0.99988	P-0.5PG-mT	P-mT-Susp	176738.9	E3 ubiquitin-protein ligase HECW2 OS=Homo sapiens OX=9606 GN=HECW2 PE=1 SV=2
A0A0D9SFG5;	3	1	17.1025	1.826E-07	4.004E-09	Infinity	1	P-0.5PG-mT	P-mT-Susp	56049.32	Zinc finger protein 302 OS=Homo sapiens OX=9606 GN=ZNF302 PE=1 SV=1
Q8N7H5	2	1	14.9698	1.447E-03	1.800E-05	Infinity	0.98935	P-mT-Susp	P-0.5PG-mT	60146.89	RNA polymerase II-associated factor 1 homolog OS=Homo sapiens OX=9606 GN=PAF1 PE=1 SV=2
P14410	2	1	15.1954	2.511E-05	3.919E-07	3.99235	1	mT blank	P-mT-Susp	210879	Sucrase-isomaltase_ intestinal OS=Homo sapiens OX=9606 GN=SI PE=1 SV=6
A0A087WT20	2	1	14.5393	4.170E-12	6.639E-13	5.21631	1	P-mT-Susp	mT blank	68406.7	DDB1- and CUL4-associated factor 13 OS=Homo sapiens OX=9606 GN=DCAF13 PE=1 SV=1
Q16630;F8WJ	2	1	11.0279	5.587E-04	7.303E-06	9.684624	0.99804	mT blank	P-mT-Susp	59380.96	Cleavage and polyadenylation specificity factor subunit 6 OS=Homo sapiens OX=9606 GN=CPSF6 PE=1
A0A0D9SF53;	2	1	10.4368	1.151E-04	1.631E-06	2.253103	0.99996	P-0.5PG-mT	P-mT-Susp	82161.5	RNA helicase OS=Homo sapiens OX=9606 GN=DDX3X PE=1 SV=1

Q5JVG8;F5H4	2	1	24.0966	4.387E-12	6.847E-13	3.364447	1	F-mT-2D	P-mT-Susp	53019.27	Zinc finger protein 506 OS=Homo sapiens OX=9606 GN=ZNF506 PE=2 SV=2
O75940	2	1	16.7286	1.686E-06	3.144E-08	3.898825	1	P-mT-Susp	mT blank	26882.23	Survival of motor neuron-related-splicing factor 30 OS=Homo sapiens OX=9606 GN=SMNDC1 PE=1 SV=
A0A2R8YEE0;	2	1	19.1412	1.134E-10	7.282E-12	4.549385	1	P-mT-Susp	mT blank	117025.3	Rho family-interacting cell polarization regulator 2 OS=Homo sapiens OX=9606 GN=RIPOR2 PE=1 SV=1
B4DWF2;E7EU	2	1	16.0351	8.570E-09	2.729E-10	2.861571	1	F-mT-2D	P-mT-Susp	61458.48	Zinc finger protein Helios OS=Homo sapiens OX=9606 GN=IKZF2 PE=1 SV=1
F8WAE6;Q86Y	2	1	10.1468	3.379E-09	1.223E-10	2.430294	1	F-mT-2D	mT blank	206233.1	Mediator of RNA polymerase II transcription subunit 12-like protein OS=Homo sapiens OX=9606 GN=M
F5H0V4;Q9HA	2	1	24.9194	8.659E-04	1.108E-05	4.007637	0.9955	mT blank	P-mT-Susp	33714.43	MLX-interacting protein (Fragment) OS=Homo sapiens OX=9606 GN=MLXIP PE=1 SV=1
E5RGK3	2	1	10.5767	1.199E-13	6.498E-14	Infinity	1	F-mT-2D	mT blank	15629.81	Zinc finger homeobox protein 4 (Fragment) OS=Homo sapiens OX=9606 GN=ZFHX4 PE=1 SV=1
G5E9C8;Q078	2	1	11.0758	6.172E-06	1.066E-07	8.223192	1	mT blank	P-mT-Susp	151827	Son of sevenless homolog 1 OS=Homo sapiens OX=9606 GN=SOS1 PE=1 SV=1
O75298;Q7RT	2	1	21.3968	3.426E-11	3.030E-12	2.861794	1	F-mT-2D	F-0.5PG-mT	59492	Reticulon-2 OS=Homo sapiens OX=9606 GN=RTN2 PE=1 SV=1
Q6P9F0;F5H0	2	1	23.259	1.633E-02	1.868E-04	4.44537	0.8329	mT blank	P-mT-Susp	78945.74	Coiled-coil domain-containing protein 62 OS=Homo sapiens OX=9606 GN=CCDC62 PE=1 SV=2
E5RIH3	2	1	18.9581	2.275E-04	3.122E-06	3.484119	0.99974	F-0.5PG-mT	P-mT-Susp	15731.65	PH and SEC7 domain-containing protein 3 (Fragment) OS=Homo sapiens OX=9606 GN=PSD3 PE=1 SV=
P17844;A0A7	2	1	16.8911	1.631E-07	3.627E-09	3.262371	1	P-mT-Susp	mT blank	69661.45	Probable ATP-dependent RNA helicase DDX5 OS=Homo sapiens OX=9606 GN=DDX5 PE=1 SV=1
G3V2J8;P0790	2	1	10.8158	4.060E-04	5.386E-06	3.477239	0.999	P-0.5PG-mT	F-mT-2D	20151.65	Heat shock protein HSP 90-alpha (Fragment) OS=Homo sapiens OX=9606 GN=HSP90AA1 PE=1 SV=1
E7ET87;E7EVI	2	1	11.9898	5.692E-12	7.714E-13	2.809316	1	P-mT-Susp	mT blank	67613.11	Transforming acidic coiled-coil-containing protein 1 OS=Homo sapiens OX=9606 GN=TACC1 PE=1 SV=1
E5RFM9;A0AV	2	1	19.3174	3.503E-11	3.065E-12	4.532627	1	P-mT-Susp	mT blank	59946.72	Transforming acidic coiled-coil-containing protein 1 (Fragment) OS=Homo sapiens OX=9606 GN=TACC
Q9NV72	2	1	11.2493	6.960E-06	1.184E-07	1.904315	1	F-0.5PG-mT	F-mT-2D	62271.81	Zinc finger protein 701 OS=Homo sapiens OX=9606 GN=ZNF701 PE=1 SV=3
P19013	2	1	25.8555	1.465E-05	2.371E-07	2.992272	1	P-mT-Susp	mT blank	56543.31	Keratin_ type II cytoskeletal 4 OS=Homo sapiens OX=9606 GN=KRT4 PE=1 SV=5
P08729	2	1	25.6374	7.417E-05	1.083E-06	2.008297	0.99999	mT blank	P-mT-Susp	51442.77	Keratin_ type II cytoskeletal 7 OS=Homo sapiens OX=9606 GN=KRT7 PE=1 SV=5
Q5RHP9	2	1	14.369	5.545E-09	1.839E-10	2.261958	1	P-mT-Susp	F-mT-2D	169150.5	Glutamate-rich protein 3 OS=Homo sapiens OX=9606 GN=ERICH3 PE=1 SV=1
P29074	2	1	10.2536	6.575E-06	1.128E-07	4.204008	1	F-0.5PG-mT	F-mT-2D	106994.7	Tyrosine-protein phosphatase non-receptor type 4 OS=Homo sapiens OX=9606 GN=PTPN4 PE=1 SV=1
O60341;R4GM	2	1	11.2685	6.478E-05	9.621E-07	Infinity	0.99999	P-mT-Susp	F-0.5PG-mT	93416.01	Lysine-specific histone demethylase 1A OS=Homo sapiens OX=9606 GN=KDM1A PE=1 SV=2
E7ESJ3;Q9UPS	2	1	9.4025	1.772E-04	2.461E-06	1.590261	0.99987	P-0.5PG-mT	P-mT-Susp	197521.5	Ankyrin repeat domain-containing protein 26 OS=Homo sapiens OX=9606 GN=ANKRD26 PE=1 SV=2
O15234	1	1	10.9942	1.116E-08	3.403E-10	3.028742	1	P-mT-Susp	F-mT-2D	76392.13	Protein CASC3 OS=Homo sapiens OX=9606 GN=CASC3 PE=1 SV=2
G3V287;G3V2	1	1	7.0506	1.174E-03	1.479E-05	1.499091	0.9924	F-0.5PG-mT	P-mT-Susp	9420.891	Spermatogenesis-associated protein 7 OS=Homo sapiens OX=9606 GN=SPATA7 PE=1 SV=1
O14494	1	1	12.1344	1.372E-13	6.498E-14	3.668895	1	P-0.5PG-mT	P-mT-Susp	32555.31	Phospholipid phosphatase 1 OS=Homo sapiens OX=9606 GN=PLPP1 PE=1 SV=1
A0A0A0MT47	1	1	6.2479	2.525E-13	9.570E-14	12.29514	1	P-mT-Susp	P-0.5PG-mT	36070.83	M-phase phosphoprotein 8 (Fragment) OS=Homo sapiens OX=9606 GN=MPHOSPH8 PE=1 SV=1
Q9UBZ4	1	1	13.5702	5.995E-15	9.545E-15	13.81745	1	P-mT-Susp	P-0.5PG-mT	58427.13	DNA-(apurinic or apyrimidinic site) endonuclease 2 OS=Homo sapiens OX=9606 GN=APEX2 PE=1 SV=1
H0YM42;O43	1	1	13.03	1.913E-03	2.350E-05	2.158788	0.98379	mT blank	F-mT-2D	18516.18	Protein regulator of cytokinesis 1 (Fragment) OS=Homo sapiens OX=9606 GN=PRC1 PE=1 SV=1
A6NMQ1	1	1	9.476	8.298E-08	1.978E-09	1.861087	1	P-mT-Susp	F-0.5PG-mT	168282.5	DNA polymerase OS=Homo sapiens OX=9606 GN=POLA1 PE=1 SV=1
E9PK01;E9PQ	1	1	10.7796	3.174E-07	6.632E-09	1.576374	1	P-0.5PG-mT	mT blank	28935.33	Elongation factor 1-delta (Fragment) OS=Homo sapiens OX=9606 GN=EEF1D PE=1 SV=1
A0A5F9ZHI2;F	4	1	33.3302	1.124E-06	2.156E-08	9.344891	1	P-mT-Susp	F-0.5PG-mT	23568.24	Adenine DNA glycosylase OS=Homo sapiens OX=9606 GN=MUTYH PE=1 SV=1
A0A087WV57	1	1	10.56	2.489E-08	6.856E-10	1.846022	1	P-mT-Susp	mT blank	86827.4	Protein kinase C-binding protein 1 OS=Homo sapiens OX=9606 GN=ZMYND8 PE=1 SV=1
F8VS10;Q86X	1	1	12.213	1.207E-11	1.354E-12	3.36726	1	P-0.5PG-mT	F-0.5PG-mT	32646.7	Spermatogenesis-associated serine-rich protein 2 OS=Homo sapiens OX=9606 GN=SPATS2 PE=1 SV=1
A0A5F9ZHJ9	1	1	9.933	6.545E-11	4.780E-12	2.308793	1	F-mT-2D	F-0.5PG-mT	23785.21	Arf-GAP with coiled-coil_ ANK repeat and PH domain-containing protein 2 (Fragment) OS=Homo sapie
Q8ND76	1	1	8.9556	4.148E-08	1.055E-09	13.83207	1	P-0.5PG-mT	P-mT-Susp	39849.94	Cyclin-Y OS=Homo sapiens OX=9606 GN=CCNY PE=1 SV=2
A0A3B3ITE4	1	1	5.1852	1.805E-03	2.224E-05	3.035649	0.98511	F-0.5PG-mT	P-mT-Susp	80093.6	Zinc finger protein 23 OS=Homo sapiens OX=9606 GN=ZNF23 PE=1 SV=1
Q96DT6	1	1	0	1.254E-07	2.837E-09	2.022054	1	P-mT-Susp	mT blank	53067.77	Cysteine protease ATG4C OS=Homo sapiens OX=9606 GN=ATG4C PE=1 SV=1
H3BNV0;J3QS	1	1	10.1632	1.144E-08	3.463E-10	2.392369	1	P-0.5PG-mT	F-mT-2D	14624.6	Leucine-rich repeat-containing protein 36 OS=Homo sapiens OX=9606 GN=LRRC36 PE=1 SV=1
P46977	1	1	5.1346	1.192E-03	1.500E-05	1.78881	0.9922	P-mT-Susp	mT blank	81157.09	Dolichyl-diphosphooligosaccharide--protein glycosyltransferase subunit STT3A OS=Homo sapiens OX=
A0A096LPK7;	1	1	10.2906	1.144E-08	3.463E-10	2.380112	1	P-0.5PG-mT	F-mT-2D	50570.65	Thioredoxin-disulfide reductase OS=Homo sapiens OX=9606 GN=TXNRD2 PE=1 SV=1
A0A2R8Y5K9;	1	1	6.046	2.103E-10	1.196E-11	2.090486	1	P-mT-Susp	mT blank	168641.8	GATOR complex protein DEPDC5 (Fragment) OS=Homo sapiens OX=9606 GN=DEPDC5 PE=1 SV=1
A6PVD3;P566	1	1	6.1114	2.255E-11	2.244E-12	3.172981	1	P-mT-Susp	mT blank	23472.27	Transcription factor SOX-10 (Fragment) OS=Homo sapiens OX=9606 GN=SOX10 PE=1 SV=1
Q8IXT1	1	1	4.6096	0.000E+00	0.000E+00	144.0393	1	P-mT-Susp	P-0.5PG-mT	113555.3	DNA damage-induced apoptosis suppressor protein OS=Homo sapiens OX=9606 GN=DDIAS PE=2 SV=2
Q9P2Y4	1	1	5.2941	4.986E-10	2.341E-11	2.779996	1	P-mT-Susp	mT blank	77960.86	Zinc finger protein 219 OS=Homo sapiens OX=9606 GN=ZNF219 PE=1 SV=2
H3BRF9	1	1	5.76	6.050E-13	1.852E-13	2.778769	1	P-mT-Susp	mT blank	50059.32	Abscission/NoCut checkpoint regulator OS=Homo sapiens OX=9606 GN=ZFYVE19 PE=1 SV=1
H0Y9P3;K7ELX	1	1	10.0724	2.602E-07	5.524E-09	6.899096	1	F-0.5PG-mT	F-mT-2D	45631.84	Centrosomal protein of 192 kDa (Fragment) OS=Homo sapiens OX=9606 GN=CEP192 PE=1 SV=1
Q6P988	1	1	11.5868	7.080E-10	3.184E-11	2.354781	1	P-mT-Susp	mT blank	56668.96	Palmitoleoyl-protein carboxylesterase NOTUM OS=Homo sapiens OX=9606 GN=NOTUM PE=1 SV=2
I3L3L5	1	1	5.4466	1.855E-08	5.256E-10	2.493999	1	P-mT-Susp	mT blank	6142.723	Zinc finger protein 771 OS=Homo sapiens OX=9606 GN=ZNF771 PE=1 SV=1
Q5JX61	1	1	6.9052	7.954E-13	2.110E-13	6.901414	1	P-mT-Susp	P-0.5PG-mT	24049.01	Copine-1 (Fragment) OS=Homo sapiens OX=9606 GN=CPNE1 PE=1 SV=1
A0A0C3SFZ9;	1	1	13.6774	2.950E-01	3.127E-03	6.249704	0.29609	P-0.5PG-mT	F-0.5PG-mT	97646.46	F-BAR domain only protein 1 OS=Homo sapiens OX=9606 GN=FCHO1 PE=1 SV=1

G8JLA2	1	1	8.8532	5.079E-03	6.034E-05	2.571458	0.94387	F-0.5PG-mT	P-mT-Susp	17260.39	Myosin light polypeptide 6 OS=Homo sapiens OX=9606 GN=MYL6 PE=1 SV=1
Q6P047	1	1	4.9506	1.280E-01	1.375E-03	Infinity	0.46551	mT blank	P-mT-Susp	33963.07	Uncharacterized protein C8orf74 OS=Homo sapiens OX=9606 GN=C8orf74 PE=1 SV=3
Q96BW9;A0A(	2	1	9.7152	2.963E-02	3.326E-04	Infinity	0.74507	F-mT-2D	mT blank	51979.36	Phosphatidate cytidylyltransferase_ mitochondrial OS=Homo sapiens OX=9606 GN=TAMM41 PE=1 SV=
A0A2R8Y661	7	1	46.1772	1.481E-04	2.087E-06	3.465738	0.99992	mT blank	P-mT-Susp	175158	Cyclic nucleotide ras GEF OS=Homo sapiens OX=9606 GN=RAPGEF2 PE=1 SV=1
P11047	2	1	9.1099	6.089E-02	6.704E-04	Infinity	0.61528	P-0.5PG-mT	P-mT-Susp	183305.8	Laminin subunit gamma-1 OS=Homo sapiens OX=9606 GN=LAMC1 PE=1 SV=3
B4DXZ6;C9JY2	2	1	11.0917	6.322E-02	6.950E-04	1.570085	0.60795	F-0.5PG-mT	P-mT-Susp	68669.49	Fragile X mental retardation syndrome-related protein 1 OS=Homo sapiens OX=9606 GN=FXR1 PE=1 S
J3KNE0;A6NKT	5	1	24.7361	1.589E-08	4.550E-10	4.364839	1	P-mT-Susp	mT blank	198991.2	RanBP2-like and GRIP domain-containing protein 3 OS=Homo sapiens OX=9606 GN=RGPD3 PE=1 SV=2
Q5H9F3	2	1	11.6482	1.343E-14	1.748E-14	Infinity	1	F-0.5PG-mT	P-0.5PG-mT	184351.4	BCL-6 corepressor-like protein 1 OS=Homo sapiens OX=9606 GN=BCORL1 PE=1 SV=1
Q7Z7K2;C9JAX	2	1	11.9154	9.175E-05	1.318E-06	6.037782	0.99998	F-mT-2D	P-mT-Susp	67006.3	Zinc finger protein 467 OS=Homo sapiens OX=9606 GN=ZNF467 PE=1 SV=1
Q3KP31	2	1	10.6474	3.246E-05	4.951E-07	1.918599	1	P-mT-Susp	mT blank	69152.8	Zinc finger protein 791 OS=Homo sapiens OX=9606 GN=ZNF791 PE=1 SV=1
Q9P0K1	2	1	9.7795	6.817E-05	1.003E-06	12.10318	0.99999	F-mT-2D	P-mT-Susp	103056.1	Disintegrin and metalloproteinase domain-containing protein 22 OS=Homo sapiens OX=9606 GN=ADA
A0A286YES2;F	5	1	28.6723	8.026E-11	5.756E-12	3.364109	1	P-mT-Susp	mT blank	198801.5	RANBP2-like and GRIP domain-containing protein 1 OS=Homo sapiens OX=9606 GN=RGPD1 PE=4 SV=1
E7EPB6;P1356	2	0	14.6486					---	---	164381	Cystic fibrosis transmembrane conductance regulator (Fragment) OS=Homo sapiens OX=9606 GN=CFT
E7EVH7;F8W6	1	0	5.0761					---	---	83223.87	Kinesin light chain OS=Homo sapiens OX=9606 PE=3 SV=2
C9JTV7;Q8N5	1	0	6.9038					---	---	22261.67	Ephexin-1 (Fragment) OS=Homo sapiens OX=9606 GN=NGEF PE=1 SV=1
E9PRL4;Q1413	1	0	11.094					---	---	37536.04	Tripartite motif-containing protein 29 OS=Homo sapiens OX=9606 GN=TRIM29 PE=1 SV=1
A0A3B3IRS5;A	1	0	8.6998					---	---	75573.8	Forkhead box P1_ isoform CRA_g OS=Homo sapiens OX=9606 GN=FOXP1 PE=1 SV=1
A0A0G2JNE3;/	1	0	11.4488					---	---	44383.03	Killer cell immunoglobulin-like receptor 2DS4 OS=Homo sapiens OX=9606 GN=KIR2DS4 PE=4 SV=1
E5RI93;E5RG7	3	0	15.1927					---	---	186175.5	Zinc finger homeobox protein 4 (Fragment) OS=Homo sapiens OX=9606 GN=ZFHX4 PE=1 SV=2
Q13342	1	0	4.958					---	---	100333.3	Nuclear body protein SP140 OS=Homo sapiens OX=9606 GN=SP140 PE=1 SV=2
O60895	1	0	5.5968					---	---	19892.84	Receptor activity-modifying protein 2 OS=Homo sapiens OX=9606 GN=RAMP2 PE=1 SV=2
A0A590UJ53;(	1	0	11.3612					---	---	96550.05	KAT8 regulatory NSL complex subunit 3 OS=Homo sapiens OX=9606 GN=KANSL3 PE=1 SV=1
A0A3B3ITF1	2	0	16.7964					---	---	114495.9	Disks large homolog 2 OS=Homo sapiens OX=9606 GN=DLG2 PE=1 SV=1
Q15583	1	0	12.3446					---	---	43525.98	Homeobox protein TGIF1 OS=Homo sapiens OX=9606 GN=TGIF1 PE=1 SV=3
Q9NX65	1	0	5.1995					---	---	80153.88	Zinc finger and SCAN domain-containing protein 32 OS=Homo sapiens OX=9606 GN=ZSCAN32 PE=1 SV
H0Y306	1	0	6.3094					---	---	20522.87	Histone-lysine N-methyltransferase SUV39H2 (Fragment) OS=Homo sapiens OX=9606 GN=SUV39H2 PI
B0QYR0;B0QY	1	0	4.8636					---	---	11045.83	BTB/POZ domain-containing protein 3 (Fragment) OS=Homo sapiens OX=9606 GN=BTBD3 PE=1 SV=8
A0A0A0MRU9	1	0	12.6788					---	---	82430.51	Non-specific serine/threonine protein kinase OS=Homo sapiens OX=9606 GN=MARK2 PE=1 SV=1
H7BZ66;Q037	1	0	9.993					---	---	69617.97	Potassium voltage-gated channel subfamily C member 4 OS=Homo sapiens OX=9606 GN=KCNC4 PE=1
G3V2R5;G3V5	1	0	6.2407					---	---	14549.91	Myc-associated factor X OS=Homo sapiens OX=9606 GN=MAX PE=1 SV=1
B5MCC0;Q8W	1	0	5.1009					---	---	21717.93	Peroxynitrite isomerase THAP4 OS=Homo sapiens OX=9606 GN=THAP4 PE=1 SV=1
Q9NTI5	2	0	22.946					---	---	165922.1	Sister chromatid cohesion protein PDS5 homolog B OS=Homo sapiens OX=9606 GN=PDS5B PE=1 SV=1
A0A0J9YWD6;	1	0	11.6134					---	---	18043.38	Deformed epidermal autoregulatory factor 1 homolog (Fragment) OS=Homo sapiens OX=9606 GN=DE
E7EQT4;G3V3	1	0	5.375					---	---	147761.9	Apoptotic chromatin condensation inducer in the nucleus OS=Homo sapiens OX=9606 GN=ACIN1 PE=1
A0A0A0MSA4	4	0	16.3247					---	---	116452.1	Band 4.1-like protein 3 OS=Homo sapiens OX=9606 GN=EPB41L3 PE=1 SV=1
B7Z6K7	1	0	4.9241					---	---	100645.1	Zinc finger protein 814 OS=Homo sapiens OX=9606 GN=ZNF814 PE=1 SV=2
K7EJ76	1	0	12.9092					---	---	35369.03	Putative Polycomb group protein ASXL3 OS=Homo sapiens OX=9606 GN=ASXL3 PE=1 SV=1
A0A5F9ZHX9;.	1	0	14.315					---	---	44916.79	DNA polymerase subunit gamma-2_ mitochondrial (Fragment) OS=Homo sapiens OX=9606 GN=POLG2
Q9P1V8	1	0	5.2087					---	---	77378.9	Sterile alpha motif domain-containing protein 15 OS=Homo sapiens OX=9606 GN=SAMD15 PE=2 SV=1
Q6NX45	1	0	5.4344					---	---	56721.37	Zinc finger protein 774 OS=Homo sapiens OX=9606 GN=ZNF774 PE=1 SV=2
A0A0C4DGA7;	1	0	10.0712					---	---	75878.59	Actin-binding LIM protein 3 OS=Homo sapiens OX=9606 GN=ABLIM3 PE=1 SV=1
E9PGI0;Q6UW	1	0	6.4302					---	---	45244.2	Arylsulfatase K OS=Homo sapiens OX=9606 GN=ARSK PE=1 SV=1
A0A6Q8PFA6;	1	0	5.3158					---	---	78726.87	Kinesin-like protein OS=Homo sapiens OX=9606 GN=KIF2A PE=1 SV=1
A0A0D9SF71	1	0	10.463					---	---	4868.59	Zinc finger E-box-binding homeobox 2 OS=Homo sapiens OX=9606 GN=ZEB2 PE=4 SV=1
P36888	1	0	13.5716					---	---	114785.6	Receptor-type tyrosine-protein kinase FLT3 OS=Homo sapiens OX=9606 GN=FLT3 PE=1 SV=2
O15440	1	0	11.3194					---	---	161744.2	Multidrug resistance-associated protein 5 OS=Homo sapiens OX=9606 GN=ABCC5 PE=1 SV=2
Q02818	1	0	5.1311					---	---	53879.39	Nucleobindin-1 OS=Homo sapiens OX=9606 GN=NUCB1 PE=1 SV=4
A0A182DWI3;	1	0	6.4518					---	---	62948.53	Thioredoxin-disulfide reductase OS=Homo sapiens OX=9606 GN=TXNRD1 PE=1 SV=1
O14576	1	0	10.3944					---	---	73468.73	Cytoplasmic dynein 1 intermediate chain 1 OS=Homo sapiens OX=9606 GN=DYNC1I1 PE=1 SV=2
A0A494C1C7;/	1	0	5.8607					---	---	26528.27	Uncharacterized protein OS=Homo sapiens OX=9606 PE=4 SV=1

F8W910	1	0	12.4108					---	---	16607.52	RNA-binding protein 5 (Fragment) OS=Homo sapiens OX=9606 GN=RBM5 PE=1 SV=8
F2Z2D4	1	0	11.8454					---	---	23587.68	Patatin-like phospholipase domain-containing protein 7 OS=Homo sapiens OX=9606 GN=PNPLA7 PE=1
H7C508	1	0	7.061					---	---	6792.365	Short transient receptor potential channel 1 (Fragment) OS=Homo sapiens OX=9606 GN=TRPC1 PE=4 S
B5ME80;F8W/	1	0	11.926					---	---	122223.6	Semaphorin-5B (Fragment) OS=Homo sapiens OX=9606 GN=SEMA5B PE=1 SV=2
A0A0G2JMX7;	2	0	21.8002					---	---	81143.03	Microtubule-associated protein OS=Homo sapiens OX=9606 GN=MAPT PE=1 SV=1
P09769;Q5TG	1	0	5.4961					---	---	60106.03	Tyrosine-protein kinase Fgr OS=Homo sapiens OX=9606 GN=FGR PE=1 SV=2
A0A0D9SFY2;	1	0	13.4076					---	---	28165.11	Secretin receptor (Fragment) OS=Homo sapiens OX=9606 GN=SCTR PE=4 SV=1
E9PLT4	4	0	27.884					---	---	6501.273	Adenine DNA glycosylase OS=Homo sapiens OX=9606 GN=MUTYH PE=4 SV=1
Q9NS91	1	0	9.7102					---	---	57078.15	E3 ubiquitin-protein ligase RAD18 OS=Homo sapiens OX=9606 GN=RAD18 PE=1 SV=2
C9J6T3	1	0	12.4258					---	---	8511.772	Zinc finger protein ZIC 4 (Fragment) OS=Homo sapiens OX=9606 GN=ZIC4 PE=4 SV=1
H7BXL6	1	0	10.8518					---	---	89532.7	Otogelin-like protein (Fragment) OS=Homo sapiens OX=9606 GN=OTOGL PE=1 SV=1
Q5VYS8	1	0	3.6274					---	---	173396.5	Terminal uridylyltransferase 7 OS=Homo sapiens OX=9606 GN=TUT7 PE=1 SV=1
A0A2R8Y4P5	1	0	11.5638					---	---	14413.38	Required for meiotic nuclear division protein 1 homolog (Fragment) OS=Homo sapiens OX=9606 GN=R
A0A3B3IRU6;/	2	0	9.7836					---	---	164828.7	Paternally-expressed gene 3 protein OS=Homo sapiens OX=9606 GN=PEG3 PE=1 SV=1
Q9C0B2	1	0	4.7616					---	---	179957.7	Cilia- and flagella-associated protein 74 OS=Homo sapiens OX=9606 GN=CFAP74 PE=2 SV=3
H0YBJ5	1	0	23.2041					---	---	6289.843	La-related protein 1 (Fragment) OS=Homo sapiens OX=9606 GN=LARP1 PE=1 SV=1
D6RCM2;D6RI	1	0	5.7524					---	---	12877.01	28S ribosomal protein S18c_ mitochondrial OS=Homo sapiens OX=9606 GN=MRPS18C PE=1 SV=1
F8VWZ5	1	0	15.463					---	---	16980.93	H2.0-like homeobox protein (Fragment) OS=Homo sapiens OX=9606 GN=HLX PE=1 SV=1
A0A0A0MR47	1	0	5.9138					---	---	70434.5	Neurotrophin receptor-interacting factor homolog OS=Homo sapiens OX=9606 GN=ZNF274 PE=1 SV=1
O94830	1	0	10.2118					---	---	81716.26	Phospholipase DDHD2 OS=Homo sapiens OX=9606 GN=DDHD2 PE=1 SV=2
A0A3B3ITZ5	1	0	6.9506					---	---	161873.6	DBF4-type zinc finger-containing protein 2 (Fragment) OS=Homo sapiens OX=9606 GN=ZDBF2 PE=1 SV
B1ANB7;Q8TD	1	0	7.5441					---	---	37756.14	Mucolipin 3_ isoform CRA_d OS=Homo sapiens OX=9606 GN=MCOLN3 PE=1 SV=1
Q8TF46;E9PI2	3	0	25.479					---	---	122270.3	DIS3-like exonuclease 1 OS=Homo sapiens OX=9606 GN=DIS3L PE=1 SV=2
H7C224	1	0	12.5966					---	---	42738.85	Interleukin-1 receptor-associated kinase 1 (Fragment) OS=Homo sapiens OX=9606 GN=IRAK1 PE=1 SV=
A0A0A0MQX1	1	0	10.1988					---	---	240513.4	Unconventional myosin-X OS=Homo sapiens OX=9606 GN=MYO10 PE=1 SV=1
H7C146	1	0	13.7904					---	---	7552.551	Microtubule-associated serine/threonine-protein kinase 4 (Fragment) OS=Homo sapiens OX=9606 GN=
A1A519;A2VC	1	0	10.8358					---	---	37728.29	Protein FAM170A OS=Homo sapiens OX=9606 GN=FAM170A PE=2 SV=1
Q6PDB4	1	0	11.0932					---	---	68701.03	Zinc finger protein 880 OS=Homo sapiens OX=9606 GN=ZNF880 PE=2 SV=2
F5H0Y6;F5H6\	1	0	12.5724					---	---	13890.18	NXPE family member 1 (Fragment) OS=Homo sapiens OX=9606 GN=NXPE1 PE=1 SV=8
A0A0U1RRM8	1	0	10.6626					---	---	62367.34	Fermitin family homolog 2 (Fragment) OS=Homo sapiens OX=9606 GN=FERMT2 PE=1 SV=1
F5H6S5;Q9NZ	1	0	5.4768					---	---	21242.83	Complement C1r subcomponent-like protein (Fragment) OS=Homo sapiens OX=9606 GN=C1RL PE=1 S
A0A1B0GTW6	7	0	43.7704					---	---	175840.1	Band 4.1-like protein 1 OS=Homo sapiens OX=9606 GN=EPB41L1 PE=1 SV=2
C9JZI2	1	0	7.4113					---	---	11637.39	Septin-2 (Fragment) OS=Homo sapiens OX=9606 GN=SEPTIN2 PE=1 SV=1
Q5JPF3	2	0	9.0724					---	---	201516.2	Ankyrin repeat domain-containing protein 36C OS=Homo sapiens OX=9606 GN=ANKRD36C PE=1 SV=3
Q969X5	2	0	11.2683					---	---	32991.54	Endoplasmic reticulum-Golgi intermediate compartment protein 1 OS=Homo sapiens OX=9606 GN=ER
C9K0V9;F8WD	1	0	6.7501					---	---	78816.97	Ataxin-7-like protein 1 (Fragment) OS=Homo sapiens OX=9606 GN=ATXN7L1 PE=1 SV=1
P98082;D6RFI	3	0	26.0682					---	---	82562.04	Disabled homolog 2 OS=Homo sapiens OX=9606 GN=DAB2 PE=1 SV=3
A0A0G2JLQ8;/	2	0	7.8626					---	---	101545.3	NACHT_ LRR and PYD domains-containing protein 2 (Fragment) OS=Homo sapiens OX=9606 GN=NLRP2
A0A6Q8PGC2;	3	0	20.7142					---	---	69410.4	Phosphatase and actin regulator OS=Homo sapiens OX=9606 GN=PHACTR1 PE=1 SV=1
A0A1B0GVP4	1	0	4.7772					---	---	214110.6	Ligand-dependent nuclear receptor corepressor-like protein OS=Homo sapiens OX=9606 GN=LCORL PE
A0A087WZY0;	1	0	4.6234					---	---	22060.09	Ceroid-lipofuscinosis neuronal protein 5 OS=Homo sapiens OX=9606 GN=CLN5 PE=1 SV=3
P35612	1	0	10.4874					---	---	81310.58	Beta-adducin OS=Homo sapiens OX=9606 GN=ADD2 PE=1 SV=3
A0A7I2V2X6;A	1	0	13.314					---	---	57929.96	60 kDa chaperonin OS=Homo sapiens OX=9606 GN=HSPD1 PE=1 SV=1
E5RGD1	1	0	11.555					---	---	25278.93	Androglobin OS=Homo sapiens OX=9606 GN=ADGB PE=1 SV=1
Q9GZU2	2	0	10.8183					---	---	183051.6	Paternally-expressed gene 3 protein OS=Homo sapiens OX=9606 GN=PEG3 PE=1 SV=1
B3KNX7;Q131	1	0	5.1283					---	---	58488.23	Non-specific serine/threonine protein kinase OS=Homo sapiens OX=9606 GN=PAK1 PE=1 SV=1
C9IZE3;C9JVX(	3	0	18.6038					---	---	20197.77	Zinc finger protein 808 (Fragment) OS=Homo sapiens OX=9606 GN=ZNF808 PE=1 SV=1
Q8IYE0	3	0	15.6666					---	---	113205.9	Coiled-coil domain-containing protein 146 OS=Homo sapiens OX=9606 GN=CCDC146 PE=1 SV=2
O15063	1	0	9.2082					---	---	117274.5	Granule associated Rac and RHOG effector protein 1 OS=Homo sapiens OX=9606 GN=GARRE1 PE=1 SV
A0A3B3ITK0;A	1	0	12.3456					---	---	134458.8	Thrombospondin-2 OS=Homo sapiens OX=9606 GN=THBS2 PE=1 SV=1

Q9Y6A1	1	0	6.1241					---	---	85736.22	Protein O-mannosyl-transferase 1 OS=Homo sapiens OX=9606 GN=POMT1 PE=1 SV=3
E9PE72;P2241	1	0	5.3894					---	---	43443.76	Ectonucleotide pyrophosphatase/phosphodiesterase family member 1 OS=Homo sapiens OX=9606 GN
G3V5M4	1	0	5.5842					---	---	14095.27	Alpha-actinin-1 (Fragment) OS=Homo sapiens OX=9606 GN=ACTN1 PE=1 SV=1
E5RGZ2;H0YKJ	1	0	4.5444					---	---	43880.51	Homeobox-containing protein 1 OS=Homo sapiens OX=9606 GN=HMBOX1 PE=1 SV=1
D6R938;E9PB(	1	0	8.7774					---	---	56925.72	Calcium/calmodulin-dependent protein kinase OS=Homo sapiens OX=9606 GN=CAMK2D PE=1 SV=1
A0A4W8VX11	2	0	8.7388					---	---	211501.6	Pericentriolar material 1 protein OS=Homo sapiens OX=9606 GN=PCM1 PE=4 SV=1
Q96NG3;A0A(	2	0	9.5043					---	---	77054.62	Outer dynein arm-docking complex subunit 4 OS=Homo sapiens OX=9606 GN=ODAD4 PE=1 SV=2
D3DQV9;H0Y3	1	0	4.9958					---	---	102843.3	Eukaryotic translation initiation factor 4 gamma 2 (Fragment) OS=Homo sapiens OX=9606 GN=EIF4G2
C9JRQ0	1	0	6.4391					---	---	15922.68	Suppressor of tumorigenicity 7 protein (Fragment) OS=Homo sapiens OX=9606 GN=ST7 PE=1 SV=1
F5H3E8;P5779	1	0	12.8456					---	---	13408.82	Calcium-binding protein 4 OS=Homo sapiens OX=9606 GN=CABP4 PE=1 SV=1
Q96FN4	2	0	8.8732					---	---	61874.28	Copine-2 OS=Homo sapiens OX=9606 GN=CPNE2 PE=1 SV=3
E9PJ80;E9PKX	1	0	6.7749					---	---	4724.853	Oxysterol-binding protein-related protein 9 OS=Homo sapiens OX=9606 GN=OSBPL9 PE=1 SV=1
A0A0G2JRY5;(	1	0	5.0522					---	---	50465.44	Transcription initiation factor TFIID subunit 4 (Fragment) OS=Homo sapiens OX=9606 GN=TAF4 PE=1 S
Q9NZV7	1	0	5.2598					---	---	62304.42	Zinc finger imprinted 2 OS=Homo sapiens OX=9606 GN=ZIM2 PE=1 SV=1
P24723	1	0	8.9822					---	---	79197.27	Protein kinase C eta type OS=Homo sapiens OX=9606 GN=PRKCH PE=1 SV=4
Q96SK3	1	0	9.784					---	---	83129.89	Zinc finger protein 607 OS=Homo sapiens OX=9606 GN=ZNF607 PE=1 SV=3
I3L2H4	1	0	12.1224					---	---	7836.641	Hepatocyte growth factor-regulated tyrosine kinase substrate (Fragment) OS=Homo sapiens OX=9606
E9PQS1;Q9H0	1	0	4.8474					---	---	26789.9	Ester hydrolase C11orf54 (Fragment) OS=Homo sapiens OX=9606 GN=C11orf54 PE=1 SV=1
D7R525;E5RJ5	1	0	12.8912					---	---	24960.04	Mitogen-activated protein kinase OS=Homo sapiens OX=9606 GN=MAPK9 PE=1 SV=1
