# Supplementary material for: Unlocking the Potential of Human-Induced Pluripotent Stem Cells: Cellular Responses and Secretome Profiles in Peptide Hydrogel 3D Culture
Source: Cells. 2024 Jan 12;13(2):143. doi: 10.3390/cells13020143 (PMC10814310; doi:10.3390/cells13020143)
Supplement: Supplementary file 1 [file cells-13-00143-s001.zip › Supplementary Table S2. Proteins profiles selected from 3D hiPSCs cultured in E8.pdf]

Supplementary Table S2. Proteins profile of 3D hiPSCs cultured in E8 medium

Accession	Peptide count	Unique peptides	Confidence score	Anova (p)	q Value	Max fold change	Power	Highest mean condition	Lowest mean condition	Mass	Description
P02787;H7	87	73	1422.1987	6.58E-10	7.30E-09	5.36132	1	E8 blank	P-0.3PG-E8-	79345.08	Serotransferrin OS=Homo sapiens OX=9606 GN=TF PE=1 SV=3
P02790;Q9	26	22	374.1824	9.49E-09	4.54E-08	4.34388	1	E8 blank	P-0.3PG-E8-	52417.79	Hemopexin OS=Homo sapiens OX=9606 GN=HPX PE=1 SV=2
P78332;E9	18	16	86.9278	0.0022038	0.00247	1.27687	0.992	E8 blank	P-0.3PG-E8-	129271.4	RNA-binding protein 6 OS=Homo sapiens OX=9606 GN=RBM6 PE=1 SV=5
O60258	20	14	109.1145	5.36E-13	1.72E-10	38.4221	1	P-0.3PG-E8-	E8 blank	25176.57	Fibroblast growth factor 17 OS=Homo sapiens OX=9606 GN=FGF17 PE=1 SV=1
A0A7I2V4I5	15	14	84.2184	1.90E-06	3.30E-06	2.44488	1	F-0.3PG-E8-	E8 blank	197391	Zinc finger CCCH domain-containing protein 13 OS=Homo sapiens OX=9606 GN=ZC3H13 PE=1 SV=1
Q5THK1;C9	16	13	95.7559	0.0001964	0.000244	1.29476	1	E8 blank	P-0.3PG-E8-	241291.9	Protein PRR14L OS=Homo sapiens OX=9606 GN=PRR14L PE=1 SV=1
A0A1B0GVI	13	13	114.1188	9.72E-07	1.83E-06	1.8369	1	E8 blank	F-0.3PG-E8-	63574.34	Keratin_ type I cytoskeletal 10 OS=Homo sapiens OX=9606 GN=KRT10 PE=1 SV=2
B7ZKJ8;Q14	14	13	162.8706	5.72E-08	1.80E-07	6.59355	1	E8 blank	P-0.3PG-E8-	104109.3	ITIH4 protein OS=Homo sapiens OX=9606 GN=ITIH4 PE=1 SV=1
Q9H792;H0	14	12	83.6328	0.0100731	0.010888	1.14383	0.8958	P-0.3PG-E8-	F-0.3PG-E8-	195159.7	Inactive tyrosine-protein kinase PEAK1 OS=Homo sapiens OX=9606 GN=PEAK1 PE=1 SV=4
P04264;A0	17	12	161.5225	3.23E-08	1.12E-07	3.09591	1	E8 blank	P-0.3PG-E8-	66209.9	Keratin_ type II cytoskeletal 1 OS=Homo sapiens OX=9606 GN=KRT1 PE=1 SV=6
Q9H9B1;A0	15	11	91.9865	1.83E-09	1.48E-08	7.86546	1	E8 blank	P-0.3PG-E8-	144089.8	Histone-lysine N-methyltransferase EHMT1 OS=Homo sapiens OX=9606 GN=EHMT1 PE=1 SV=4
Q5TCS8;J3K	15	11	105.056	6.17E-09	3.47E-08	5.50706	1	P-0.3PG-E8-	E8 blank	222838.6	Adenylate kinase 9 OS=Homo sapiens OX=9606 GN=AK9 PE=1 SV=2
A8MYJ1;A0	10	10	50.4005	7.72E-11	1.90E-09	14.721	1	P-0.3PG-E8-	E8 blank	163153.1	A-kinase anchor protein 13 OS=Homo sapiens OX=9606 GN=AKAP13 PE=1 SV=3
A0A0C4DH	13	10	73.0751	1.28E-07	3.34E-07	1.79947	1	P-0.3PG-E8-	E8 blank	178352.8	Latent-transforming growth factor beta-binding protein 4 OS=Homo sapiens OX=9606 GN=LTBP4 PE=1 SV=1
A0A669KB7	13	10	74.981	6.27E-07	1.25E-06	3.23651	1	E8 blank	P-0.3PG-E8-	217419.6	Microtubule-associated protein OS=Homo sapiens OX=9606 GN=MAP2 PE=1 SV=1
Q92608;E5	11	10	58.9666	1.41E-08	6.11E-08	8.27711	1	F-0.3PG-E8-	E8 blank	213260.4	Dedicator of cytokinesis protein 2 OS=Homo sapiens OX=9606 GN=DOCK2 PE=1 SV=2
P02768;A0	14	10	151.7058	2.31E-10	4.00E-09	150.855	1	P-0.3PG-E8-	E8 blank	71362.71	Albumin OS=Homo sapiens OX=9606 GN=ALB PE=1 SV=2
E9PMT2;F8	13	10	78.3685	0.4356509	0.437014	1.02738	0.1496	E8 blank	P-0.3PG-E8-	160934.8	LIM domain only protein 7 OS=Homo sapiens OX=9606 GN=LMO7 PE=1 SV=1
Q86U86;E7	11	9	69.2947	7.09E-09	3.82E-08	36.9035	1	F-0.3PG-E8-	E8 blank	194202.8	Protein polybromo-1 OS=Homo sapiens OX=9606 GN=PBRM1 PE=1 SV=1
Q6UB98	9	9	59.0844	5.22E-06	8.26E-06	1.5821	1	F-0.3PG-E8-	P-0.3PG-E8-	237191.6	Ankyrin repeat domain-containing protein 12 OS=Homo sapiens OX=9606 GN=ANKRD12 PE=1 SV=3
P20849	10	9	58.2893	1.70E-09	1.43E-08	4.73469	1	E8 blank	P-0.3PG-E8-	92496.69	Collagen alpha-1(IX) chain OS=Homo sapiens OX=9606 GN=COL9A1 PE=1 SV=3
P31946	11	8	87.1214	8.19E-08	2.41E-07	5.91279	1	P-0.3PG-E8-	E8 blank	28196.51	14-3-3 protein beta/alpha OS=Homo sapiens OX=9606 GN=YWHAB PE=1 SV=3
P58397	8	8	47.2367	9.19E-06	1.38E-05	1.66909	1	E8 blank	P-0.3PG-E8-	182637.2	A disintegrin and metalloproteinase with thrombospondin motifs 12 OS=Homo sapiens OX=9606 GN=ADAMTS
O75095	10	8	49.85	2.33E-06	3.99E-06	1.99133	1	F-0.3PG-E8-	E8 blank	174871.9	Multiple epidermal growth factor-like domains protein 6 OS=Homo sapiens OX=9606 GN=MEGF6 PE=1 SV=4
Q9NS15;H0	10	8	47.3732	6.69E-09	3.64E-08	16.1213	1	P-0.3PG-E8-	E8 blank	146545	Latent-transforming growth factor beta-binding protein 3 OS=Homo sapiens OX=9606 GN=LTBP3 PE=1 SV=4
A0A1B0GTV	7	7	35.0462	0.0532901	0.055453	1.08969	0.577	F-0.3PG-E8-	P-0.3PG-E8-	141126.2	Tight junction protein ZO-2 OS=Homo sapiens OX=9606 GN=TJP2 PE=1 SV=1
A0A2R8Y3N	8	7	42.6113	2.20E-08	8.51E-08	3.21985	1	E8 blank	P-0.3PG-E8-	74234.3	Uncharacterized protein OS=Homo sapiens OX=9606 PE=4 SV=1
A0A5F9ZHN	7	7	41.0762	0.0104666	0.011295	1.24938	0.891	P-0.3PG-E8-	F-0.3PG-E8-	129653.9	Tyrosine-protein kinase OS=Homo sapiens OX=9606 GN=JAK1 PE=1 SV=1
Q6P280	7	7	31.0335	3.79E-06	6.22E-06	2.32047	1	F-0.3PG-E8-	E8 blank	67518.42	Zinc finger protein 529 OS=Homo sapiens OX=9606 GN=ZNF529 PE=1 SV=2
P01106	9	7	75.8264	0.0011157	0.001296	1.58573	0.9986	F-0.3PG-E8-	E8 blank	51135.39	Myc proto-oncogene protein OS=Homo sapiens OX=9606 GN=MYC PE=1 SV=2
A0A669KB1	10	7	71.3639	0.0002503	0.000308	1.72084	1	E8 blank	F-0.3PG-E8-	211323.5	Golgi-specific brefeldin A-resistance guanine nucleotide exchange factor 1 OS=Homo sapiens OX=9606 GN=GBI
Q99795	11	7	67.4585	9.26E-09	4.46E-08	17.9631	1	P-0.3PG-E8-	E8 blank	36259.29	Cell surface A33 antigen OS=Homo sapiens OX=9606 GN=GPA33 PE=1 SV=1
P05787	10	7	69.4078	1.44E-05	2.12E-05	1.28496	1	E8 blank	P-0.3PG-E8-	53704.36	Keratin_ type II cytoskeletal 8 OS=Homo sapiens OX=9606 GN=KRT8 PE=1 SV=7
Q9NQ90;F5	6	6	29.467	0.0001229	0.000158	1.50849	1	P-0.3PG-E8-	E8 blank	114768	Anoctamin-2 OS=Homo sapiens OX=9606 GN=ANO2 PE=1 SV=2
A0A2R8Y59	6	6	40.55	9.07E-06	1.37E-05	7.17463	1	P-0.3PG-E8-	E8 blank	247036.9	A-kinase anchor protein 9 OS=Homo sapiens OX=9606 GN=AKAP9 PE=1 SV=2
P04004	7	6	38.931	5.85E-05	7.84E-05	3.18685	1	E8 blank	P-0.3PG-E8-	55104.04	Vitronectin OS=Homo sapiens OX=9606 GN=VTN PE=1 SV=1
F5H101;Q7	6	6	30.6068	0.0059521	0.0065	1.16347	0.9488	F-0.3PG-E8-	E8 blank	122506	Nucleolar protein 8 OS=Homo sapiens OX=9606 GN=NOL8 PE=1 SV=1
A1Z1Q3;A0	6	6	28.4155	1.87E-05	2.69E-05	1.54658	1	F-0.3PG-E8-	E8 blank	47934.56	ADP-ribose glycohydrolase MACROD2 OS=Homo sapiens OX=9606 GN=MACROD2 PE=1 SV=2
A0A5F9ZGY	6	6	23.7794	0.152977	0.156144	1.14382	0.3375	P-0.3PG-E8-	E8 blank	208918.8	Ankyrin-2 OS=Homo sapiens OX=9606 GN=ANK2 PE=1 SV=1
Q4LE39;X6	6	6	31.7588	0.0022484	0.002511	1.33897	0.9916	E8 blank	F-0.3PG-E8-	148893.2	AT-rich interactive domain-containing protein 4B OS=Homo sapiens OX=9606 GN=ARID4B PE=1 SV=2
A0A669KA3	7	6	36.5929	1.06E-07	2.97E-07	2.14824	1	E8 blank	F-0.3PG-E8-	221297.4	Proprotein convertase subtilisin/kexin type 5 OS=Homo sapiens OX=9606 GN=PCSK5 PE=1 SV=1
Q8WYB5;A	7	6	43.0101	2.79E-08	1.02E-07	2.27531	1	P-0.3PG-E8-	E8 blank	234286.9	Histone acetyltransferase KAT6B OS=Homo sapiens OX=9606 GN=KAT6B PE=1 SV=3
G3XAE9;Q9	6	6	36.5987	1.28E-08	5.68E-08	5.81109	1	P-0.3PG-E8-	E8 blank	196689.1	KIAA0423_ isoform CRA_a OS=Homo sapiens OX=9606 GN=TOGARAM1 PE=1 SV=1
Q9P227	7	6	40.9496	1.92E-08	7.60E-08	4.10188	1	E8 blank	P-0.3PG-E8-	163446.7	Rho GTPase-activating protein 23 OS=Homo sapiens OX=9606 GN=ARHGAP23 PE=1 SV=2
A0A3F2YNX	9	6	40.6019	5.85E-10	7.17E-09	9.13102	1	P-0.3PG-E8-	E8 blank	227963.8	Histone acetyltransferase OS=Homo sapiens OX=9606 GN=KAT6A PE=1 SV=1

A0A7P0T9K	8	6	37.3366	2.72E-08	9.97E-08	3.86461	1	F-0.3PG-E8-	E8 blank	184446.1	Inactive ubiquitin carboxyl-terminal hydrolase 54 OS=Homo sapiens OX=9606 GN=USP54 PE=4 SV=1
P09038;A0	18	6	144.2762	5.77E-08	1.80E-07	3.952	1	E8 blank	P-0.3PG-E8-	31112.17	Fibroblast growth factor 2 OS=Homo sapiens OX=9606 GN=FGF2 PE=1 SV=3
P49736;F8V	8	6	56.1122	1.72E-11	8.49E-10	47.1231	1	P-0.3PG-E8-	E8 blank	102580.6	DNA replication licensing factor MCM2 OS=Homo sapiens OX=9606 GN=MCM2 PE=1 SV=4
P17020;E9F	6	6	58.3896	6.53E-09	3.58E-08	4.49531	1	P-0.3PG-E8-	E8 blank	78866.82	Zinc finger protein 16 OS=Homo sapiens OX=9606 GN=ZNF16 PE=1 SV=3
Q5JQC9;A0	6	5	30.3502	4.98E-06	7.96E-06	4.20835	1	P-0.3PG-E8-	E8 blank	95903.07	A-kinase anchor protein 4 OS=Homo sapiens OX=9606 GN=AKAP4 PE=1 SV=1
E9PC84;F5H	6	5	27.7672	6.68E-05	8.88E-05	1.69162	1	P-0.3PG-E8-	E8 blank	216499.6	Tenascin OS=Homo sapiens OX=9606 GN=TNC PE=1 SV=1
Q86UP2;G3	6	5	28.4095	8.77E-08	2.56E-07	6.77535	1	P-0.3PG-E8-	E8 blank	156560.6	Kinectin OS=Homo sapiens OX=9606 GN=KTN1 PE=1 SV=1
Q9UHC6	5	5	33.2009	9.89E-10	9.75E-09	7.80911	1	F-0.3PG-E8-	E8 blank	150277.3	Contactin-associated protein-like 2 OS=Homo sapiens OX=9606 GN=CNTNAP2 PE=1 SV=1
A0A6Q8PFF	5	5	23.0745	3.56E-08	1.22E-07	4.15244	1	E8 blank	P-0.3PG-E8-	162649.2	IQ motif and SEC7 domain-containing protein 2 OS=Homo sapiens OX=9606 GN=IQSEC2 PE=1 SV=1
O60237	6	5	42.6444	2.76E-09	1.88E-08	3.56356	1	E8 blank	P-0.3PG-E8-	110860.2	Protein phosphatase 1 regulatory subunit 12B OS=Homo sapiens OX=9606 GN=PPP1R12B PE=1 SV=2
A0A2R8YDF	5	5	37.0484	2.71E-05	3.80E-05	2.05612	1	E8 blank	P-0.3PG-E8-	222800.3	Sodium channel protein OS=Homo sapiens OX=9606 GN=SCN9A PE=1 SV=1
Q96SK3;K7	5	5	36.371	3.51E-06	5.82E-06	1.99907	1	E8 blank	P-0.3PG-E8-	83129.89	Zinc finger protein 607 OS=Homo sapiens OX=9606 GN=ZNF607 PE=1 SV=3
G3V5V3;O6	6	5	34.6632	6.09E-08	1.89E-07	2.82492	1	E8 blank	P-0.3PG-E8-	114348.4	Nuclear export mediator factor NEMF (Fragment) OS=Homo sapiens OX=9606 GN=NEMF PE=1 SV=8
Q9UK13	5	5	25.6942	2.50E-09	1.76E-08	6.12429	1	P-0.3PG-E8-	E8 blank	73648.79	Zinc finger protein 221 OS=Homo sapiens OX=9606 GN=ZNF221 PE=1 SV=4
Q9Y3B9	5	5	34.4242	1.79E-07	4.36E-07	2.53043	1	E8 blank	P-0.3PG-E8-	31655.35	RRP15-like protein OS=Homo sapiens OX=9606 GN=RRP15 PE=1 SV=2
E9PKP7;P17	5	5	22.3428	0.0001789	0.000224	1.33165	1	F-0.3PG-E8-	E8 blank	87778.82	Nucleolar transcription factor 1 OS=Homo sapiens OX=9606 GN=UBTF PE=1 SV=1
Q14966;A0	6	5	35.0695	4.61E-07	9.52E-07	1.99265	1	E8 blank	P-0.3PG-E8-	222050.8	Zinc finger protein 638 OS=Homo sapiens OX=9606 GN=ZNF638 PE=1 SV=2
A0A0G2JNC	5	5	40.1665	1.68E-08	6.97E-08	6.53207	1	P-0.3PG-E8-	E8 blank	143300.1	Baculoviral IAP repeat-containing protein 1 OS=Homo sapiens OX=9606 GN=NAIP PE=1 SV=1
Q05707;A0	5	5	28.5012	2.25E-07	5.32E-07	2.90888	1	P-0.3PG-E8-	E8 blank	194599.3	Collagen alpha-1(XIV) chain OS=Homo sapiens OX=9606 GN=COL14A1 PE=1 SV=3
P29317	6	5	30.6203	1.89E-08	7.53E-08	6.67414	1	E8 blank	P-0.3PG-E8-	109749.3	Ephrin type-A receptor 2 OS=Homo sapiens OX=9606 GN=EPHA2 PE=1 SV=2
A0A0G2JM	6	5	37.2432	5.59E-10	7.17E-09	4.85029	1	E8 blank	P-0.3PG-E8-	81143.03	Microtubule-associated protein OS=Homo sapiens OX=9606 GN=MAPT PE=1 SV=1
P82970	8	5	50.4829	0.0020605	0.002325	1.35374	0.9931	E8 blank	P-0.3PG-E8-	31524.64	High mobility group nucleosome-binding domain-containing protein 5 OS=Homo sapiens OX=9606 GN=HMGN5
P57071;E7E	7	5	39.6635	1.61E-05	2.34E-05	3.58902	1	E8 blank	F-0.3PG-E8-	172234.6	PR domain zinc finger protein 15 OS=Homo sapiens OX=9606 GN=PRDM15 PE=1 SV=4
O14647;A0	7	5	42.9259	2.97E-05	4.14E-05	1.80886	1	P-0.3PG-E8-	E8 blank	212313.2	Chromodomain-helicase-DNA-binding protein 2 OS=Homo sapiens OX=9606 GN=CHD2 PE=1 SV=2
Q96JN2;C9J	7	5	41.6648	4.39E-07	9.22E-07	16.8904	1	P-0.3PG-E8-	E8 blank	135699.3	Coiled-coil domain-containing protein 136 OS=Homo sapiens OX=9606 GN=CCDC136 PE=1 SV=3
Q6P0N0	7	5	34.4364	6.11E-10	7.17E-09	34.1311	1	P-0.3PG-E8-	E8 blank	129998.1	Mis18-binding protein 1 OS=Homo sapiens OX=9606 GN=MIS18BP1 PE=1 SV=1
A0A0C4DG	6	5	35.2677	6.77E-10	7.35E-09	51.388	1	P-0.3PG-E8-	E8 blank	171235.7	THO complex subunit 2 OS=Homo sapiens OX=9606 GN=THOC2 PE=1 SV=1
P55075	6	5	37.9705	9.25E-10	9.41E-09	15.0334	1	P-0.3PG-E8-	E8 blank	26753.63	Fibroblast growth factor 8 OS=Homo sapiens OX=9606 GN=FGF8 PE=1 SV=1
O43320	7	5	48.4407	0.0262169	0.027869	1.44843	0.7349	E8 blank	F-0.3PG-E8-	23872.9	Fibroblast growth factor 16 OS=Homo sapiens OX=9606 GN=FGF16 PE=1 SV=1
A0A1B0GV4	6	5	44.8002	1.21E-09	1.12E-08	6.50277	1	P-0.3PG-E8-	E8 blank	186021.3	Kinesin-like protein KIF21A (Fragment) OS=Homo sapiens OX=9606 GN=KIF21A PE=1 SV=1
A0A3B3IRU	8	5	42.4465	9.93E-08	2.84E-07	3.40406	1	P-0.3PG-E8-	E8 blank	164828.7	Paternally-expressed gene 3 protein OS=Homo sapiens OX=9606 GN=PEG3 PE=1 SV=1
Q9ULD6	6	5	31.7338	1.39E-10	2.97E-09	8.43794	1	P-0.3PG-E8-	E8 blank	106788.9	Protein inturned OS=Homo sapiens OX=9606 GN=INTU PE=1 SV=2
P38398;A0	6	5	32.3447	0.0682498	0.070561	1.47307	0.5191	F-0.3PG-E8-	P-0.3PG-E8-	210230.3	Breast cancer type 1 susceptibility protein OS=Homo sapiens OX=9606 GN=BRCA1 PE=1 SV=2
Q92922	4	4	20.2251	7.80E-09	4.08E-08	37.2275	1	P-0.3PG-E8-	E8 blank	123380.5	SWI/SNF complex subunit SMARCC1 OS=Homo sapiens OX=9606 GN=SMARCC1 PE=1 SV=3
Q5TF58;H0	4	4	22.1492	0.0006393	0.000755	1.28924	0.9998	P-0.3PG-E8-	E8 blank	57841.48	Intermediate filament family orphan 2 OS=Homo sapiens OX=9606 GN=IFFO2 PE=2 SV=3
E7EWD6;Q	4	4	23.0424	1.12E-08	5.15E-08	2.81885	1	P-0.3PG-E8-	E8 blank	151777.8	Putative Polycomb group protein ASXL2 OS=Homo sapiens OX=9606 GN=ASXL2 PE=1 SV=1
A0A0G2JM	4	4	24.0884	9.33E-07	1.77E-06	6.65894	1	E8 blank	P-0.3PG-E8-	114004.6	NACHT_ LRR and PYD domains-containing protein 7 OS=Homo sapiens OX=9606 GN=NLRP7 PE=1 SV=1
Q8NEN0;A0	4	4	25.5146	7.50E-08	2.25E-07	5.5282	1	P-0.3PG-E8-	E8 blank	98121.32	Armadillo repeat-containing protein 2 OS=Homo sapiens OX=9606 GN=ARMC2 PE=2 SV=4
E7ESJ3;Q9U	4	4	28.5721	3.49E-07	7.55E-07	11.3971	1	F-0.3PG-E8-	E8 blank	197521.5	Ankyrin repeat domain-containing protein 26 OS=Homo sapiens OX=9606 GN=ANKRD26 PE=1 SV=2
A0A7P0TAK	4	4	28.9779	0.0002471	0.000305	1.79126	1	P-0.3PG-E8-	E8 blank	168046.8	WD repeat-containing protein 62 OS=Homo sapiens OX=9606 GN=WDR62 PE=4 SV=1
B7ZB24;F5H	4	4	27.9577	2.97E-11	1.19E-09	37.7153	1	P-0.3PG-E8-	E8 blank	99158.65	Multiple PDZ domain protein OS=Homo sapiens OX=9606 GN=MPDZ PE=1 SV=1
A0A0U1RRI	4	4	25.4903	5.48E-08	1.73E-07	4.57896	1	P-0.3PG-E8-	E8 blank	109153.3	PHD finger protein 14 OS=Homo sapiens OX=9606 GN=PHF14 PE=1 SV=1
Q12888;A6	4	4	20.3501	3.47E-05	4.77E-05	2.28032	1	E8 blank	F-0.3PG-E8-	215627.7	TP53-binding protein 1 OS=Homo sapiens OX=9606 GN=TP53BP1 PE=1 SV=2
A0A0A0MR	4	4	24.2111	2.94E-06	4.92E-06	2.85917	1	E8 blank	F-0.3PG-E8-	93097.78	Fibroblast growth factor receptor OS=Homo sapiens OX=9606 GN=FGFR2 PE=1 SV=1
Q5QPR3;Q9	4	4	21.1665	1.61E-06	2.84E-06	2.63446	1	E8 blank	F-0.3PG-E8-	91292.48	Cyclin-dependent kinase 11A OS=Homo sapiens OX=9606 GN=CDK11A PE=1 SV=1
F8VU39;J3K	4	4	31.8438	7.31E-07	1.43E-06	4.01017	1	P-0.3PG-E8-	E8 blank	212876.5	Bromodomain adjacent to zinc finger domain protein 2A OS=Homo sapiens OX=9606 GN=BAZ2A PE=1 SV=1
A0A2R8Y59	4	4	20.5675	6.81E-08	2.09E-07	33.9667	1	P-0.3PG-E8-	E8 blank	74596.79	Transcriptional repressor CTCF OS=Homo sapiens OX=9606 GN=CTCF PE=1 SV=1
Q2PPJ7;H7	4	4	19.5453	0.0003976	0.000478	4.03431	1	P-0.3PG-E8-	E8 blank	213222.4	Ral GTPase-activating protein subunit alpha-2 OS=Homo sapiens OX=9606 GN=RALGAPA2 PE=1 SV=2
F8VZ81;Q9	4	4	20.2266	1.66E-07	4.06E-07	5.96078	1	P-0.3PG-E8-	E8 blank	72506.03	TBC1 domain family member 30 OS=Homo sapiens OX=9606 GN=TBC1D30 PE=1 SV=1
A0A096LNL	4	4	25.1983	7.35E-07	1.44E-06	12.6545	1	E8 blank	P-0.3PG-E8-	153317.2	DNA helicase (Fragment) OS=Homo sapiens OX=9606 GN=ATRX PE=1 SV=1

H0Y6W5;Q(	4	4	24.5103	1.91E-09	1.53E-08	8.34763	1	E8 blank	P-0.3PG-E8-	97604.6	Zinc finger protein basonuclin-2 (Fragment) OS=Homo sapiens OX=9606 GN=BNC2 PE=1 SV=1
A0A0A0MS	4	4	39.2386	2.67E-06	4.54E-06	6.80195	1	P-0.3PG-E8-	E8 blank	34654.11	Protein PRRC2C OS=Homo sapiens OX=9606 GN=PRRC2C PE=1 SV=1
Q5VTB9;Q5	5	4	31.3872	8.26E-09	4.17E-08	3.38	1	E8 blank	P-0.3PG-E8-	63791.87	E3 ubiquitin-protein ligase RNF220 OS=Homo sapiens OX=9606 GN=RNF220 PE=1 SV=1
Q86XL3	4	4	16.6868	4.81E-11	1.40E-09	59.6352	1	P-0.3PG-E8-	E8 blank	104969.9	Ankyrin repeat and LEM domain-containing protein 2 OS=Homo sapiens OX=9606 GN=ANKLE2 PE=1 SV=4
Q8TEY7	4	4	18.086	1.15E-06	2.09E-06	7.66899	1	E8 blank	P-0.3PG-E8-	108608.9	Ubiquitin carboxyl-terminal hydrolase 33 OS=Homo sapiens OX=9606 GN=USP33 PE=1 SV=2
A0A024R4E	5	4	25.4739	2.70E-07	6.19E-07	2.01422	1	P-0.3PG-E8-	E8 blank	142067.1	High density lipoprotein binding protein (Vigilin)_ isoform CRA_a OS=Homo sapiens OX=9606 GN=HDLBP PE=1
O00267;M(	4	4	18.5695	2.77E-05	3.88E-05	3.0968	1	F-0.3PG-E8-	E8 blank	121399	Transcription elongation factor SPT5 OS=Homo sapiens OX=9606 GN=SUPT5H PE=1 SV=1
O75197	6	4	31.5155	1.41E-07	3.58E-07	4.22321	1	E8 blank	P-0.3PG-E8-	182395.4	Low-density lipoprotein receptor-related protein 5 OS=Homo sapiens OX=9606 GN=LRP5 PE=1 SV=2
H0YJG4;A0	6	4	46.5485	3.95E-07	8.40E-07	1.64345	1	P-0.3PG-E8-	E8 blank	209392.2	Chromodomain-helicase-DNA-binding protein 8 (Fragment) OS=Homo sapiens OX=9606 GN=CHD8 PE=1 SV=2
P52756;C9J	6	4	33.4805	1.66E-07	4.06E-07	17.5586	1	P-0.3PG-E8-	E8 blank	92667.22	RNA-binding protein 5 OS=Homo sapiens OX=9606 GN=RBM5 PE=1 SV=2
Q13045;J3I	4	4	23.3184	1.18E-08	5.34E-08	14.0449	1	P-0.3PG-E8-	E8 blank	146234.1	Protein flightless-1 homolog OS=Homo sapiens OX=9606 GN=FLII PE=1 SV=2
Q86VI1;F5H	4	4	21.4182	2.22E-07	5.26E-07	4.01888	1	P-0.3PG-E8-	E8 blank	82247.92	Exocyst complex component 3-like protein OS=Homo sapiens OX=9606 GN=EXOC3L1 PE=1 SV=2
E9PMZ8;Q8	3	3	12.0999	3.32E-07	7.30E-07	6.3432	1	F-0.3PG-E8-	E8 blank	154238.2	T-lymphoma invasion and metastasis-inducing protein 2 (Fragment) OS=Homo sapiens OX=9606 GN=TIAM2 PE
J3QLK5;Q8(	3	3	21.9625	1.80E-08	7.27E-08	17.7416	1	P-0.3PG-E8-	E8 blank	70029.16	Serine/threonine-protein kinase tousled-like 2 OS=Homo sapiens OX=9606 GN=TLK2 PE=1 SV=1
Q12789	3	3	13.784	2.23E-06	3.82E-06	2.33759	1	P-0.3PG-E8-	E8 blank	241213.3	General transcription factor 3C polypeptide 1 OS=Homo sapiens OX=9606 GN=GTF3C1 PE=1 SV=4
Q9C0A0;A0	3	3	16.3692	4.24E-08	1.39E-07	23.2366	1	F-0.3PG-E8-	E8 blank	147270.5	Contactin-associated protein-like 4 OS=Homo sapiens OX=9606 GN=CNTNAP4 PE=1 SV=3
P02452	3	3	20.7725	3.22E-09	2.15E-08	4.40029	1	F-0.3PG-E8-	E8 blank	139968.1	Collagen alpha-1(I) chain OS=Homo sapiens OX=9606 GN=COL1A1 PE=1 SV=5
A0A087X1Z	3	3	13.7718	1.71E-09	1.43E-08	13.1342	1	P-0.3PG-E8-	E8 blank	119503.9	Gamma-tubulin complex component OS=Homo sapiens OX=9606 GN=TUBGCP5 PE=1 SV=1
Q96T23	3	3	12.7961	1.25E-11	8.49E-10	27.6232	1	P-0.3PG-E8-	E8 blank	165360.4	Remodeling and spacing factor 1 OS=Homo sapiens OX=9606 GN=RSF1 PE=1 SV=2
Q96GC6;M(	3	3	15.9727	2.37E-09	1.70E-08	14.9836	1	P-0.3PG-E8-	E8 blank	75318.05	Neurotrophin receptor-interacting factor homolog OS=Homo sapiens OX=9606 GN=ZNF274 PE=1 SV=2
A0A3B3IT2	3	3	17.5933	1.20E-07	3.21E-07	8.66218	1	P-0.3PG-E8-	E8 blank	194567.3	Kinesin-like protein KIF1A OS=Homo sapiens OX=9606 GN=KIF1A PE=1 SV=1
A0A1W2PC	4	3	33.8061	2.82E-06	4.76E-06	1.39282	1	F-0.3PG-E8-	E8 blank	86006.34	Sodium channel protein type 8 subunit alpha OS=Homo sapiens OX=9606 GN=SCN8A PE=1 SV=1
A0A590UJ5	3	3	29.0614	4.90E-07	1.00E-06	2.32318	1	F-0.3PG-E8-	E8 blank	101496	Low-density lipoprotein receptor-related protein 8 OS=Homo sapiens OX=9606 GN=LRP8 PE=1 SV=1
Q8TBY8	3	3	18.1792	8.47E-09	4.24E-08	4.41322	1	E8 blank	P-0.3PG-E8-	119033.8	Polyamine-modulated factor 1-binding protein 1 OS=Homo sapiens OX=9606 GN=PMFBP1 PE=2 SV=3
A0A3B3ITZ	3	3	23.1247	1.76E-12	2.30E-10	22.118	1	P-0.3PG-E8-	E8 blank	161873.6	DBF4-type zinc finger-containing protein 2 (Fragment) OS=Homo sapiens OX=9606 GN=ZDBF2 PE=1 SV=1
C9K0V9;Q9	3	3	23.1462	3.33E-12	2.91E-10	21.3577	1	F-0.3PG-E8-	E8 blank	78816.97	Ataxin-7-like protein 1 (Fragment) OS=Homo sapiens OX=9606 GN=ATXN7L1 PE=1 SV=1
Q9UGU0;A(	3	3	13.938	4.32E-06	7.02E-06	1.64177	1	E8 blank	P-0.3PG-E8-	213254.1	Transcription factor 20 OS=Homo sapiens OX=9606 GN=TCF20 PE=1 SV=3
H3BPJ7;A0	3	3	22.119	3.68E-07	7.91E-07	48.2106	1	E8 blank	P-0.3PG-E8-	68711.24	Transcription factor 4 OS=Homo sapiens OX=9606 GN=TCF4 PE=1 SV=1
A0A2R8Y5U	4	3	20.258	3.58E-05	4.91E-05	66.9705	1	P-0.3PG-E8-	E8 blank	160789.8	Polycomb group protein ASXL1 OS=Homo sapiens OX=9606 GN=ASXL1 PE=1 SV=1
H3BRA9;Q8	3	3	13.5376	1.50E-05	2.21E-05	2.05323	1	P-0.3PG-E8-	E8 blank	127809.3	A disintegrin and metalloproteinase with thrombospondin motifs 17 OS=Homo sapiens OX=9606 GN=ADAMTS
H0YBJ4;O0	3	3	13.1715	3.25E-05	4.50E-05	1.78012	1	E8 blank	P-0.3PG-E8-	83822.32	Matrilin-2 (Fragment) OS=Homo sapiens OX=9606 GN=MATN2 PE=1 SV=1
P55198	3	3	13.61	5.84E-07	1.17E-06	2.6019	1	E8 blank	F-0.3PG-E8-	113530.8	Protein AF-17 OS=Homo sapiens OX=9606 GN=MLLT6 PE=1 SV=3
Q96LW1	3	3	13.1195	0.0052255	0.005735	1.2963	0.9581	E8 blank	P-0.3PG-E8-	72411.33	Zinc finger protein 354B OS=Homo sapiens OX=9606 GN=ZNF354B PE=1 SV=1
K7EIE6;Q6V	4	3	24.9546	0.0019546	0.002221	1.21866	0.9939	P-0.3PG-E8-	E8 blank	49746.54	Zinc finger protein 562 OS=Homo sapiens OX=9606 GN=ZNF562 PE=1 SV=1
O95838;I3L	3	3	18.3981	3.10E-05	4.30E-05	2.13195	1	P-0.3PG-E8-	E8 blank	63913.98	Glucagon-like peptide 2 receptor OS=Homo sapiens OX=9606 GN=GLP2R PE=2 SV=1
O94892	3	3	16.9641	1.61E-06	2.84E-06	5.17913	1	P-0.3PG-E8-	E8 blank	76889.32	Zinc finger protein 432 OS=Homo sapiens OX=9606 GN=ZNF432 PE=1 SV=1
A0A0A0MR	3	3	12.5632	5.39E-06	8.51E-06	20.759	1	E8 blank	F-0.3PG-E8-	177866.5	MAP kinase-activating death domain protein OS=Homo sapiens OX=9606 GN=MADD PE=1 SV=1
Q9Y2H1	3	3	20.3606	0.0001895	0.000236	1.40025	1	E8 blank	P-0.3PG-E8-	54231.08	Serine/threonine-protein kinase 38-like OS=Homo sapiens OX=9606 GN=STK38L PE=1 SV=3
Q9P0W8;G	3	3	17.1253	0.0610884	0.06326	1.12342	0.545	E8 blank	F-0.3PG-E8-	68232.42	Spermatogenesis-associated protein 7 OS=Homo sapiens OX=9606 GN=SPATA7 PE=1 SV=3
Q9P266	3	3	22.5941	1.27E-05	1.88E-05	1.98441	1	F-0.3PG-E8-	E8 blank	149434.5	Junctional protein associated with coronary artery disease OS=Homo sapiens OX=9606 GN=JCAD PE=1 SV=3
A0A087WV	3	3	21.4863	0.0022362	0.002502	1.2301	0.9917	P-0.3PG-E8-	E8 blank	157011.2	Rho GTPase-activating protein 21 OS=Homo sapiens OX=9606 GN=ARHGAP21 PE=1 SV=1
Q9NUL3;E7	3	3	13.9606	4.44E-07	9.29E-07	4.39015	1	E8 blank	P-0.3PG-E8-	62836.7	Double-stranded RNA-binding protein Staufen homolog 2 OS=Homo sapiens OX=9606 GN=STAU2 PE=1 SV=2
O96028	3	3	12.8457	3.21E-08	1.12E-07	3.54463	1	E8 blank	F-0.3PG-E8-	156307.4	Histone-lysine N-methyltransferase NSD2 OS=Homo sapiens OX=9606 GN=NSD2 PE=1 SV=1
Q8NCN4;H(	3	3	14.1412	4.98E-08	1.60E-07	7.584	1	P-0.3PG-E8-	E8 blank	78277.92	E3 ubiquitin-protein ligase RNF169 OS=Homo sapiens OX=9606 GN=RNF169 PE=1 SV=2
A0A7P0T84	3	3	16.7342	0.0011574	0.001337	1.48036	0.9985	E8 blank	F-0.3PG-E8-	43353.94	A-kinase anchor protein 8-like OS=Homo sapiens OX=9606 GN=AKAP8L PE=4 SV=1
A0A7P0T96	3	3	15.4126	1.04E-09	1.01E-08	5.87514	1	E8 blank	F-0.3PG-E8-	190782.8	Androglobin OS=Homo sapiens OX=9606 GN=ADGB PE=4 SV=1
P08575	3	3	25.123	7.01E-08	2.14E-07	4.84743	1	F-0.3PG-E8-	E8 blank	148969.4	Receptor-type tyrosine-protein phosphatase C OS=Homo sapiens OX=9606 GN=PTPRC PE=1 SV=3
Q8NDX5	3	3	11.7731	0.001137	0.001318	1.34128	0.9986	E8 blank	F-0.3PG-E8-	106960.1	Polyhomeotic-like protein 3 OS=Homo sapiens OX=9606 GN=PHC3 PE=1 SV=1
Q9UPX8;A(	3	3	15.4213	1.08E-08	5.00E-08	6.42954	1	P-0.3PG-E8-	E8 blank	159335.5	SH3 and multiple ankyrin repeat domains protein 2 OS=Homo sapiens OX=9606 GN=SHANK2 PE=1 SV=3
A0A5F9ZHE	3	3	17.7328	3.73E-08	1.26E-07	4.68944	1	E8 blank	P-0.3PG-E8-	54538.96	DNA primase OS=Homo sapiens OX=9606 GN=PRIM1 PE=1 SV=1

Q96DT7	3	3	14.7745	2.00E-06	3.46E-06	3.50416	1	E8 blank	P-0.3PG-E8-	96034.69	Zinc finger and BTB domain-containing protein 10 OS=Homo sapiens OX=9606 GN=ZBTB10 PE=1 SV=2
A0A087WW	3	3	16.1059	1.63E-10	3.26E-09	36.6912	1	P-0.3PG-E8-	E8 blank	122371.3	DNA mismatch repair protein Msh6 OS=Homo sapiens OX=9606 GN=MSH6 PE=1 SV=1
Q13796;C9	3	3	15.1353	9.98E-09	4.74E-08	4.35946	1	P-0.3PG-E8-	E8 blank	177607.9	Protein Shroom2 OS=Homo sapiens OX=9606 GN=SHROOM2 PE=1 SV=1
D6RJB7;Q8	3	3	20.7416	3.14E-07	7.04E-07	2.72931	1	F-0.3PG-E8-	E8 blank	219731.4	Ankyrin repeat domain-containing protein 31 OS=Homo sapiens OX=9606 GN=ANKRD31 PE=4 SV=2
A0A3B3ISZ	3	3	17.5358	0.0012164	0.0014	1.20119	0.9983	F-0.3PG-E8-	E8 blank	117888.5	Signal peptide_ CUB and EGF-like domain-containing protein 2 OS=Homo sapiens OX=9606 GN=SCUBE2 PE=1 S
A8K0R7;H0	3	3	14.9987	6.37E-09	3.52E-08	8.67004	1	E8 blank	F-0.3PG-E8-	88624.16	Zinc finger protein 839 OS=Homo sapiens OX=9606 GN=ZNF839 PE=2 SV=1
Q96MT3;A0	3	3	18.0848	1.35E-06	2.43E-06	2.15941	1	P-0.3PG-E8-	E8 blank	96239.34	Prickle-like protein 1 OS=Homo sapiens OX=9606 GN=PRICKLE1 PE=1 SV=2
E7EPI0;Q9P	3	3	19.979	2.92E-05	4.08E-05	2.38371	1	F-0.3PG-E8-	E8 blank	150955.3	Inhibitor of Bruton tyrosine kinase OS=Homo sapiens OX=9606 GN=IBTK PE=1 SV=1
Q15650;H0	3	3	15.9491	0.000184	0.00023	1.47532	1	F-0.3PG-E8-	E8 blank	67001.71	Activating signal cointegrator 1 OS=Homo sapiens OX=9606 GN=TRIP4 PE=1 SV=4
H0Y9M2;A0	3	3	12.8826	3.00E-08	1.06E-07	9.40646	1	P-0.3PG-E8-	E8 blank	151736.3	Zinc finger protein 827 OS=Homo sapiens OX=9606 GN=ZNF827 PE=1 SV=2
Q8NFQ8	3	3	17.3897	1.67E-08	6.96E-08	3.64999	1	P-0.3PG-E8-	E8 blank	51491.5	Torsin-1A-interacting protein 2 OS=Homo sapiens OX=9606 GN=TOR1AIP2 PE=1 SV=1
A0A1W2PP	3	3	21.8789	0.000174	0.000218	1.31787	1	F-0.3PG-E8-	E8 blank	42108.2	Choline O-acetyltransferase OS=Homo sapiens OX=9606 GN=CHAT PE=1 SV=1
A0A0D9SFR	3	3	25.0572	4.72E-06	7.57E-06	4.75548	1	E8 blank	P-0.3PG-E8-	116091.4	Diacylglycerol kinase OS=Homo sapiens OX=9606 GN=DGKH PE=1 SV=1
Q99590;A0	3	3	12.6149	4.43E-11	1.40E-09	5.76396	1	P-0.3PG-E8-	E8 blank	166420.3	Protein SCAF11 OS=Homo sapiens OX=9606 GN=SCAF11 PE=1 SV=2
H0Y2P0;P1	3	3	24.5025	2.66E-06	4.54E-06	1.8942	1	P-0.3PG-E8-	E8 blank	31351.34	CD44 antigen (Fragment) OS=Homo sapiens OX=9606 GN=CD44 PE=1 SV=1
A0A1W2PP	3	3	21.3149	0.0004534	0.000542	1.25396	0.9999	F-0.3PG-E8-	P-0.3PG-E8-	36745.7	Lipoyl synthase (Fragment) OS=Homo sapiens OX=9606 GN=LIAS PE=1 SV=1
Q86XN6	3	3	15.657	1.13E-06	2.07E-06	2.28358	1	E8 blank	P-0.3PG-E8-	90681.6	Zinc finger protein 761 OS=Homo sapiens OX=9606 GN=ZNF761 PE=1 SV=3
A0A0A0MS	3	3	15.6338	2.63E-08	9.79E-08	8.43989	1	E8 blank	F-0.3PG-E8-	83145.8	Exocyst complex component 5 OS=Homo sapiens OX=9606 GN=EXOC5 PE=1 SV=1
H0Y626	3	3	22.0838	0.0042656	0.00469	1.61031	0.9701	E8 blank	P-0.3PG-E8-	109740	Uncharacterized protein OS=Homo sapiens OX=9606 PE=4 SV=2
G5E9X3;Q9	3	3	15.2529	1.75E-08	7.14E-08	2.43697	1	P-0.3PG-E8-	E8 blank	75401.82	Fibronectin type III domain containing 3A_ isoform CRA_f OS=Homo sapiens OX=9606 GN=FNDC3A PE=1 SV=1
Q8IZC7	3	3	15.6954	1.72E-07	4.20E-07	2.77937	1	P-0.3PG-E8-	E8 blank	51936.22	Zinc finger protein 101 OS=Homo sapiens OX=9606 GN=ZNF101 PE=1 SV=1
D9ZGF5	14	3	100.9078	0.003464	0.003822	1.24899	0.9795	E8 blank	P-0.3PG-E8-	17481.95	Fibroblast growth factor OS=Homo sapiens OX=9606 GN=FGF2 PE=3 SV=1
O15068;H7	4	3	27.2519	1.46E-09	1.30E-08	10.1802	1	E8 blank	P-0.3PG-E8-	129420.9	Guanine nucleotide exchange factor DBS OS=Homo sapiens OX=9606 GN=MCF2L PE=1 SV=2
E5RJ97;Q9U	4	3	19.265	7.26E-06	1.11E-05	2.45731	1	P-0.3PG-E8-	E8 blank	100766	Zinc finger transcription factor Trps1 OS=Homo sapiens OX=9606 GN=TRPS1 PE=1 SV=1
Q9P2N2	5	3	32.9428	2.37E-09	1.70E-08	21.352	1	P-0.3PG-E8-	E8 blank	82458.97	Rho GTPase-activating protein 28 OS=Homo sapiens OX=9606 GN=ARHGAP28 PE=1 SV=3
O14530;B8	4	3	19.8523	0.0026156	0.002911	1.15559	0.9883	F-0.3PG-E8-	E8 blank	26705.16	Thioredoxin domain-containing protein 9 OS=Homo sapiens OX=9606 GN=TXNDC9 PE=1 SV=2
Q03181	4	3	21.2721	1.97E-07	4.74E-07	2.09096	1	P-0.3PG-E8-	E8 blank	50701.91	Peroxisome proliferator-activated receptor delta OS=Homo sapiens OX=9606 GN=PPARD PE=1 SV=1
O60486;B4	5	3	33.5264	3.82E-07	8.16E-07	2.90786	1	F-0.3PG-E8-	E8 blank	178536.6	Plexin-C1 OS=Homo sapiens OX=9606 GN=PLXNC1 PE=1 SV=1
Q14839;A0	17	3	86.5254	1.05E-06	1.95E-06	12.8388	1	P-0.3PG-E8-	E8 blank	219544.7	Chromodomain-helicase-DNA-binding protein 4 OS=Homo sapiens OX=9606 GN=CHD4 PE=1 SV=2
Q5F1R6;A0	4	3	14.4205	8.29E-11	1.97E-09	13.8828	1	P-0.3PG-E8-	E8 blank	62484.21	DnaJ homolog subfamily C member 21 OS=Homo sapiens OX=9606 GN=DNAJC21 PE=1 SV=2
E9PDF6;O4	5	3	33.3174	1.18E-08	5.34E-08	12.7745	1	P-0.3PG-E8-	E8 blank	129507.6	Unconventional myosin-Ib OS=Homo sapiens OX=9606 GN=MYO1B PE=1 SV=1
A8MXP9;A0	5	3	34.9778	2.76E-07	6.28E-07	3.14203	1	P-0.3PG-E8-	E8 blank	100480.6	Matrin-3 OS=Homo sapiens OX=9606 GN=MATR3 PE=1 SV=1
Q96SE7;M0	5	3	25.2089	4.42E-06	7.15E-06	1.71342	1	E8 blank	P-0.3PG-E8-	98279.65	Zinc finger protein 347 OS=Homo sapiens OX=9606 GN=ZNF347 PE=1 SV=2
A0A0U1RR	5	3	22.6808	3.72E-08	1.26E-07	21.2176	1	F-0.3PG-E8-	E8 blank	217446.6	C-myc promoter-binding protein OS=Homo sapiens OX=9606 GN=DENND4A PE=1 SV=2
A0A0A0MS	5	3	32.4775	3.25E-08	1.12E-07	2.25385	1	E8 blank	P-0.3PG-E8-	44539.24	Immunoglobulin heavy constant gamma 1 (Fragment) OS=Homo sapiens OX=9606 GN=IGHG1 PE=1 SV=1
P35527;K7	4	3	28.1927	2.53E-05	3.58E-05	1.86175	1	E8 blank	F-0.3PG-E8-	62292.54	Keratin_ type I cytoskeletal 9 OS=Homo sapiens OX=9606 GN=KRT9 PE=1 SV=3
Q562F6	5	3	28.3273	5.01E-06	7.99E-06	2.22473	1	E8 blank	P-0.3PG-E8-	145879.9	Shugoshin 2 OS=Homo sapiens OX=9606 GN=SGO2 PE=1 SV=2
Q63HK3	4	3	19.4159	5.44E-09	3.18E-08	4.5674	1	E8 blank	P-0.3PG-E8-	112822.5	Zinc finger protein with KRAB and SCAN domains 2 OS=Homo sapiens OX=9606 GN=ZKSCAN2 PE=1 SV=2
Q12906;K7	5	3	36.3122	7.68E-06	1.17E-05	1.78577	1	E8 blank	P-0.3PG-E8-	95737.75	Interleukin enhancer-binding factor 3 OS=Homo sapiens OX=9606 GN=ILF3 PE=1 SV=3
Q06455	4	3	26.9281	2.64E-08	9.79E-08	4.59939	1	E8 blank	P-0.3PG-E8-	68364.94	Protein CBFA2T1 OS=Homo sapiens OX=9606 GN=RUNX1T1 PE=1 SV=2
Q9NZV7	5	3	27.5122	2.38E-07	5.57E-07	4.43003	1	E8 blank	P-0.3PG-E8-	62304.42	Zinc finger imprinted 2 OS=Homo sapiens OX=9606 GN=ZIM2 PE=1 SV=1
Q9Y4D1;G3	5	3	23.736	1.84E-08	7.36E-08	9.41606	1	P-0.3PG-E8-	E8 blank	124044	Disheveled-associated activator of morphogenesis 1 OS=Homo sapiens OX=9606 GN=DAAM1 PE=1 SV=2
Q13123	5	3	23.4436	0.0080315	0.008711	1.26315	0.9217	E8 blank	P-0.3PG-E8-	65716.43	Protein Red OS=Homo sapiens OX=9606 GN=IK PE=1 SV=3
Q6DN90;A0	5	3	29.2776	0.001034	0.001205	1.36363	0.9989	E8 blank	P-0.3PG-E8-	109170	IQ motif and SEC7 domain-containing protein 1 OS=Homo sapiens OX=9606 GN=IQSEC1 PE=1 SV=1
P01308;A6	4	3	45.195	1.36E-06	2.43E-06	4.66937	1	E8 blank	P-0.3PG-E8-	12323.09	Insulin OS=Homo sapiens OX=9606 GN=INS PE=1 SV=1
Q8IWE4	4	3	19.8702	1.78E-09	1.46E-08	4.78183	1	E8 blank	P-0.3PG-E8-	34861.65	DCN1-like protein 3 OS=Homo sapiens OX=9606 GN=DCUN1D3 PE=1 SV=1
Q9P219;G3	4	3	26.8344	8.26E-05	0.000108	1.69374	1	E8 blank	P-0.3PG-E8-	229371.1	Protein Daple OS=Homo sapiens OX=9606 GN=CCDC88C PE=1 SV=3
Q01826;C9	5	3	25.8655	2.18E-09	1.62E-08	3.74032	1	F-0.3PG-E8-	E8 blank	86299.32	DNA-binding protein SATB1 OS=Homo sapiens OX=9606 GN=SATB1 PE=1 SV=1
P13796;Q5	5	3	25.736	6.69E-06	1.04E-05	2.28042	1	F-0.3PG-E8-	E8 blank	70858.8	Plastin-2 OS=Homo sapiens OX=9606 GN=LCP1 PE=1 SV=6
Q8N141	4	3	20.4461	0.015288	0.016305	1.22943	0.8354	F-0.3PG-E8-	P-0.3PG-E8-	64231.86	Zinc finger protein 82 homolog OS=Homo sapiens OX=9606 GN=ZFP82 PE=1 SV=1

Q9H7D0;H	4	3	24.5463	0.0537386	0.055829	1.08589	0.575	F-0.3PG-E8-	P-0.3PG-E8-	216335.6	Dedicator of cytokinesis protein 5 OS=Homo sapiens OX=9606 GN=DOCK5 PE=1 SV=3
Q9BRK4;B1	4	3	20.0098	5.75E-05	7.76E-05	19.0961	1	E8 blank	F-0.3PG-E8-	73443.78	Leucine zipper putative tumor suppressor 2 OS=Homo sapiens OX=9606 GN=LZTS2 PE=1 SV=2
Q96SN8;A0	6	3	30.4217	8.06E-05	0.000106	1.57689	1	E8 blank	P-0.3PG-E8-	216577.6	CDK5 regulatory subunit-associated protein 2 OS=Homo sapiens OX=9606 GN=CDK5RAP2 PE=1 SV=5
A0A1B0GU	4	3	24.3264	0.0523182	0.05453	2.59378	0.5812	P-0.3PG-E8-	E8 blank	101870.1	Protein Aster-B OS=Homo sapiens OX=9606 GN=GRAMD1B PE=1 SV=1
Q6P158	4	3	14.4069	1.84E-05	2.66E-05	3.23967	1	E8 blank	P-0.3PG-E8-	157201.3	Putative ATP-dependent RNA helicase DHX57 OS=Homo sapiens OX=9606 GN=DHX57 PE=1 SV=2
E7EV71;Q1	4	3	30.0584	1.39E-06	2.49E-06	1.4609	1	F-0.3PG-E8-	E8 blank	156018.6	Latent-transforming growth factor beta-binding protein 1 OS=Homo sapiens OX=9606 GN=LTBP1 PE=1 SV=2
A0A0D9SF	4	3	31.0084	0.0606855	0.062944	1.05403	0.5466	E8 blank	P-0.3PG-E8-	56049.32	Zinc finger protein 302 OS=Homo sapiens OX=9606 GN=ZNF302 PE=1 SV=1
Q96MT7	4	3	24.1774	5.90E-10	7.17E-09	5.71202	1	P-0.3PG-E8-	E8 blank	215120.3	Cilia- and flagella-associated protein 44 OS=Homo sapiens OX=9606 GN=CFAP44 PE=1 SV=2
B1AHL2;P2	4	3	31.8264	3.74E-10	5.57E-09	3.42567	1	P-0.3PG-E8-	E8 blank	82492.33	Fibulin-1 OS=Homo sapiens OX=9606 GN=FBLN1 PE=1 SV=1
Q9P2E3	4	3	20.6487	9.90E-05	0.000129	1.41497	1	E8 blank	F-0.3PG-E8-	225245.4	NFX1-type zinc finger-containing protein 1 OS=Homo sapiens OX=9606 GN=ZNFX1 PE=2 SV=2
Q9GZX5	4	3	27.1022	2.11E-09	1.59E-08	2.84231	1	F-0.3PG-E8-	E8 blank	61323.05	Zinc finger protein 350 OS=Homo sapiens OX=9606 GN=ZNF350 PE=1 SV=3
G3V200;O7	6	3	30.8615	2.12E-08	8.30E-08	5.38491	1	F-0.3PG-E8-	E8 blank	142564.7	Liprin-alpha-2 OS=Homo sapiens OX=9606 GN=PPFIA2 PE=1 SV=2
Q13643	4	3	26.3613	0.135924	0.139181	1.11018	0.3627	E8 blank	F-0.3PG-E8-	33187.95	Four and a half LIM domains protein 3 OS=Homo sapiens OX=9606 GN=FHL3 PE=1 SV=4
Q9H8V3;C9	4	3	22.1159	4.71E-09	2.93E-08	6.77096	1	E8 blank	P-0.3PG-E8-	104531.5	Protein ECT2 OS=Homo sapiens OX=9606 GN=ECT2 PE=1 SV=4
Q8WVS4;H	4	3	27.362	0.0006301	0.000745	1.18518	0.9998	P-0.3PG-E8-	E8 blank	123369.3	Cytoplasmic dynein 2 intermediate chain 1 OS=Homo sapiens OX=9606 GN=DYNC2I1 PE=1 SV=3
Q9BXU1	2	2	10.9842	0.0151038	0.016136	1.24083	0.8374	F-0.3PG-E8-	E8 blank	116720.6	Serine/threonine-protein kinase 31 OS=Homo sapiens OX=9606 GN=STK31 PE=2 SV=2
H0YLG5;Q9	2	2	13.3315	0.0009315	0.001088	2.5347	0.9992	F-0.3PG-E8-	E8 blank	31491.84	Regulator of microtubule dynamics protein 3 (Fragment) OS=Homo sapiens OX=9606 GN=RMDN3 PE=1 SV=1
F8W7T7;P1	2	2	17.1354	7.06E-11	1.86E-09	78.774	1	P-0.3PG-E8-	E8 blank	73602.61	Zinc finger protein 44 OS=Homo sapiens OX=9606 GN=ZNF44 PE=1 SV=1
M0R208;Q	2	2	24.6722	2.48E-08	9.40E-08	14.6986	1	P-0.3PG-E8-	E8 blank	20682.82	ATP-dependent Clp protease proteolytic subunit OS=Homo sapiens OX=9606 GN=CLPP PE=1 SV=1
A0A6Q8PF	2	2	10.185	1.59E-09	1.39E-08	17.0784	1	P-0.3PG-E8-	E8 blank	98456.44	Centrosomal protein of 104 kDa OS=Homo sapiens OX=9606 GN=CEP104 PE=1 SV=1
E9PD25;O4	2	2	20.6955	1.10E-07	3.06E-07	10.8137	1	P-0.3PG-E8-	E8 blank	119616.9	Metalloendopeptidase OS=Homo sapiens OX=9606 GN=TLL1 PE=1 SV=1
A8MVX0;H	2	2	15.4439	0.0014775	0.001691	1.52857	0.997	E8 blank	F-0.3PG-E8-	95374.47	Rho guanine nucleotide exchange factor 33 OS=Homo sapiens OX=9606 GN=ARHGEF33 PE=2 SV=2
F5H6W4;Q	2	2	12.8119	6.14E-10	7.17E-09	19.8353	1	P-0.3PG-E8-	E8 blank	70750.25	E3 ubiquitin-protein ligase MARCHF7 OS=Homo sapiens OX=9606 GN=MARCHF7 PE=1 SV=1
Q7KZ85;J3	2	2	22.4688	7.46E-07	1.45E-06	6.46151	1	P-0.3PG-E8-	E8 blank	200327.6	Transcription elongation factor SPT6 OS=Homo sapiens OX=9606 GN=SUPT6H PE=1 SV=2
Q01581	2	2	13.7286	7.50E-05	9.91E-05	1.5341	1	F-0.3PG-E8-	E8 blank	57864	Hydroxymethylglutaryl-CoA synthase_ cytoplasmic OS=Homo sapiens OX=9606 GN=HMGCS1 PE=1 SV=2
Q8NBR6	2	2	10.5553	3.15E-10	5.05E-09	9.20811	1	F-0.3PG-E8-	E8 blank	67847.65	Ubiquitin carboxyl-terminal hydrolase MINDY-2 OS=Homo sapiens OX=9606 GN=MINDY2 PE=1 SV=2
C9IZD3;Q6	2	2	21.4518	0.0003624	0.000438	1.56692	1	E8 blank	F-0.3PG-E8-	24237.23	Rho GTPase-activating protein 17 (Fragment) OS=Homo sapiens OX=9606 GN=ARHGAP17 PE=1 SV=3
Q969W8	2	2	10.2261	3.66E-07	7.90E-07	23.5566	1	P-0.3PG-E8-	E8 blank	50416.88	Zinc finger protein 566 OS=Homo sapiens OX=9606 GN=ZNF566 PE=1 SV=1
Q13263;M	2	2	13.3655	1.32E-05	1.94E-05	1.28862	1	P-0.3PG-E8-	F-0.3PG-E8-	90317.64	Transcription intermediary factor 1-beta OS=Homo sapiens OX=9606 GN=TRIM28 PE=1 SV=5
A0A087WX	2	2	14.3715	3.85E-10	5.61E-09	11.2217	1	E8 blank	F-0.3PG-E8-	93393.24	Zinc finger protein 33B OS=Homo sapiens OX=9606 GN=ZNF33B PE=1 SV=1
Q9H116;Q5	2	2	20.0396	1.08E-06	2.00E-06	2.3978	1	F-0.3PG-E8-	E8 blank	82145.72	GDNF-inducible zinc finger protein 1 OS=Homo sapiens OX=9606 GN=GZF1 PE=1 SV=1
Q96CP6;M	2	2	17.1004	3.24E-11	1.22E-09	56.9635	1	P-0.3PG-E8-	E8 blank	81364.57	Protein Aster-A OS=Homo sapiens OX=9606 GN=GRAMD1A PE=1 SV=2
F8W6I7;A0	2	2	14.3571	5.49E-07	1.11E-06	3.57819	1	P-0.3PG-E8-	E8 blank	33269.3	Helix-destabilizing protein OS=Homo sapiens OX=9606 GN=HNRNPA1 PE=1 SV=2
P42285;H0	2	2	14.706	2.17E-10	4.00E-09	27.1091	1	P-0.3PG-E8-	E8 blank	118831.5	Exosome RNA helicase MTR4 OS=Homo sapiens OX=9606 GN=MTREX PE=1 SV=3
H7C1P9	2	2	9.3789	0.0017922	0.002044	3.85419	0.9951	F-0.3PG-E8-	E8 blank	109291.5	Titin (Fragment) OS=Homo sapiens OX=9606 GN=TTN PE=1 SV=1
A2RRD8;M	2	2	10.7986	1.26E-07	3.31E-07	1.7631	1	F-0.3PG-E8-	E8 blank	61151.35	Zinc finger protein 320 OS=Homo sapiens OX=9606 GN=ZNF320 PE=1 SV=1
A0A2R8Y54	2	2	9.9813	3.04E-08	1.07E-07	33.4172	1	P-0.3PG-E8-	E8 blank	205496.3	InaD-like protein OS=Homo sapiens OX=9606 GN=PATJ PE=1 SV=1
K7ER83	2	2	10.4089	1.53E-07	3.85E-07	3.16062	1	F-0.3PG-E8-	E8 blank	26715.76	Protein phosphatase 1 regulatory subunit 12C (Fragment) OS=Homo sapiens OX=9606 GN=PPP1R12C PE=1 SV=
A0A7I2RN4	2	2	16.7392	4.81E-08	1.55E-07	6.93486	1	P-0.3PG-E8-	E8 blank	43234.27	DnaJ homolog subfamily B member 5 OS=Homo sapiens OX=9606 GN=DNAJB5 PE=1 SV=1
Q5T089;B4	2	2	4.5468	0.0003525	0.000427	2.7702	1	F-0.3PG-E8-	E8 blank	54306.36	MORN repeat-containing protein 1 OS=Homo sapiens OX=9606 GN=MORN1 PE=2 SV=2
Q9ULM2;M	2	2	11.6242	7.18E-06	1.10E-05	1.91421	1	P-0.3PG-E8-	E8 blank	63393.39	Zinc finger protein 490 OS=Homo sapiens OX=9606 GN=ZNF490 PE=1 SV=2
A0A087X0Z	2	2	10.5327	0.0142459	0.01527	1.34339	0.8468	F-0.3PG-E8-	E8 blank	14261.99	Rho GTPase-activating protein 23 (Fragment) OS=Homo sapiens OX=9606 GN=ARHGAP23 PE=1 SV=1
A0A087WX	2	2	15.0718	3.79E-06	6.22E-06	4.97759	1	P-0.3PG-E8-	E8 blank	54736.48	Clathrin heavy chain 2 OS=Homo sapiens OX=9606 GN=CLTCL1 PE=1 SV=1
Q9H6S0;D6	2	2	9.3041	0.0002479	0.000306	1.79663	1	E8 blank	P-0.3PG-E8-	161674.4	3'-5' RNA helicase YTHDC2 OS=Homo sapiens OX=9606 GN=YTHDC2 PE=1 SV=2
Q9UDR5	2	2	20.3906	4.21E-11	1.40E-09	19.1753	1	P-0.3PG-E8-	E8 blank	102873.4	Alpha-aminoadipic semialdehyde synthase_ mitochondrial OS=Homo sapiens OX=9606 GN=AASS PE=1 SV=1
Q92630;A0	2	2	17.8686	1.56E-05	2.27E-05	5.37199	1	F-0.3PG-E8-	E8 blank	67165.01	Dual specificity tyrosine-phosphorylation-regulated kinase 2 OS=Homo sapiens OX=9606 GN=DYRK2 PE=1 SV=
O95219	2	2	16.2882	0.0003478	0.000422	10.3533	1	E8 blank	F-0.3PG-E8-	52194.06	Sorting nexin-4 OS=Homo sapiens OX=9606 GN=SNX4 PE=1 SV=1
Q96JB5;J3K	2	2	11.4853	4.83E-09	2.93E-08	217.072	1	P-0.3PG-E8-	E8 blank	57262.93	CDK5 regulatory subunit-associated protein 3 OS=Homo sapiens OX=9606 GN=CDK5RAP3 PE=1 SV=2
Q13751	2	2	12.698	1.09E-06	2.01E-06	1.69173	1	E8 blank	P-0.3PG-E8-	133450.6	Laminin subunit beta-3 OS=Homo sapiens OX=9606 GN=LAMB3 PE=1 SV=1
Q53F19;K7	2	2	9.6443	3.57E-05	4.90E-05	1.60281	1	F-0.3PG-E8-	E8 blank	70706.72	Nuclear cap-binding protein subunit 3 OS=Homo sapiens OX=9606 GN=NCBP3 PE=1 SV=2

A0A087WY	2	2	13.5747	8.09E-09	4.15E-08	4.68949	1	E8 blank	P-0.3PG-E8-	168525.3	Neuroblastoma breakpoint family member 26 OS=Homo sapiens OX=9606 GN=NBPF26 PE=4 SV=3
Q7Z3K6;A8	2	2	10.9357	6.04E-10	7.17E-09	Infinity	1	F-0.3PG-E8-	E8 blank	62063.95	Mesoderm induction early response protein 3 OS=Homo sapiens OX=9606 GN=MIER3 PE=1 SV=2
A0A087X1U	2	2	11.2112	1.31E-05	1.93E-05	261.822	1	E8 blank	F-0.3PG-E8-	83658.63	Arf-GAP with GTPase_ ANK repeat and PH domain-containing protein 1 OS=Homo sapiens OX=9606 GN=AGAP
P41182	2	2	10.1214	0.0029722	0.003296	5.59156	0.9848	F-0.3PG-E8-	E8 blank	80671.52	B-cell lymphoma 6 protein OS=Homo sapiens OX=9606 GN=BCL6 PE=1 SV=1
G5E9G7;Q9	2	2	8.6371	4.06E-08	1.35E-07	6.28241	1	P-0.3PG-E8-	E8 blank	186224.2	Neurexin 2_ isoform CRA_a OS=Homo sapiens OX=9606 GN=NRXN2 PE=1 SV=1
E7ESG2;Q9	2	2	15.3772	2.89E-05	4.04E-05	1.5623	1	E8 blank	P-0.3PG-E8-	145944	Claspin OS=Homo sapiens OX=9606 GN=CLSPN PE=1 SV=1
Q15029	2	2	9.561	0.0003355	0.000408	2.84574	1	E8 blank	P-0.3PG-E8-	110405.4	116 kDa U5 small nuclear ribonucleoprotein component OS=Homo sapiens OX=9606 GN=EFTUD2 PE=1 SV=1
A0A0C4DG	2	2	11.3069	0.0144632	0.015477	1.4506	0.8444	E8 blank	P-0.3PG-E8-	121109.8	Cell division cycle and apoptosis regulator protein 1 (Fragment) OS=Homo sapiens OX=9606 GN=CCAR1 PE=1 S
Q9H2L5;Q5	2	2	11.9648	2.47E-08	9.40E-08	2.37033	1	F-0.3PG-E8-	E8 blank	37147.48	Ras association domain-containing protein 4 OS=Homo sapiens OX=9606 GN=RASSF4 PE=1 SV=2
E9PEP6	2	2	9.2517	4.22E-06	6.86E-06	2.44687	1	F-0.3PG-E8-	E8 blank	102552.1	Neuropilin OS=Homo sapiens OX=9606 GN=NRP1 PE=1 SV=2
A0A0A0MR	2	2	14.8458	4.01E-06	6.54E-06	1.83249	1	E8 blank	F-0.3PG-E8-	85953.53	Actin-binding LIM protein 1 OS=Homo sapiens OX=9606 GN=ABLIM1 PE=1 SV=1
O75534	2	2	13.857	1.79E-05	2.58E-05	1.99693	1	E8 blank	P-0.3PG-E8-	89740.2	Cold shock domain-containing protein E1 OS=Homo sapiens OX=9606 GN=CSDE1 PE=1 SV=2
Q07890;G3	2	2	18.4668	4.76E-09	2.93E-08	7.26791	1	P-0.3PG-E8-	E8 blank	154348.1	Son of sevenless homolog 2 OS=Homo sapiens OX=9606 GN=SOS2 PE=1 SV=2
E9PCJ7	2	2	9.2043	9.90E-05	0.000129	2.44896	1	E8 blank	P-0.3PG-E8-	42067.99	Putative E3 ubiquitin-protein ligase UBR7 OS=Homo sapiens OX=9606 GN=UBR7 PE=1 SV=2
Q96AC1	2	2	9.4619	6.24E-05	8.31E-05	2.62867	1	E8 blank	F-0.3PG-E8-	78488	Fermitin family homolog 2 OS=Homo sapiens OX=9606 GN=FERMT2 PE=1 SV=1
Q9ULL1	2	2	10.86	1.54E-07	3.86E-07	3.55982	1	F-0.3PG-E8-	E8 blank	156637.3	Pleckstrin homology domain-containing family G member 1 OS=Homo sapiens OX=9606 GN=PLEKHG1 PE=1 SV
Q6NXS1;P4	2	2	9.9529	5.16E-07	1.05E-06	10.2941	1	E8 blank	F-0.3PG-E8-	23163.47	Protein phosphatase inhibitor 2 family member B OS=Homo sapiens OX=9606 GN=PPP1R2B PE=1 SV=2
E7ENM8;P	2	2	9.815	1.07E-07	3.00E-07	6.87341	1	P-0.3PG-E8-	E8 blank	77485.6	Tyrosine-protein kinase OS=Homo sapiens OX=9606 GN=FES PE=1 SV=1
Q14676	2	2	8.4751	2.93E-08	1.05E-07	6.30443	1	P-0.3PG-E8-	E8 blank	227806.9	Mediator of DNA damage checkpoint protein 1 OS=Homo sapiens OX=9606 GN=MDC1 PE=1 SV=3
A6NI28;H0	2	2	11.288	4.70E-06	7.54E-06	1.5248	1	E8 blank	P-0.3PG-E8-	99310.56	Rho GTPase-activating protein 42 OS=Homo sapiens OX=9606 GN=ARHGAP42 PE=1 SV=3
C9J240;E7E	2	2	11.1553	1.25E-05	1.86E-05	1.50542	1	E8 blank	F-0.3PG-E8-	125898.8	Anion exchange protein OS=Homo sapiens OX=9606 GN=SLC4A10 PE=1 SV=1
P17036;Q8	2	2	17.5413	2.68E-07	6.16E-07	1.98247	1	E8 blank	P-0.3PG-E8-	51885.42	Zinc finger protein 3 OS=Homo sapiens OX=9606 GN=ZNF3 PE=1 SV=3
M0R2P6;Q	2	2	8.0256	8.80E-06	1.33E-05	4.34592	1	P-0.3PG-E8-	E8 blank	74335.05	SH3KBP1 binding protein 1_ isoform CRA_c OS=Homo sapiens OX=9606 GN=SHKBP1 PE=1 SV=1
Q9NQ38	2	2	15.4474	6.58E-06	1.02E-05	1.3506	1	F-0.3PG-E8-	P-0.3PG-E8-	124421.5	Serine protease inhibitor Kazal-type 5 OS=Homo sapiens OX=9606 GN=SPINK5 PE=1 SV=2
Q8TF05;J3K	2	2	9.0758	4.75E-09	2.93E-08	15.0242	1	P-0.3PG-E8-	E8 blank	108429.9	Serine/threonine-protein phosphatase 4 regulatory subunit 1 OS=Homo sapiens OX=9606 GN=PPP4R1 PE=1 SV
G3V3E1;G3	2	2	10.0636	1.05E-06	1.95E-06	7.6475	1	F-0.3PG-E8-	E8 blank	96841.37	SKI family transcriptional corepressor 1 OS=Homo sapiens OX=9606 GN=SKOR1 PE=3 SV=1
Q8TEW0;Q	2	2	10.7239	0.1787252	0.181558	1.09051	0.3055	F-0.3PG-E8-	E8 blank	151936.6	Partitioning defective 3 homolog OS=Homo sapiens OX=9606 GN=PARD3 PE=1 SV=2
D6RCC7;Q9	2	2	15.4468	4.12E-07	8.71E-07	2.77663	1	E8 blank	P-0.3PG-E8-	125104	Protein FAM135A OS=Homo sapiens OX=9606 GN=FAM135A PE=1 SV=1
A0A0A0MR	2	2	21.6824	7.36E-09	3.90E-08	5.32644	1	E8 blank	P-0.3PG-E8-	74544.31	Nucleolar and coiled-body phosphoprotein 1 (Fragment) OS=Homo sapiens OX=9606 GN=NOLC1 PE=1 SV=1
A6PVD3;P5	2	2	15.7383	1.68E-13	1.08E-10	147.518	1	P-0.3PG-E8-	E8 blank	23472.27	Transcription factor SOX-10 (Fragment) OS=Homo sapiens OX=9606 GN=SOX10 PE=1 SV=1
A0A0G2JLG	2	2	14.6228	0.0057121	0.006259	1.17518	0.9519	P-0.3PG-E8-	F-0.3PG-E8-	113731.6	IQ motif and SEC7 domain-containing protein 3 (Fragment) OS=Homo sapiens OX=9606 GN=IQSEC3 PE=1 SV=1
P54277;Q3	2	2	9.939	2.79E-07	6.32E-07	4.87192	1	P-0.3PG-E8-	E8 blank	106686.1	PMS1 protein homolog 1 OS=Homo sapiens OX=9606 GN=PMS1 PE=1 SV=1
J3KT14	2	2	8.8767	1.05E-05	1.58E-05	1.27863	1	F-0.3PG-E8-	P-0.3PG-E8-	28563.26	THO complex subunit 1 OS=Homo sapiens OX=9606 GN=THOC1 PE=1 SV=1
O94925	2	2	14.4194	2.51E-07	5.83E-07	6.12817	1	F-0.3PG-E8-	E8 blank	74316.67	Glutaminase kidney isoform_ mitochondrial OS=Homo sapiens OX=9606 GN=GLS PE=1 SV=1
H7BYY2;H7	2	2	9.9307	3.38E-10	5.28E-09	8.98091	1	P-0.3PG-E8-	E8 blank	139860.1	BCL-6 corepressor (Fragment) OS=Homo sapiens OX=9606 GN=BCOR PE=1 SV=1
G5E9A6;P5	2	2	11.2609	2.38E-07	5.57E-07	3.0119	1	E8 blank	P-0.3PG-E8-	106057.7	Ubiquitin carboxyl-terminal hydrolase OS=Homo sapiens OX=9606 GN=USP11 PE=1 SV=1
Q8IWJ2;B8	4	2	19.4075	0.0007315	0.000859	1.79254	0.9997	P-0.3PG-E8-	E8 blank	196993.4	GRIP and coiled-coil domain-containing protein 2 OS=Homo sapiens OX=9606 GN=GCC2 PE=1 SV=4
Q96H12;X6	2	2	9.3788	7.10E-06	1.09E-05	2.39198	1	P-0.3PG-E8-	E8 blank	32705.35	Myb/SANT-like DNA-binding domain-containing protein 3 OS=Homo sapiens OX=9606 GN=MSANTD3 PE=1 SV=
P61224	2	2	9.8727	0.1258912	0.129114	1.36832	0.3793	F-0.3PG-E8-	P-0.3PG-E8-	21052.94	Ras-related protein Rap-1b OS=Homo sapiens OX=9606 GN=RAP1B PE=1 SV=1
J3KMZ8;Q9	2	2	13.8517	1.17E-06	2.11E-06	2.4908	1	E8 blank	P-0.3PG-E8-	46920.91	Zinc finger protein ubi-d4 OS=Homo sapiens OX=9606 GN=DPF2 PE=1 SV=1
P48751;F8	2	2	10.1767	1.95E-09	1.54E-08	11.8747	1	P-0.3PG-E8-	E8 blank	136247.3	Anion exchange protein 3 OS=Homo sapiens OX=9606 GN=SLC4A3 PE=1 SV=2
Q8WUB2;F	2	2	15.041	8.28E-06	1.26E-05	1.69795	1	E8 blank	P-0.3PG-E8-	31020.05	Protein FAM216A OS=Homo sapiens OX=9606 GN=FAM216A PE=2 SV=1
B7Z3H4;Q5	2	2	19.3566	1.53E-08	6.46E-08	3.3613	1	P-0.3PG-E8-	E8 blank	67242.3	Beta-transducin repeat containing isoform 4 OS=Homo sapiens OX=9606 GN=BTRC PE=1 SV=1
H0Y954	2	2	14.635	2.13E-06	3.67E-06	7.90198	1	E8 blank	F-0.3PG-E8-	19316.8	Transforming acidic coiled-coil-containing protein 2 (Fragment) OS=Homo sapiens OX=9606 GN=TACC2 PE=1 S
P08621	2	2	8.9538	1.16E-06	2.10E-06	4.45741	1	E8 blank	F-0.3PG-E8-	51613.78	U1 small nuclear ribonucleoprotein 70 kDa OS=Homo sapiens OX=9606 GN=SNRNP70 PE=1 SV=2
Q5JXC2	2	2	9.7656	6.12E-06	9.53E-06	5.36209	1	P-0.3PG-E8-	E8 blank	43394.4	Migration and invasion-inhibitory protein OS=Homo sapiens OX=9606 GN=MIIP PE=1 SV=3
B4DLR6;B4	2	2	9.8709	6.16E-07	1.23E-06	3.84401	1	P-0.3PG-E8-	E8 blank	35316.66	Protocadherin-18 OS=Homo sapiens OX=9606 GN=PCDH18 PE=1 SV=1
A0A087WS	3	2	15.976	5.05E-07	1.03E-06	4.67906	1	F-0.3PG-E8-	E8 blank	98240.14	Zinc finger protein 43 OS=Homo sapiens OX=9606 GN=ZNF43 PE=1 SV=1
H0Y6E7;Q9	3	2	14.9362	2.85E-06	4.79E-06	1.97256	1	F-0.3PG-E8-	E8 blank	31745.77	RNA-binding motif protein_ X chromosome (Fragment) OS=Homo sapiens OX=9606 GN=RBMX PE=1 SV=2
Q0D2K5	3	2	21.9415	2.80E-10	4.59E-09	7.68219	1	P-0.3PG-E8-	E8 blank	23057.31	Putative EGF-like and EMI domain-containing protein 1 OS=Homo sapiens OX=9606 GN=EGFEM1P PE=5 SV=1

Q6PG37;E9	4	2	29.8945	1.05E-07	2.97E-07	2.22715	1	F-0.3PG-E8-	E8 blank	76738.37	Zinc finger protein 790 OS=Homo sapiens OX=9606 GN=ZNF790 PE=2 SV=2
A0A0C4DGI	2	2	9.7895	2.02E-06	3.48E-06	5.16285	1	E8 blank	P-0.3PG-E8-	54155.02	Phosphatidylinositol 4-phosphate 3-kinase C2 domain-containing subunit alpha OS=Homo sapiens OX=9606 GN
Q86WZ6	3	2	27.0042	5.14E-07	1.05E-06	10.3776	1	P-0.3PG-E8-	E8 blank	94656.23	Zinc finger protein 227 OS=Homo sapiens OX=9606 GN=ZNF227 PE=1 SV=1
D6R9G8;Q9	2	2	10.3017	5.60E-06	8.82E-06	1.75371	1	F-0.3PG-E8-	E8 blank	94714.11	Synphilin-1 (Fragment) OS=Homo sapiens OX=9606 GN=SNCAIP PE=1 SV=2
Q9UBG0	2	2	10.0002	1.39E-07	3.55E-07	34.0978	1	F-0.3PG-E8-	E8 blank	169868.2	C-type mannose receptor 2 OS=Homo sapiens OX=9606 GN=MRC2 PE=1 SV=2
Q9H4L5	3	2	19.8719	0.1547448	0.157526	1.13988	0.3351	F-0.3PG-E8-	P-0.3PG-E8-	102193.2	Oxysterol-binding protein-related protein 3 OS=Homo sapiens OX=9606 GN=OSBPL3 PE=1 SV=1
Q8TDD1	2	2	8.9304	0.0004594	0.000548	2.569	0.9999	F-0.3PG-E8-	E8 blank	98880.09	ATP-dependent RNA helicase DDX54 OS=Homo sapiens OX=9606 GN=DDX54 PE=1 SV=2
P49916	5	2	31.7545	1.39E-08	6.08E-08	Infinity	1	P-0.3PG-E8-	E8 blank	114389.8	DNA ligase 3 OS=Homo sapiens OX=9606 GN=LIG3 PE=1 SV=2
K7EQ05;P2	2	2	10.1931	5.76E-06	9.02E-06	1.90541	1	P-0.3PG-E8-	E8 blank	50406.96	Progranulin OS=Homo sapiens OX=9606 GN=GRN PE=1 SV=1
A5PLK6;H3	4	2	21.2948	2.61E-07	6.04E-07	3.30186	1	F-0.3PG-E8-	E8 blank	126886	Regulator of G-protein signaling protein-like OS=Homo sapiens OX=9606 GN=RGSL1 PE=2 SV=1
Q9P2G1	3	2	15.0159	0.0001603	0.000202	1.32642	1	F-0.3PG-E8-	P-0.3PG-E8-	124111.8	Ankyrin repeat and IBR domain-containing protein 1 OS=Homo sapiens OX=9606 GN=ANKIB1 PE=1 SV=3
A0A0A0MR	2	2	9.379	8.56E-10	8.85E-09	9.53834	1	P-0.3PG-E8-	E8 blank	92983.68	Nucleosome-remodeling factor subunit BPTF OS=Homo sapiens OX=9606 GN=BPTF PE=1 SV=1
P46063	2	2	9.9168	1.17E-07	3.16E-07	39.839	1	P-0.3PG-E8-	E8 blank	74484	ATP-dependent DNA helicase Q1 OS=Homo sapiens OX=9606 GN=RECQL PE=1 SV=3
A0A7P0TAK	2	2	17.0237	8.51E-07	1.63E-06	10.0715	1	F-0.3PG-E8-	E8 blank	200049.9	Neurogenic locus notch homolog protein 1 OS=Homo sapiens OX=9606 GN=NOTCH1 PE=4 SV=1
H0YH68	2	2	10.9586	1.68E-05	2.43E-05	2.75013	1	P-0.3PG-E8-	E8 blank	21240.53	Serine/threonine-protein kinase WNK1 (Fragment) OS=Homo sapiens OX=9606 GN=WNK1 PE=1 SV=8
Q9HCM1	3	2	15.4177	4.45E-07	9.29E-07	2.88479	1	P-0.3PG-E8-	E8 blank	196682.5	Retroelement silencing factor 1 OS=Homo sapiens OX=9606 GN=RESF1 PE=1 SV=3
Q86X53;E5	3	2	18.9284	8.90E-09	4.36E-08	4.89194	1	E8 blank	P-0.3PG-E8-	49212.55	Glutamate-rich protein 1 OS=Homo sapiens OX=9606 GN=ERICH1 PE=1 SV=1
A0A0J9YW	3	2	22.7943	2.31E-05	3.29E-05	1.69892	1	P-0.3PG-E8-	E8 blank	36426.88	Proprotein convertase subtilisin/kexin type 6 OS=Homo sapiens OX=9606 GN=PCSK6 PE=1 SV=1
Q6NSJ2	2	2	9.2264	0.0508261	0.053148	1.16327	0.588	E8 blank	P-0.3PG-E8-	72653.22	Pleckstrin homology-like domain family B member 3 OS=Homo sapiens OX=9606 GN=PHLDB3 PE=1 SV=3
A0A1B0GTF	3	2	20.9363	1.00E-06	1.87E-06	5.46009	1	E8 blank	F-0.3PG-E8-	104585.4	Mitogen-activated protein kinase kinase kinase 19 (Fragment) OS=Homo sapiens OX=9606 GN=MAP3K19 PE=4
Q8N1I0;H0	4	2	21.6507	1.89E-06	3.30E-06	4.23143	1	P-0.3PG-E8-	E8 blank	227031.6	Dedicator of cytokinesis protein 4 OS=Homo sapiens OX=9606 GN=DOCK4 PE=1 SV=3
Q96E09	2	2	21.8466	5.75E-06	9.02E-06	2.25582	1	F-0.3PG-E8-	E8 blank	30814.27	PPP2R1A-PPP2R2A-interacting phosphatase regulator 1 OS=Homo sapiens OX=9606 GN=PABIR1 PE=1 SV=1
A0A6I8PIK4	2	2	9.9381	0.000494	0.000589	1.23152	0.9999	F-0.3PG-E8-	P-0.3PG-E8-	52923.55	DnaJ homolog subfamily C member 7 OS=Homo sapiens OX=9606 GN=DNAJC7 PE=1 SV=1
F8VVT9;J3K	4	2	20.4614	1.67E-08	6.96E-08	2.87114	1	P-0.3PG-E8-	E8 blank	125389.1	Arf-GAP with GTPase_ ANK repeat and PH domain-containing protein 2 OS=Homo sapiens OX=9606 GN=AGAP
A0A286YEY	4	2	22.94	6.80E-07	1.34E-06	1.6767	1	F-0.3PG-E8-	E8 blank	44547.17	Immunoglobulin heavy constant gamma 2 (Fragment) OS=Homo sapiens OX=9606 GN=IGHG2 PE=1 SV=1
P78563	2	2	10.1198	0.0033501	0.003702	1.28799	0.9808	F-0.3PG-E8-	E8 blank	81162.74	Double-stranded RNA-specific editase 1 OS=Homo sapiens OX=9606 GN=ADARB1 PE=1 SV=1
Q460N5;A0	3	2	19.0444	2.28E-09	1.68E-08	15.1076	1	P-0.3PG-E8-	E8 blank	204853.4	Protein mono-ADP-ribosyltransferase PARP14 OS=Homo sapiens OX=9606 GN=PARP14 PE=1 SV=3
Q96JM7;E9	4	2	29.8209	1.45E-06	2.57E-06	1.83746	1	E8 blank	P-0.3PG-E8-	89705.45	Lethal(3)malignant brain tumor-like protein 3 OS=Homo sapiens OX=9606 GN=L3MBTL3 PE=1 SV=2
Q13137;D6	3	2	13.8548	0.2370996	0.240096	1.11495	0.2507	E8 blank	P-0.3PG-E8-	52995.29	Calcium-binding and coiled-coil domain-containing protein 2 OS=Homo sapiens OX=9606 GN=CALCOCO2 PE=1
Q8TDI0;K7E	3	2	18.7469	1.08E-09	1.04E-08	32.7248	1	P-0.3PG-E8-	E8 blank	224646.7	Chromodomain-helicase-DNA-binding protein 5 OS=Homo sapiens OX=9606 GN=CHD5 PE=1 SV=1
P15172	3	2	15.1919	9.29E-08	2.69E-07	29.8028	1	P-0.3PG-E8-	E8 blank	35071.69	Myoblast determination protein 1 OS=Homo sapiens OX=9606 GN=MYOD1 PE=1 SV=3
Q9ULE3;F8	4	2	33.1358	6.28E-09	3.50E-08	5.82813	1	F-0.3PG-E8-	E8 blank	114765.4	DENN domain-containing protein 2A OS=Homo sapiens OX=9606 GN=DENND2A PE=1 SV=4
Q92620	3	2	19.3201	0.013784	0.0148	3.01867	0.8519	E8 blank	P-0.3PG-E8-	141358.4	Pre-mRNA-splicing factor ATP-dependent RNA helicase PRP16 OS=Homo sapiens OX=9606 GN=DHX38 PE=1 SV
A0A2R8Y7U	2	2	13.2711	1.08E-08	5.00E-08	4.72104	1	E8 blank	P-0.3PG-E8-	181377.2	GATOR complex protein DEPDC5 OS=Homo sapiens OX=9606 GN=DEPDC5 PE=1 SV=1
A0A7P0T8V	3	2	18.0748	0.0001306	0.000167	3.26624	1	F-0.3PG-E8-	E8 blank	70393.57	Oligophrenin-1 OS=Homo sapiens OX=9606 GN=OPHN1 PE=4 SV=1
A0A0A0MS	3	2	20.6002	7.25E-09	3.87E-08	4.83844	1	P-0.3PG-E8-	E8 blank	38137.16	Negative elongation factor E (Fragment) OS=Homo sapiens OX=9606 GN=NELFE PE=1 SV=6
O43719;Q5	2	2	9.703	1.27E-06	2.29E-06	30.2925	1	E8 blank	F-0.3PG-E8-	86423.16	HIV Tat-specific factor 1 OS=Homo sapiens OX=9606 GN=HTATSF1 PE=1 SV=1
Q7Z3V5	3	2	14.1299	1.16E-07	3.14E-07	1.72313	1	P-0.3PG-E8-	E8 blank	73244.74	Zinc finger protein 571 OS=Homo sapiens OX=9606 GN=ZNF571 PE=2 SV=3
K7ER00;K7E	3	2	28.7743	3.72E-10	5.57E-09	12.7247	1	P-0.3PG-E8-	E8 blank	62452.44	Phenylalanine--tRNA ligase OS=Homo sapiens OX=9606 GN=FARSA PE=1 SV=1
F5H630;P5	3	2	14.3683	4.35E-05	5.94E-05	3.90025	1	E8 blank	P-0.3PG-E8-	87666.55	Zinc finger protein 84 OS=Homo sapiens OX=9606 GN=ZNF84 PE=1 SV=1
H7C4C9;Q3	2	2	20.303	4.45E-06	7.17E-06	1.76137	1	E8 blank	P-0.3PG-E8-	70248.94	Protein FAM161A (Fragment) OS=Homo sapiens OX=9606 GN=FAM161A PE=1 SV=1
Q5JVG8;F5	2	2	11.2952	2.61E-08	9.79E-08	8.11414	1	F-0.3PG-E8-	E8 blank	53019.27	Zinc finger protein 506 OS=Homo sapiens OX=9606 GN=ZNF506 PE=2 SV=2
P52738	2	2	9.7383	1.37E-07	3.51E-07	2.81951	1	E8 blank	F-0.3PG-E8-	54364.84	Zinc finger protein 140 OS=Homo sapiens OX=9606 GN=ZNF140 PE=1 SV=2
K7EJD3;K7E	2	2	10.6077	3.45E-07	7.49E-07	2.98264	1	E8 blank	P-0.3PG-E8-	22622.07	Galectin-3-binding protein OS=Homo sapiens OX=9606 GN=LGALS3BP PE=1 SV=1
P41002	2	2	10.5151	0.0001358	0.000173	851.616	1	E8 blank	P-0.3PG-E8-	89464.77	Cyclin-F OS=Homo sapiens OX=9606 GN=CCNF PE=1 SV=2
Q8IVG5	6	2	39.0317	1.30E-07	3.38E-07	3.92031	1	P-0.3PG-E8-	E8 blank	186243.7	Sterile alpha motif domain-containing protein 9-like OS=Homo sapiens OX=9606 GN=SAMD9L PE=1 SV=2
E9PG71;P5	3	2	15.4985	5.14E-05	6.98E-05	2.66753	1	P-0.3PG-E8-	E8 blank	107370	Receptor protein-tyrosine kinase OS=Homo sapiens OX=9606 GN=EPHA4 PE=1 SV=1
Q08999	3	2	12.423	9.94E-07	1.87E-06	3.15881	1	P-0.3PG-E8-	E8 blank	129792.5	Retinoblastoma-like protein 2 OS=Homo sapiens OX=9606 GN=RBL2 PE=1 SV=3
A0A7P0T8C	3	2	17.0245	2.88E-09	1.94E-08	7.66609	1	F-0.3PG-E8-	E8 blank	71069.51	Sorting nexin-9 OS=Homo sapiens OX=9606 GN=SNX9 PE=4 SV=1
Q96NG8;A0	3	2	15.4546	5.18E-08	1.65E-07	3.56807	1	P-0.3PG-E8-	E8 blank	61866.4	Zinc finger protein 582 OS=Homo sapiens OX=9606 GN=ZNF582 PE=2 SV=1

Q96NB3	3	2	14.2494	2.29E-05	3.27E-05	2.1254	1	F-0.3PG-E8-	E8 blank	42169.82	Zinc finger protein 830 OS=Homo sapiens OX=9606 GN=ZNF830 PE=1 SV=2
A0A286YFJ	6	2	37.1111	1.13E-08	5.19E-08	5.0603	1	E8 blank	P-0.3PG-E8-	44459.01	Immunoglobulin heavy constant gamma 4 (Fragment) OS=Homo sapiens OX=9606 GN=IGHG4 PE=1 SV=1
Q7RTP6	6	2	33.9598	2.88E-07	6.50E-07	1.69628	1	P-0.3PG-E8-	E8 blank	225435.7	[F-actin]-monooxygenase MICAL3 OS=Homo sapiens OX=9606 GN=MICAL3 PE=1 SV=2
H0YLN8;Q9	3	2	13.7303	0.0020112	0.002274	1.46023	0.9935	F-0.3PG-E8-	E8 blank	214736.4	Non-specific serine/threonine protein kinase OS=Homo sapiens OX=9606 GN=TRPM7 PE=1 SV=1
B9EG95;O9	3	2	15.3572	5.77E-05	7.78E-05	5.40121	1	P-0.3PG-E8-	E8 blank	61243.2	ZNF682 protein OS=Homo sapiens OX=9606 GN=ZNF682 PE=1 SV=1
A0A7P0T7Z	3	2	16.2493	0.6171284	0.618093	1.17217	0.1026	P-0.3PG-E8-	E8 blank	136809.4	Jouberin OS=Homo sapiens OX=9606 GN=AHI1 PE=4 SV=1
Q6ZSZ6;H0	6	2	29.8065	0.0961919	0.09929	1.16321	0.4396	F-0.3PG-E8-	P-0.3PG-E8-	118885.9	Teashirt homolog 1 OS=Homo sapiens OX=9606 GN=TSHZ1 PE=1 SV=2
Q8TD23	3	2	19.1088	5.13E-06	8.16E-06	21.1057	1	F-0.3PG-E8-	E8 blank	68522.97	Zinc finger protein 675 OS=Homo sapiens OX=9606 GN=ZNF675 PE=1 SV=3
Q9Y2L6;E9	3	2	12.7756	1.25E-05	1.86E-05	4.88439	1	E8 blank	F-0.3PG-E8-	118902.4	FERM domain-containing protein 4B OS=Homo sapiens OX=9606 GN=FRMD4B PE=1 SV=4
Q13136;E9	2	2	9.7936	1.14E-07	3.11E-07	6.63114	1	P-0.3PG-E8-	E8 blank	136349.3	Liprin-alpha-1 OS=Homo sapiens OX=9606 GN=PPFIA1 PE=1 SV=1
C9J066;E9P	3	2	13.1784	1.49E-08	6.35E-08	21.1102	1	P-0.3PG-E8-	E8 blank	240189.4	Ninein OS=Homo sapiens OX=9606 GN=NIN PE=1 SV=1
E5RFZ5;E5	2	2	16.7682	1.23E-07	3.25E-07	82.9324	1	P-0.3PG-E8-	E8 blank	20173.66	Double-strand-break repair protein rad21 homolog OS=Homo sapiens OX=9606 GN=RAD21 PE=1 SV=1
A0A2R8Y57	4	2	35.2163	5.46E-09	3.18E-08	10.5681	1	E8 blank	P-0.3PG-E8-	87150.8	Band 4.1 OS=Homo sapiens OX=9606 GN=EPB41 PE=1 SV=1
P14868	3	2	24.5797	4.02E-07	8.54E-07	12.7105	1	P-0.3PG-E8-	E8 blank	57535.52	Aspartate--tRNA ligase_ cytoplasmic OS=Homo sapiens OX=9606 GN=DARS1 PE=1 SV=2
Q8N108	3	2	21.891	4.93E-10	6.73E-09	7.71882	1	F-0.3PG-E8-	E8 blank	58325.5	Mesoderm induction early response protein 1 OS=Homo sapiens OX=9606 GN=MIER1 PE=1 SV=2
E9PAQ1;P2	2	2	10.5169	5.40E-09	3.18E-08	22.4495	1	E8 blank	P-0.3PG-E8-	47485.54	Properdin OS=Homo sapiens OX=9606 GN=CFP PE=1 SV=1
Q2VY69	3	2	14.7242	1.55E-07	3.86E-07	6.30234	1	F-0.3PG-E8-	E8 blank	71243	Zinc finger protein 284 OS=Homo sapiens OX=9606 GN=ZNF284 PE=2 SV=1
Q13105	3	2	12.6331	7.32E-06	1.12E-05	42.9753	1	P-0.3PG-E8-	E8 blank	89809.56	Zinc finger and BTB domain-containing protein 17 OS=Homo sapiens OX=9606 GN=ZBTB17 PE=1 SV=3
A0A3B3IU3	3	2	13.5103	1.12E-07	3.09E-07	20.165	1	E8 blank	F-0.3PG-E8-	111469.9	Unconventional myosin-Ig OS=Homo sapiens OX=9606 GN=MYO1G PE=1 SV=1
A7E2Y1;A0	5	2	30.7824	6.74E-07	1.33E-06	7.36892	1	E8 blank	F-0.3PG-E8-	227099.8	Myosin-7B OS=Homo sapiens OX=9606 GN=MYH7B PE=1 SV=4
A6QL64;A0	7	2	47.1177	1.58E-07	3.92E-07	5.2153	1	E8 blank	P-0.3PG-E8-	216325.4	Ankyrin repeat domain-containing protein 36A OS=Homo sapiens OX=9606 GN=ANKRD36 PE=2 SV=4
A0A087WX	3	2	15.7928	5.98E-06	9.33E-06	1.61554	1	E8 blank	P-0.3PG-E8-	52629.95	Triacylglycerol lipase OS=Homo sapiens OX=9606 GN=PNLIPRP2 PE=1 SV=1
A0A087WU	3	2	24.7654	0.0058898	0.006443	1.17593	0.9496	F-0.3PG-E8-	P-0.3PG-E8-	65993.49	Zinc finger protein 714 OS=Homo sapiens OX=9606 GN=ZNF714 PE=1 SV=1
A0A6Q8PG	2	2	8.1555	3.18E-07	7.09E-07	7.57287	1	P-0.3PG-E8-	E8 blank	73210.86	Ligand-dependent corepressor (Fragment) OS=Homo sapiens OX=9606 GN=LCOR PE=1 SV=1
A0A7I2YQ9	3	2	15.2587	6.34E-11	1.77E-09	4.83567	1	P-0.3PG-E8-	E8 blank	67451.82	T-plasminogen activator OS=Homo sapiens OX=9606 GN=PLAT PE=1 SV=1
A0A590UJ8	2	2	10.9673	3.81E-06	6.23E-06	2.096	1	P-0.3PG-E8-	E8 blank	88398.27	Arginine-glutamic acid dipeptide repeats protein (Fragment) OS=Homo sapiens OX=9606 GN=RERE PE=1 SV=1
H7BXL6	3	2	14.3581	3.14E-05	4.35E-05	5.55607	1	F-0.3PG-E8-	E8 blank	89532.7	Otogelin-like protein (Fragment) OS=Homo sapiens OX=9606 GN=OTOGL PE=1 SV=1
A0A5F9ZHN	3	2	28.2874	0.1098018	0.112793	1.21292	0.4096	E8 blank	P-0.3PG-E8-	37579.5	L-lactate dehydrogenase OS=Homo sapiens OX=9606 GN=LDHB PE=1 SV=1
A8MWY0;C	3	2	13.4215	0.00217	0.00244	2.0463	0.9923	E8 blank	F-0.3PG-E8-	117207.2	Endosome/lysosome-associated apoptosis and autophagy regulator family member 2 OS=Homo sapiens OX=96
A0A087X2E	1	1	9.3642	1.83E-06	3.20E-06	26.266	1	P-0.3PG-E8-	E8 blank	61279.08	Baculoviral IAP repeat-containing protein 2 OS=Homo sapiens OX=9606 GN=BIRC2 PE=1 SV=1
B5BNW5;E	1	1	10.931	9.14E-07	1.74E-06	99.0735	1	F-0.3PG-E8-	E8 blank	25676.53	Calcium-activated potassium channel subunit beta-2 OS=Homo sapiens OX=9606 GN=KCNMB2 PE=2 SV=1
H0YJV5	1	1	4.3723	2.13E-07	5.09E-07	4.27676	1	P-0.3PG-E8-	E8 blank	10775.02	Kinectin (Fragment) OS=Homo sapiens OX=9606 GN=KTN1 PE=1 SV=1
E9PNR8;Q9	1	1	7.1594	1.09E-07	3.03E-07	6.9962	1	F-0.3PG-E8-	E8 blank	3563.811	Hypoxia-inducible factor 1-alpha inhibitor OS=Homo sapiens OX=9606 GN=HIF1AN PE=1 SV=1
H7BYC7	1	1	5.4644	0.0029971	0.003318	647.452	0.9845	E8 blank	F-0.3PG-E8-	15758.33	Neurexin-1-beta OS=Homo sapiens OX=9606 GN=NRXN1 PE=1 SV=2
F8VZ96;O6	1	1	9.942	0.001188	0.00137	2.14629	0.9984	E8 blank	P-0.3PG-E8-	13473.3	NUAK family SNF1-like kinase 1 (Fragment) OS=Homo sapiens OX=9606 GN=NUAK1 PE=1 SV=1
Q8NEP3	1	1	4.5415	7.94E-09	4.11E-08	15.1826	1	E8 blank	P-0.3PG-E8-	80881.64	Dynein axonemal assembly factor 1 OS=Homo sapiens OX=9606 GN=DNAAF1 PE=2 SV=5
H0Y5P0	1	1	5.1453	2.72E-07	6.21E-07	3.22686	1	P-0.3PG-E8-	E8 blank	81204.73	Forkhead-associated domain-containing protein 1 (Fragment) OS=Homo sapiens OX=9606 GN=FHAD1 PE=1 SV
A0A087WS	1	1	5.8888	7.62E-08	2.27E-07	10.137	1	P-0.3PG-E8-	E8 blank	18888.08	TIMM23B-AGAP6 readthrough (NMD candidate) OS=Homo sapiens OX=9606 GN=TIMM23B-AGAP6 PE=4 SV=2
B2RXH2	1	1	5.8962	1.05E-06	1.95E-06	21.1015	1	E8 blank	F-0.3PG-E8-	57488.68	Lysine-specific demethylase 4E OS=Homo sapiens OX=9606 GN=KDM4E PE=1 SV=1
Q8NDX1	1	1	10.5386	2.00E-09	1.55E-08	4.31342	1	P-0.3PG-E8-	E8 blank	117390	PH and SEC7 domain-containing protein 4 OS=Homo sapiens OX=9606 GN=PSD4 PE=1 SV=2
A0A7P0T87	1	1	11.9638	9.33E-05	0.000121	3.4938	1	E8 blank	P-0.3PG-E8-	119529.6	DNA damage-binding protein 1 OS=Homo sapiens OX=9606 GN=DDB1 PE=4 SV=1
H0YLK9	1	1	4.7293	3.26E-08	1.12E-07	5.0842	1	F-0.3PG-E8-	E8 blank	19871.03	E3 ubiquitin-protein ligase Arkadia (Fragment) OS=Homo sapiens OX=9606 GN=RNF111 PE=1 SV=1
O75526	1	1	8.1044	4.17E-07	8.79E-07	93.4799	1	P-0.3PG-E8-	E8 blank	42985.35	RNA-binding motif protein_ X-linked-like-2 OS=Homo sapiens OX=9606 GN=RBMXL2 PE=1 SV=3
H3BTI0;Q9	1	1	10.0588	2.01E-09	1.55E-08	27.7515	1	F-0.3PG-E8-	E8 blank	57273.4	Cysteine-rich secretory protein LCCL domain-containing 2 OS=Homo sapiens OX=9606 GN=CRISPLD2 PE=1 SV=
M0R0P8;M	1	1	10.3186	0.0001011	0.000131	3.96422	1	E8 blank	P-0.3PG-E8-	244997	Unconventional myosin-IXb OS=Homo sapiens OX=9606 GN=MYO9B PE=1 SV=1
C9JUF0;E7E	1	1	5.2691	6.24E-10	7.17E-09	351.524	1	E8 blank	P-0.3PG-E8-	6325.851	Eukaryotic initiation factor 4A-II (Fragment) OS=Homo sapiens OX=9606 GN=EIF4A2 PE=1 SV=1
A0A7I2V45	1	1	5.2329	5.70E-09	3.29E-08	8.94242	1	E8 blank	P-0.3PG-E8-	9233.405	Heterogeneous nuclear ribonucleoprotein A1 (Fragment) OS=Homo sapiens OX=9606 GN=HNRNPA1 PE=1 SV=
Q9UIE0	1	1	8.2296	1.23E-07	3.25E-07	13.9441	1	P-0.3PG-E8-	E8 blank	56310.02	Zinc finger protein 230 OS=Homo sapiens OX=9606 GN=ZNF230 PE=1 SV=3
G8JLA2	2	1	20.9332	2.17E-06	3.72E-06	1.65556	1	F-0.3PG-E8-	E8 blank	17260.39	Myosin light polypeptide 6 OS=Homo sapiens OX=9606 GN=MYL6 PE=1 SV=1
H0YBN0;Q8	1	1	11.2138	9.21E-09	4.46E-08	4.19333	1	P-0.3PG-E8-	E8 blank	19778.42	Vacuolar protein sorting-associated protein 37A (Fragment) OS=Homo sapiens OX=9606 GN=VPS37A PE=1 SV=

J3KT77;Q9U	1	1	4.5497	3.59E-05	4.91E-05	1.57537	1	F-0.3PG-E8-	P-0.3PG-E8-	19397.83	DARPP-32 OS=Homo sapiens OX=9606 GN=PPP1R1B PE=1 SV=1
Q8N3L3	1	1	5.9152	2.29E-10	4.00E-09	7.18509	1	P-0.3PG-E8-	E8 blank	77032.24	Beta-taxilin OS=Homo sapiens OX=9606 GN=TXLNB PE=1 SV=3
O95409	1	1	4.8449	1.04E-08	4.89E-08	32.9373	1	P-0.3PG-E8-	E8 blank	55633.25	Zinc finger protein ZIC 2 OS=Homo sapiens OX=9606 GN=ZIC2 PE=1 SV=2
H7C4K3	1	1	5.2135	1.16E-06	2.10E-06	27.2667	1	P-0.3PG-E8-	E8 blank	85139.68	Integrin beta OS=Homo sapiens OX=9606 GN=ITGB1 PE=1 SV=2
A0A5F9ZHV	1	1	6.278	0.7533287	0.753329	1.05187	0.0792	F-0.3PG-E8-	P-0.3PG-E8-	66369.49	AF4/FMR2 family member 3 (Fragment) OS=Homo sapiens OX=9606 GN=AFF3 PE=1 SV=1
A0A087X25	1	1	9.966	0.2288902	0.23215	1.08216	0.2572	P-0.3PG-E8-	E8 blank	87082.99	Zinc finger protein 615 OS=Homo sapiens OX=9606 GN=ZNF615 PE=1 SV=1
A0A0A0MS	1	1	4.5691	4.74E-11	1.40E-09	9.34406	1	P-0.3PG-E8-	E8 blank	74535.74	(E2-independent) E3 ubiquitin-conjugating enzyme FATS (Fragment) OS=Homo sapiens OX=9606 GN=C10orf90
Q08752	1	1	4.9733	1.60E-09	1.39E-08	46.9054	1	P-0.3PG-E8-	E8 blank	41162.82	Peptidyl-prolyl cis-trans isomerase D OS=Homo sapiens OX=9606 GN=PPID PE=1 SV=3
Q04771	1	1	5.0536	4.66E-09	2.93E-08	7.86265	1	E8 blank	P-0.3PG-E8-	58350.54	Activin receptor type-1 OS=Homo sapiens OX=9606 GN=ACVR1 PE=1 SV=1
P21675	1	1	5.8417	1.23E-07	3.25E-07	6.15537	1	E8 blank	F-0.3PG-E8-	214103.3	Transcription initiation factor TFIID subunit 1 OS=Homo sapiens OX=9606 GN=TAF1 PE=1 SV=2
B5MCV5	1	1	11.4562	4.21E-09	2.75E-08	20.7588	1	P-0.3PG-E8-	E8 blank	23362.76	Histone deacetylase 11 (Fragment) OS=Homo sapiens OX=9606 GN=HDAC11 PE=1 SV=1
A0A7P0T9L	1	1	5.6639	0.0002244	0.000278	2.02676	1	E8 blank	P-0.3PG-E8-	77863.84	Dystrobrevin alpha OS=Homo sapiens OX=9606 GN=DTNA PE=4 SV=1
F5GY68;F5H	1	1	4.951	8.25E-09	4.17E-08	5.25041	1	E8 blank	F-0.3PG-E8-	24827.75	Anaphase-promoting complex subunit 5 (Fragment) OS=Homo sapiens OX=9606 GN=ANAPC5 PE=1 SV=1
A0A2R8Y46	1	1	6.2438	7.83E-09	4.08E-08	56.6509	1	P-0.3PG-E8-	E8 blank	33500.69	Serine/threonine-protein kinase B-raf OS=Homo sapiens OX=9606 GN=BRAF PE=1 SV=1
A0A0A6YYE	1	1	3.9039	1.52E-05	2.22E-05	1.84294	1	E8 blank	P-0.3PG-E8-	57449.97	Collybistin OS=Homo sapiens OX=9606 GN=ARHGEF9 PE=1 SV=1
Q96NW4	1	1	9.92	2.05E-05	2.95E-05	1.71415	1	E8 blank	P-0.3PG-E8-	118809.1	Ankyrin repeat domain-containing protein 27 OS=Homo sapiens OX=9606 GN=ANKRD27 PE=1 SV=2
Q9BQY9	1	1	10.9598	3.42E-07	7.46E-07	32.0912	1	P-0.3PG-E8-	E8 blank	28127.12	Dysbindin domain-containing protein 2 OS=Homo sapiens OX=9606 GN=DBNDD2 PE=1 SV=3
H0Y9C6;Q7	1	1	11.7664	5.97E-08	1.86E-07	4.82058	1	E8 blank	P-0.3PG-E8-	17637.53	Acyl-CoA dehydrogenase family member 11 (Fragment) OS=Homo sapiens OX=9606 GN=ACAD11 PE=1 SV=1
A0A087WU	1	1	5.825	0.0026948	0.002994	2.23975	0.9876	E8 blank	P-0.3PG-E8-	16799.93	Transmembrane and coiled-coil domain-containing protein 3 (Fragment) OS=Homo sapiens OX=9606 GN=TMC
A0A087WY	1	1	5.5766	1.58E-07	3.92E-07	Infinity	1	F-0.3PG-E8-	E8 blank	27336.6	Laminin subunit alpha-5 (Fragment) OS=Homo sapiens OX=9606 GN=LAMA5 PE=1 SV=1
F5H0R1;Q9	1	1	4.3534	2.26E-07	5.32E-07	5.70837	1	P-0.3PG-E8-	E8 blank	99804.08	RNA-binding motif protein 21 OS=Homo sapiens OX=9606 GN=TUT1 PE=1 SV=1
H7BYF4;O9	1	1	4.9883	1.21E-08	5.43E-08	3.33312	1	P-0.3PG-E8-	E8 blank	13197.83	UBX domain-containing protein 7 (Fragment) OS=Homo sapiens OX=9606 GN=UBXN7 PE=1 SV=1
F8VPF5;F8V	1	1	10.898	0.0001322	0.000169	7.62872	1	E8 blank	F-0.3PG-E8-	16897.84	5'-AMP-activated protein kinase subunit gamma-1 (Fragment) OS=Homo sapiens OX=9606 GN=PRKAG1 PE=1 S
B4DFF3;F6T	1	1	5.6622	9.46E-10	9.47E-09	7.6013	1	E8 blank	P-0.3PG-E8-	42091.5	Battenin OS=Homo sapiens OX=9606 GN=CLN3 PE=1 SV=1
Q8NEP9	1	1	4.3381	0.0002343	0.00029	1.35061	1	F-0.3PG-E8-	P-0.3PG-E8-	75251.5	Zinc finger protein 555 OS=Homo sapiens OX=9606 GN=ZNF555 PE=1 SV=4
E9PPW0;Q9	1	1	4.5547	1.87E-07	4.52E-07	9.15227	1	P-0.3PG-E8-	E8 blank	19486.73	Transcription factor SOX-13 (Fragment) OS=Homo sapiens OX=9606 GN=SOX13 PE=1 SV=8
H0YML8;P7	1	1	4.712	0.0007319	0.000859	3.51887	0.9997	P-0.3PG-E8-	E8 blank	27650.82	Iroquois-class homeodomain protein IRX-5 OS=Homo sapiens OX=9606 GN=IRX5 PE=1 SV=1
Q8IYD1	1	1	9.8592	7.70E-10	8.11E-09	6.94182	1	F-0.3PG-E8-	E8 blank	69510.64	Eukaryotic peptide chain release factor GTP-binding subunit ERF3B OS=Homo sapiens OX=9606 GN=GSPT2 PE=
G3V126;Q9	1	1	3.7094	8.83E-09	4.35E-08	7.45076	1	E8 blank	F-0.3PG-E8-	52140.49	V-type proton ATPase subunit H OS=Homo sapiens OX=9606 GN=ATP6V1H PE=1 SV=1
D3DUJ3;K7	1	1	5.8714	1.26E-07	3.31E-07	5.07053	1	P-0.3PG-E8-	E8 blank	36740.17	Protein tyrosine phosphatase_ non-receptor type 2_ isoform CRA_d OS=Homo sapiens OX=9606 GN=PTPN2 PE
F8W978;Q9	1	1	5.2282	2.96E-08	1.05E-07	5.3501	1	P-0.3PG-E8-	E8 blank	52821.77	Cytochrome P450 4F11 OS=Homo sapiens OX=9606 GN=CYP4F11 PE=1 SV=1
Q99496	1	1	4.854	0.0001565	0.000198	1.53008	1	E8 blank	F-0.3PG-E8-	38111.66	E3 ubiquitin-protein ligase RING2 OS=Homo sapiens OX=9606 GN=RNF2 PE=1 SV=1
H3BS51;H3	1	1	10.4814	1.16E-07	3.14E-07	10.9053	1	P-0.3PG-E8-	E8 blank	70413.37	Sphingomyelin phosphodiesterase OS=Homo sapiens OX=9606 GN=SMPD3 PE=1 SV=1
U3KQ61	1	1	6.0121	0.0018029	0.002053	1.18362	0.995	E8 blank	P-0.3PG-E8-	3498.883	M-phase phosphoprotein 9 (Fragment) OS=Homo sapiens OX=9606 GN=MPHOSPH9 PE=4 SV=1
A0A7P0Q1I	1	1	4.8463	2.45E-07	5.71E-07	4.70076	1	E8 blank	P-0.3PG-E8-	149755.5	Zinc finger protein 423 OS=Homo sapiens OX=9606 GN=ZNF423 PE=4 SV=1
Q6UXG2	1	1	5.7511	2.01E-08	7.91E-08	12.6929	1	E8 blank	P-0.3PG-E8-	114518.2	Endosome/lysosome-associated apoptosis and autophagy regulator 1 OS=Homo sapiens OX=9606 GN=ELAPOR
F8W9S8;J3I	1	1	9.1276	9.64E-08	2.78E-07	3.80685	1	P-0.3PG-E8-	E8 blank	71106.35	Lethal(3)malignant brain tumor-like protein 4 OS=Homo sapiens OX=9606 GN=L3MBTL4 PE=1 SV=1
O95985	1	1	9.9846	1.42E-08	6.11E-08	3.59703	1	P-0.3PG-E8-	E8 blank	98601.08	DNA topoisomerase 3-beta-1 OS=Homo sapiens OX=9606 GN=TOP3B PE=1 SV=1
Q14974	1	1	8.7972	4.11E-08	1.36E-07	44.9224	1	F-0.3PG-E8-	E8 blank	98482	Importin subunit beta-1 OS=Homo sapiens OX=9606 GN=KPNB1 PE=1 SV=2
M0R2Q5	1	1	4.1089	2.60E-08	9.79E-08	10.2193	1	P-0.3PG-E8-	E8 blank	19537.98	Fibrillin-3 (Fragment) OS=Homo sapiens OX=9606 GN=FBN3 PE=4 SV=1
E7ENC7	1	1	6.4893	1.59E-07	3.93E-07	60.361	1	P-0.3PG-E8-	E8 blank	4127.974	Negative elongation factor E OS=Homo sapiens OX=9606 GN=NELFE PE=1 SV=1
Q96QB1	1	1	4.5094	0.0122628	0.013189	2.86445	0.8694	E8 blank	P-0.3PG-E8-	172302.5	Rho GTPase-activating protein 7 OS=Homo sapiens OX=9606 GN=DLC1 PE=1 SV=4
A0A1B0GX(	1	1	4.5769	2.89E-07	6.50E-07	5.11868	1	F-0.3PG-E8-	E8 blank	19512.64	Ubiquitin-conjugating enzyme E2 E3 (Fragment) OS=Homo sapiens OX=9606 GN=UBE2E3 PE=1 SV=1
Q63HQ2	1	1	5.516	1.10E-06	2.01E-06	2.58027	1	F-0.3PG-E8-	E8 blank	113038.6	Pikachurin OS=Homo sapiens OX=9606 GN=EGFLAM PE=1 SV=2
E7EX95;E9F	1	1	5.9613	0.0997654	0.102813	1.15275	0.4313	E8 blank	F-0.3PG-E8-	12942.23	LIM domain-binding protein 2 OS=Homo sapiens OX=9606 GN=LDB2 PE=1 SV=1
Q8IZS8	1	1	5.3937	6.03E-09	3.45E-08	Infinity	1	E8 blank	P-0.3PG-E8-	124152.1	Voltage-dependent calcium channel subunit alpha-2/delta-3 OS=Homo sapiens OX=9606 GN=CACNA2D3 PE=1
A0A6Q8PG	1	1	10.5474	1.29E-06	2.32E-06	6.59126	1	E8 blank	F-0.3PG-E8-	82860.86	Chloride channel protein OS=Homo sapiens OX=9606 GN=CLCN4 PE=1 SV=1
P40426	1	1	4.5022	0.0230431	0.024536	12.0917	0.761	P-0.3PG-E8-	E8 blank	47474.76	Pre-B-cell leukemia transcription factor 3 OS=Homo sapiens OX=9606 GN=PBX3 PE=1 SV=1
K7EQS5;K7I	1	1	5.509	0.0005147	0.000612	9.10777	0.9999	F-0.3PG-E8-	E8 blank	11396.07	CBP80/20-dependent translation initiation factor (Fragment) OS=Homo sapiens OX=9606 GN=CTIF PE=1 SV=1
D6RIA1	1	1	4.9923	0.1020227	0.10497	5.51961	0.4262	E8 blank	F-0.3PG-E8-	18270.46	E3 SUMO-protein ligase ZNF451 (Fragment) OS=Homo sapiens OX=9606 GN=ZNF451 PE=1 SV=1

H0YGT2;P2	1	1	5.6871	0.0012423	0.001427	228.883	0.9981	F-0.3PG-E8-	E8 blank	13499.21	Cation-dependent mannose-6-phosphate receptor (Fragment) OS=Homo sapiens OX=9606 GN=M6PR PE=1 SV=
A0A1W2PS	1	1	4.9987	2.00E-07	4.79E-07	Infinity	1	F-0.3PG-E8-	E8 blank	56827.66	Gap junction protein OS=Homo sapiens OX=9606 GN=GJA10 PE=1 SV=1
P61978;Q5	1	1	4.4119	1.12E-07	3.08E-07	5.93625	1	P-0.3PG-E8-	E8 blank	51261.48	Heterogeneous nuclear ribonucleoprotein K OS=Homo sapiens OX=9606 GN=HNRNPK PE=1 SV=1
H7C4F9	1	1	5.7572	2.62E-05	3.69E-05	679.63	1	F-0.3PG-E8-	E8 blank	9715.898	Protein disulfide-isomerase A5 (Fragment) OS=Homo sapiens OX=9606 GN=PDIA5 PE=1 SV=1
E9PI60;Q9U	1	1	5.1226	0.2945289	0.297312	1.34908	0.2119	E8 blank	F-0.3PG-E8-	125824	Bromodomain and PHD finger-containing protein 3 OS=Homo sapiens OX=9606 GN=BRPF3 PE=1 SV=1
A0A0U1RR	1	1	10.4818	3.36E-08	1.15E-07	19.156	1	P-0.3PG-E8-	E8 blank	18481.58	Histone H2A OS=Homo sapiens OX=9606 GN=hCG_2039566 PE=3 SV=1
E9PD53;Q9	1	1	5.3806	2.82E-06	4.76E-06	40.8678	1	E8 blank	F-0.3PG-E8-	145139.6	Structural maintenance of chromosomes protein OS=Homo sapiens OX=9606 GN=SMC4 PE=1 SV=1
A0A494C0H	1	1	5.467	0.000253	0.000311	6.10614	1	F-0.3PG-E8-	E8 blank	30730.5	Immunoglobulin superfamily member 1 (Fragment) OS=Homo sapiens OX=9606 GN=IGSF1 PE=1 SV=1
A0A087WZ	1	1	4.779	0.0011547	0.001336	1.54666	0.9985	E8 blank	P-0.3PG-E8-	51388.03	Cyclin-dependent kinase 16 OS=Homo sapiens OX=9606 GN=CDK16 PE=1 SV=1
Q9UM13	1	1	4.9753	0.0010489	0.00122	8.71807	0.9989	E8 blank	P-0.3PG-E8-	21423.25	Anaphase-promoting complex subunit 10 OS=Homo sapiens OX=9606 GN=ANAPC10 PE=1 SV=1
A0A5F9ZGP	1	1	6.0104	6.28E-07	1.25E-06	202.959	1	E8 blank	P-0.3PG-E8-	8557.355	Activating signal cointegrator 1 complex subunit 1 OS=Homo sapiens OX=9606 GN=ASCC1 PE=1 SV=1
P55347	1	1	11.0062	1.16E-07	3.14E-07	67.169	1	P-0.3PG-E8-	E8 blank	48005.92	Homeobox protein PKNOX1 OS=Homo sapiens OX=9606 GN=PKNOX1 PE=1 SV=3
D6RBF8	1	1	4.2795	0.0001143	0.000147	70.786	1	P-0.3PG-E8-	E8 blank	16762.4	SRSF protein kinase 1 (Fragment) OS=Homo sapiens OX=9606 GN=SRPK1 PE=1 SV=1
E5RG30	1	1	4.632	1.46E-08	6.24E-08	Infinity	1	P-0.3PG-E8-	E8 blank	7806.361	Sorting nexin-16 (Fragment) OS=Homo sapiens OX=9606 GN=SNX16 PE=1 SV=1
A0A5F9ZI2	1	1	4.6208	0.0020033	0.002269	38.0871	0.9936	F-0.3PG-E8-	E8 blank	127779.2	Guanine nucleotide exchange factor H1 OS=Homo sapiens OX=9606 GN=ARHGEF2 PE=1 SV=1
Q13064	1	1	4.3974	2.18E-07	5.19E-07	52.2089	1	E8 blank	P-0.3PG-E8-	56785.32	Probable E3 ubiquitin-protein ligase makorin-3 OS=Homo sapiens OX=9606 GN=MKRN3 PE=1 SV=1
B1AM48;Q	1	1	5.8687	8.38E-05	0.00011	22.1013	1	E8 blank	P-0.3PG-E8-	20900.48	ELAV-like protein (Fragment) OS=Homo sapiens OX=9606 GN=ELAVL2 PE=1 SV=1
B4DTC4;C9	1	1	5.0837	0.2860229	0.289181	3.12009	0.217	F-0.3PG-E8-	E8 blank	22113.16	Polyhomeotic-like protein 3 OS=Homo sapiens OX=9606 GN=PHC3 PE=1 SV=1
Q96EP0	1	1	5.1215	1.90E-06	3.31E-06	Infinity	1	P-0.3PG-E8-	E8 blank	122731.6	E3 ubiquitin-protein ligase RNF31 OS=Homo sapiens OX=9606 GN=RNF31 PE=1 SV=1
A0A5S8K7B	1	1	7.0335	0.0122386	0.013185	243.448	0.8697	E8 blank	F-0.3PG-E8-	123924.6	Protein CC2D2B OS=Homo sapiens OX=9606 GN=CC2D2B PE=4 SV=1
E7ETK1	1	1	4.7019	0.0009284	0.001086	3.44257	0.9992	F-0.3PG-E8-	P-0.3PG-E8-	19106.68	Enhancer of polycomb homolog (Fragment) OS=Homo sapiens OX=9606 GN=EPC2 PE=1 SV=1
A0A7P0T9B	1	1	5.1933	0.0017424	0.001991	2.21075	0.9954	F-0.3PG-E8-	P-0.3PG-E8-	204795.1	ADAMTS-like protein 1 (Fragment) OS=Homo sapiens OX=9606 GN=ADAMTSL1 PE=4 SV=1
M0R0C0	1	1	4.3507	0.0003629	0.000438	2.14543	1	F-0.3PG-E8-	P-0.3PG-E8-	63368.4	Endothelial zinc finger protein-induced by tumor necrosis factor alpha OS=Homo sapiens OX=9606 GN=ZNF71 I
F5GWS9	1	1	6.3301	0.0003641	0.000439	12.5841	1	E8 blank	P-0.3PG-E8-	8906.233	Oral cancer overexpressed 1 OS=Homo sapiens OX=9606 GN=LTO1 PE=1 SV=1
B3KUS5;Q7	1	1	5.5731	4.44E-06	7.17E-06	5.1448	1	F-0.3PG-E8-	E8 blank	55907.38	Ubiquitin carboxyl-terminal hydrolase OS=Homo sapiens OX=9606 GN=USP30 PE=1 SV=1
Q6AI08	1	1	4.1453	5.50E-05	7.44E-05	291.097	1	E8 blank	P-0.3PG-E8-	130492.3	HEAT repeat-containing protein 6 OS=Homo sapiens OX=9606 GN=HEATR6 PE=1 SV=1
F8VV52	1	1	3.9544	0.0333992	0.035328	1.45285	0.6833	P-0.3PG-E8-	E8 blank	58791.8	CCR4-NOT transcription complex subunit 2 (Fragment) OS=Homo sapiens OX=9606 GN=CNOT2 PE=1 SV=1
A0A669KAX	1	1	5.6455	0.0002875	0.000352	2.31111	1	F-0.3PG-E8-	P-0.3PG-E8-	9299.269	Aryl hydrocarbon receptor nuclear translocator-like protein 1 OS=Homo sapiens OX=9606 GN=ARNTL PE=4 SV=
O43763	1	1	6.0333	7.91E-05	0.000104	6.63334	1	E8 blank	P-0.3PG-E8-	30308.53	T-cell leukemia homeobox protein 2 OS=Homo sapiens OX=9606 GN=TLX2 PE=1 SV=2
Q9P2H3	1	1	3.4547	2.22E-10	4.00E-09	Infinity	1	P-0.3PG-E8-	E8 blank	88719.32	Intraflagellar transport protein 80 homolog OS=Homo sapiens OX=9606 GN=IFT80 PE=1 SV=3
C9J7L6;Q9H	1	1	5.6483	1.24E-12	2.30E-10	Infinity	1	E8 blank	P-0.3PG-E8-	11678.22	Actin-related protein 8 (Fragment) OS=Homo sapiens OX=9606 GN=ACTR8 PE=1 SV=1
A0A0C4DG	1	1	5.503	0.0023135	0.002579	2.68548	0.9911	E8 blank	P-0.3PG-E8-	4972.324	Protein crumbs homolog 1 (Fragment) OS=Homo sapiens OX=9606 GN=CRB1 PE=4 SV=1
F5H514;H0	1	1	4.7121	4.58E-09	2.93E-08	Infinity	1	P-0.3PG-E8-	E8 blank	91568.51	RAS guanyl-releasing protein 1 OS=Homo sapiens OX=9606 GN=RASGRP1 PE=1 SV=2
H0YJ91	1	1	4.4919	1.14E-09	1.08E-08	7129301	1	E8 blank	P-0.3PG-E8-	150692.7	Pecanex-like protein (Fragment) OS=Homo sapiens OX=9606 GN=PCNX1 PE=1 SV=1
H7C4V3	1	1	5.3893	1.43E-06	2.55E-06	21.7044	1	E8 blank	P-0.3PG-E8-	13160.01	Ski-like protein (Fragment) OS=Homo sapiens OX=9606 GN=SKIL PE=1 SV=1
B8ZZI7;Q6Z	1	1	5.2245	0.3152758	0.317754	1.08476	0.2003	F-0.3PG-E8-	E8 blank	129884.6	Cation channel sperm-associated protein subunit gamma OS=Homo sapiens OX=9606 GN=CATSPERG PE=1 SV=
A0A087WZ	1	1	5.2014	0.375308	0.377665	1.31078	0.1721	F-0.3PG-E8-	E8 blank	17368.96	WW domain-containing oxidoreductase OS=Homo sapiens OX=9606 GN=WWOX PE=1 SV=1
A0A3B3IRY	1	1	4.6525	5.81E-05	7.80E-05	1.62885	1	E8 blank	P-0.3PG-E8-	119572.9	Receptor protein-tyrosine kinase OS=Homo sapiens OX=9606 GN=EPHB1 PE=1 SV=1
Q99856	1	1	5.4271	1.42E-07	3.59E-07	48.231	1	E8 blank	F-0.3PG-E8-	62946.1	AT-rich interactive domain-containing protein 3A OS=Homo sapiens OX=9606 GN=ARID3A PE=1 SV=2
E9PC52;Q1	1	1	5.8093	3.63E-12	2.91E-10	482046	1	P-0.3PG-E8-	E8 blank	47280.37	Histone-binding protein RBBP7 OS=Homo sapiens OX=9606 GN=RBBP7 PE=1 SV=1
Q6S5L8	1	1	5.0136	7.48E-07	1.45E-06	4.20274	1	F-0.3PG-E8-	E8 blank	69868.59	SHC-transforming protein 4 OS=Homo sapiens OX=9606 GN=SHC4 PE=1 SV=1
O14990	1	1	5.1492	0.0004384	0.000525	2.66821	1	E8 blank	P-0.3PG-E8-	22773.78	Protein phosphatase inhibitor 2 family member C OS=Homo sapiens OX=9606 GN=PPP1R2C PE=1 SV=1
F8WDL7	1	1	10.5038	8.01E-07	1.54E-06	2.38323	1	F-0.3PG-E8-	E8 blank	12712.76	Nucleolus and neural progenitor protein OS=Homo sapiens OX=9606 GN=NEPRO PE=1 SV=1
H0YAA4	1	1	5.2142	3.99E-08	1.33E-07	60.6035	1	E8 blank	P-0.3PG-E8-	4149.739	Zinc finger FYVE domain-containing protein 16 (Fragment) OS=Homo sapiens OX=9606 GN=ZFYVE16 PE=4 SV=1
Q96NW7	1	1	8.263	7.98E-08	2.36E-07	26.694	1	F-0.3PG-E8-	E8 blank	173835.7	Leucine-rich repeat-containing protein 7 OS=Homo sapiens OX=9606 GN=LRRC7 PE=1 SV=1
Q9C0F3	1	1	4.9033	0.0012747	0.001462	4.74676	0.998	F-0.3PG-E8-	E8 blank	55702.65	Zinc finger protein 436 OS=Homo sapiens OX=9606 GN=ZNF436 PE=1 SV=2
Q9HCE3	1	1	5.3442	2.92E-06	4.90E-06	12.4862	1	P-0.3PG-E8-	E8 blank	144091.4	Zinc finger protein 532 OS=Homo sapiens OX=9606 GN=ZNF532 PE=1 SV=2
M9MMK7	1	1	9.8236	1.31E-07	3.39E-07	2.86257	1	E8 blank	P-0.3PG-E8-	51233.78	Dynein heavy chain 14_ axonemal OS=Homo sapiens OX=9606 GN=DNAH14 PE=1 SV=1
M0R1L6;Q8	1	1	5.1229	3.45E-06	5.75E-06	3.71233	1	F-0.3PG-E8-	E8 blank	23852.63	Zinc finger protein 563 (Fragment) OS=Homo sapiens OX=9606 GN=ZNF563 PE=1 SV=1
Q7Z340	1	1	4.3767	6.82E-08	2.09E-07	4.14636	1	E8 blank	F-0.3PG-E8-	79796.11	Zinc finger protein 551 OS=Homo sapiens OX=9606 GN=ZNF551 PE=1 SV=3

E9PLB6	1	1	6.4452	4.31E-10	6.00E-09	66.764	1	P-0.3PG-E8-	E8 blank	8830.837	DENN domain-containing protein 2B (Fragment) OS=Homo sapiens OX=9606 GN=DENND2B PE=1 SV=1
A0A590UJN	1	1	4.9776	3.76E-06	6.20E-06	1.95686	1	F-0.3PG-E8-	E8 blank	67810.38	Enhancer of polycomb homolog OS=Homo sapiens OX=9606 GN=EPC1 PE=1 SV=1
P49006	1	1	5.2578	1.78E-06	3.13E-06	10.9934	1	P-0.3PG-E8-	E8 blank	19585.84	MARCKS-related protein OS=Homo sapiens OX=9606 GN=MARCKSL1 PE=1 SV=2
B4DXZ6;E7I	1	1	4.4514	0.0001476	0.000188	1.5765	1	F-0.3PG-E8-	P-0.3PG-E8-	68669.49	Fragile X mental retardation syndrome-related protein 1 OS=Homo sapiens OX=9606 GN=FXR1 PE=1 SV=1
A0A6Q8PG	1	1	4.2058	7.70E-07	1.49E-06	3.91207	1	E8 blank	P-0.3PG-E8-	200999.3	Sodium channel protein OS=Homo sapiens OX=9606 GN=SCN11A PE=3 SV=1
C9JRQ0	1	1	12.6568	0.0002886	0.000352	6.41626	1	P-0.3PG-E8-	E8 blank	15922.68	Suppressor of tumorigenicity 7 protein (Fragment) OS=Homo sapiens OX=9606 GN=ST7 PE=1 SV=1
A8MWA4	1	1	4.3371	9.49E-07	1.79E-06	2.58956	1	F-0.3PG-E8-	E8 blank	35552.16	Putative zinc finger protein 705E OS=Homo sapiens OX=9606 GN=ZNF705E PE=3 SV=2
Q9NUZ1	1	1	5.1365	6.34E-07	1.25E-06	2.39993	1	E8 blank	F-0.3PG-E8-	62422.92	Acyl-coenzyme A oxidase-like protein OS=Homo sapiens OX=9606 GN=ACOXL PE=2 SV=3
O95197	1	1	4.5099	0.0426342	0.044874	1.68614	0.6285	F-0.3PG-E8-	E8 blank	113238.5	Reticulon-3 OS=Homo sapiens OX=9606 GN=RTN3 PE=1 SV=2
A0A494C0C	1	1	5.4749	0.0006275	0.000743	244.479	0.9998	P-0.3PG-E8-	E8 blank	58003.28	Sodium channel and clathrin linker 1 OS=Homo sapiens OX=9606 GN=SCLT1 PE=1 SV=1
K7EMR1	1	1	4.6131	5.04E-05	6.86E-05	107.132	1	P-0.3PG-E8-	E8 blank	19381.5	Progranulin (Fragment) OS=Homo sapiens OX=9606 GN=GRN PE=1 SV=1
P02652;V9C	1	1	11.3136	5.89E-06	9.21E-06	11.3582	1	E8 blank	F-0.3PG-E8-	11289.09	Apolipoprotein A-II OS=Homo sapiens OX=9606 GN=APOA2 PE=1 SV=1
Q9HCC9	1	1	4.7321	3.76E-07	8.07E-07	7.34765	1	F-0.3PG-E8-	E8 blank	98315.37	Lateral signaling target protein 2 homolog OS=Homo sapiens OX=9606 GN=ZFYVE28 PE=1 SV=3
Q9BX82	1	1	4.3748	0.0001938	0.000241	5.48787	1	P-0.3PG-E8-	E8 blank	75119.04	Zinc finger protein 471 OS=Homo sapiens OX=9606 GN=ZNF471 PE=2 SV=1
Q5R372	1	1	4.4798	0.0294352	0.031238	1.45889	0.7107	P-0.3PG-E8-	E8 blank	93425.53	Rab GTPase-activating protein 1-like OS=Homo sapiens OX=9606 GN=RABGAP1L PE=1 SV=1
Q08397	1	1	4.7259	2.62E-05	3.69E-05	5.40546	1	P-0.3PG-E8-	E8 blank	63794.14	Lysyl oxidase homolog 1 OS=Homo sapiens OX=9606 GN=LOXL1 PE=1 SV=2
B4DWF2	1	1	4.9075	2.34E-08	8.99E-08	9.95652	1	E8 blank	P-0.3PG-E8-	61458.48	Zinc finger protein Helios OS=Homo sapiens OX=9606 GN=IKZF2 PE=1 SV=1
Q8NFN8	1	1	4.5085	0.0001511	0.000192	3.65298	1	F-0.3PG-E8-	E8 blank	90465.89	Probable G-protein coupled receptor 156 OS=Homo sapiens OX=9606 GN=GPR156 PE=2 SV=2
B1AMX8;Q	1	1	4.4708	3.18E-07	7.09E-07	Infinity	1	F-0.3PG-E8-	E8 blank	22103.63	TNF receptor-associated factor 2 (Fragment) OS=Homo sapiens OX=9606 GN=TRAF2 PE=1 SV=1
A4QPE4	1	1	5.3434	1.61E-11	8.49E-10	Infinity	1	F-0.3PG-E8-	E8 blank	106180	SPTBN2 protein OS=Homo sapiens OX=9606 GN=SPTBN2 PE=1 SV=1
F8W6D1;Q	1	1	10.3436	1.00E-05	1.50E-05	7.5144	1	F-0.3PG-E8-	E8 blank	36893.01	E3 ubiquitin-protein ligase RNF216 OS=Homo sapiens OX=9606 GN=RNF216 PE=1 SV=1
C9JMU2	1	1	10.4872	2.81E-06	4.76E-06	34.2808	1	P-0.3PG-E8-	E8 blank	6658.126	WW domain-containing adapter protein with coiled-coil (Fragment) OS=Homo sapiens OX=9606 GN=WAC PE=
Q9Y6I4	1	1	10.7352	3.46E-06	5.75E-06	102.211	1	E8 blank	P-0.3PG-E8-	60493.9	Ubiquitin carboxyl-terminal hydrolase 3 OS=Homo sapiens OX=9606 GN=USP3 PE=1 SV=2
I3L0K7;I3L2	1	1	4.3032	1.53E-10	3.17E-09	6.94194	1	E8 blank	F-0.3PG-E8-	57448.06	Heat shock protein 75 kDa_ mitochondrial OS=Homo sapiens OX=9606 GN=TRAP1 PE=1 SV=1
C9JDL7;H0Y	1	1	5.1636	4.49E-07	9.34E-07	12.5382	1	P-0.3PG-E8-	E8 blank	10946.73	RING finger protein 215 (Fragment) OS=Homo sapiens OX=9606 GN=RNF215 PE=1 SV=8
A0A0C4DGI	1	1	4.5879	0.0004173	0.000501	276.689	1	P-0.3PG-E8-	E8 blank	32713.47	Sorting nexin-29 (Fragment) OS=Homo sapiens OX=9606 GN=SNX29 PE=1 SV=1
F5H2H9	1	1	5.491	7.99E-07	1.54E-06	9.67916	1	E8 blank	P-0.3PG-E8-	18421.19	Activating transcription factor 7-interacting protein 1 (Fragment) OS=Homo sapiens OX=9606 GN=ATF7IP PE=1
A0A1W2PP	1	1	5.4211	2.65E-09	1.83E-08	78.3388	1	P-0.3PG-E8-	E8 blank	46455.41	Cytoplasmic polyadenylated homeobox-like OS=Homo sapiens OX=9606 GN=CPHXL PE=3 SV=1
O75081	2	1	9.9109	4.37E-08	1.42E-07	12.5804	1	P-0.3PG-E8-	E8 blank	72161.6	Protein CBFA2T3 OS=Homo sapiens OX=9606 GN=CBFA2T3 PE=1 SV=2
Q8NA42;K7	2	1	14.1295	0.0516343	0.053905	1.50734	0.5843	P-0.3PG-E8-	F-0.3PG-E8-	56323.82	Zinc finger protein 383 OS=Homo sapiens OX=9606 GN=ZNF383 PE=1 SV=1
M0QZA6;M	2	1	11.6437	0.0435054	0.045716	1.09898	0.6239	E8 blank	P-0.3PG-E8-	13039.47	Zinc finger protein 415 (Fragment) OS=Homo sapiens OX=9606 GN=ZNF415 PE=1 SV=1
H0Y8C6;O0	2	1	8.8895	0.3963193	0.398183	1.27892	0.1637	P-0.3PG-E8-	E8 blank	125288.3	Importin-5 (Fragment) OS=Homo sapiens OX=9606 GN=IPO5 PE=1 SV=1
A0A7P0T8C	2	1	9.9007	4.75E-07	9.78E-07	10.77	1	E8 blank	P-0.3PG-E8-	54709.48	Protein disulfide-isomerase OS=Homo sapiens OX=9606 GN=P4HB PE=4 SV=1
O95235	2	1	15.1476	1.34E-08	5.87E-08	31.7698	1	P-0.3PG-E8-	E8 blank	101304.6	Kinesin-like protein KIF20A OS=Homo sapiens OX=9606 GN=KIF20A PE=1 SV=1
A0A2R8Y4V	2	1	9.5288	0.000696	0.00082	5.73255	0.9997	E8 blank	P-0.3PG-E8-	93093.8	Epidermal growth factor receptor kinase substrate 8 OS=Homo sapiens OX=9606 GN=EPS8 PE=1 SV=1
Q7Z3Y9	2	1	16.9213	3.30E-07	7.26E-07	7.77569	1	E8 blank	P-0.3PG-E8-	52652.07	Keratin_ type I cytoskeletal 26 OS=Homo sapiens OX=9606 GN=KRT26 PE=1 SV=2
B0QZ19	1	1	6.1892	6.04E-05	8.06E-05	Infinity	1	E8 blank	P-0.3PG-E8-	7613.959	PHD finger protein 20 (Fragment) OS=Homo sapiens OX=9606 GN=PHF20 PE=4 SV=1
Q9BTA9;C9	2	1	9.7715	7.48E-05	9.90E-05	4.52457	1	F-0.3PG-E8-	E8 blank	71066.53	WW domain-containing adapter protein with coiled-coil OS=Homo sapiens OX=9606 GN=WAC PE=1 SV=3
F8WA39;Q	2	1	9.3883	0.0361346	0.038159	1.1522	0.6659	F-0.3PG-E8-	P-0.3PG-E8-	76459.46	Phosphatidylinositol-3_5-bisphosphate 3-phosphatase OS=Homo sapiens OX=9606 GN=MTMR1 PE=1 SV=1
O94782	2	1	9.4114	0.000104	0.000134	14.3534	1	P-0.3PG-E8-	E8 blank	89233.84	Ubiquitin carboxyl-terminal hydrolase 1 OS=Homo sapiens OX=9606 GN=USP1 PE=1 SV=1
B5MCT2;C9	2	1	16.2549	6.61E-10	7.30E-09	9.65299	1	P-0.3PG-E8-	E8 blank	57194.88	ETS translocation variant 1 OS=Homo sapiens OX=9606 GN=ETV1 PE=1 SV=2
A0A494C0U	2	1	12.1841	2.68E-07	6.16E-07	5.32449	1	F-0.3PG-E8-	E8 blank	131786.6	Tripeptidyl-peptidase 2 (Fragment) OS=Homo sapiens OX=9606 GN=TPP2 PE=1 SV=1
A0A494C04	2	1	12.3712	8.41E-07	1.61E-06	7.22191	1	E8 blank	F-0.3PG-E8-	84505.86	Serine/threonine-protein kinase TBK1 OS=Homo sapiens OX=9606 GN=TBK1 PE=1 SV=1
H3BUL7	2	1	11.0298	3.53E-06	5.85E-06	2.36403	1	E8 blank	P-0.3PG-E8-	2368.567	E3 SUMO-protein ligase PIAS1 OS=Homo sapiens OX=9606 GN=PIAS1 PE=4 SV=1
A0A0A0MT	2	1	18.5645	1.27E-09	1.17E-08	65.9994	1	P-0.3PG-E8-	E8 blank	48313.24	NADPH:adrenodoxin oxidoreductase_ mitochondrial OS=Homo sapiens OX=9606 GN=FDXR PE=1 SV=1
A0A087X0V	2	1	11.4896	7.41E-08	2.24E-07	16.8852	1	P-0.3PG-E8-	E8 blank	75087.83	2'-5' oligoadenylate synthase (Fragment) OS=Homo sapiens OX=9606 GN=OAS2 PE=1 SV=2
P33241	2	1	20.2082	1.30E-10	2.86E-09	181.149	1	P-0.3PG-E8-	E8 blank	37419.73	Lymphocyte-specific protein 1 OS=Homo sapiens OX=9606 GN=LSP1 PE=1 SV=1
A0A7P0Z49	2	1	15.0995	0.006435	0.007015	1.21141	0.9425	P-0.3PG-E8-	E8 blank	71975.4	E3 ubiquitin-protein ligase NEDD4-like (Fragment) OS=Homo sapiens OX=9606 GN=NEDD4L PE=4 SV=1
H0Y519	2	1	15.9397	1.56E-05	2.27E-05	2.40011	1	E8 blank	F-0.3PG-E8-	9338.177	Guanine nucleotide exchange factor DBS (Fragment) OS=Homo sapiens OX=9606 GN=MCF2L PE=1 SV=1
H3BUY5	2	1	10.0712	1.35E-07	3.46E-07	52.8318	1	P-0.3PG-E8-	E8 blank	28745.53	RNA-binding motif protein_ X chromosome OS=Homo sapiens OX=9606 GN=RBMX PE=1 SV=1

M0R283	2	1	11.8622	5.18E-05	7.02E-05	2.77888	1	E8 blank	P-0.3PG-E8-	13106.76	RAC-beta serine/threonine-protein kinase (Fragment) OS=Homo sapiens OX=9606 GN=AKT2 PE=1 SV=1
Q9BZF3	2	1	10.3468	0.0079928	0.008684	47.6677	0.9222	P-0.3PG-E8-	E8 blank	107446.7	Oxysterol-binding protein-related protein 6 OS=Homo sapiens OX=9606 GN=OSBPL6 PE=1 SV=1
Q14112;A0	2	1	9.3342	4.98E-09	2.98E-08	15.6202	1	F-0.3PG-E8-	E8 blank	154048.2	Nidogen-2 OS=Homo sapiens OX=9606 GN=NID2 PE=1 SV=3
H7BYU6;J3I	2	1	9.9863	7.14E-06	1.10E-05	12.1828	1	F-0.3PG-E8-	E8 blank	143940.2	Zinc finger protein 521 OS=Homo sapiens OX=9606 GN=ZNF521 PE=1 SV=2
A0A0C3SFZ	1	1	10.5028	5.81E-07	1.17E-06	4.1023	1	P-0.3PG-E8-	E8 blank	119887.2	Histone-lysine N-methyltransferase MECOM OS=Homo sapiens OX=9606 GN=MECOM PE=1 SV=1
O60290	2	1	14.7621	6.87E-06	1.06E-05	58.9284	1	E8 blank	P-0.3PG-E8-	133422.3	Zinc finger protein 862 OS=Homo sapiens OX=9606 GN=ZNF862 PE=2 SV=2
F5H026;M0	2	1	16.8992	0.0040018	0.004407	1.32693	0.9733	F-0.3PG-E8-	E8 blank	14551.66	Zinc finger protein 880 OS=Homo sapiens OX=9606 GN=ZNF880 PE=4 SV=1
A0A1B0GV	2	1	20.1852	1.70E-08	7.00E-08	10.6807	1	P-0.3PG-E8-	E8 blank	141616.7	HECT-type E3 ubiquitin transferase OS=Homo sapiens OX=9606 GN=NEDD4L PE=1 SV=1
A0A7I2V2J	2	1	9.4057	2.90E-08	1.04E-07	Infinity	1	P-0.3PG-E8-	E8 blank	131723	Laminin subunit beta-1 OS=Homo sapiens OX=9606 GN=LAMB1 PE=1 SV=1
F1T0E5;Q9	2	1	9.7061	1.28E-07	3.34E-07	8.17776	1	P-0.3PG-E8-	E8 blank	153085.3	Calcium-dependent secretion activator 1 OS=Homo sapiens OX=9606 GN=CADPS PE=1 SV=1
Q9NUC0	2	1	15.09	0.0002707	0.000332	6.19298	1	F-0.3PG-E8-	P-0.3PG-E8-	40089.54	SERTA domain-containing protein 4 OS=Homo sapiens OX=9606 GN=SERTAD4 PE=1 SV=1
Q9UBZ9	2	1	8.5983	7.64E-08	2.27E-07	3.98618	1	P-0.3PG-E8-	E8 blank	139445.8	DNA repair protein REV1 OS=Homo sapiens OX=9606 GN=REV1 PE=1 SV=1
A0A087WS	2	1	11.4755	1.13E-05	1.68E-05	3.06136	1	F-0.3PG-E8-	E8 blank	60820.38	Thioredoxin-disulfide reductase OS=Homo sapiens OX=9606 GN=TXNRD1 PE=1 SV=1
Q9UHB9	2	1	13.9876	2.43E-10	4.09E-09	11.0265	1	P-0.3PG-E8-	E8 blank	71242.96	Signal recognition particle subunit SRP68 OS=Homo sapiens OX=9606 GN=SRP68 PE=1 SV=2
D2WEZ3	2	1	12.6116	6.01E-05	8.05E-05	Infinity	1	F-0.3PG-E8-	E8 blank	99703.08	Neurogenic locus notch homolog protein 2 OS=Homo sapiens OX=9606 GN=NOTCH2 PE=1 SV=1
P22735	2	1	14.1758	0.0365708	0.038556	1.84052	0.6632	E8 blank	P-0.3PG-E8-	90585.24	Protein-glutamine gamma-glutamyltransferase K OS=Homo sapiens OX=9606 GN=TGM1 PE=1 SV=4
H3BRW8;Q	2	1	9.752	0.0476621	0.049921	1.37159	0.6029	F-0.3PG-E8-	P-0.3PG-E8-	25188.52	Calcium-binding and coiled-coil domain-containing protein 1 (Fragment) OS=Homo sapiens OX=9606 GN=CALC
Q9ULG6;H3	2	1	25.4804	4.01E-10	5.71E-09	119.209	1	P-0.3PG-E8-	E8 blank	88081.83	Cell cycle progression protein 1 OS=Homo sapiens OX=9606 GN=CCPG1 PE=1 SV=3
Q9UKI8;B3	2	1	14.1661	6.17E-09	3.47E-08	3.36382	1	E8 blank	P-0.3PG-E8-	87270.03	Serine/threonine-protein kinase tousled-like 1 OS=Homo sapiens OX=9606 GN=TLK1 PE=1 SV=2
Q96ST3;H3	3	1	21.2325	8.66E-09	4.31E-08	8.10087	1	P-0.3PG-E8-	E8 blank	145973.7	Paired amphipathic helix protein Sin3a OS=Homo sapiens OX=9606 GN=SIN3A PE=1 SV=2
A0A7I2YQJ	3	1	22.039	1.02E-07	2.91E-07	2.90614	1	E8 blank	P-0.3PG-E8-	9290.105	Heterogeneous nuclear ribonucleoprotein A1 (Fragment) OS=Homo sapiens OX=9606 GN=HNRNPA1 PE=1 SV=
A0A0C4DF	3	1	18.629	4.88E-07	1.00E-06	60.6846	1	P-0.3PG-E8-	E8 blank	188218.4	Terminal uridylyltransferase 4 OS=Homo sapiens OX=9606 GN=TUT4 PE=1 SV=1
H0Y8B3;Q9	3	1	22.8286	4.15E-08	1.36E-07	18.0385	1	F-0.3PG-E8-	E8 blank	59145.26	Adenosylhomocysteinase 3 (Fragment) OS=Homo sapiens OX=9606 GN=AHCYL2 PE=1 SV=1
Q8N2N9	4	1	22.6414	0.0454781	0.047711	1.41123	0.6137	E8 blank	P-0.3PG-E8-	155122.4	Ankyrin repeat domain-containing protein 36B OS=Homo sapiens OX=9606 GN=ANKRD36B PE=1 SV=4
A0A0A0MR	4	1	19.0237	2.53E-05	3.58E-05	2.76316	1	F-0.3PG-E8-	E8 blank	102744.3	Band 4.1-like protein 3 OS=Homo sapiens OX=9606 GN=EPB41L3 PE=1 SV=1
Q8NDV7	4	1	29.8896	0.0088165	0.009546	116.635	0.9117	F-0.3PG-E8-	E8 blank	211095.9	Trinucleotide repeat-containing gene 6A protein OS=Homo sapiens OX=9606 GN=TNRC6A PE=1 SV=2
P08910	3	1	16.7412	0.0001515	0.000192	9.19183	1	P-0.3PG-E8-	E8 blank	49113.39	Monoacylglycerol lipase ABHD2 OS=Homo sapiens OX=9606 GN=ABHD2 PE=1 SV=1
A0A1W2PR	4	1	46.9769	9.83E-08	2.83E-07	42.0107	1	P-0.3PG-E8-	E8 blank	57530.8	Glial fibrillary acidic protein OS=Homo sapiens OX=9606 GN=GFAP PE=1 SV=1
Q13360;K7	4	1	25.523	3.29E-07	7.26E-07	17.4764	1	P-0.3PG-E8-	E8 blank	56378.42	Zinc finger protein 177 OS=Homo sapiens OX=9606 GN=ZNF177 PE=1 SV=4
Q5JPF3;A0	4	1	32.4436	8.73E-11	2.00E-09	32.2805	1	P-0.3PG-E8-	E8 blank	201516.2	Ankyrin repeat domain-containing protein 36C OS=Homo sapiens OX=9606 GN=ANKRD36C PE=1 SV=3
Q13009	4	1	22.4686	9.07E-05	0.000118	1.84788	1	P-0.3PG-E8-	E8 blank	178820	Rho guanine nucleotide exchange factor TIAM1 OS=Homo sapiens OX=9606 GN=TIAM1 PE=1 SV=2
A0A087WW	6	1	39.8088	0.0020752	0.002338	1.56194	0.993	F-0.3PG-E8-	E8 blank	25214.08	Bromodomain adjacent to zinc finger domain protein 1A (Fragment) OS=Homo sapiens OX=9606 GN=BAZ1A PE
A0A2R8YD4	7	1	42.9861	4.42E-08	1.43E-07	3.44971	1	E8 blank	P-0.3PG-E8-	171503.1	DNA helicase (Fragment) OS=Homo sapiens OX=9606 GN=CHD4 PE=1 SV=1
A0A2R8Y8	8	1	47.8721	9.68E-06	1.45E-05	2.88094	1	E8 blank	F-0.3PG-E8-	190869	DNA helicase (Fragment) OS=Homo sapiens OX=9606 GN=CHD4 PE=1 SV=1
Q9NRL2	9	1	60.2804	0.1548226	0.157526	6.29499	0.335	E8 blank	P-0.3PG-E8-	180356.4	Bromodomain adjacent to zinc finger domain protein 1A OS=Homo sapiens OX=9606 GN=BAZ1A PE=1 SV=2
H0YCD1	4	1	22.7868	0.0001602	0.000202	1.63461	1	E8 blank	F-0.3PG-E8-	22830.69	Adenylate kinase 9 (Fragment) OS=Homo sapiens OX=9606 GN=AK9 PE=1 SV=8
Q3T906	2	1	8.9015	0.0019969	0.002266	1.54112	0.9936	E8 blank	P-0.3PG-E8-	144876.7	N-acetylglucosamine-1-phosphotransferase subunits alpha/beta OS=Homo sapiens OX=9606 GN=GNPTAB PE=
H0Y9K7;H0	3	1	23.4083	5.18E-06	8.22E-06	Infinity	1	E8 blank	F-0.3PG-E8-	26419.74	Splicing factor_ proline- and glutamine-rich (Fragment) OS=Homo sapiens OX=9606 GN=SFPQ PE=1 SV=1
A0A2R8Y5	3	1	12.2668	2.16E-08	8.40E-08	Infinity	1	P-0.3PG-E8-	E8 blank	195029.8	Tuberin OS=Homo sapiens OX=9606 GN=TSC2 PE=1 SV=1
Q14790	2	1	12.9515	0.0320876	0.033997	15.2212	0.6921	F-0.3PG-E8-	E8 blank	56132.58	Caspase-8 OS=Homo sapiens OX=9606 GN=CASP8 PE=1 SV=1
A0A1W2PS	2	1	23.4184	5.43E-08	1.72E-07	3.50406	1	E8 blank	P-0.3PG-E8-	7470.47	Glial fibrillary acidic protein (Fragment) OS=Homo sapiens OX=9606 GN=GFAP PE=1 SV=1
P19013	2	1	21.789	2.66E-08	9.82E-08	9.89731	1	P-0.3PG-E8-	E8 blank	56543.31	Keratin_ type II cytoskeletal 4 OS=Homo sapiens OX=9606 GN=KRT4 PE=1 SV=5
Q6KB66	2	1	21.6193	0.0001415	0.00018	277.781	1	P-0.3PG-E8-	E8 blank	51038.63	Keratin_ type II cytoskeletal 80 OS=Homo sapiens OX=9606 GN=KRT80 PE=1 SV=2
P08729	2	1	29.5478	2.87E-08	1.04E-07	2.82946	1	E8 blank	P-0.3PG-E8-	51442.77	Keratin_ type II cytoskeletal 7 OS=Homo sapiens OX=9606 GN=KRT7 PE=1 SV=5
Q13867;K7	2	1	13.6749	3.24E-07	7.18E-07	5.50428	1	F-0.3PG-E8-	E8 blank	53189.66	Bleomycin hydrolase OS=Homo sapiens OX=9606 GN=BLMH PE=1 SV=1
Q9Y247	2	1	16.5595	1.99E-12	2.30E-10	75.5404	1	P-0.3PG-E8-	E8 blank	38765.77	Protein FAM50B OS=Homo sapiens OX=9606 GN=FAM50B PE=1 SV=1
P23246	3	1	22.7121	1.77E-08	7.17E-08	3.03323	1	P-0.3PG-E8-	E8 blank	76263.6	Splicing factor_ proline- and glutamine-rich OS=Homo sapiens OX=9606 GN=SFPQ PE=1 SV=2
Q5T0T0	2	1	14.8592	8.45E-06	1.28E-05	3.0897	1	E8 blank	P-0.3PG-E8-	33877.4	E3 ubiquitin-protein ligase MARCHF8 OS=Homo sapiens OX=9606 GN=MARCHF8 PE=1 SV=1
A0A2R8Y6	2	1	16.9744	2.06E-05	2.95E-05	1.25949	1	P-0.3PG-E8-	E8 blank	177570.8	HECT-type E3 ubiquitin transferase OS=Homo sapiens OX=9606 GN=HECW2 PE=1 SV=1
A0A2R8Y4T	2	1	14.5546	0.0068327	0.007436	1.1199	0.9373	P-0.3PG-E8-	E8 blank	201147.7	Tensin-1 OS=Homo sapiens OX=9606 GN=TNS1 PE=1 SV=1

Q6ZN06	2	1	18.5992	7.75E-05	0.000102	2.51561	1	E8 blank	P-0.3PG-E8-	73887.67	Zinc finger protein 813 OS=Homo sapiens OX=9606 GN=ZNF813 PE=2 SV=2
A0A7I2V49	2	1	7.2158	5.70E-10	7.17E-09	24.7042	1	P-0.3PG-E8-	E8 blank	189706.5	DNA (cytosine-5)-methyltransferase OS=Homo sapiens OX=9606 GN=DNMT1 PE=1 SV=1
Q8WVE0	2	1	18.7245	5.72E-07	1.15E-06	27.4014	1	F-0.3PG-E8-	E8 blank	24904.81	EEF1A lysine methyltransferase 1 OS=Homo sapiens OX=9606 GN=EEF1AKMT1 PE=1 SV=1
Q9Y2J2	3	1	12.0064	0.0007905	0.000926	3.08095	0.9995	F-0.3PG-E8-	P-0.3PG-E8-	121533.2	Band 4.1-like protein 3 OS=Homo sapiens OX=9606 GN=EPB41L3 PE=1 SV=2
A0A087WY	3	1	19.3497	0.1446975	0.147928	5.60637	0.3493	F-0.3PG-E8-	E8 blank	98176.24	Zinc finger protein 107 OS=Homo sapiens OX=9606 GN=ZNF107 PE=1 SV=1
E9PK83;P25	2	1	15.956	0.0003348	0.000408	1.42429	1	E8 blank	P-0.3PG-E8-	17467.88	Rhombotin-1 OS=Homo sapiens OX=9606 GN=LMO1 PE=1 SV=1
C9JU31	2	1	8.3806	3.54E-06	5.85E-06	3.32429	1	E8 blank	P-0.3PG-E8-	51084.03	Coiled-coil domain-containing protein 136 OS=Homo sapiens OX=9606 GN=CCDC136 PE=1 SV=1
A0A7I2V44	1	1	4.5681	1.47E-11	8.49E-10	Infinity	1	E8 blank	P-0.3PG-E8-	34105.28	Kielin/chordin-like protein (Fragment) OS=Homo sapiens OX=9606 GN=KCP PE=1 SV=1
Q9BZM3	1	1	9.131	7.50E-08	2.25E-07	2.8227	1	E8 blank	P-0.3PG-E8-	32544.33	GS homeobox 2 OS=Homo sapiens OX=9606 GN=GSX2 PE=1 SV=2
Q5SW98	1	1	11.3816	2.64E-09	1.83E-08	9.90662	1	P-0.3PG-E8-	E8 blank	14477.95	Phosphatidylinositol 4-phosphate 3-kinase C2 domain-containing subunit beta (Fragment) OS=Homo sapiens O
C9J186;M0	1	1	11.028	1.02E-07	2.91E-07	46.5353	1	E8 blank	P-0.3PG-E8-	18775.69	Dermokine OS=Homo sapiens OX=9606 GN=DMKN PE=1 SV=1
E5RG86;E5	1	1	11.1114	4.26E-11	1.40E-09	57.4972	1	P-0.3PG-E8-	E8 blank	7175.22	Focal adhesion kinase 1 (Fragment) OS=Homo sapiens OX=9606 GN=PTK2 PE=1 SV=1
Q8N5R6;A0	2	1	11.4551	3.31E-05	4.58E-05	8.70734	1	P-0.3PG-E8-	E8 blank	107615.2	Coiled-coil domain-containing protein 33 OS=Homo sapiens OX=9606 GN=CCDC33 PE=1 SV=3
A0A590UJC	1	1	11.8644	1.70E-06	2.99E-06	6.6933	1	E8 blank	F-0.3PG-E8-	86034	85/88 kDa calcium-independent phospholipase A2 OS=Homo sapiens OX=9606 GN=PLA2G6 PE=1 SV=1
Q9HCL3	1	1	4.6741	8.71E-07	1.66E-06	1.71837	1	E8 blank	P-0.3PG-E8-	65231.2	Zinc finger protein 14 homolog OS=Homo sapiens OX=9606 GN=ZFP14 PE=1 SV=2
A0A087WZ	1	1	10.2996	4.85E-09	2.93E-08	19.0118	1	P-0.3PG-E8-	E8 blank	32899.75	Ribonuclease H2 subunit B OS=Homo sapiens OX=9606 GN=RNASEH2B PE=1 SV=2
Q3LI72	1	1	14.1438	2.34E-05	3.32E-05	17.3843	1	F-0.3PG-E8-	E8 blank	7852.332	Keratin-associated protein 19-5 OS=Homo sapiens OX=9606 GN=KRTAP19-5 PE=1 SV=1
P03915	1	1	11.6266	3.38E-07	7.39E-07	15.1749	1	P-0.3PG-E8-	E8 blank	67368.86	NADH-ubiquinone oxidoreductase chain 5 OS=Homo sapiens OX=9606 GN=MT-ND5 PE=1 SV=2
K7EQT8	1	1	10.495	1.41E-07	3.58E-07	2.15397	1	E8 blank	P-0.3PG-E8-	14264.82	HAUS augmin-like complex subunit 1 OS=Homo sapiens OX=9606 GN=HAUS1 PE=1 SV=1
D7R525;J3K	1	1	11.9846	7.26E-11	1.86E-09	719.453	1	P-0.3PG-E8-	E8 blank	24960.04	Mitogen-activated protein kinase OS=Homo sapiens OX=9606 GN=MAPK9 PE=1 SV=1
P23497	1	1	9.6558	6.26E-10	7.17E-09	33.1388	1	F-0.3PG-E8-	E8 blank	101557.3	Nuclear autoantigen Sp-100 OS=Homo sapiens OX=9606 GN=SP100 PE=1 SV=3
Q16760	1	1	0	8.30E-08	2.43E-07	74.8899	1	E8 blank	P-0.3PG-E8-	136692.8	Diacylglycerol kinase delta OS=Homo sapiens OX=9606 GN=DGKD PE=1 SV=4
A0A286YF1	1	1	4.9594	1.39E-09	1.26E-08	117.477	1	P-0.3PG-E8-	E8 blank	62570.75	Methylenetetrahydrofolate reductase OS=Homo sapiens OX=9606 GN=MTHFR PE=1 SV=1
Q14C86	1	1	10.335	7.38E-05	9.79E-05	1.42507	1	F-0.3PG-E8-	P-0.3PG-E8-	166462.9	GTPase-activating protein and VPS9 domain-containing protein 1 OS=Homo sapiens OX=9606 GN=GAPVD1 PE=
Q9UMW8	1	1	11.5444	1.27E-08	5.64E-08	26.2389	1	P-0.3PG-E8-	E8 blank	44037.36	Ubl carboxyl-terminal hydrolase 18 OS=Homo sapiens OX=9606 GN=USP18 PE=1 SV=1
H0YBZ9	1	1	6.0802	4.69E-09	2.93E-08	3.48346	1	F-0.3PG-E8-	E8 blank	8590.412	Survival motor neuron protein (Fragment) OS=Homo sapiens OX=9606 GN=SMN2 PE=1 SV=1
H0YFS2	1	1	10.9761	0.0022031	0.00247	234.572	0.992	P-0.3PG-E8-	E8 blank	26161.72	4F2 cell-surface antigen heavy chain (Fragment) OS=Homo sapiens OX=9606 GN=SLC3A2 PE=1 SV=1
P31273	1	1	11.2994	2.39E-09	1.70E-08	49.2782	1	F-0.3PG-E8-	E8 blank	27982.67	Homeobox protein Hox-C8 OS=Homo sapiens OX=9606 GN=HOXC8 PE=1 SV=2
P55259	1	1	9.0368	2.15E-12	2.30E-10	250.94	1	P-0.3PG-E8-	E8 blank	61134.38	Pancreatic secretory granule membrane major glycoprotein GP2 OS=Homo sapiens OX=9606 GN=GP2 PE=1 SV
Q7L3T8	1	1	5.5136	0.0001013	0.000131	3.75866	1	E8 blank	P-0.3PG-E8-	54289.26	Probable proline--tRNA ligase_ mitochondrial OS=Homo sapiens OX=9606 GN=PARS2 PE=1 SV=1
A0A1W2PP	1	1	5.0318	2.55E-11	1.09E-09	24.0634	1	P-0.3PG-E8-	E8 blank	21097.95	Uncharacterized protein KIAA1614 (Fragment) OS=Homo sapiens OX=9606 GN=KIAA1614 PE=1 SV=1
Q8WX93	2	1	7.704	1.03E-06	1.92E-06	53.3239	1	P-0.3PG-E8-	E8 blank	151932.7	Palladin OS=Homo sapiens OX=9606 GN=PALLD PE=1 SV=3
I6L8A6;Q99	2	1	10.1162	0.0005968	0.000708	3.27071	0.9998	P-0.3PG-E8-	E8 blank	103922.5	CtBP-interacting protein OS=Homo sapiens OX=9606 GN=RBBP8 PE=1 SV=1
Q14681	1	1	11.7198	2.20E-10	4.00E-09	8.24483	1	E8 blank	P-0.3PG-E8-	28812.24	BTB/POZ domain-containing protein KCTD2 OS=Homo sapiens OX=9606 GN=KCTD2 PE=1 SV=3
F8VU08;Q0	1	1	6.5138	1.73E-05	2.51E-05	2.83326	1	E8 blank	P-0.3PG-E8-	11367.14	Hematopoietically-expressed homeobox protein HHEX OS=Homo sapiens OX=9606 GN=HHEX PE=1 SV=1
G3V2Q1;P0	1	1	10.709	1.43E-11	8.49E-10	6.67637	1	P-0.3PG-E8-	E8 blank	33627.96	Heterogeneous nuclear ribonucleoproteins C1/C2 OS=Homo sapiens OX=9606 GN=HNRNPC PE=1 SV=1
P09871	1	1	10.1112	7.72E-10	8.11E-09	15.9881	1	P-0.3PG-E8-	E8 blank	78224.29	Complement C1s subcomponent OS=Homo sapiens OX=9606 GN=C1S PE=1 SV=1
E9PBE1;Q0	1	1	5.2057	3.86E-08	1.30E-07	3.71396	1	P-0.3PG-E8-	E8 blank	39814.18	Protein kinase C zeta type OS=Homo sapiens OX=9606 GN=PRKCZ PE=1 SV=1
K7ELH4;K7E	1	1	11.979	1.88E-11	8.59E-10	45.1736	1	P-0.3PG-E8-	E8 blank	4409.936	Intercellular adhesion molecule 3 (Fragment) OS=Homo sapiens OX=9606 GN=ICAM3 PE=1 SV=1
A0A1B0GTN	1	1	4.9309	3.69E-09	2.44E-08	12.5009	1	E8 blank	P-0.3PG-E8-	248764.8	Voltage-dependent L-type calcium channel subunit alpha OS=Homo sapiens OX=9606 GN=CACNA1D PE=1 SV=
H0Y3M0;H0	1	1	10.5586	7.05E-08	2.14E-07	8.93574	1	P-0.3PG-E8-	E8 blank	12707.79	Phosducin-like protein (Fragment) OS=Homo sapiens OX=9606 GN=PDCL PE=1 SV=1
Q12767	1	1	10.224	1.63E-09	1.39E-08	25.5184	1	P-0.3PG-E8-	E8 blank	153717.4	Transmembrane protein 94 OS=Homo sapiens OX=9606 GN=TMEM94 PE=1 SV=1
Q92628;D6	2	1	9.8827	0.0001613	0.000203	71.007	1	P-0.3PG-E8-	E8 blank	157127.2	Uncharacterized protein KIAA0232 OS=Homo sapiens OX=9606 GN=KIAA0232 PE=1 SV=5
Q38SD2	2	1	8.5083	2.10E-09	1.59E-08	Infinity	1	P-0.3PG-E8-	E8 blank	228529.7	Leucine-rich repeat serine/threonine-protein kinase 1 OS=Homo sapiens OX=9606 GN=LRRK1 PE=1 SV=3
Q13103	2	1	10.4025	3.37E-05	4.64E-05	2.60774	1	F-0.3PG-E8-	P-0.3PG-E8-	24622.87	Secreted phosphoprotein 24 OS=Homo sapiens OX=9606 GN=SPP2 PE=1 SV=1
A0A1P0B7D	1	1	14.0712	4.57E-07	9.49E-07	3.02092	1	P-0.3PG-E8-	E8 blank	48839.26	Mitogen-activated protein kinase OS=Homo sapiens OX=9606 GN=MAPK10 PE=1 SV=3
Q8N567	1	0	5.5075					---	---	31161.38	Zinc finger CCHC domain-containing protein 9 OS=Homo sapiens OX=9606 GN=ZCCHC9 PE=1 SV=2
F8WCT0	1	0	5.9008					---	---	3965.473	RNA-binding protein 6 OS=Homo sapiens OX=9606 GN=RBM6 PE=4 SV=1
M0QXV1	1	0	6.1018					---	---	16702.41	Inactive serine/threonine-protein kinase VRK3 (Fragment) OS=Homo sapiens OX=9606 GN=VRK3 PE=1 SV=1
A0A087WV	2	0	10.5959					---	---	10894.76	Latent-transforming growth factor beta-binding protein 4 (Fragment) OS=Homo sapiens OX=9606 GN=LTBP4 P

Q9NPI1	1	0	4.3639					---	---	74594.92	Bromodomain-containing protein 7 OS=Homo sapiens OX=9606 GN=BRD7 PE=1 SV=1
Q99453	1	0	5.1642					---	---	31906.15	Paired mesoderm homeobox protein 2B OS=Homo sapiens OX=9606 GN=PHOX2B PE=2 SV=2
A0A590UK	3	0	17.8508					---	---	125660.9	AP-3 complex subunit beta OS=Homo sapiens OX=9606 GN=AP3B2 PE=1 SV=1
Q8NEM1;E	3	0	24.0342					---	---	63679.94	Zinc finger protein 680 OS=Homo sapiens OX=9606 GN=ZNF680 PE=1 SV=2
Q8TAK5	1	0	10.6546					---	---	48649.67	GA-binding protein subunit beta-2 OS=Homo sapiens OX=9606 GN=GABPB2 PE=1 SV=1
Q9ULH7	1	0	5.301					---	---	118355.4	Myocardin-related transcription factor B OS=Homo sapiens OX=9606 GN=MRTFB PE=1 SV=3
Q92834;A0	5	0	18.8813					---	---	114413.4	X-linked retinitis pigmentosa GTPase regulator OS=Homo sapiens OX=9606 GN=RPGR PE=1 SV=2
Q92625	1	0	7.3746					---	---	124077.5	Ankyrin repeat and SAM domain-containing protein 1A OS=Homo sapiens OX=9606 GN=ANKS1A PE=1 SV=4
E7EWZ1;P1	1	0	4.8394					---	---	47464.46	Myb-related protein A OS=Homo sapiens OX=9606 GN=MYBL1 PE=1 SV=1
A0A7I2V44	1	0	4.8206					---	---	40671.21	Cathepsin B OS=Homo sapiens OX=9606 GN=CTSB PE=1 SV=1
K7ESF4;Q9	1	0	5.1502					---	---	23184.77	Glucose-6-phosphate isomerase (Fragment) OS=Homo sapiens OX=9606 PE=4 SV=1
P35908;H0	4	0	26.2697					---	---	65718.1	Keratin_ type II cytoskeletal 2 epidermal OS=Homo sapiens OX=9606 GN=KRT2 PE=1 SV=2
E9PSA5;H0	1	0	11.8666					---	---	26170.52	Alpha-ketoglutarate-dependent dioxygenase alkB homolog 3 (Fragment) OS=Homo sapiens OX=9606 GN=ALKB
B1AKL4;Q9	1	0	5.4562					---	---	105892.1	Eukaryotic translation initiation factor 4E transporter OS=Homo sapiens OX=9606 GN=EIF4ENIF1 PE=1 SV=1
Q13367;A0	3	0	13.9849					---	---	119686.8	AP-3 complex subunit beta-2 OS=Homo sapiens OX=9606 GN=AP3B2 PE=1 SV=2
A0A1W2PC	1	0	12.7832					---	---	54484.93	Squalene synthase OS=Homo sapiens OX=9606 GN=FDFT1 PE=1 SV=1
A0A087WT	1	0	5.5721					---	---	20476.03	Oxysterol-binding protein-related protein 2 (Fragment) OS=Homo sapiens OX=9606 GN=OSBPL2 PE=1 SV=1
O60765	1	0	4.927					---	---	71061.74	Zinc finger protein 354A OS=Homo sapiens OX=9606 GN=ZNF354A PE=1 SV=2
G3V2N3;P5	1	0	6.1126					---	---	22557.11	Paired mesoderm homeobox protein 1 OS=Homo sapiens OX=9606 GN=PRRX1 PE=1 SV=1
Q9UJW2	1	0	5.2686					---	---	55973.24	Tubulointerstitial nephritis antigen OS=Homo sapiens OX=9606 GN=TINAG PE=2 SV=3
Q9Y2K1	1	0	5.0767					---	---	84069.2	Zinc finger and BTB domain-containing protein 1 OS=Homo sapiens OX=9606 GN=ZBTB1 PE=1 SV=3
P04259;P0	3	0	20.8708					---	---	60352.17	Keratin_ type II cytoskeletal 6B OS=Homo sapiens OX=9606 GN=KRT6B PE=1 SV=5
P38159	2	0	9.5021					---	---	42331.91	RNA-binding motif protein_ X chromosome OS=Homo sapiens OX=9606 GN=RBMX PE=1 SV=3
H0Y9M1	1	0	12.2202					---	---	27850.45	Spermatogenesis-associated protein 20 (Fragment) OS=Homo sapiens OX=9606 GN=SPATA20 PE=1 SV=1
Q96BN6	1	0	13.4766					---	---	65131.53	Primary cilium assembly protein FAM149B1 OS=Homo sapiens OX=9606 GN=FAM149B1 PE=1 SV=2
H0Y4M6;H	2	0	16.6718					---	---	36010.6	Guanine nucleotide exchange factor DBS (Fragment) OS=Homo sapiens OX=9606 GN=MCF2L PE=1 SV=1
H0YDV1	1	0	5.9567					---	---	5765.462	Zinc finger protein 195 (Fragment) OS=Homo sapiens OX=9606 GN=ZNF195 PE=1 SV=1
C9J0A5;C9J	1	0	4.3241					---	---	16392.3	E3 ubiquitin protein ligase (Fragment) OS=Homo sapiens OX=9606 GN=RNF20 PE=1 SV=1
Q86W92	1	0	4.0217					---	---	114594.6	Liprin-beta-1 OS=Homo sapiens OX=9606 GN=PPFIBP1 PE=1 SV=2
Q8IUB9	1	0	5.1447					---	---	9406.862	Keratin-associated protein 19-1 OS=Homo sapiens OX=9606 GN=KRTAP19-1 PE=1 SV=2
F5H5B4;Q1	1	0	13.7346					---	---	49770.36	Non-specific serine/threonine protein kinase OS=Homo sapiens OX=9606 GN=STK4 PE=1 SV=1
G3V2J9	1	0	11.039					---	---	145198.9	Tubulin polyglutamylase TTLL5 OS=Homo sapiens OX=9606 GN=TTLL5 PE=1 SV=1
H7C0P3	2	0	10.6955					---	---	16449.93	Nuclear receptor corepressor 2 (Fragment) OS=Homo sapiens OX=9606 GN=NCOR2 PE=1 SV=1
P48960	1	0	4.2585					---	---	94663.74	Adhesion G protein-coupled receptor E5 OS=Homo sapiens OX=9606 GN=ADGRE5 PE=1 SV=4
Q96PM5	2	0	9.9168					---	---	31764.25	RING finger and CHY zinc finger domain-containing protein 1 OS=Homo sapiens OX=9606 GN=RCHY1 PE=1 SV=
D6W5U7;Q	1	0	8.5172					---	---	140432.5	Cohesin subunit SA-3 OS=Homo sapiens OX=9606 GN=STAG3 PE=1 SV=1
M0R297;Q	1	0	5.451					---	---	17183.23	Zinc finger protein 677 (Fragment) OS=Homo sapiens OX=9606 GN=ZNF677 PE=1 SV=1
P55000	1	0	10.812					---	---	11813.27	Secreted Ly-6/uPAR-related protein 1 OS=Homo sapiens OX=9606 GN=SLURP1 PE=1 SV=2
A6PWM2;C	1	0	5.76					---	---	26384.14	Protein disulfide isomerase CRELD2 (Fragment) OS=Homo sapiens OX=9606 GN=CRELD2 PE=1 SV=1
A0A087X2F	1	0	11.9152					---	---	5913.826	Adenomatous polyposis coli protein (Fragment) OS=Homo sapiens OX=9606 GN=APC PE=4 SV=1
Q9BUQ8	1	0	10.1872					---	---	95924.82	Probable ATP-dependent RNA helicase DDX23 OS=Homo sapiens OX=9606 GN=DDX23 PE=1 SV=3
Q9UBS5	2	0	8.7919					---	---	109404	Gamma-aminobutyric acid type B receptor subunit 1 OS=Homo sapiens OX=9606 GN=GABBR1 PE=1 SV=1
A0A7I2V2X	1	0	12.7934					---	---	57929.96	60 kDa chaperonin OS=Homo sapiens OX=9606 GN=HSPD1 PE=1 SV=1
B4E2Q0;H0	1	0	4.9915					---	---	105853.8	Calcium-transporting ATPase OS=Homo sapiens OX=9606 GN=ATP2C1 PE=1 SV=1
C9J102;C9J	1	0	14.2568					---	---	15538.11	Histone deacetylase (Fragment) OS=Homo sapiens OX=9606 GN=HDAC7 PE=1 SV=1
F8VWZ5	1	0	13.1262					---	---	16980.93	H2.0-like homeobox protein (Fragment) OS=Homo sapiens OX=9606 GN=HLX PE=1 SV=1
Q99593	1	0	11.1198					---	---	58224.23	T-box transcription factor TBX5 OS=Homo sapiens OX=9606 GN=TBX5 PE=1 SV=2
Q9BRV8	1	0	10.2498					---	---	23892.13	Suppressor of IKBKE 1 OS=Homo sapiens OX=9606 GN=SIKE1 PE=1 SV=1
A0A0C4DG	1	0	10.1976					---	---	14315.24	Prolyl hydroxylase EGLN2 OS=Homo sapiens OX=9606 GN=EGLN2 PE=1 SV=1
A2A2D0;P1	1	0	13.2326					---	---	9793.226	Stathmin (Fragment) OS=Homo sapiens OX=9606 GN=STMN1 PE=1 SV=8

E9PRV7;P25	1	0	6.4642					---	---	47221.43	G protein-coupled receptor kinase OS=Homo sapiens OX=9606 GN=GRK2 PE=1 SV=1
Q13615	1	0	4.5881					---	---	136242.3	Myotubularin-related protein 3 OS=Homo sapiens OX=9606 GN=MTMR3 PE=1 SV=3
A0A494C0J	1	0	5.9524					---	---	60908.46	Zinc finger protein 136 OS=Homo sapiens OX=9606 GN=ZNF136 PE=1 SV=1
H7BXE3	1	0	5.1165					---	---	49076.26	SAFB-like transcription modulator (Fragment) OS=Homo sapiens OX=9606 GN=SLTM PE=1 SV=1
A0A2U3TZN	1	0	12.346					---	---	29513.92	Uncharaterized LOC112694756 OS=Homo sapiens OX=9606 GN=LOC112694756 PE=4 SV=1
A6NJL1	1	0	8.251					---	---	56681.41	Zinc finger and SCAN domain-containing protein 5B OS=Homo sapiens OX=9606 GN=ZSCAN5B PE=1 SV=1
A0A1B0GU	1	0	13.0756					---	---	13329.43	Proline-rich transmembrane protein 2 OS=Homo sapiens OX=9606 GN=PRRT2 PE=4 SV=1
Q9HCR9	1	0	4.7606					---	---	105892.4	Dual 3'_5'-cyclic-AMP and -GMP phosphodiesterase 11A OS=Homo sapiens OX=9606 GN=PDE11A PE=1 SV=2
Q16832;Q5	1	0	5.4754					---	---	97648.94	Discoidin domain-containing receptor 2 OS=Homo sapiens OX=9606 GN=DDR2 PE=1 SV=2
H0Y3V3	1	0	4.9618					---	---	52939.03	Adhesion G protein-coupled receptor L2 (Fragment) OS=Homo sapiens OX=9606 GN=ADGRL2 PE=1 SV=1
H0YMX5;O	1	0	5.0829					---	---	17370.03	Homeobox protein Meis2 (Fragment) OS=Homo sapiens OX=9606 GN=MEIS2 PE=1 SV=2
R4GMU5	1	0	4.5664					---	---	6397.702	Dipeptidyl peptidase 2 (Fragment) OS=Homo sapiens OX=9606 GN=DPP7 PE=1 SV=1
Q10570	1	0	4.7486					---	---	162138.7	Cleavage and polyadenylation specificity factor subunit 1 OS=Homo sapiens OX=9606 GN=CPSF1 PE=1 SV=2
Q8N9K5	1	0	4.5127					---	---	64326.94	Zinc finger protein 565 OS=Homo sapiens OX=9606 GN=ZNF565 PE=2 SV=2
A0A0B4J23	1	0	16.4016					---	---	23492.52	Immunoglobulin lambda-like polypeptide 5 OS=Homo sapiens OX=9606 GN=IGLL5 PE=1 SV=1
